# Can Dermoscopy Be Used to Predict if a Melanoma Is In Situ or Invasive?

**DOI:** 10.5826/dpc.1103a79

**Published:** 2021-05-20

**Authors:** Sam Polesie, Edvin Jergéus, Martin Gillstedt, Hannah Ceder, Johan Dahlén Gyllencreutz, Julia Fougelberg, Eva Johansson Backman, Jenna Pakka, Oscar Zaar, John Paoli

**Affiliations:** 1 Department of Dermatology and Venereology, Institute of Clinical Sciences, Sahlgrenska Academy, University of Gothenburg, Gothenburg, Sweden; 2 Region Västra Götaland, Sahlgrenska University Hospital, Department of Dermatology and Venereology, Gothenburg, Sweden

**Keywords:** Breslow thickness, Dermoscopy, Inter Observer Variability, Melanoma, Projections and Predictions

## Abstract

**Background:**

The preoperative prediction of whether melanomas are invasive or in situ can influence initial management.

**Objectives:**

This study evaluated the accuracy rate, interobserver concordance, sensitivity and specificity in determining if a melanoma is invasive or in situ, as well as the ability to predict invasive melanoma thickness based on clinical and dermoscopic images.

**Methods:**

In this retrospective, single-center investigation, 7 dermatologists independently reviewed clinical and dermoscopic images of melanomas to predict if they were invasive or in situ and, if invasive, their Breslow thickness. Fleiss’ and Cohen’s kappa (κ) were used for interobserver concordance and agreement with histopathological diagnosis.

**Results:**

We included 184 melanomas (110 invasive and 74 in situ). Diagnostic accuracy ranged from 67.4% to 76.1%. Accuracy rates for in situ and invasive melanomas were 57.5% (95% confidence interval [CI], 53.1%–61.8%) and 81.7% (95% CI, 78.8%–84.4%), respectively. Interobserver concordance was moderate (κ = 0.47; 95% CI, 0.44–0.51). Sensitivity for predicting invasiveness ranged from 63.6% to 91.8% for 7 observers, while specificity was 32.4%–82.4%. For all correctly predicted invasive melanomas, agreement between predictions and correct thickness over or under 1.0 mm was moderate (κ = 0.52; 95% CI, 0.45–0.58). All invasive melanomas incorrectly predicted by any observer as in situ had a thickness <1.0 mm. All 32 melanomas >1.0 mm were correctly predicted to be invasive by all observers.

**Conclusions:**

Accuracy rates for predicting thick melanomas were excellent, melanomas inaccurately predicted as in situ were all thin, and interobserver concordance for predicting in situ or invasive melanomas was moderate. Preoperative dermoscopy of suspected melanomas is recommended for choosing appropriate surgical margins.

## Introduction

The clinical diagnosis of melanoma is most often straightforward, as suggested by the fact that the majority are detected by the patient and not the dermatologist [1]. Nonetheless, predicting preoperatively whether a melanoma is in situ or invasive is often challenging, since dermoscopic features for in situ melanomas are similar to those of thin invasive melanomas [2,3]. Such a preoperative prediction is important since it helps select optimal clinical surgical margins for the diagnostic excision. International guidelines recommend a 1- to 3-mm margin when invasive melanoma is suspected, to facilitate an expected subsequent wide local excision and, potentially, a sentinel lymph node biopsy [4,5]. However, if in situ melanoma is the primary clinical suspicion, the lesion may be excised directly with the recommended 5-mm surgical margin [6], conveniently avoiding an unnecessary second surgical procedure. For well-demarcated lesions not located in chronic sun-damaged skin, this pragmatic approach to optimizing the choice of surgical margins during the diagnostic excision is nowadays common practice in many Swedish centers.

In a recent investigation, Lallas et al [3] analyzed dermoscopic features of 1,285 lesions including 325 in situ melanomas and 102 invasive melanomas with a Breslow thickness <0.75 mm. In multivariable analyses including in situ melanomas, nevi, seborrheic keratosis, basal cell carcinoma, Bowens disease and Reed nevi the study identified atypical network, regression, irregular hyperpigmented areas, prominent skin markings and angulated lines as positive dermoscopic markers for in situ melanomas. When findings for invasive and in situ melanomas were compared, a multicomponent global pattern and blue-white veil were indicative of invasive melanomas, whereas extensive regression was the only indicator of in situ melanoma [3]. In an earlier study, Silva et al [7] concluded that thin melanomas (Breslow thickness <1.0 mm) tend to have asymmetry in 2 axes, ≥3 colors, atypical dots or globules, atypical network or streaks, while in situ melanomas tend to have ≤2 colors. Blue-white veil and milky red areas were both associated with invasive disease [7]. While the abovementioned results are important in a research setting, their usefulness in a real-life clinical setting is more uncertain.

The primary objective of this investigation was to explore dermatologists’ accuracy in discriminating in situ melanomas from invasive melanomas.

## Methods

We performed a retrospective, single-center investigation, including primary melanomas with available clinical and dermoscopic images obtained from our department. The study was approved by the regional ethics review board in Gothenburg (approval number, D283-18). All dermoscopic images included in the study (Supplementary File) had been acquired using a polarized light setting. Melanomas that were previously biopsied or with images of suboptimal quality were excluded. All tumors were histopathologically confirmed by the hospital’s dermatopathology team. To avoid recall bias by the observers, the study only included cases that were diagnosed during 2016 (ie, >3 years prior to study initiation). The images were independently reviewed by 2 residents and 5 board-certified dermatologists. All observers had received training in dermoscopy (ie, had attended at least one dermoscopy course) apart from their daily use of dermoscopy in routine clinical practice.

### Setting

The dermatologists’ primary objective was to determine whether melanomas were invasive or in situ. If considered invasive, they estimated the Breslow thickness by selecting one of the following intervals: 0–1.0; 1.01–2.0; 2.01–4.0 and >4.0 mm.

### Outcomes

The primary outcome measure was the accuracy rate in predicting in situ vs invasive melanoma, compared to the pathology report. The secondary outcomes were: (i) accuracy rate of the majority response (ie, decision of ≥4 observers), (ii) interobserver concordance, (iii) sensitivity and specificity with respect to invasive melanoma, and (iv) ability to classify invasive melanoma thickness greater or less than 1.0 mm.

### Statistical Analysis

All data were analyzed using R version 3.5.3 (https://www.r-project.org/). To measure interobserver concordance between the 7 observers and agreement compared to the pathology report, Fleiss’ and Cohen’s kappa (κ) were used [8,9]. The interobserver agreement was interpreted as poor (≤0), slight (>0 to 0.20), fair (>0.2 to 0.4), moderate (>0.4 to 0.6), substantial (>0.6 to 0.8) or almost perfect (>0.8).

## Results

Overall, 184 melanomas (110 invasive and 74 in situ melanomas) from 177 patients were included. The patients’ median age was 67 years (interquartile range, 57–77 years) and 83 (47%) were female. Melanomas located on the trunk as well as the upper and lower extremities constituted 88% of all lesions (n = 162).

Overall diagnostic accuracy rates for all observers are presented in Table 1. The individual accuracy rates (ie, proportion of predictions that agreed with the pathology report) ranged from 67.4% to 76.1%.

**Figure 1:**
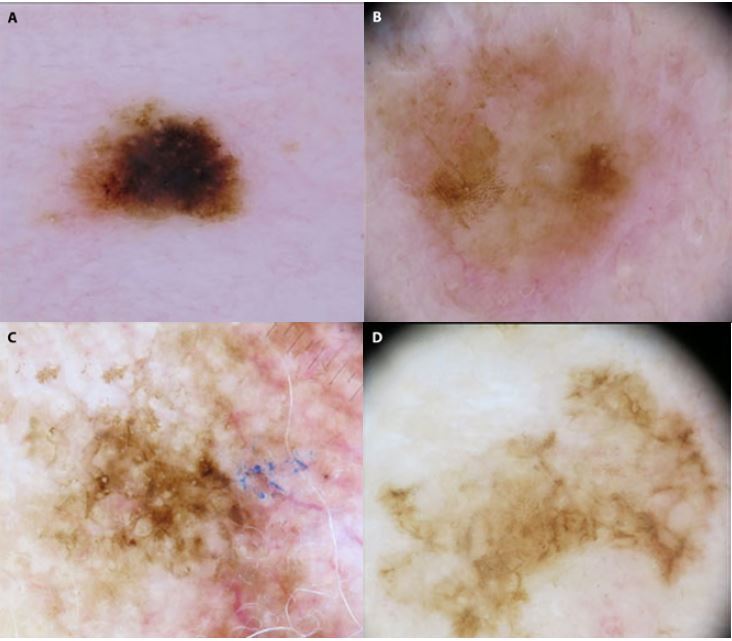
Dermoscopic images of the 4 invasive melanomas that were predicted as in situ by all observers. All 4 lesions had a Breslow thickness ≤0.5 mm. Lesions (a) and (b) were located on the upper extremities, and lesions (c) and (d) were located on the scalp and arm, respectively.

When the majority decision for each lesion was considered separately, the accuracy rate was 75.0%. Among the 7 dermatologists, the interobserver concordance was moderate [κ = 0.47, 95% confidence interval (CI), 0.44–0.51]. For 69 and 17 lesions, there was complete consensus among the observers that the lesions were invasive and in situ, respectively. For the invasive melanomas, the consensus response was true for 63 cases (91.3%). The corresponding number among in situ melanomas was 13 (76.5%). Four melanomas were incorrectly predicted by all observers as in situ, but all had a Breslow thickness ≤0.5 mm (Figure 1). Furthermore, all invasive melanomas that were predicted by any observer as in situ melanomas were <1.0 mm thick. The thickest melanoma classified as in situ by any observer had a Breslow thickness of 0.9 mm. None of the lesions erroneously classified as in situ by any observer was ulcerated. Conversely, all melanomas >1.0 mm in thickness (n = 32) were correctly classified as invasive melanomas by all observers.

**Figure 2:**
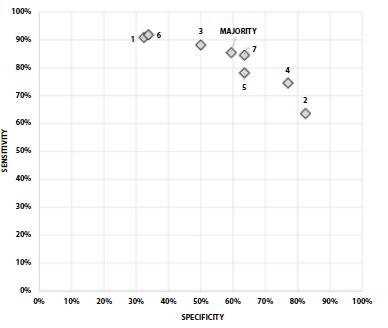
Sensitivity and specificity for the classification of invasive melanomas, for 7 dermatologists and for the majority decision (ie, decision of ≥4 observers).

The sensitivity for predicting invasive melanoma ranged from 63.6% to 91.8% for the 7 observers (Figure 2). The specificity ranged from 32.4% to 82.4% for the 7 observers.

The Breslow thickness predictions were further analyzed for all invasive melanomas that were correctly classified (629 of 770 predictions, 81.7%). For these assessments, the agreement (κ) between the observers and the pathology report for a Breslow thickness over or under 1.0 mm was 0.52 (95% CI, 0.45–0.58). For the invasive melanomas where all observers agreed on the correct diagnosis (n = 63), the interobserver concordance for predicting a Breslow thickness over or under 1.0 mm was also moderate (κ = 0.54; 95% CI, 0.49–0.59).

## Discussion

We report an accuracy rate ranging from 67.4% to 76.1% for predicting whether a melanoma was invasive or in situ based on clinical and dermoscopic images. For invasive lesions specifically, concordance for classification of Breslow thickness over or under 1.0 mm was moderate compared to the pathology report. On the other hand, invasive melanomas >1.0 mm in thickness were all correctly classified as invasive by all observers; all invasive melanomas predicted incorrectly as in situ had a Breslow thickness ≤0.9 mm, and none of these erroneously classified lesions were ulcerated. It is important and reassuring that all melanomas predicted to be in situ were histopathologically confirmed as either in situ or thin melanomas with a good prognosis.

A strength of this investigation is that we included more observers and melanomas than previous investigations performed in similar settings [10–12]. While other investigations primarily focused on describing specific dermoscopic findings in invasive and in situ melanomas, our aim was to assess their usefulness in making precise diagnostic predictions. Moreover, the dermoscopic images evaluated in this study are all shared in an online resource (Supplementary File), which is exceedingly rare. Polarized light setting was used for all cases in this study, but different dermatoscopes and camera setups were used when acquiring the images. Specific meta-data, including age, skin type and relevant medical history were intentionally omitted, as the primary aim was to address the diagnostic accuracy based on images themselves. Inclusion of these details could potentially have improved the accuracy rates [12].

For the diagnosis of primary melanomas, dermoscopic evaluation outperforms evaluation with the naked eye [13]. Nevertheless, the technique is more accurate when interpreted with the patient present, rather than analyzing dermoscopy images alone [14,15]. We also acknowledge the artificial setup in this investigation. Making predictions such as these in real-life may have given different results. Although the observers were not blinded to the fact that all lesions were either invasive or in situ melanomas, the study setting mimicked the very common scenario faced by physicians once a decision has been made to excise a melanocytic lesion in order to rule out melanoma. It is also important to underline that this investigation only included observers affiliated with a single academic center. As such, the evaluations could be expected to be more uniform since the same reference for learning has often been applied. Lastly, the experience of the dermoscopist carrying out this type of evaluation is crucial, and the validity of our findings should be further assessed in multicenter studies at an international level.

The incidence of melanoma has been rising drastically in Europe during the past decades, and this incidence increase has especially been driven by more cases of thin invasive melanoma and in situ melanoma [16]. In fact, nowadays, more than half of all melanomas in Sweden are diagnosed in their in situ growth phase [17]. Rises in melanoma incidence and our quest to diagnose melanomas as early as possible will inevitably result in large numbers of excisions worldwide. Although experts in dermoscopy excise substantially fewer suspicious skin lesions to find a single melanoma, a recent meta-analysis including 29 studies and almost 400,000 excisions showed that the overall number of pigmented lesions that need to be excised to find a melanoma is 9.7 [18]. With increased knowledge about the early diagnosis of melanoma, we have to find pragmatic solutions to minimize the number of unnecessary excisions of benign lesions. Furthermore, studies have shown that 2-mm surgical margins, as internationally recommended for lesions suspected to be melanoma, result in incomplete excision rates of up to 24% [19,20].

It therefore seems reasonable to use a slightly larger surgical margin when in situ melanoma is suspected, to minimize the risk of incomplete diagnostic excisions, while also reducing the number of unnecessary wide local excisions afterwards. We therefore suggest that a 5-mm surgical margin is used when experienced dermoscopy users have a strong preoperative suspicion of in situ melanoma and no suspicion of invasiveness. We understand that this suggestion might be controversial for some, since this could lead to a slightly larger scar than necessary in cases when the excised lesion turned out to be a nevus. However, for atypical melanocytic lesions on surgically less-challenging body parts with a high dermoscopic suspicion of in situ melanoma, this treatment strategy could potentially lower the incomplete excision rates, minimize the number of unnecessary subsequent wide local excisions, lessen the morbidity for the patient and increase cost-effectiveness. Moreover, it would not limit the ability of performing a wide local excision with a 1-cm margin in the eventual case of a thin invasive melanoma (<1 mm Breslow thickness) being confirmed, nor are there any studies showing that a 5-mm margin would affect the possibility of performing a sentinel lymph node biopsy in the rare case of a thicker invasive melanoma being confirmed.

Although dermoscopy cannot perfectly predict if a melanoma is invasive or in situ, preoperative dermoscopic assessment of suspected melanomas should be recommended to choose the most appropriate surgical margins.

## Supplementary Information



## Figures and Tables

**Table 1:** Diagnostic Accuracy, by Lesion Characteristic^1^

Characteristic	Lesions, n (%)	Accuracy, % (95% CI)
In situ melanoma	74 (40)	57.5 (53.1–61.8)
Invasive melanoma	110 (60)	81.7 (78.8–84.4)
Breslow thickness^2^ (mm)		
<0.50	38 (21)	60.2 (54.0–66.1)
0.51 – 0.80	27 (15)	84.1 (78.1–89.0)
0.81 – 1.00	13 (7)	94.5 (87.6–98.2)
1.01 – 2.00	18 (10)	100 (97.1–100)
2.01 – 4.00	9 (5)	100 (94.3–100)
>4.00	5 (3)	100 (90.0–100)

CI = confidence interval.

1 Values are aggregated responses for all 7 observers.

2 For 110 invasive melanomas.

## References

[1] McGuire ST, Secrest AM, Andrulonis R, Ferris LK (2011). Surveillance of patients for early detection of melanoma: patterns in dermatologist vs patient discovery. Arch Dermatol.

[2] Pizzichetta MA, Argenziano G, Talamini R (2001). Dermoscopic criteria for melanoma in situ are similar to those for early invasive melanoma. Cancer.

[3] Lallas A, Longo C, Manfredini M (2018). Accuracy of dermoscopic criteria for the diagnosis of melanoma in situ. JAMA Dermatol.

[4] Swedish guidelines for malignant melanoma [Article in Swedish].

[5] Garbe C, Amaral T, Peris K, European Dermatology Form (EDF); European Association of Dermato-Oncology (EADO); European Organization for Research and Treatment of Cancer (EORTC) (2020). European consensus-based interdisciplinary guideline for melanoma. Part 2: Treatment - Update 2019. Eur J Cancer.

[6] Swetter SM, Tsao H, Bichakjian CK (2019). Guidelines of care for the management of primary cutaneous melanoma. J Am Acad Dermatol.

[7] Silva VP, Ikino JK, Sens MM, Nunes DH, Di Giunta G (2013). Dermoscopic features of thin melanomas: a comparative study of melanoma in situ and invasive melanomas smaller than or equal to 1mm. An Bras Dermatol.

[8] Fleiss JL (1971). Measuring nominal scale agreement among many raters. Psychological Bulletin.

[9] Cohen J (1960). A coefficient of agreement for nominal scales. Educational and Psychological Measurement.

[10] Carli P, de Giorgi V, Palli D, Giannotti V, Giannotti B (2000). Preoperative assessment of melanoma thickness by ABCD score of dermatoscopy. J Am Acad Dermatol.

[11] Tan E, Oakley A, Soyer HP (2010). Interobserver variability of teledermoscopy: an international study. Br J Dermatol.

[12] Argenziano G, Fabbrocini G, Carli P, De Giorgi V, Delfino M (1999). Clinical and dermatoscopic criteria for the preoperative evaluation of cutaneous melanoma thickness. J Am Acad Dermatol.

[13] Vestergaard ME, Macaskill P, Holt PE, Menzies SW (2008). Dermoscopy compared with naked eye examination for the diagnosis of primary melanoma: a meta-analysis of studies performed in a clinical setting. Br J Dermatol.

[14] Dinnes J, Deeks JJ, Chuchu N, Cochrane Skin Cancer Diagnostic Test Accuracy Group (2018). Dermoscopy, with and without visual inspection, for diagnosing melanoma in adults. Cochrane Database Syst Rev.

[15] Carli P, De Giorgi V, Argenziano G, Palli D, Giannotti B (2002). Pre-operative diagnosis of pigmented skin lesions: in vivo dermoscopy performs better than dermoscopy on photographic images. J Eur Acad Dermatol Venereol.

[16] Sacchetto L, Zanetti R, Comber H (2018). Trends in incidence of thick, thin and in situ melanoma in Europe. Eur J Cancer.

[17] The National Board of Health and Welfare Statistics on Cancer Incidence 2018 [Article in Swedish].

[18] Petty AJ, Ackerson B, Garza R (2020). Meta-analysis of number needed to treat for diagnosis of melanoma by clinical setting. J Am Acad Dermatol.

[19] Bakhai M, Hopster D, Wakeel R (2010). A retrospective study comparing the accuracy of prehistology diagnosis and surgical excision of malignant melanomas by general practitioners and hospital specialists. Clin Exp Dermatol.

[20] Murchie P, Sinclair E, Lee AJ (2011). Primary excision of cutaneous melanoma: does the location of excision matter. Br J General Practice.

