# Supplementary material for: Can Dermoscopy Be Used to Predict if a Melanoma Is In Situ or Invasive?
Source: Dermatol Pract Concept. 2021 May 20;11(3):e2021079. doi: 10.5826/dpc.1103a79 (PMC8172039; doi:10.5826/dpc.1103a79)
Supplement: Supplementary file 1 [file Supplementary_file_Polesie_et_al.pdf]

Case number 1

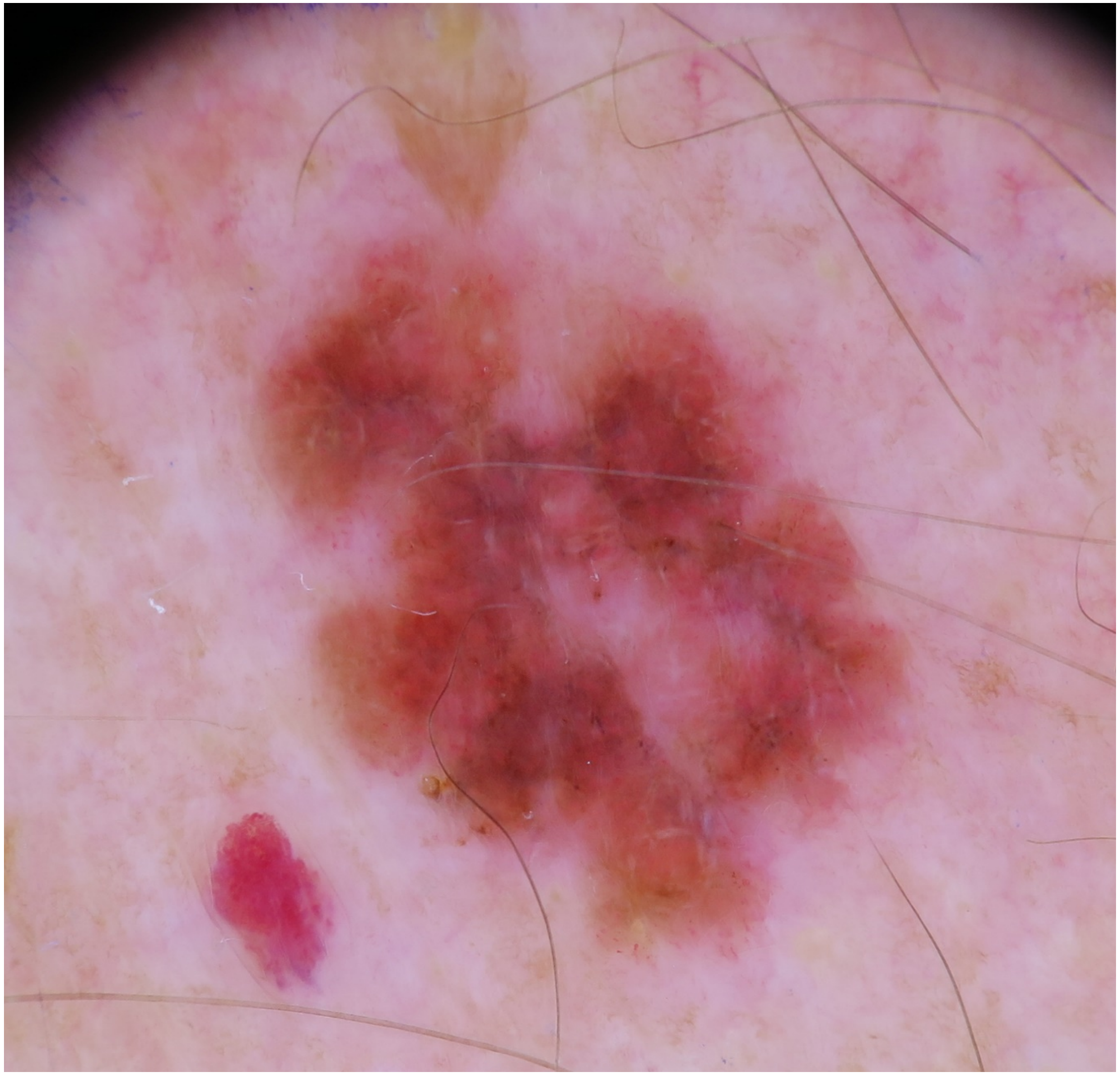

Location: Trunk

Invasive) Breslow interval: 0.1-0.5 mm

Case number 2

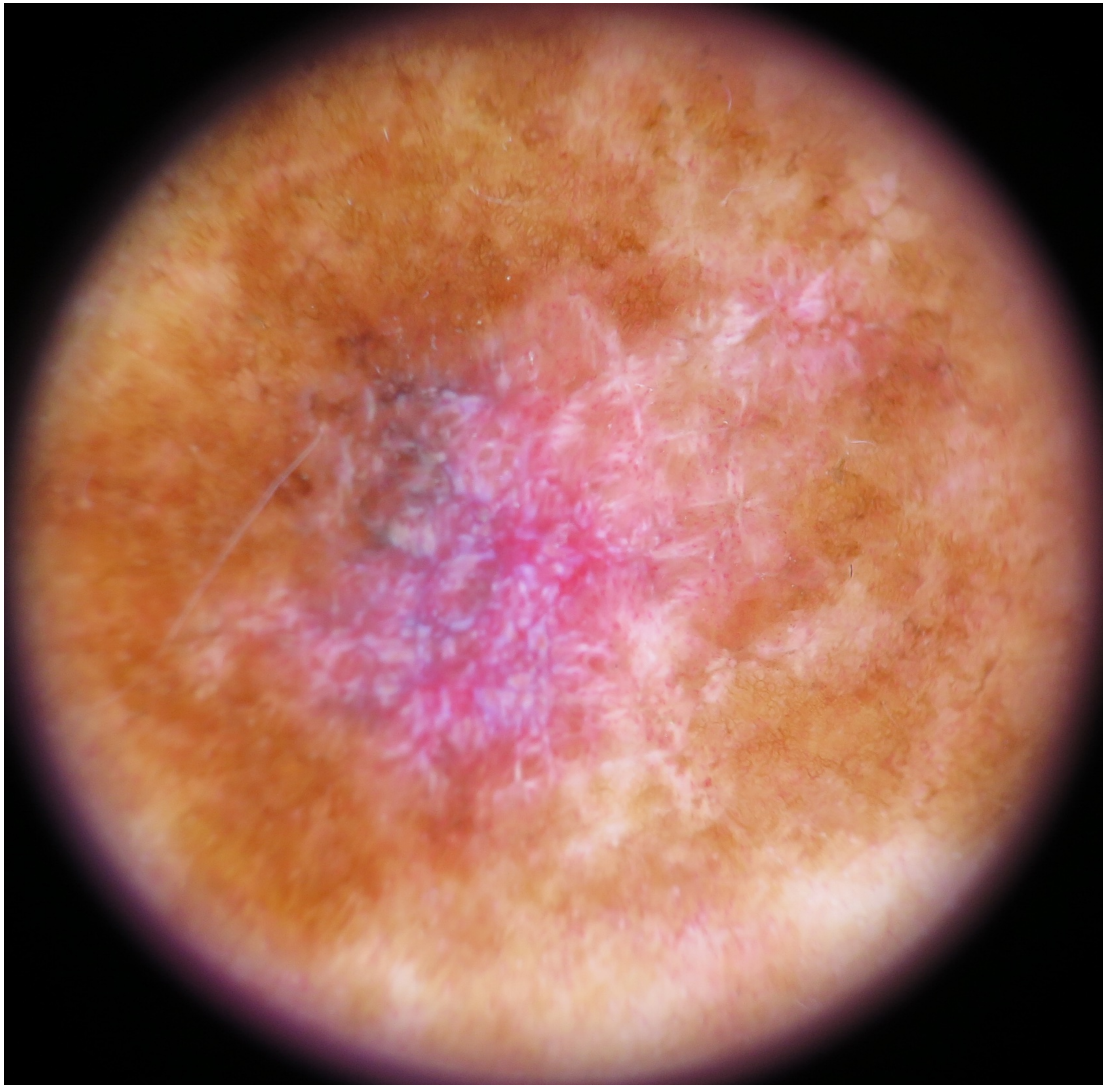

Location: Lower extremity

Invasive) Breslow interval: 0.1-0.5 mm

Case number 3

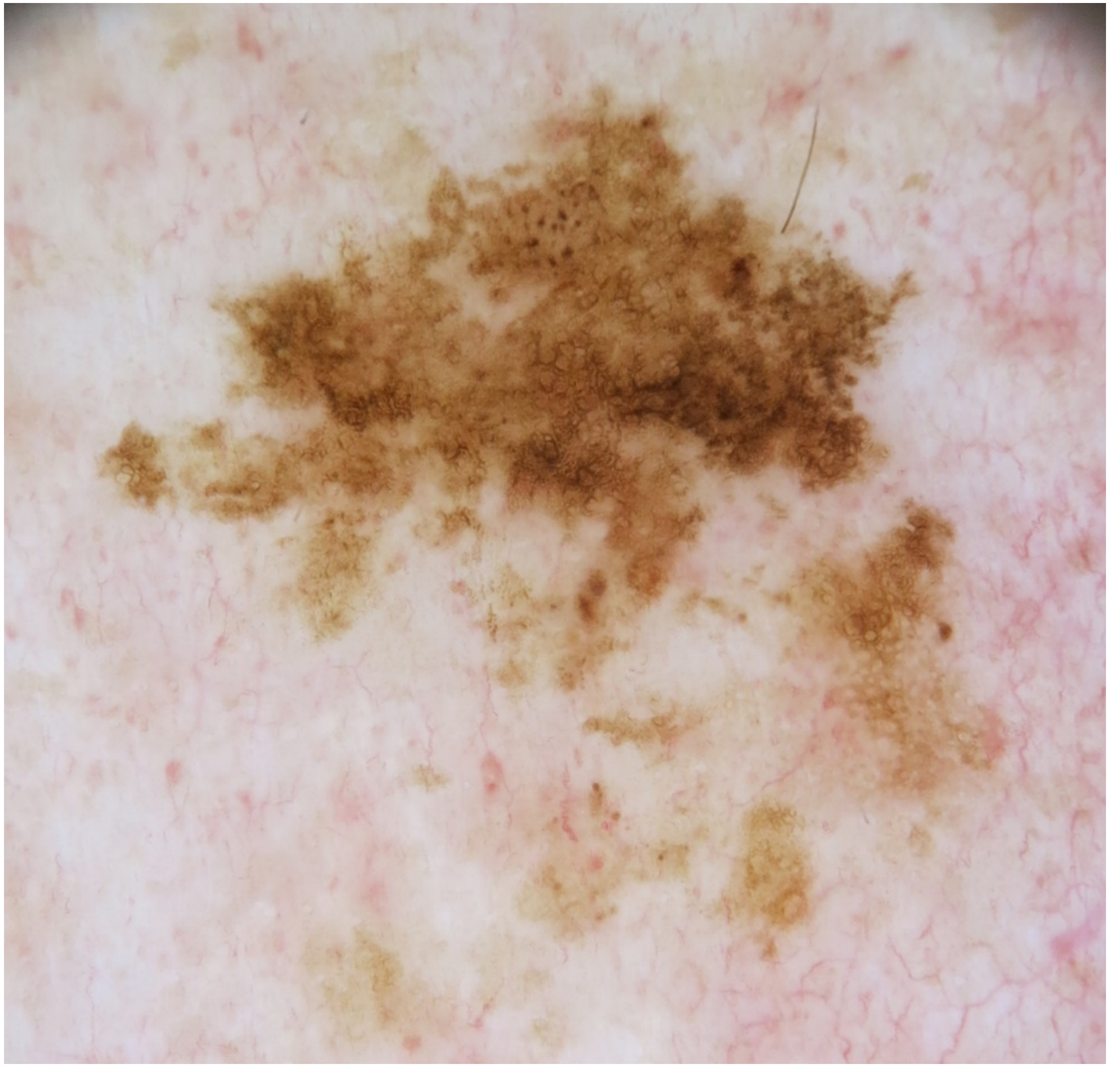

Location: Lower extremity

In situ melanoma

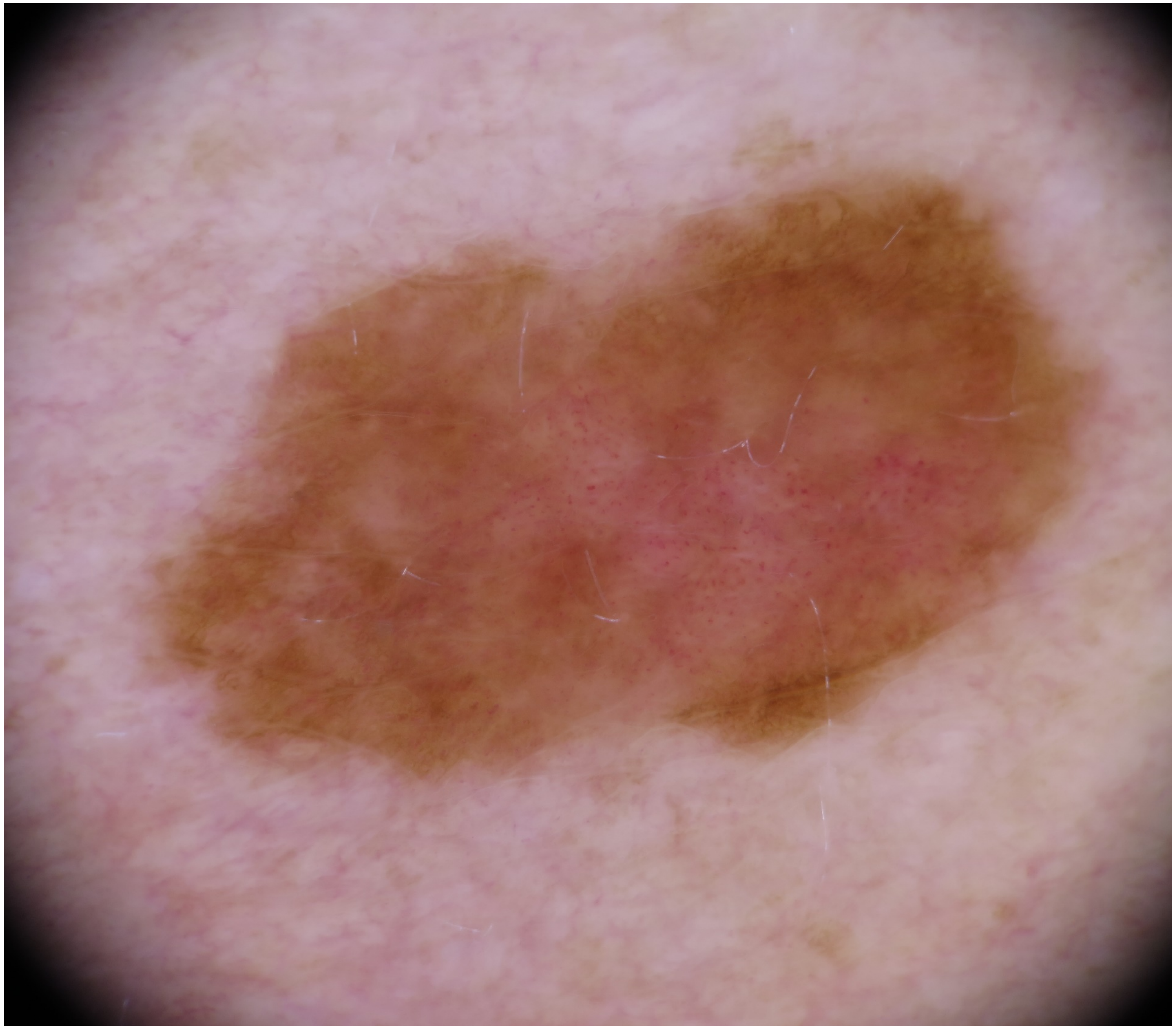

Location: Lower extremity

In situ melanoma

Case number 5

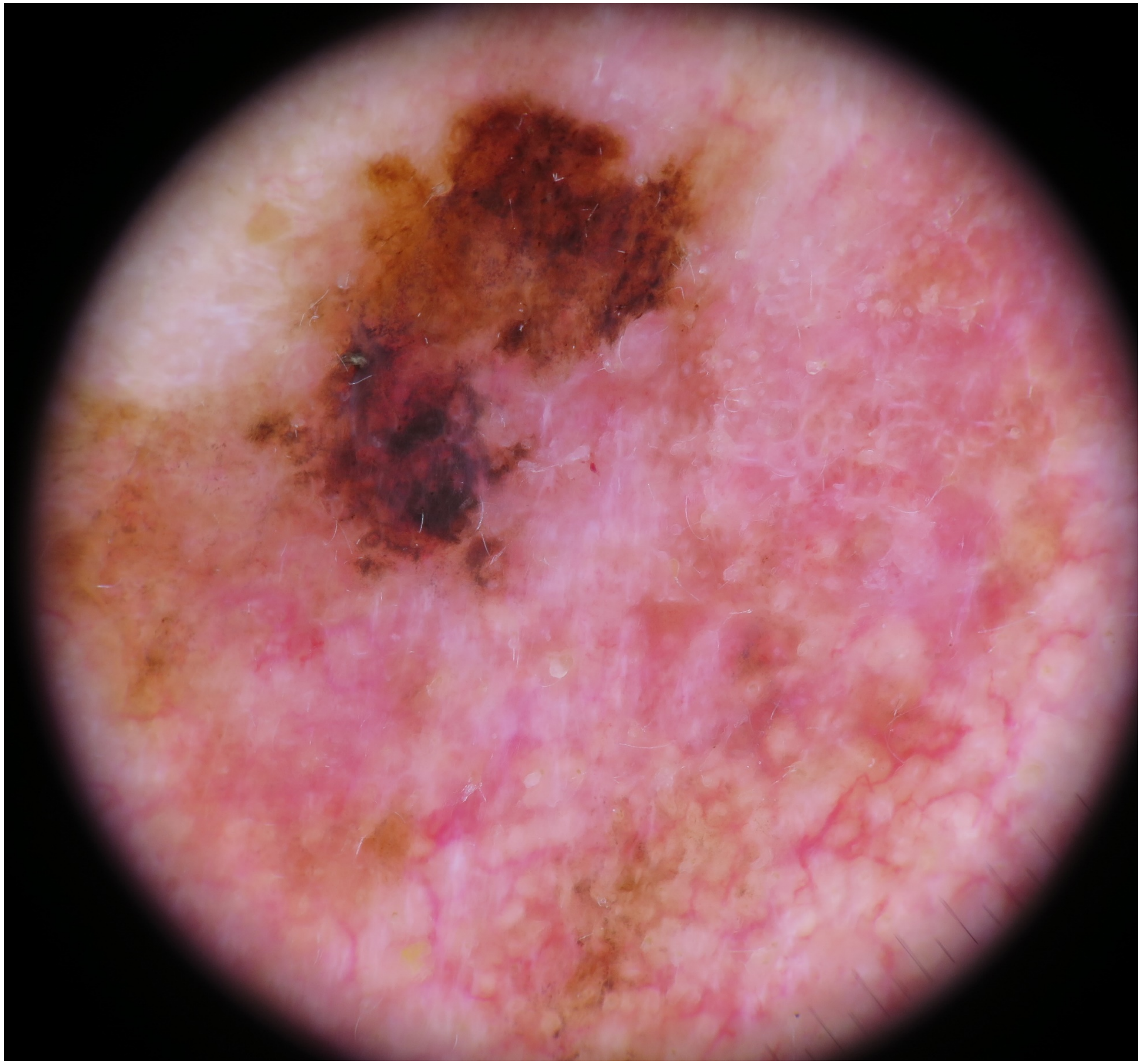

Location: Face

Invasive) Breslow interval: 0.6-0.8 mm

Case number 6

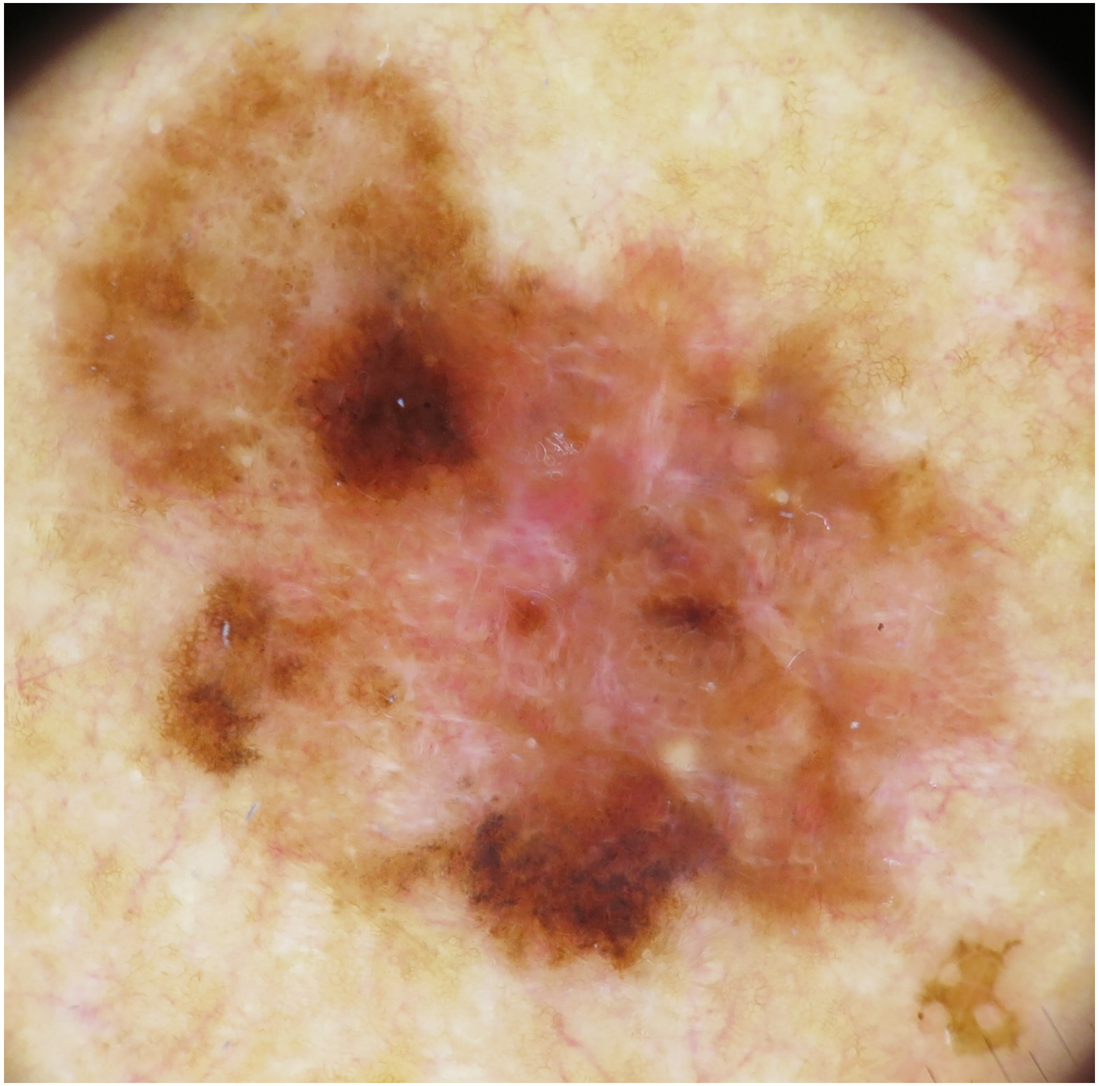

Location: Upper extremity

Invasive) Breslow interval: 0.6-0.8 mm

Case number 7

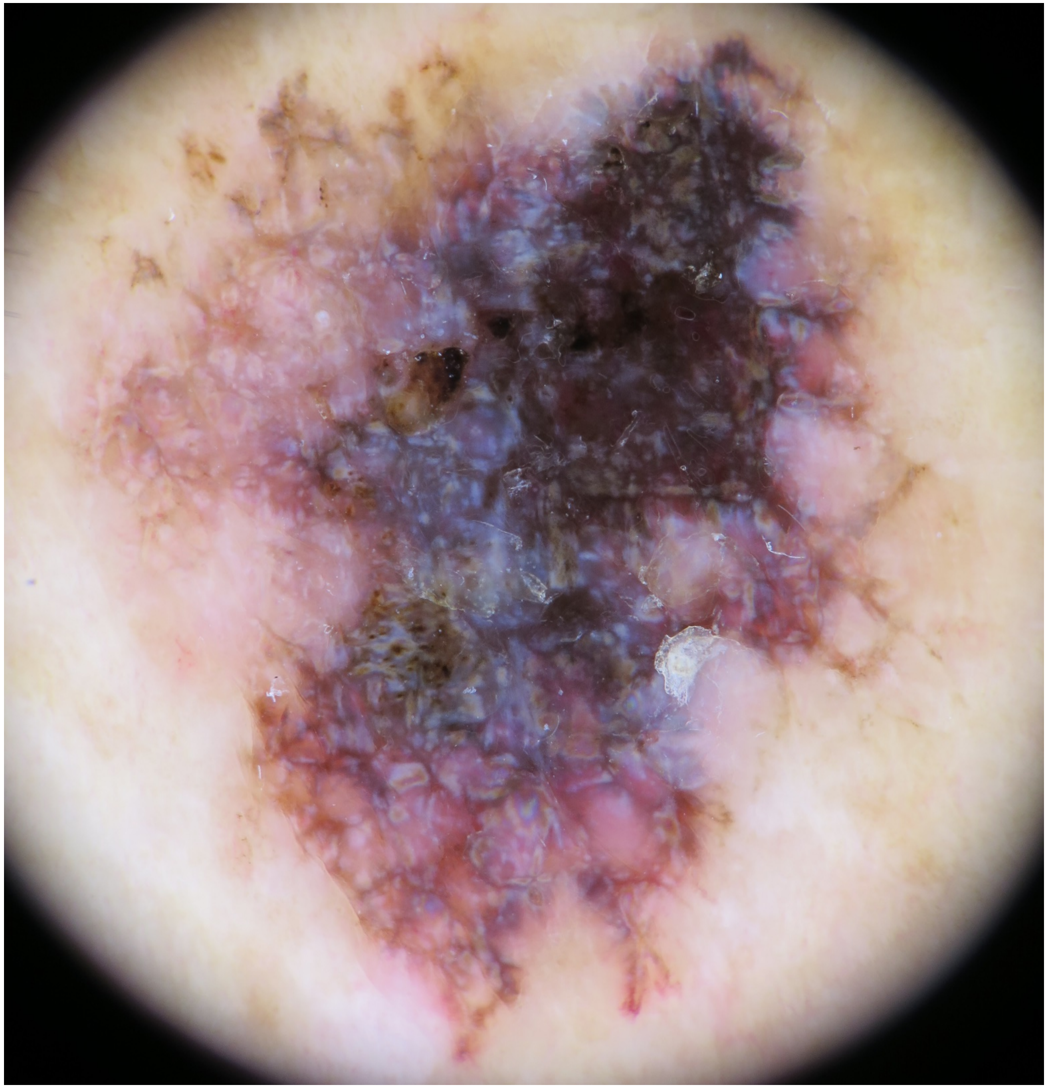

Location: Lower extremity

Invasive) Breslow interval: 1.1-2.0 mm

Case number 8

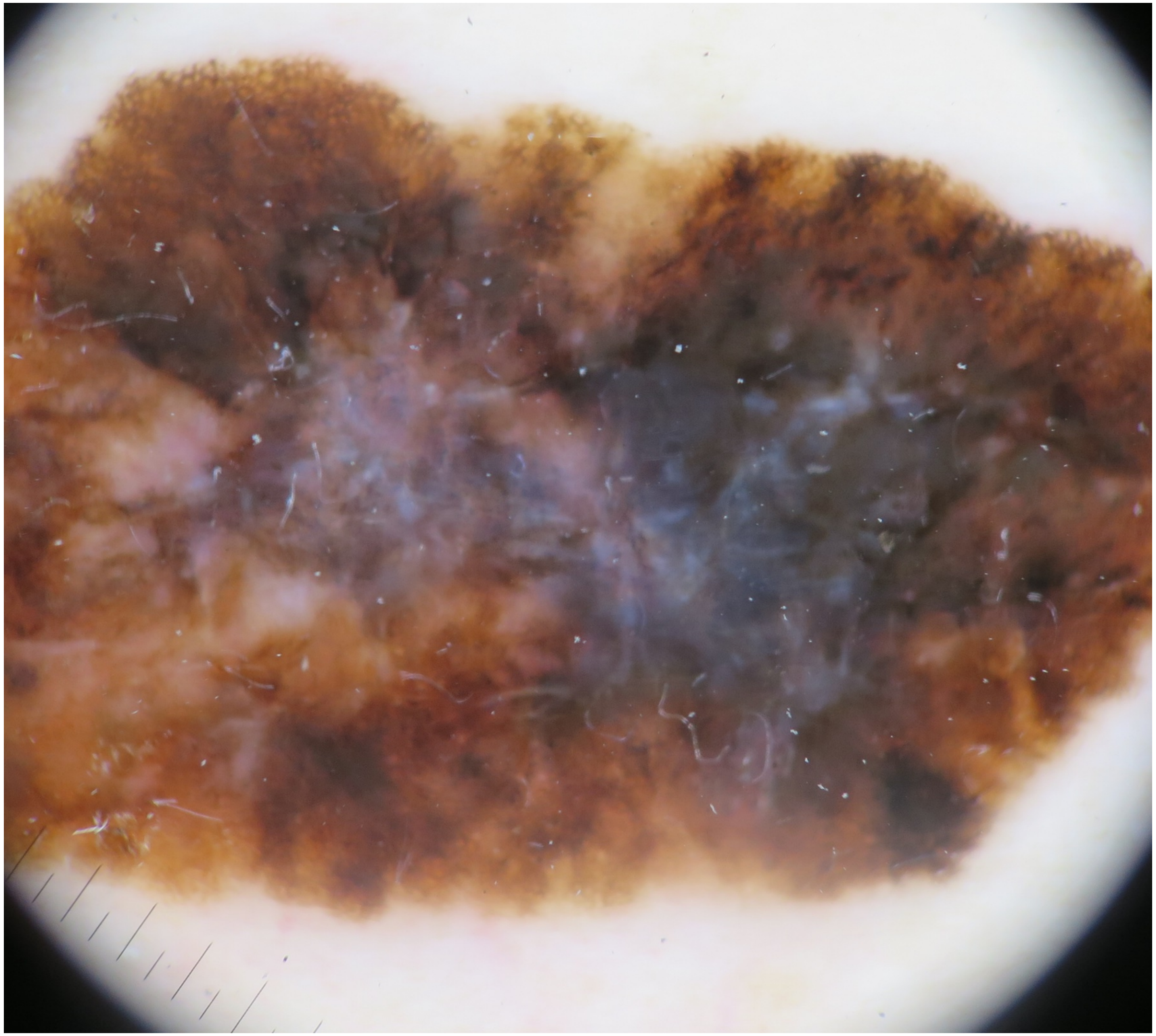

Location: Trunk

Invasive) Breslow interval: 0.9-1.0 mm

Case number 9

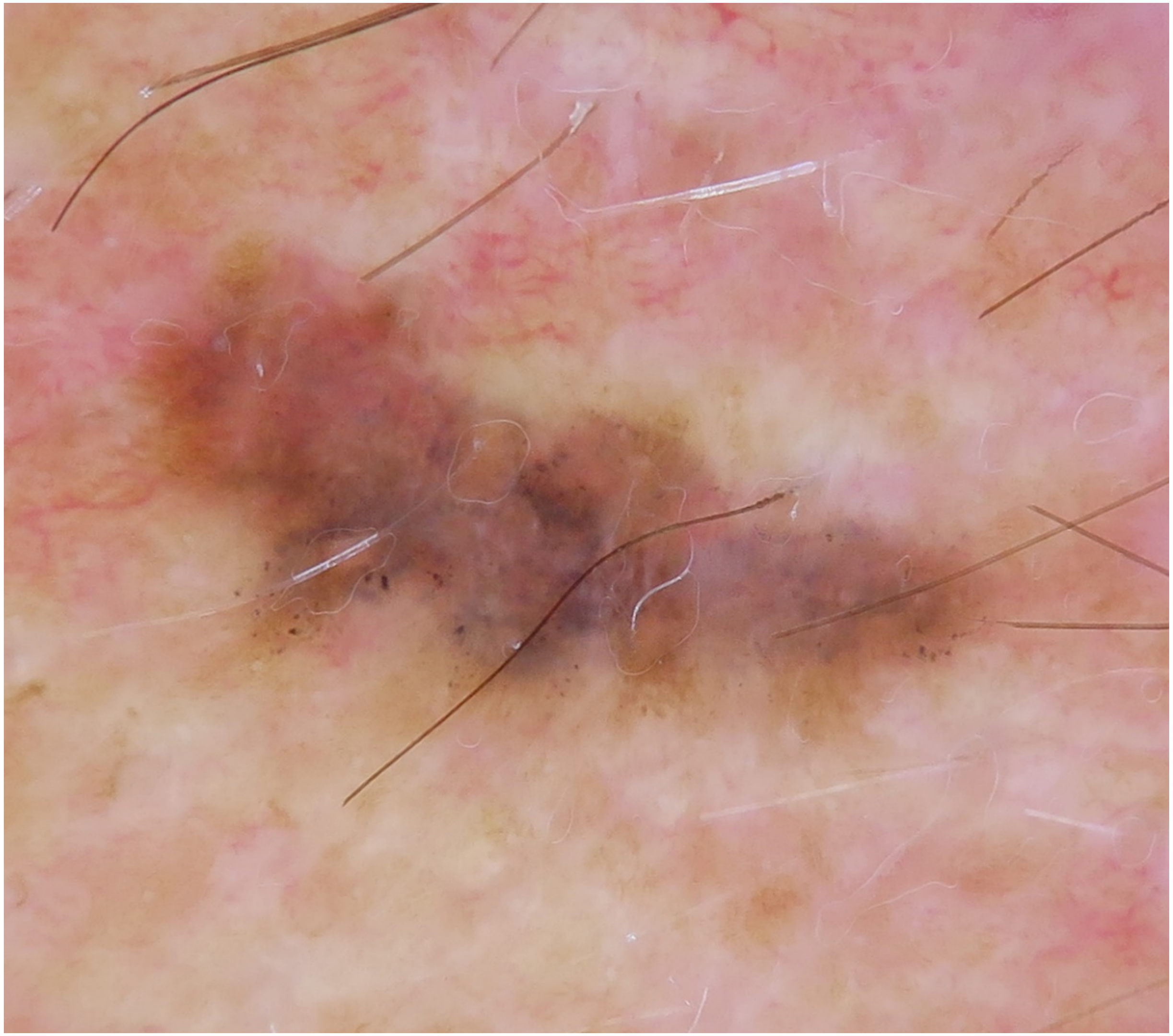

Location: Neck

Invasive) Breslow interval: 0.1-0.5 mm

Case number 10

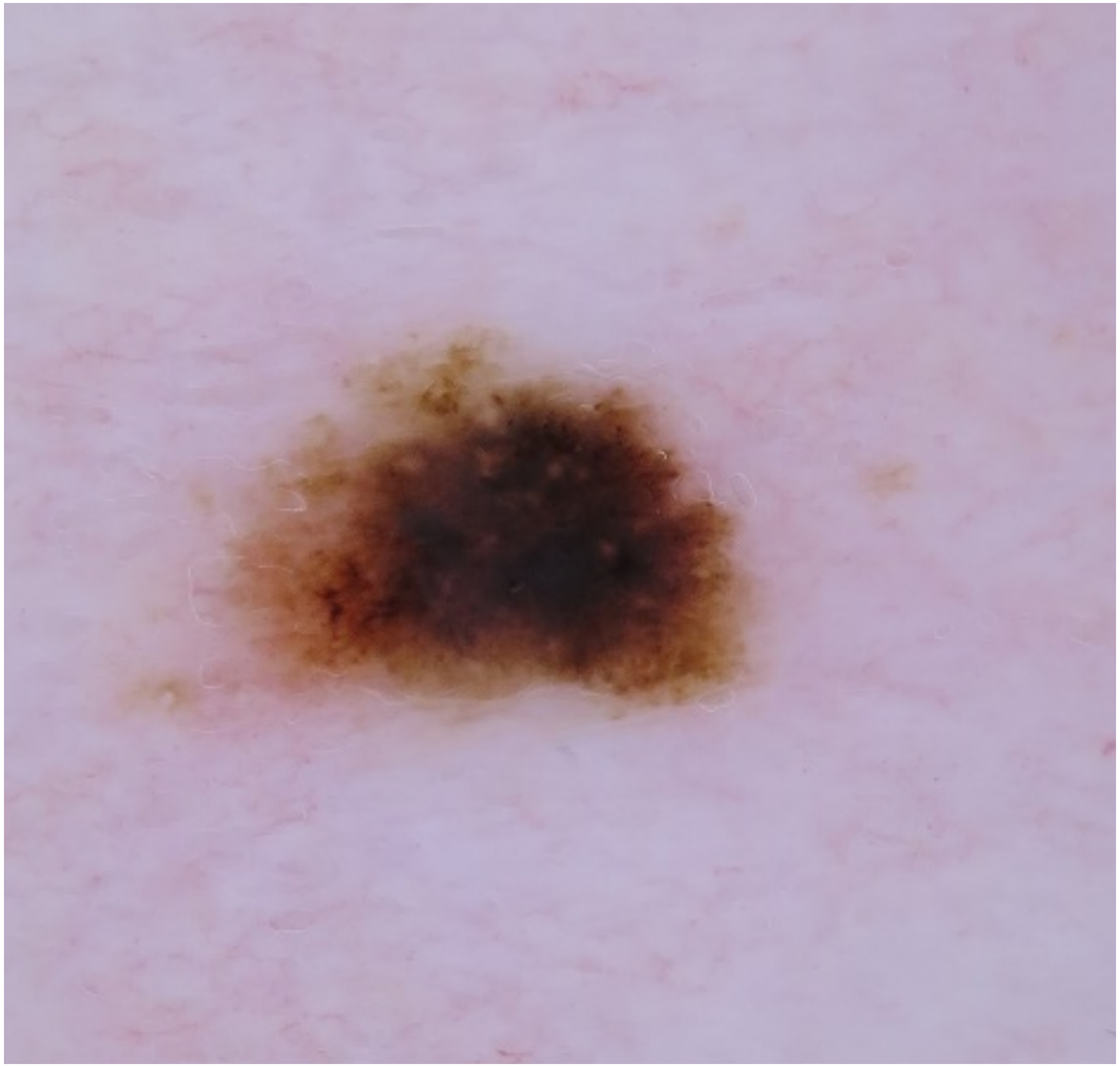

Location: Upper extremity

Invasive) Breslow interval: 0.1-0.5 mm

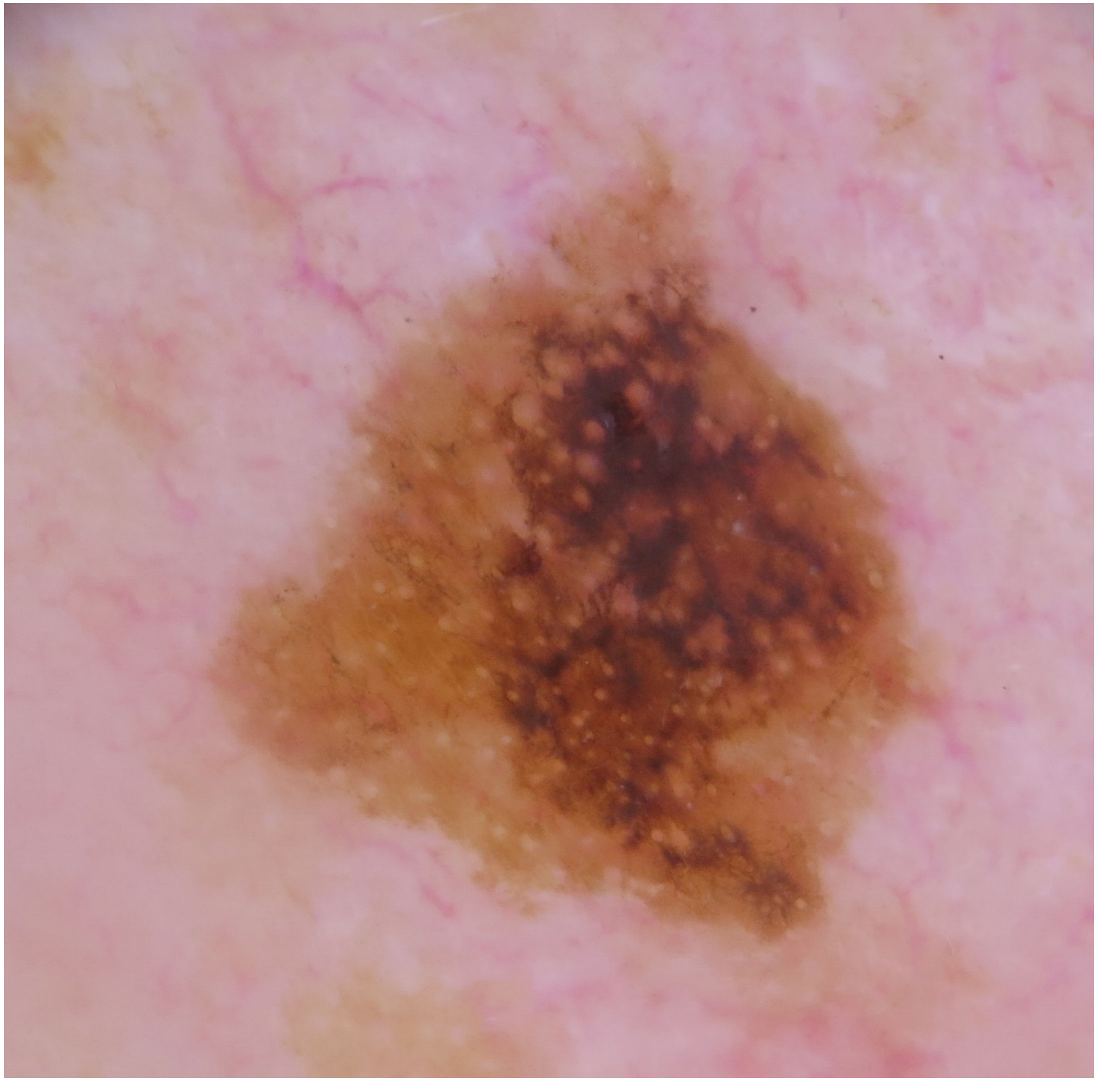

Location: Face

In situ melanoma

Case number 12

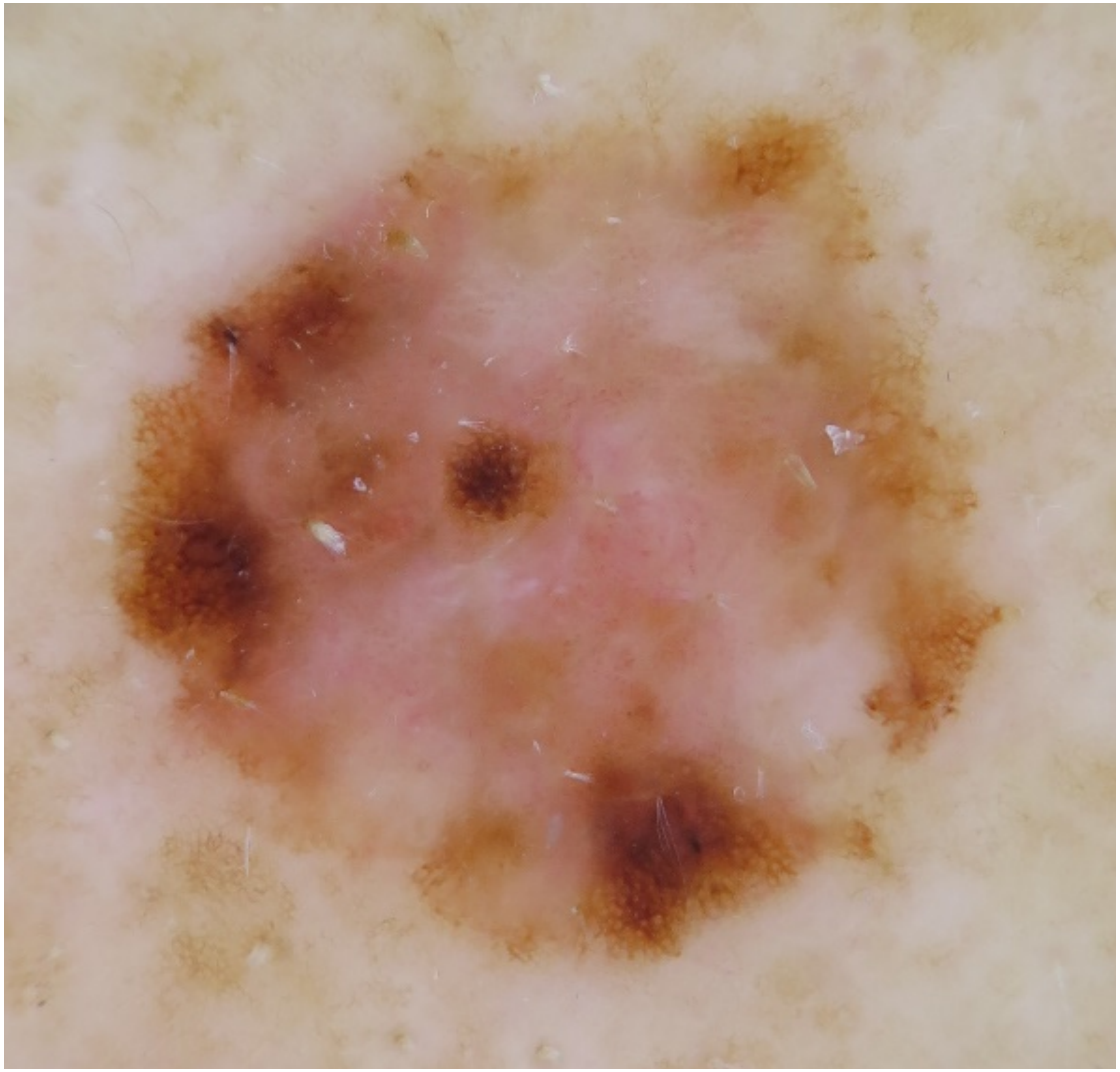

Location: Trunk

In situ melanoma

Case number 13

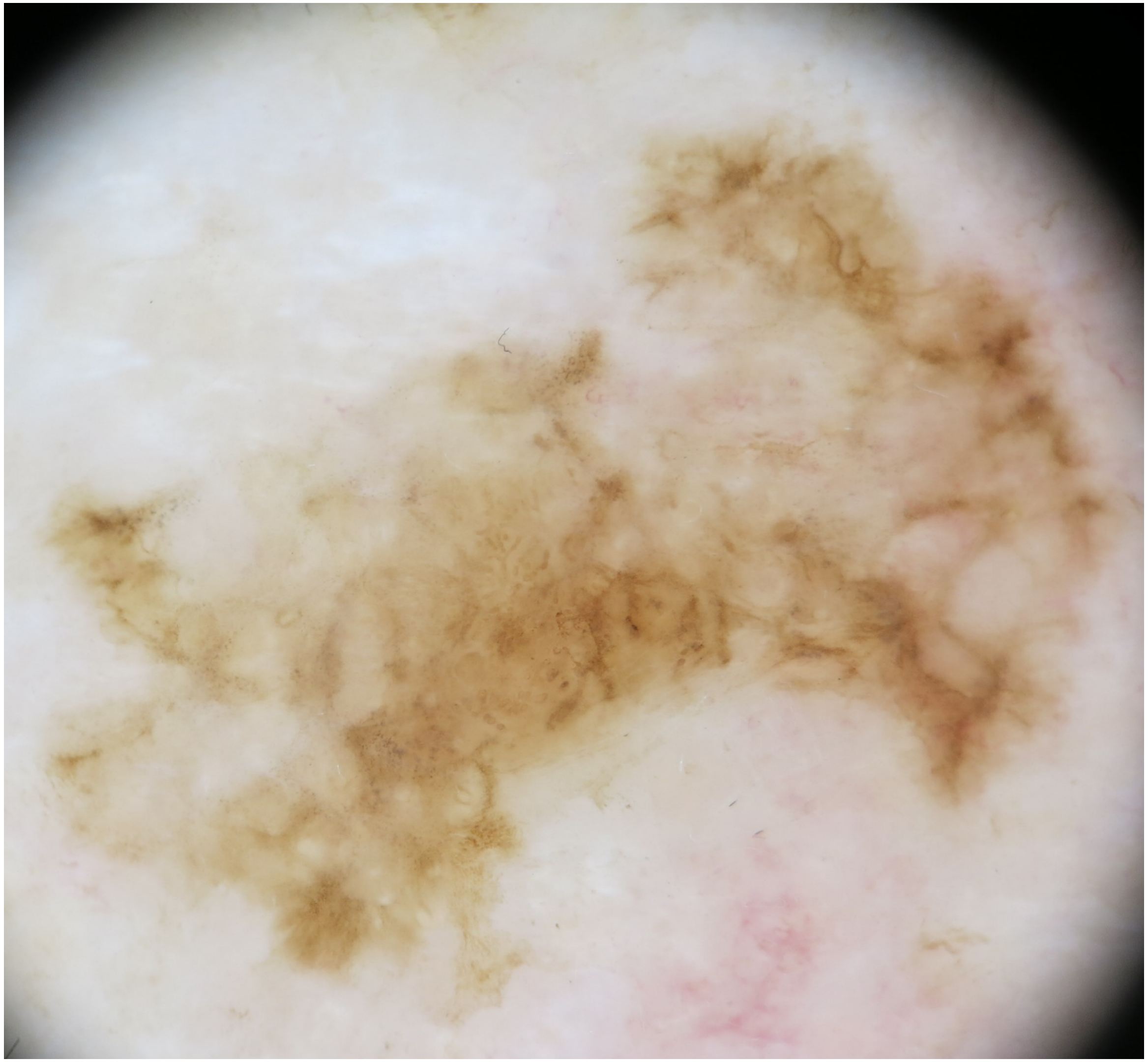

Location: Trunk

Invasive) Breslow interval: 0.1-0.5 mm

Case number 14

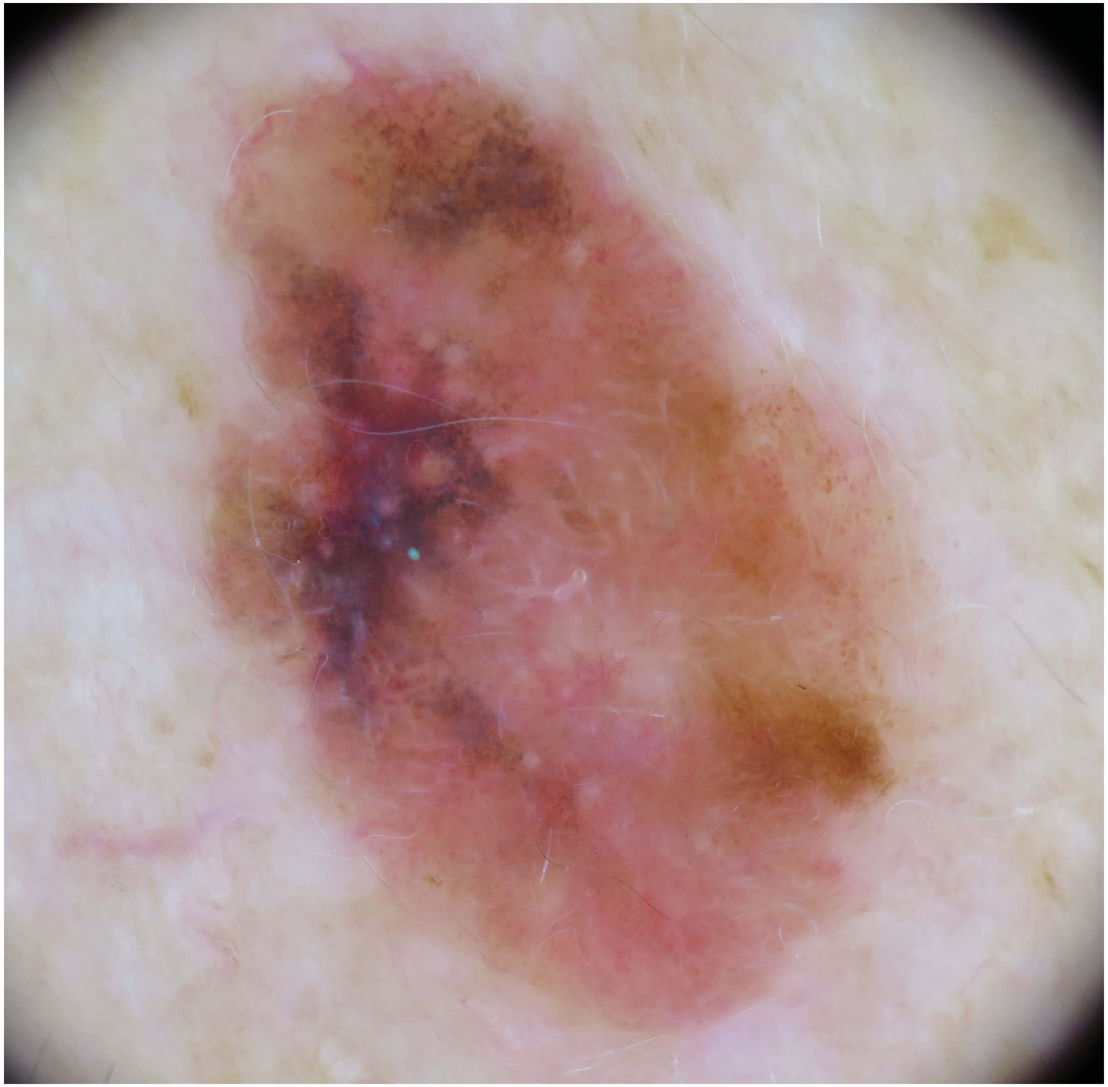

Location: Trunk

Invasive) Breslow interval: 0.9-1.0 mm

Case number 15

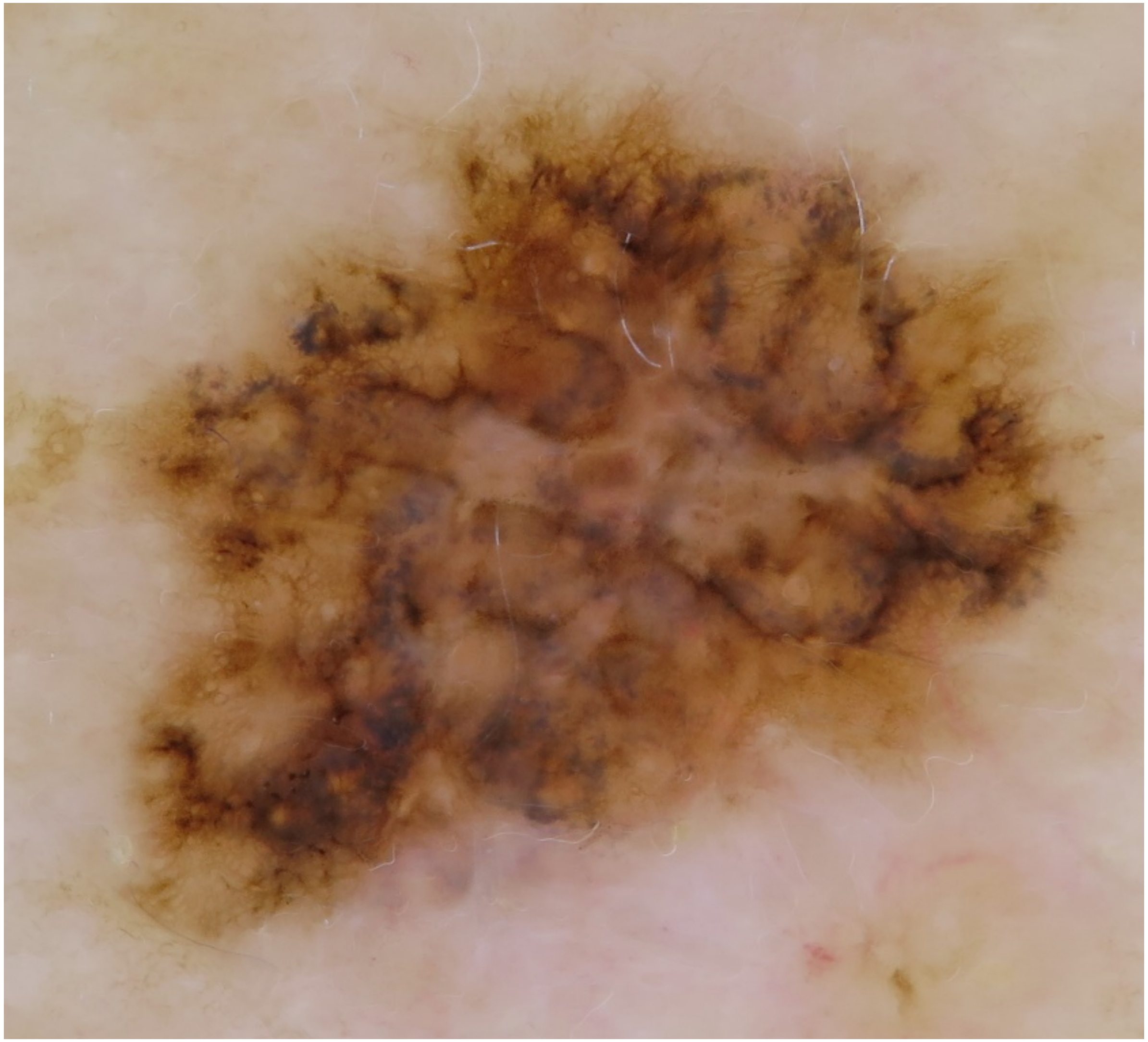

Location: Upper extremity

Invasive) Breslow interval: 0.1-0.5 mm

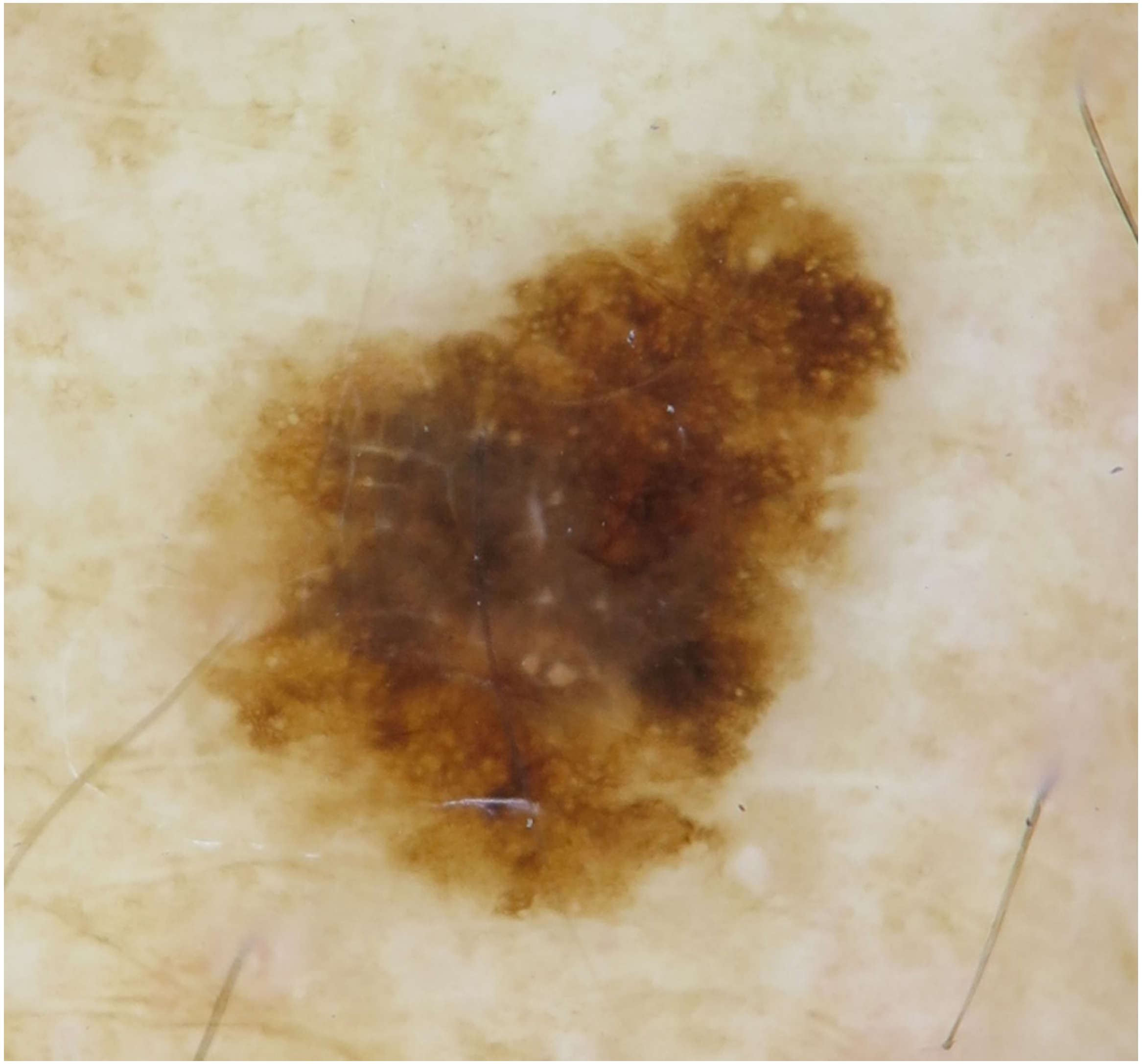

Location: Lower extremity

Invasive) Breslow interval: 0.1-0.5 mm

Case number 17

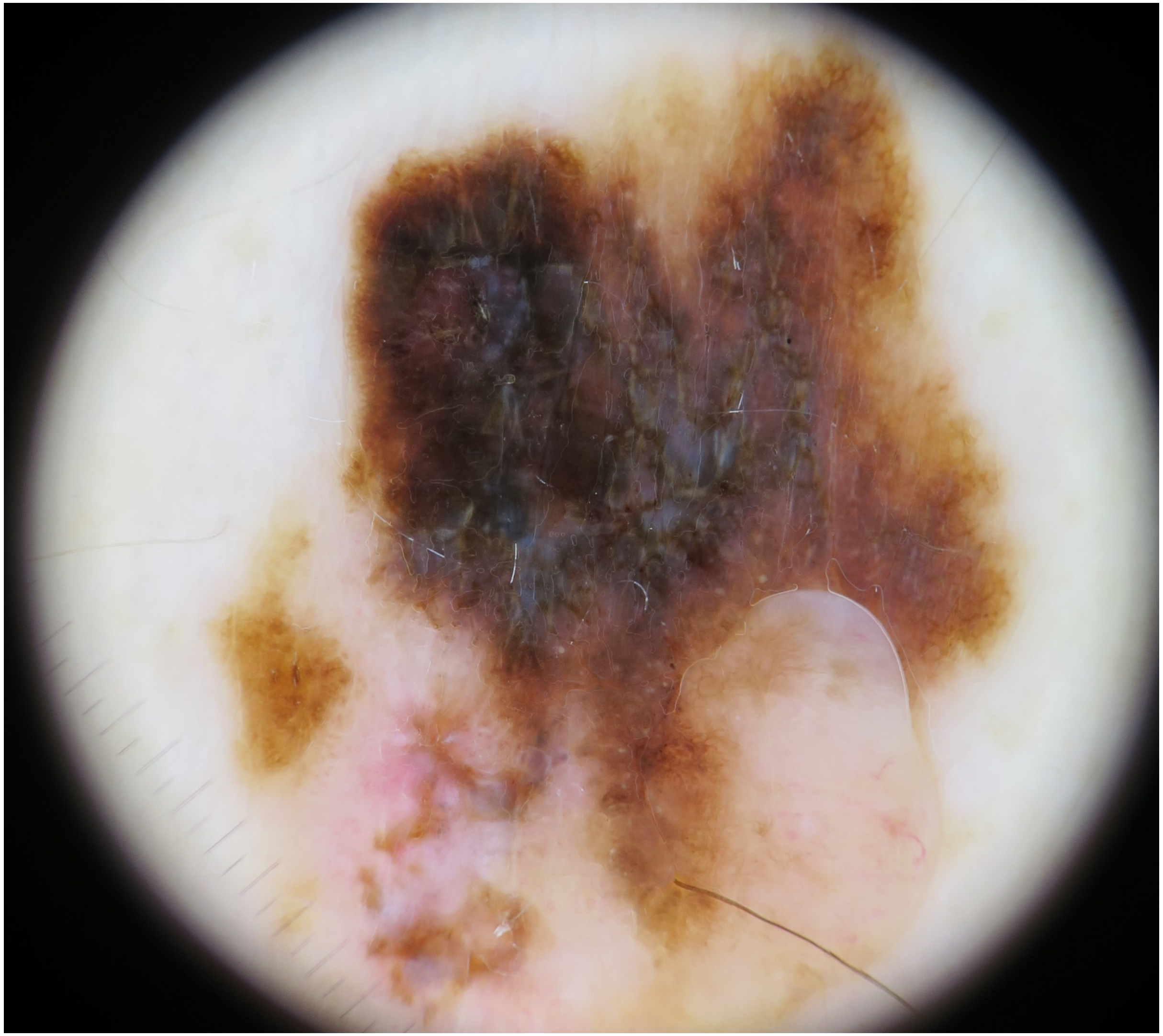

Location: Trunk

Invasive) Breslow interval: 1.1-2.0 mm

Case number 18

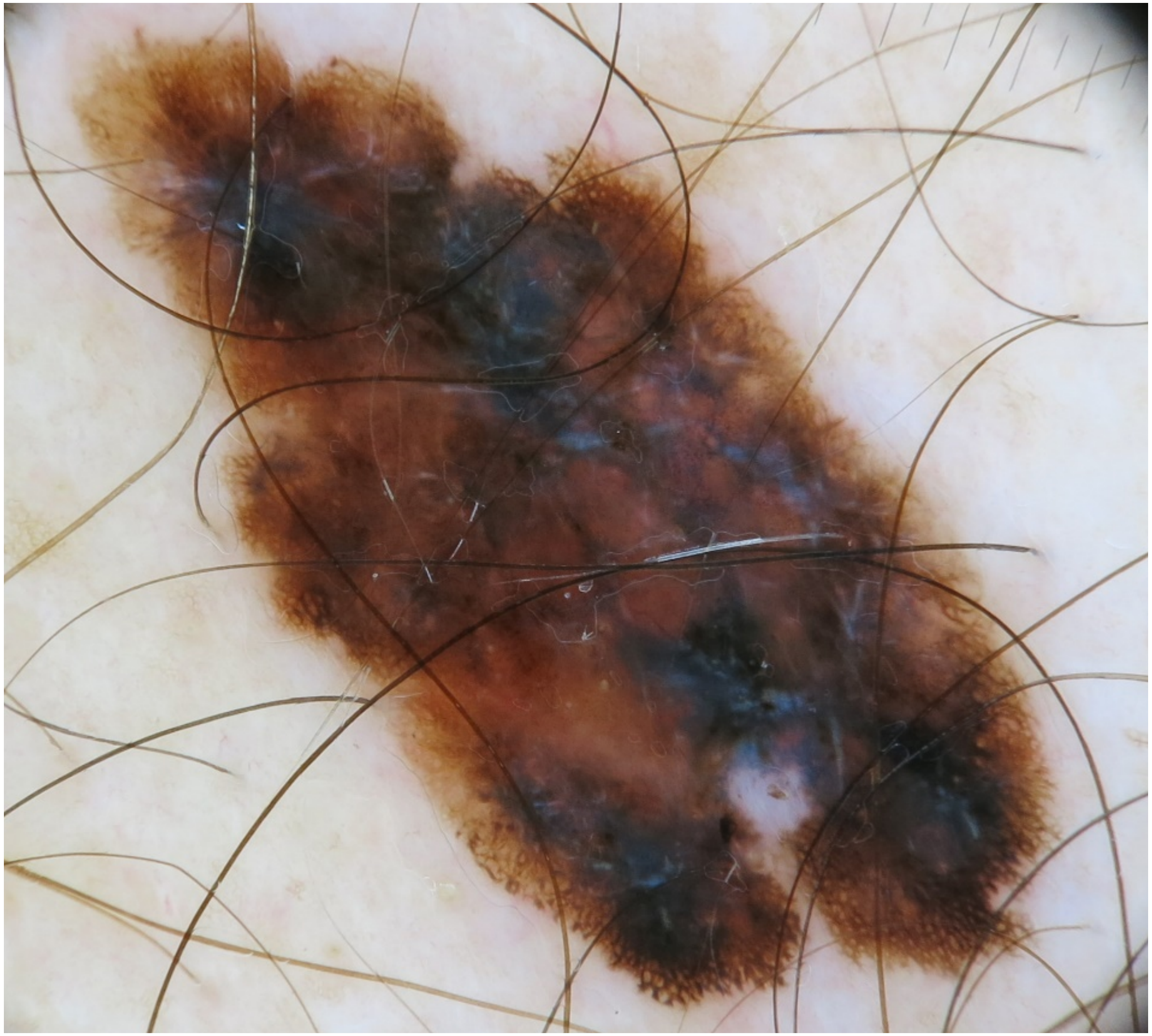

Location: Trunk

Invasive) Breslow interval: 0.6-0.8 mm

Case number 19

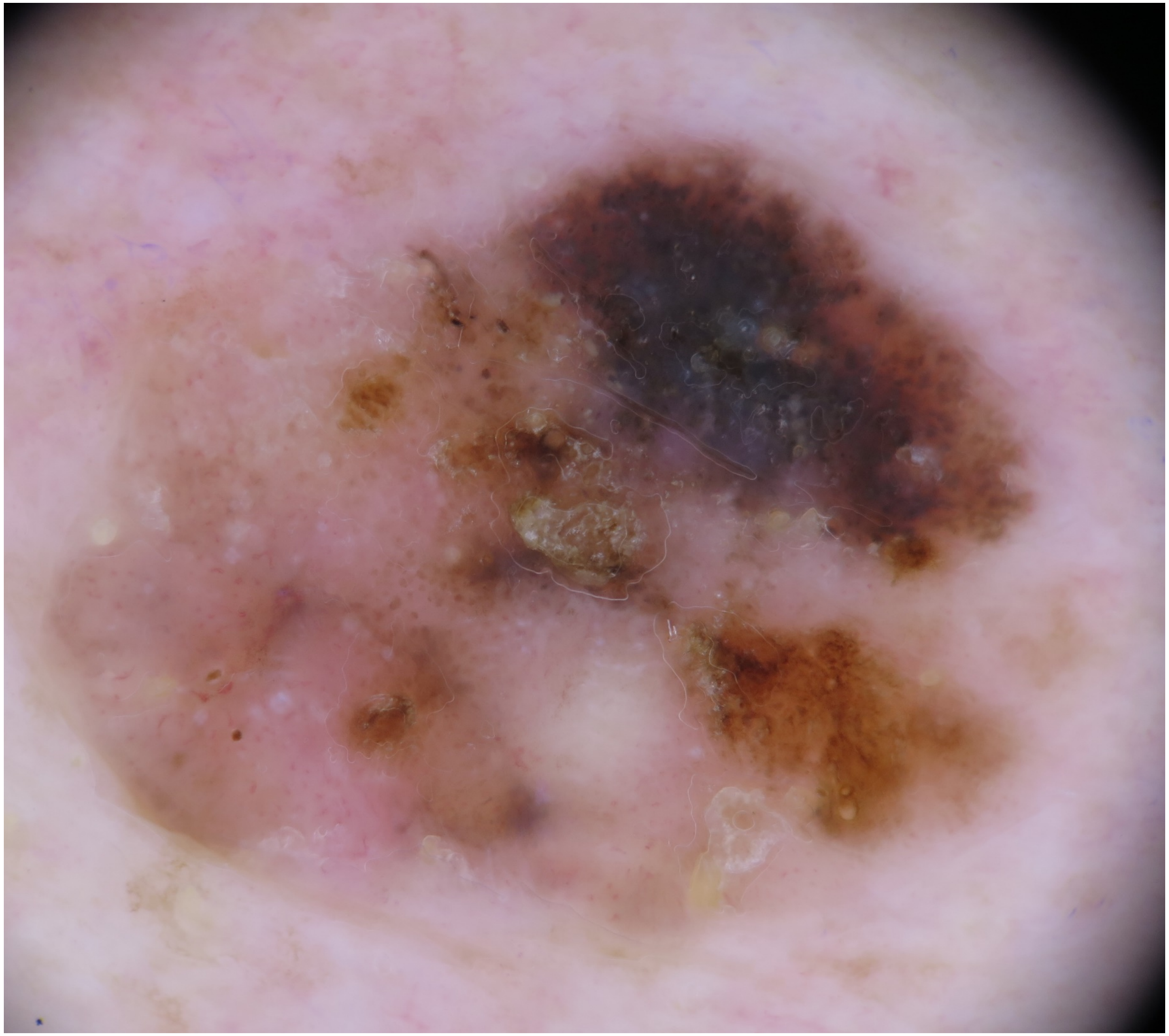

Location: Trunk

Invasive) Breslow interval: 1.1-2.0 mm

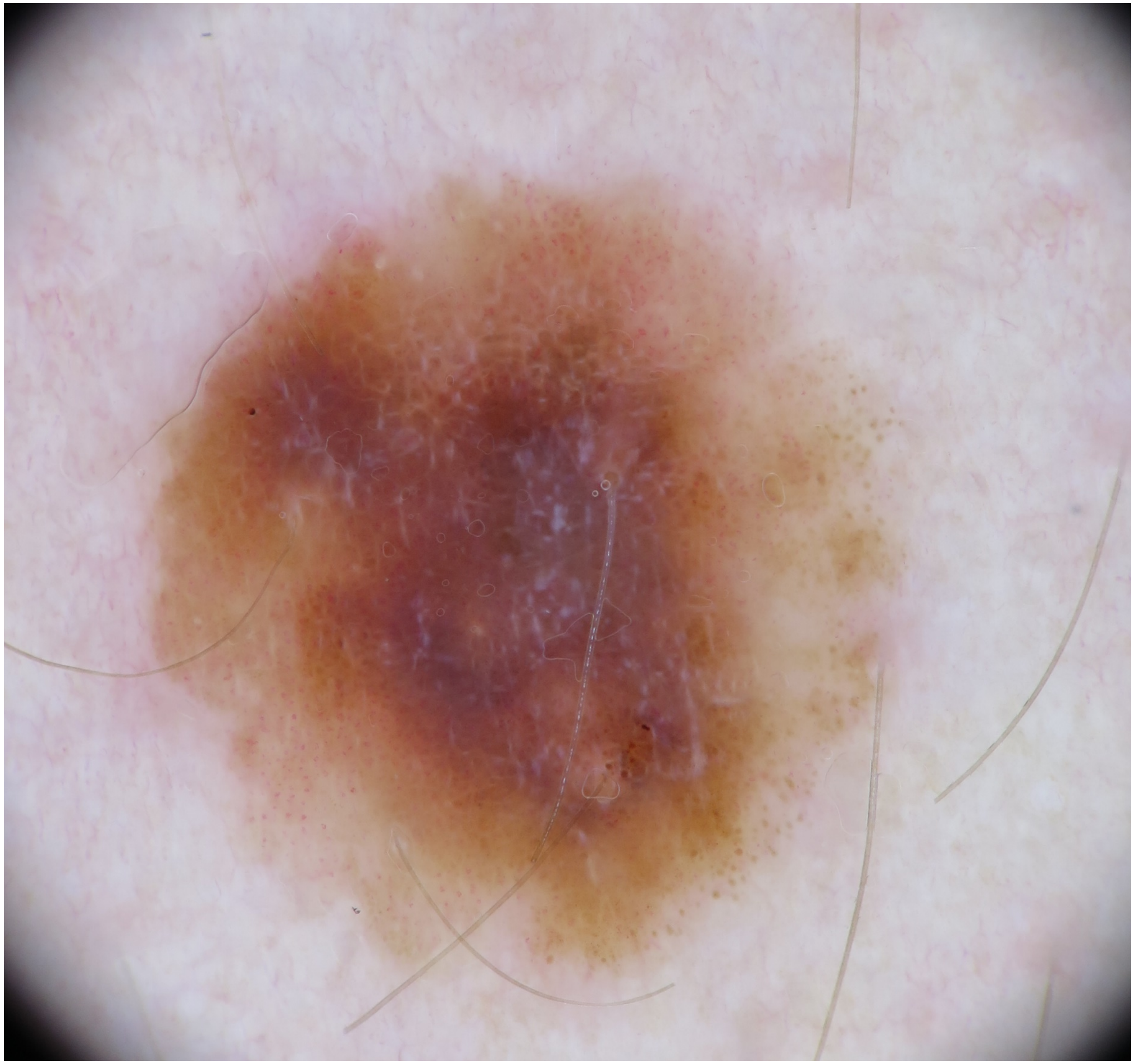

Location: Lower extremity

Invasive) Breslow interval: 0.6-0.8 mm

Case number 21

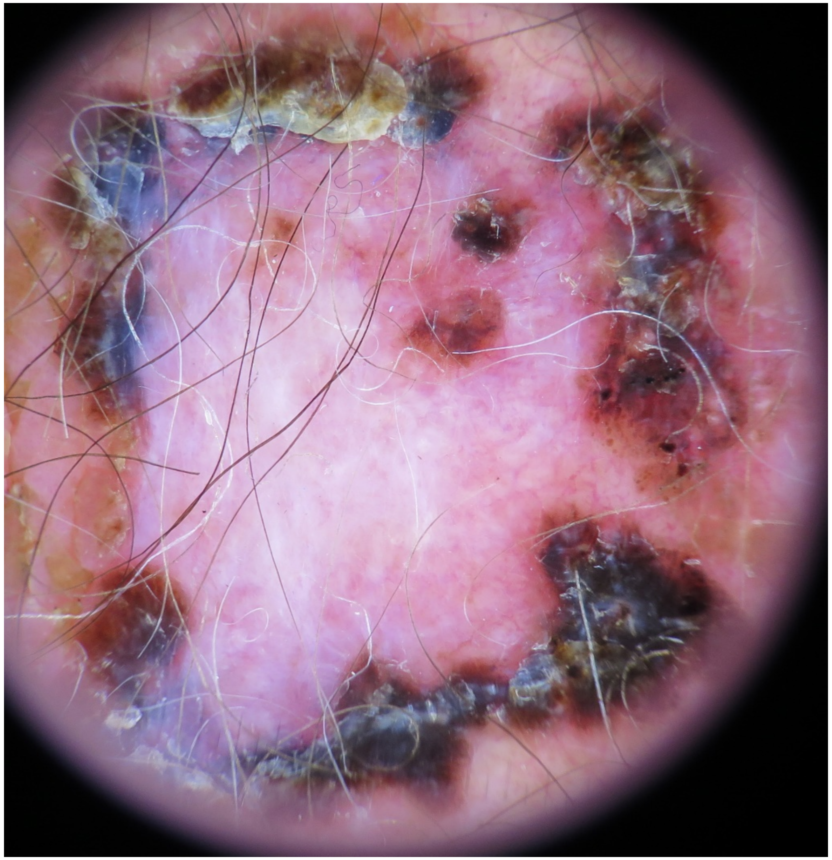

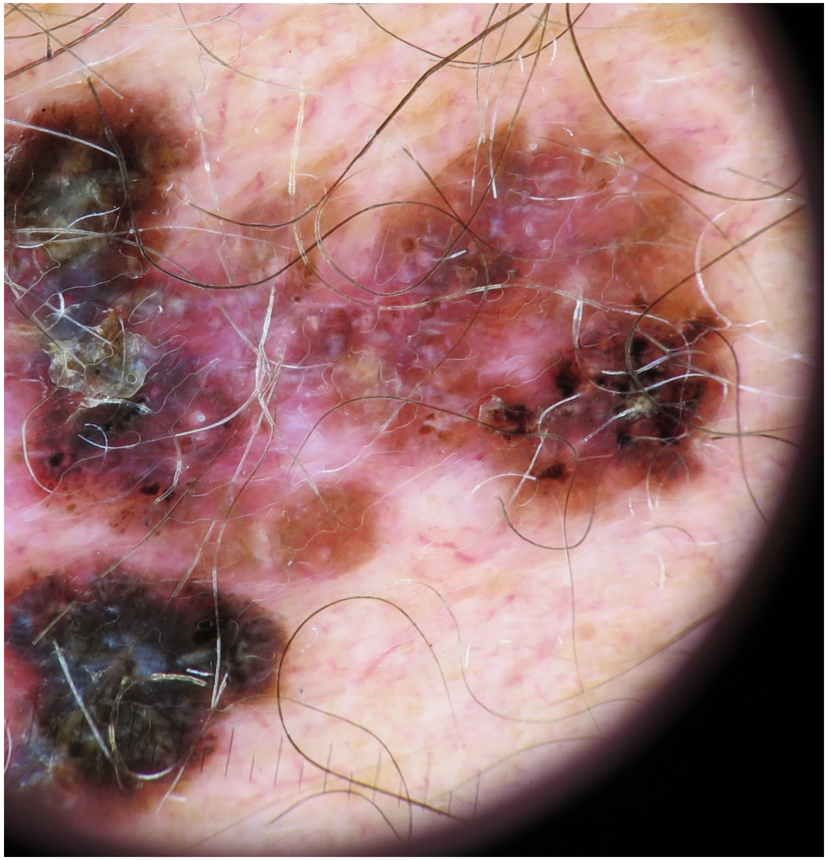

Location: Trunk

Invasive) Breslow interval: 1.1-2.0 mm

Case number 22

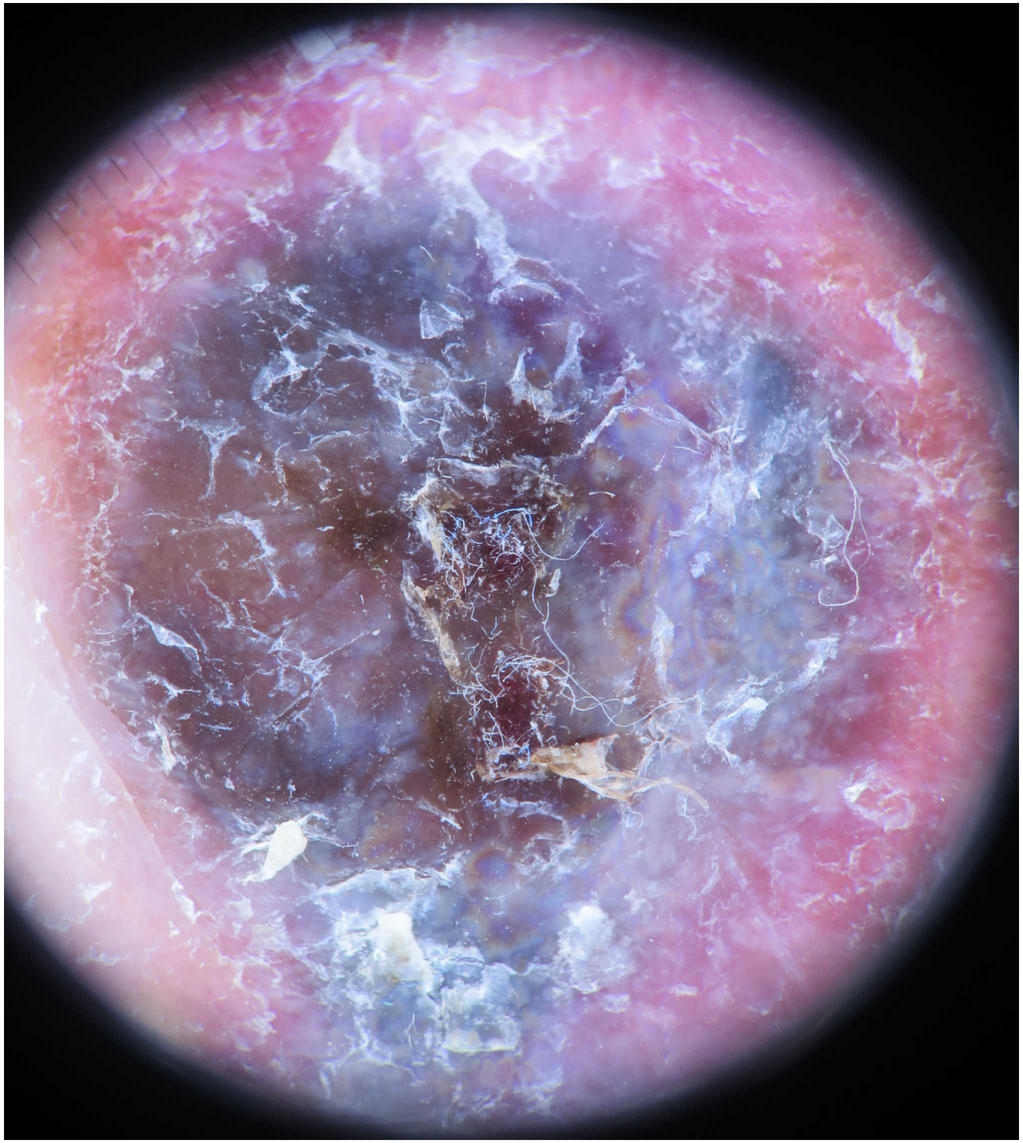

Location: Trunk

Invasive) Breslow interval: > 4mm

Case number 23

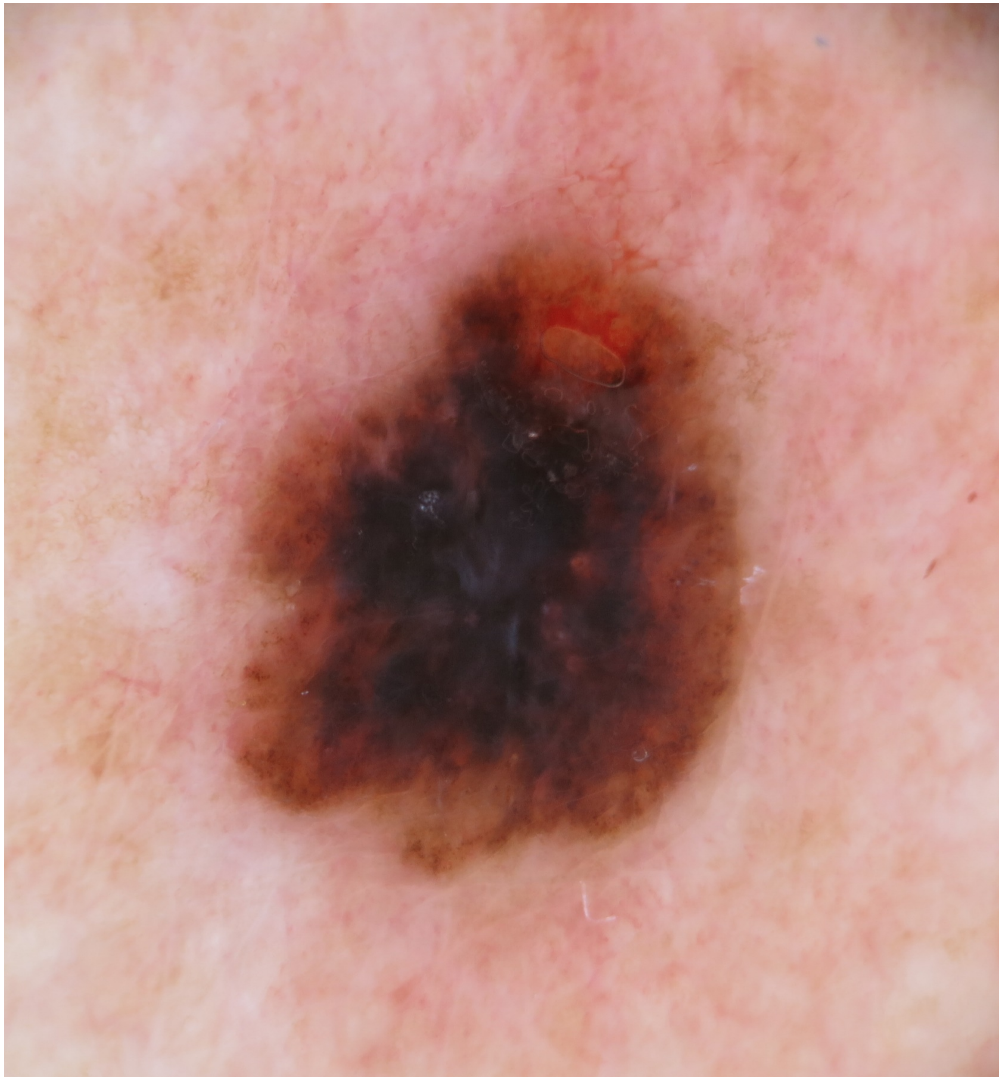

Location: Trunk

Invasive) Breslow interval: 0.6-0.8 mm

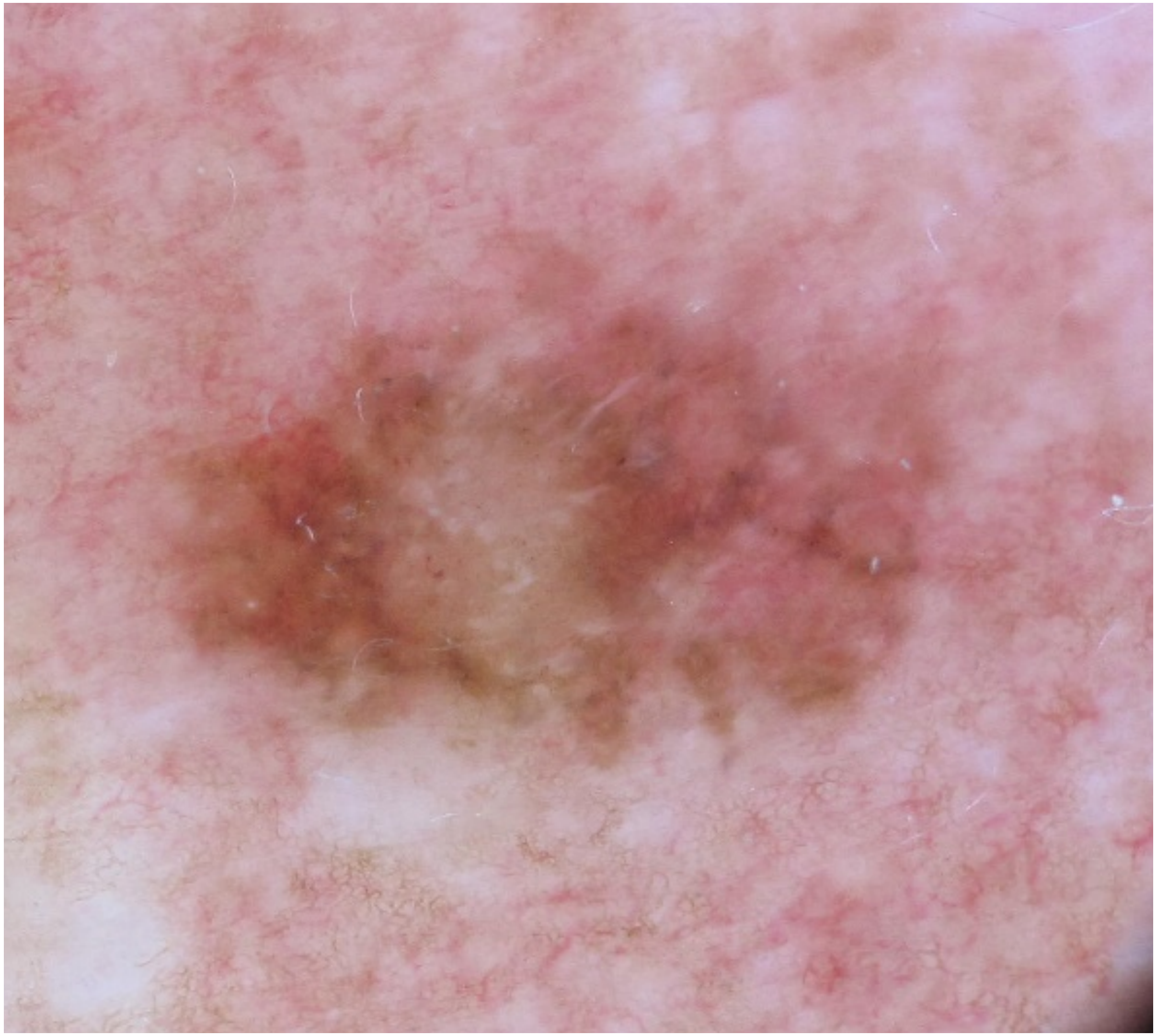

Location: Trunk

Invasive) Breslow interval: 0.1-0.5 mm

Case number 25

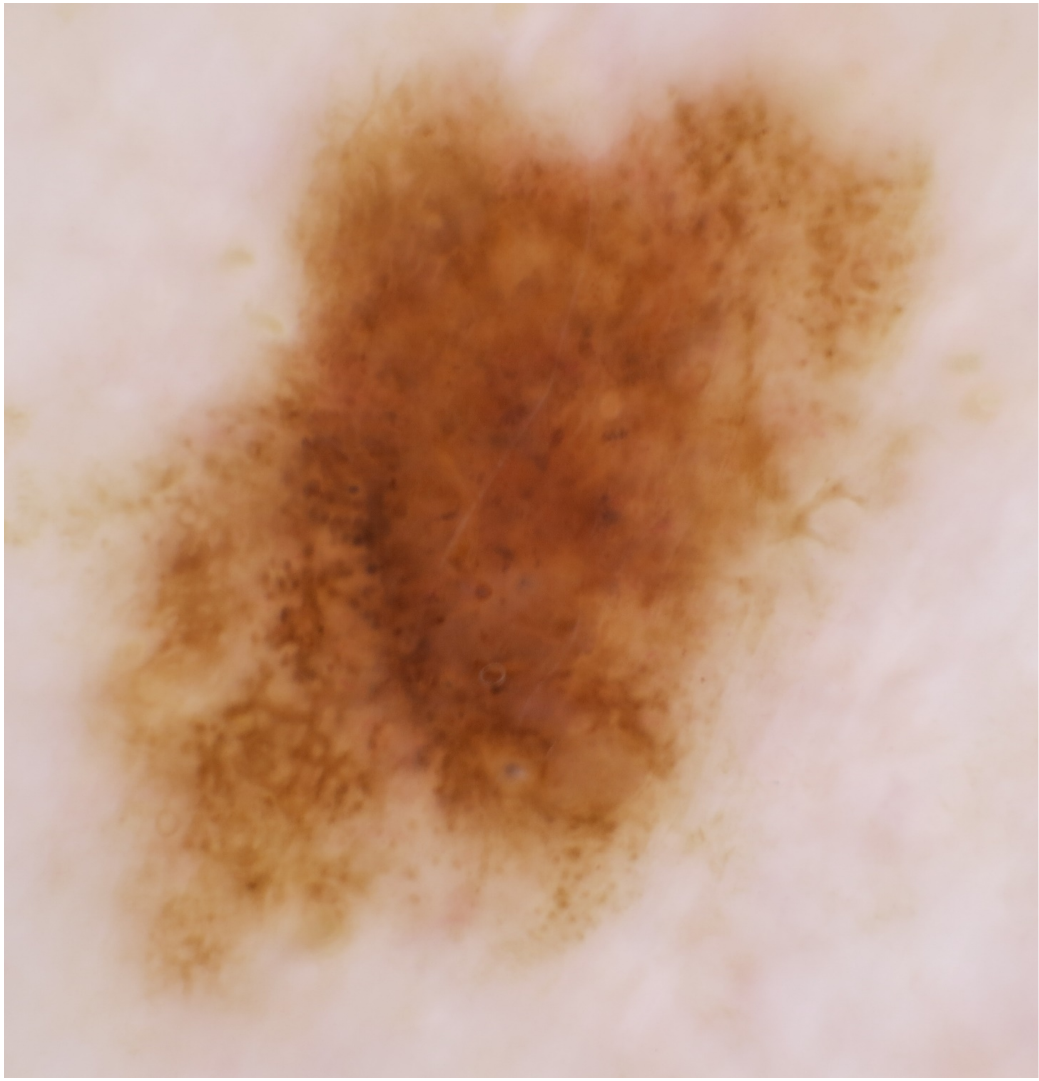

Location: Trunk

In situ melanoma

Case number 26

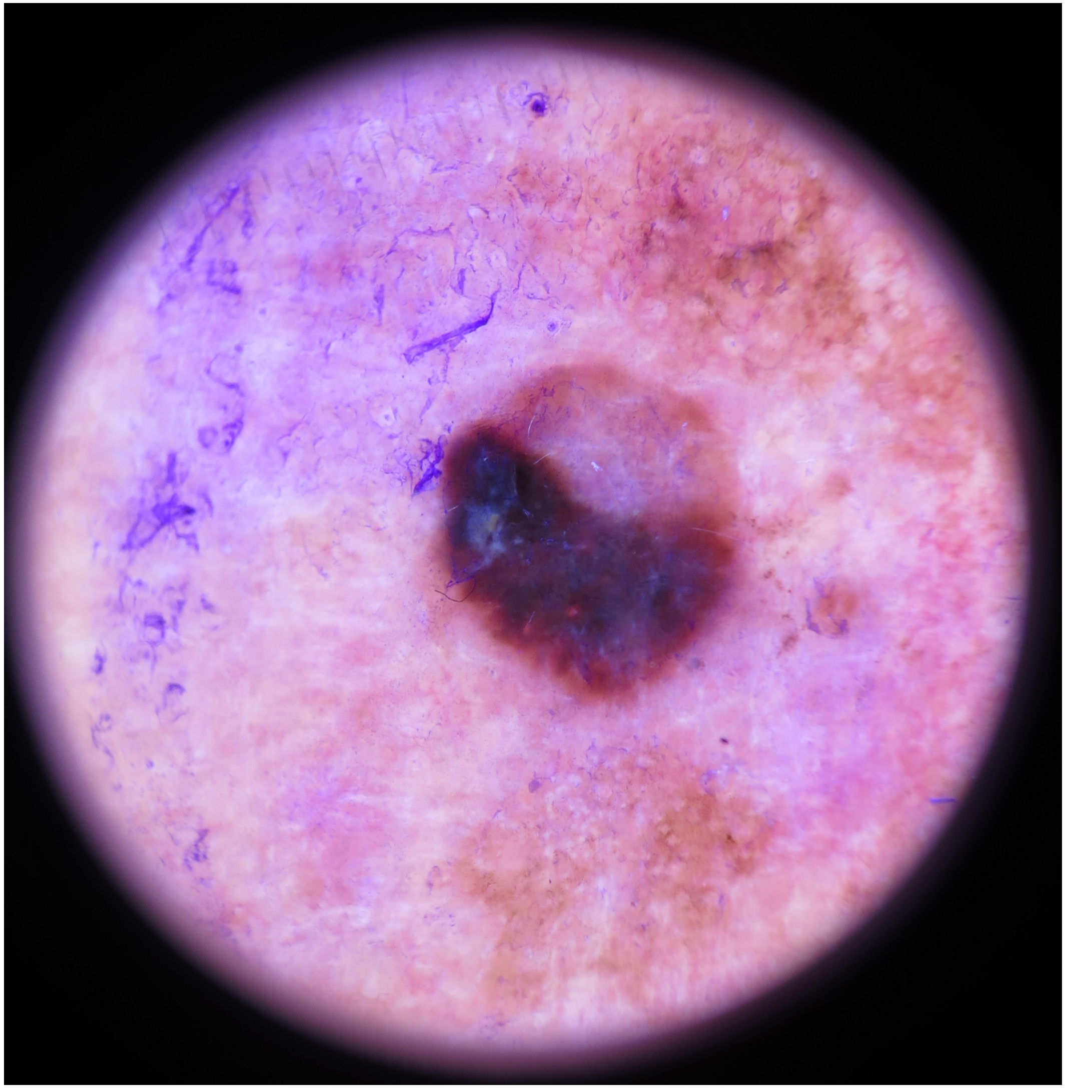

Location: Scalp

Invasive) Breslow interval: 0.9-1.0 mm

Case number 27

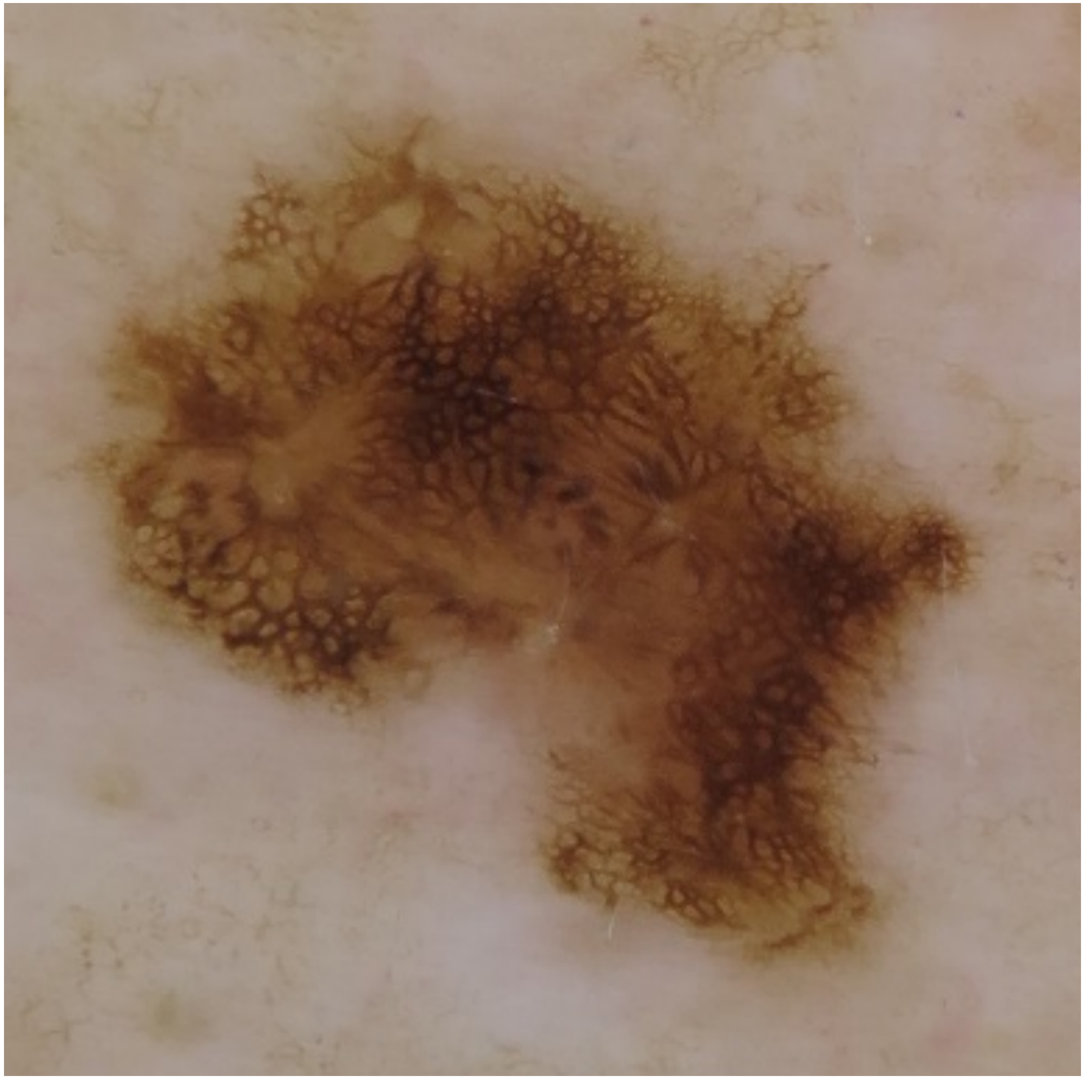

Location: Trunk

In situ melanoma

Case number 28

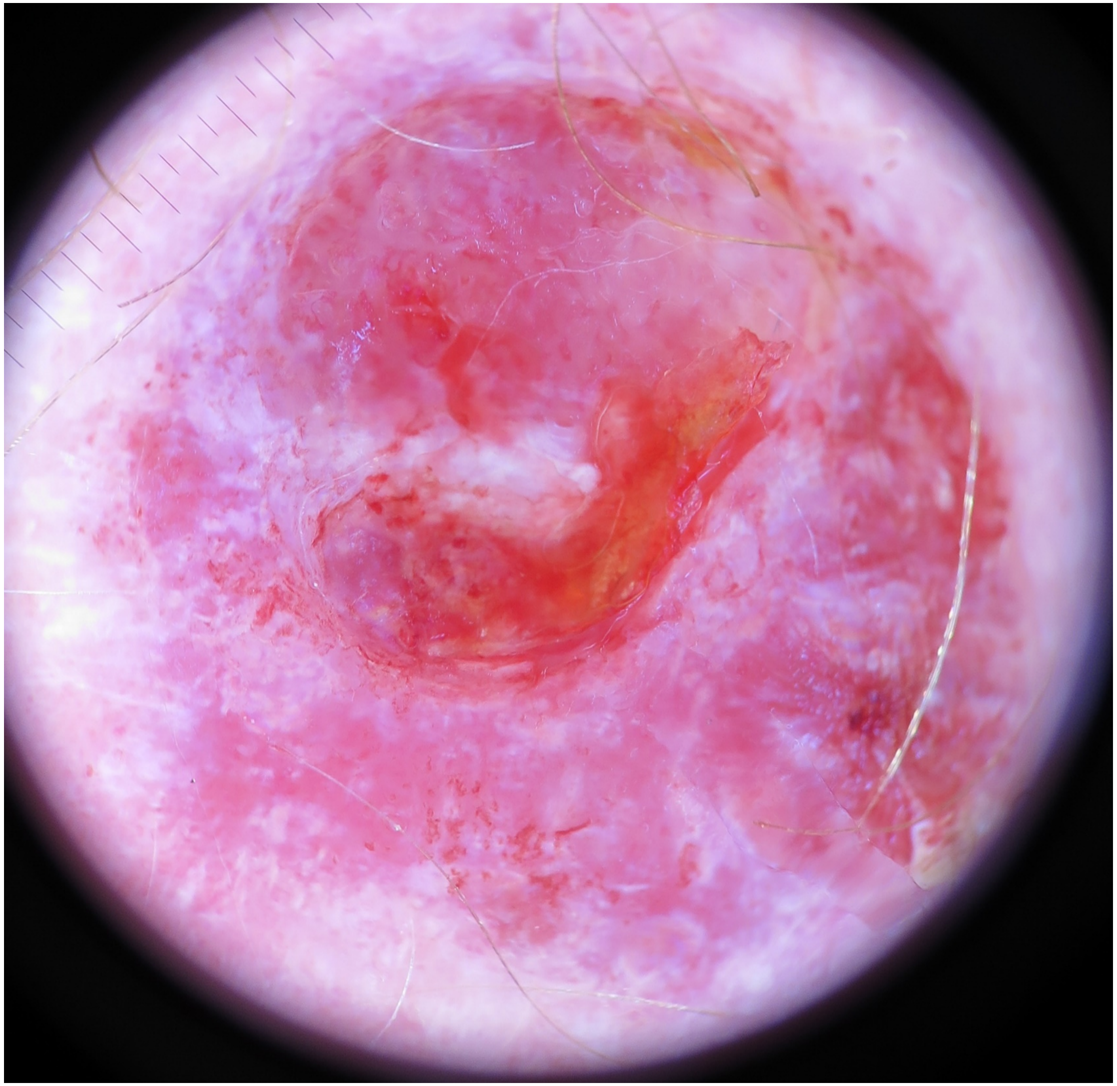

Location: Lower extremity

Invasive) Breslow interval: 2.1-4.0 mm

Case number 29

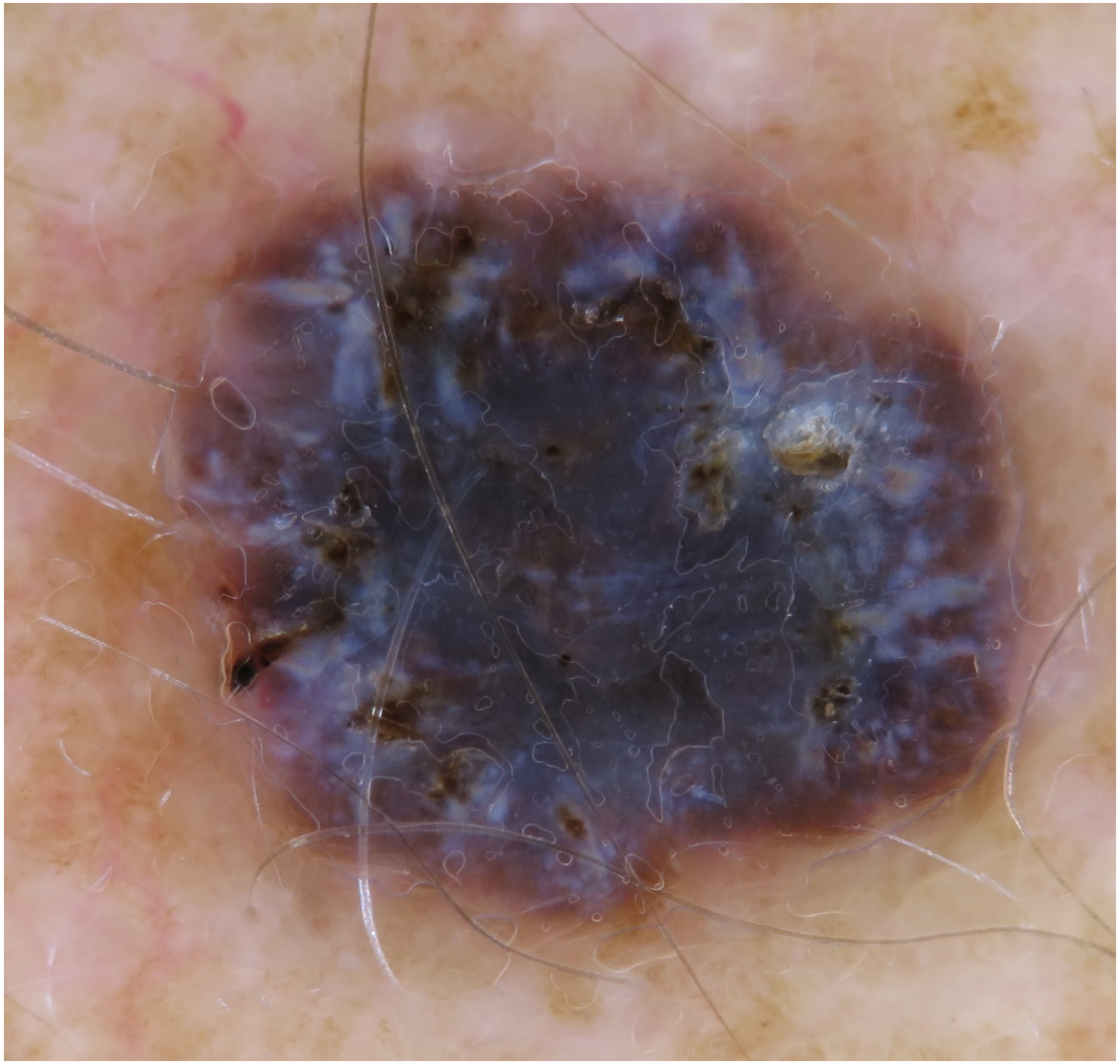

Location: Upper extremity

Invasive) Breslow interval: 1.1-2.0 mm

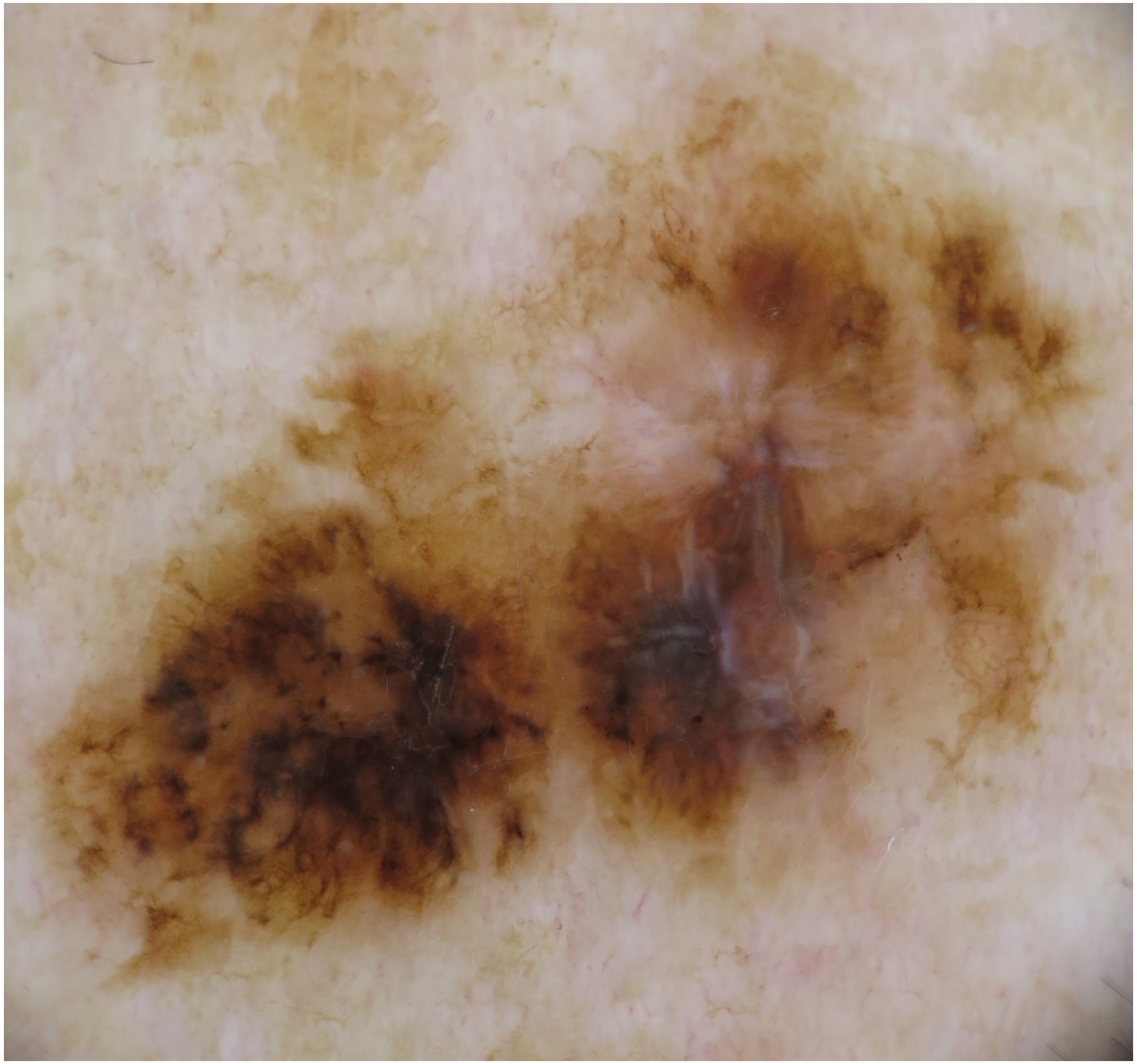

Location: Lower extremity

In situ melanoma

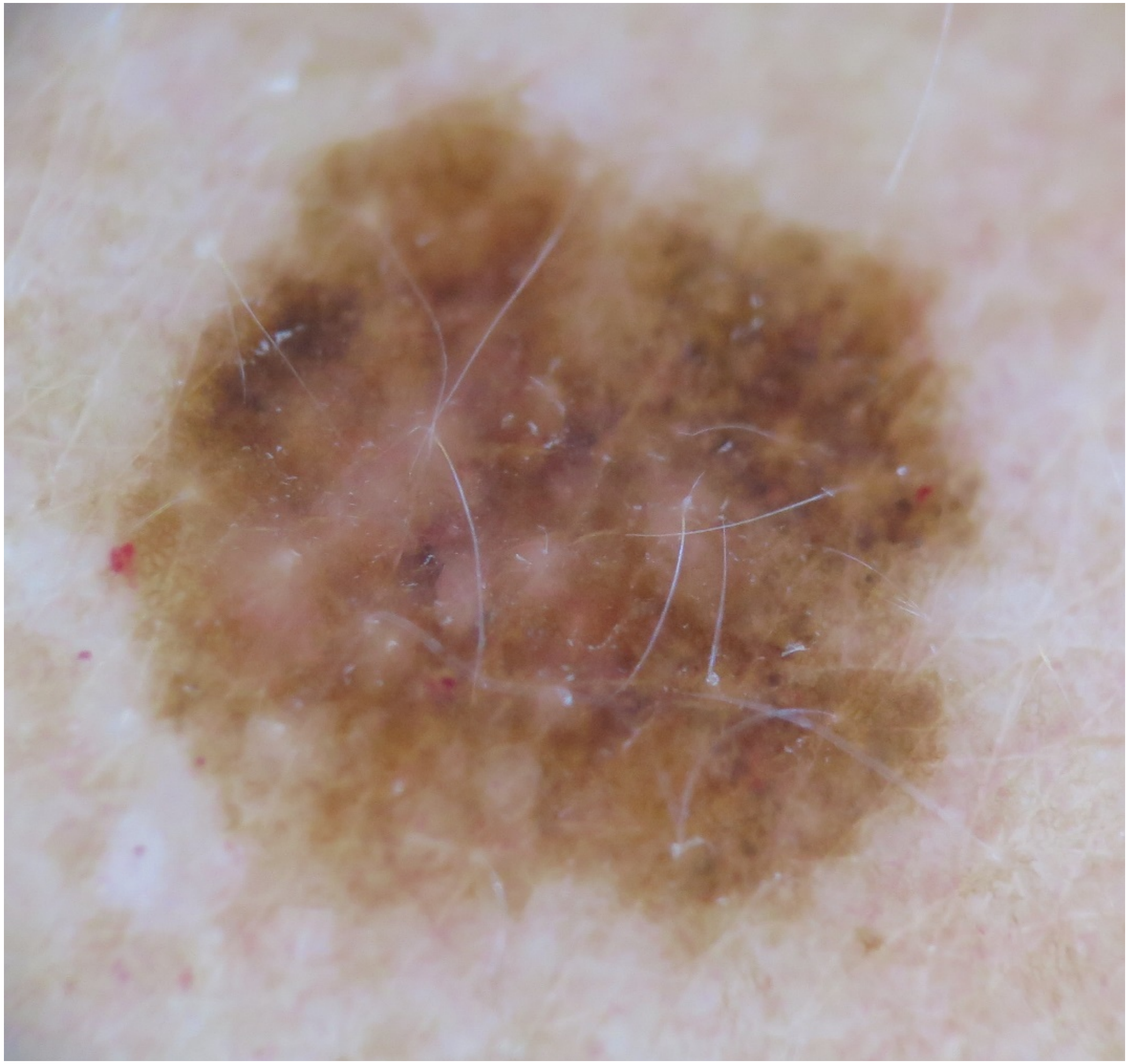

Location: Trunk

In situ melanoma

Case number 32

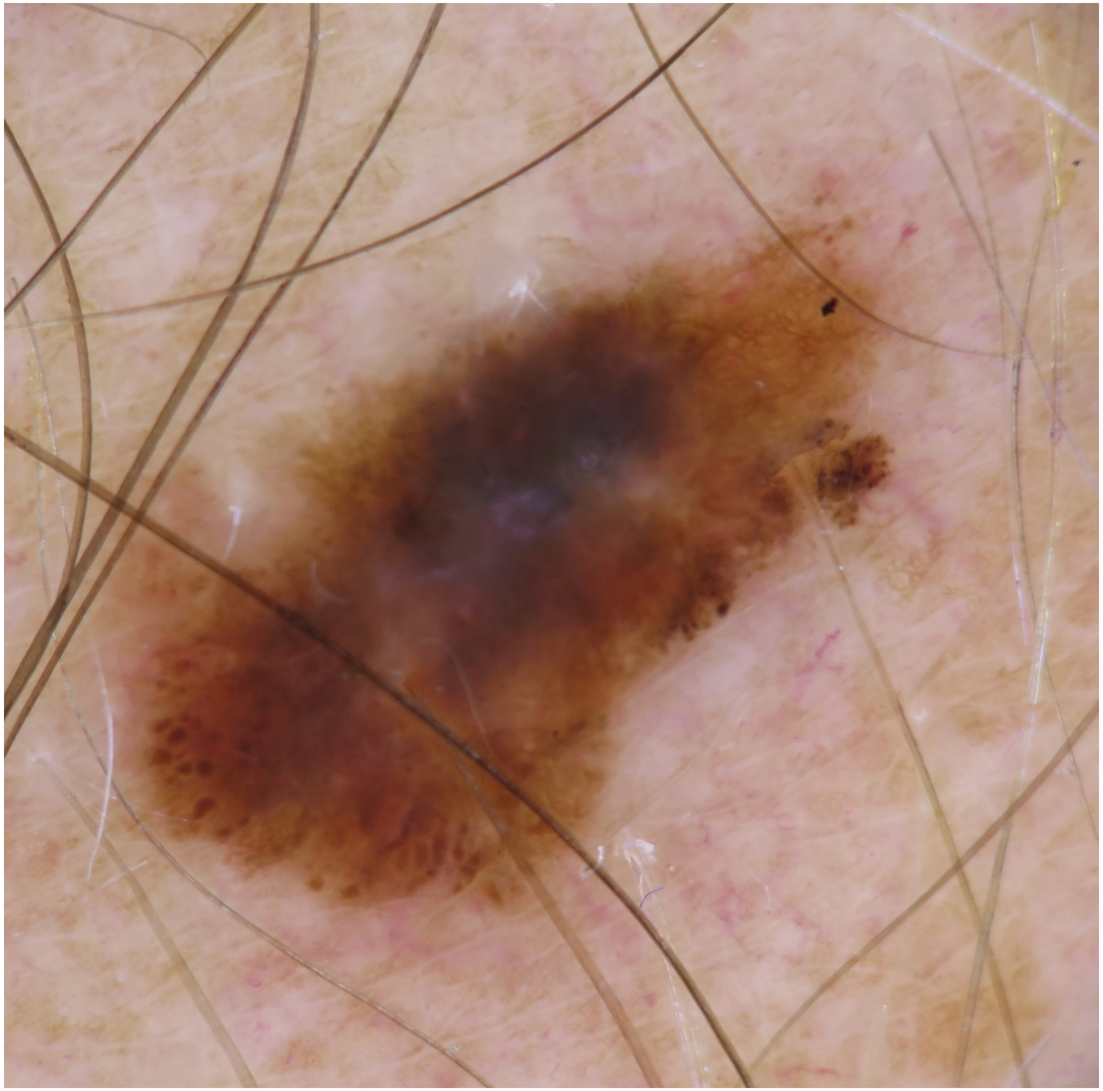

Location: Trunk

In situ melanoma

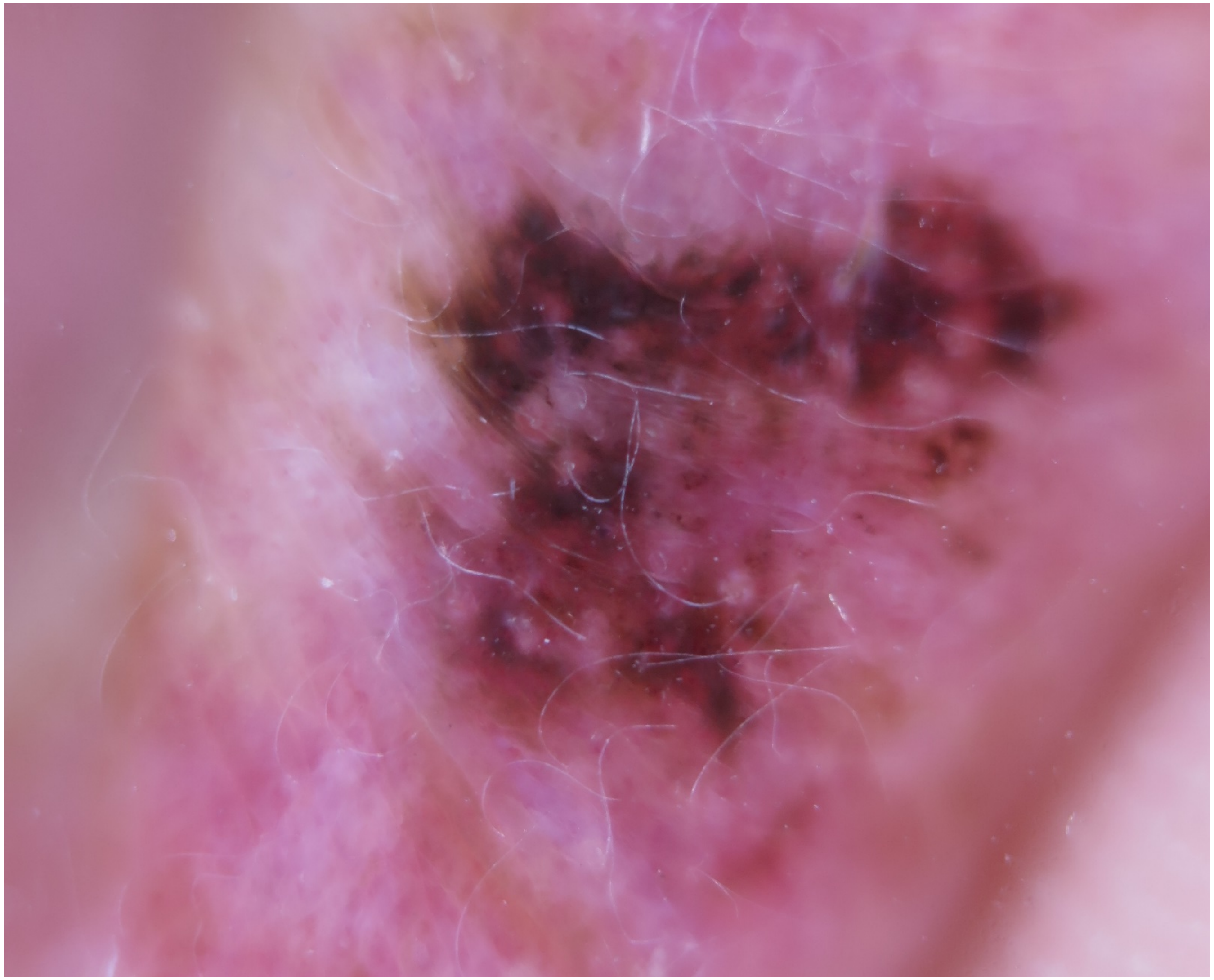

Location: Ear

In situ melanoma

Case number 34

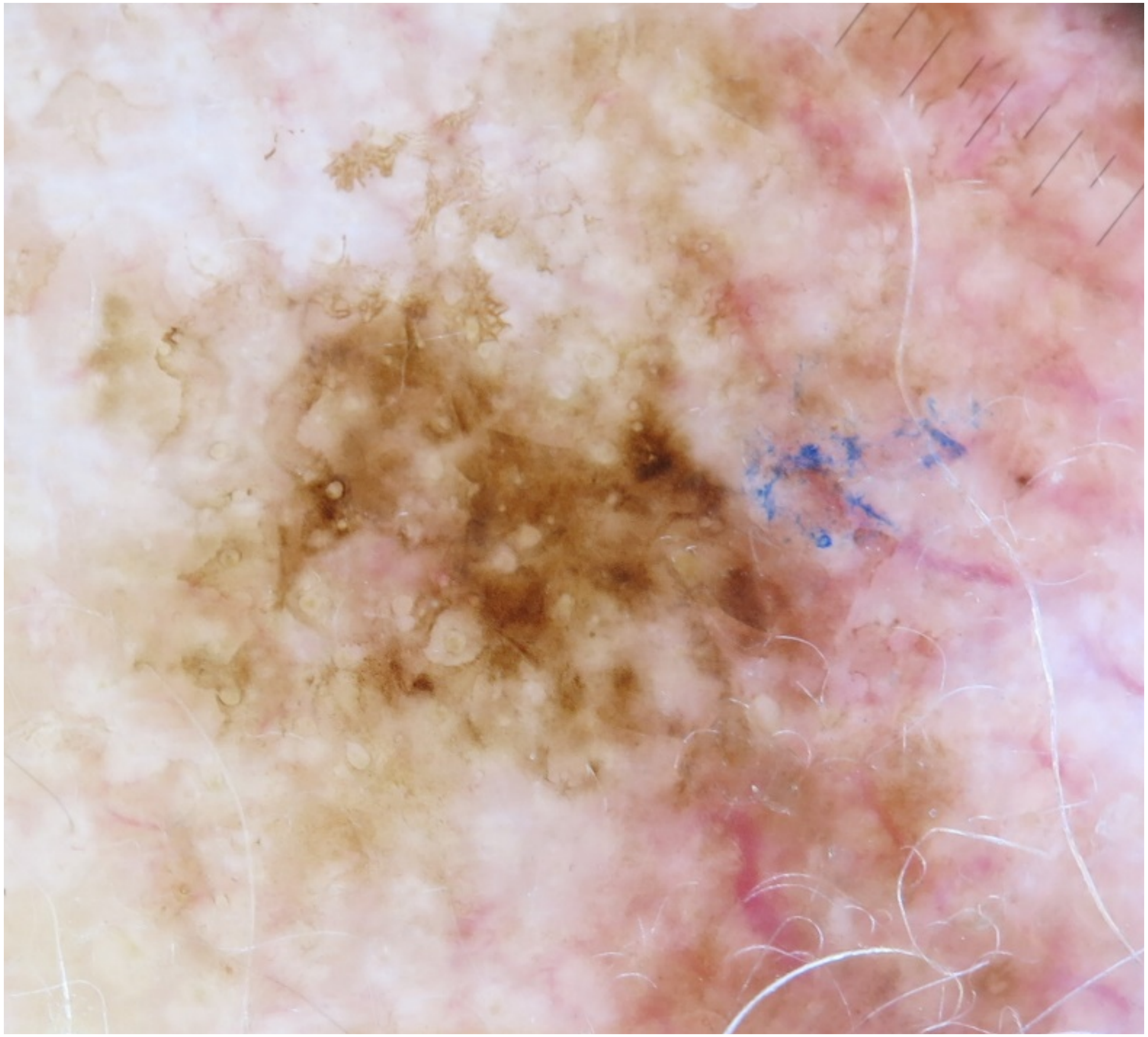

Location: Scalp

Invasive) Breslow interval: 0.1-0.5 mm

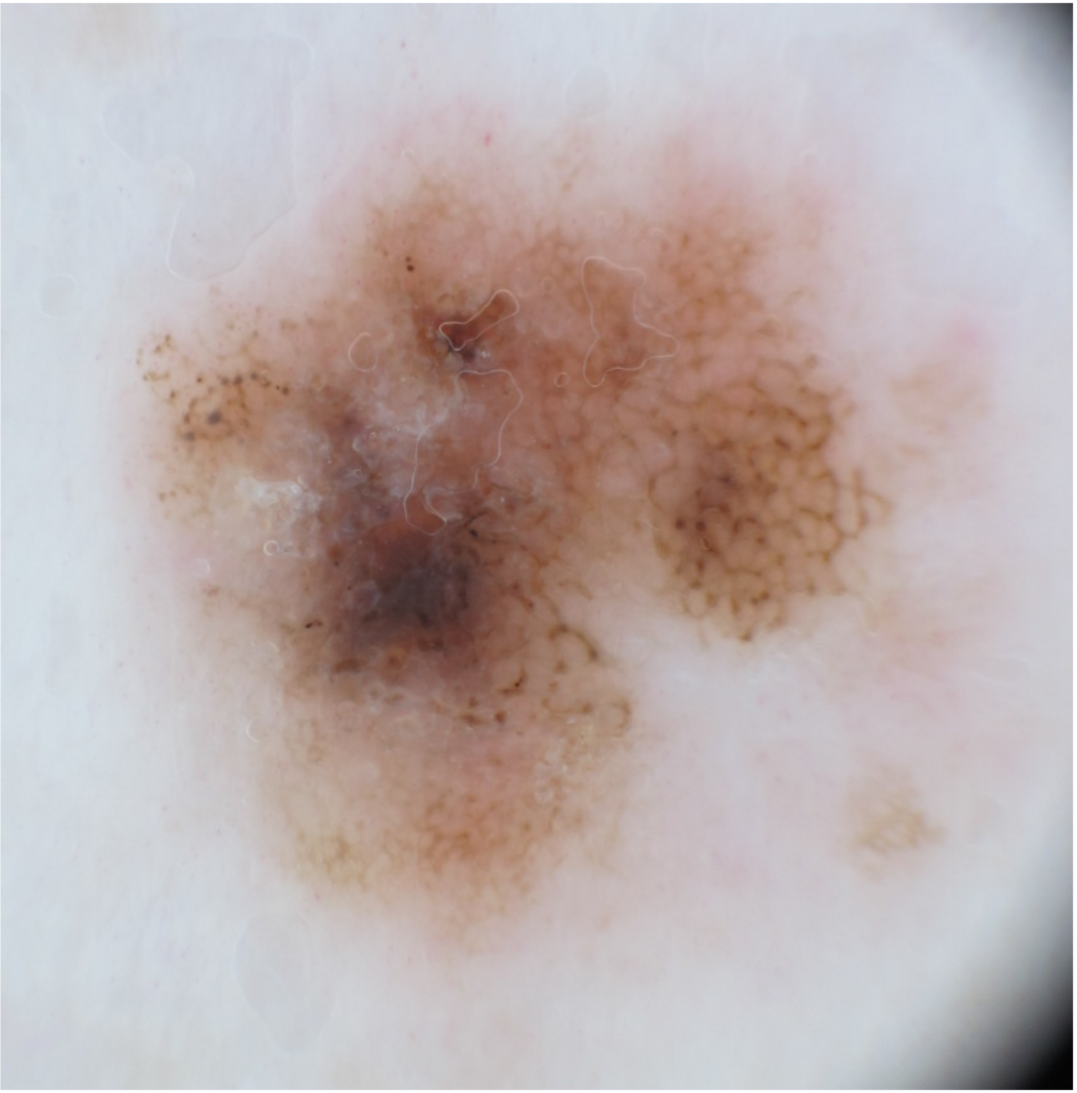

Location: Lower extremity

Invasive) Breslow interval: 0.6-0.8 mm

Case number 36

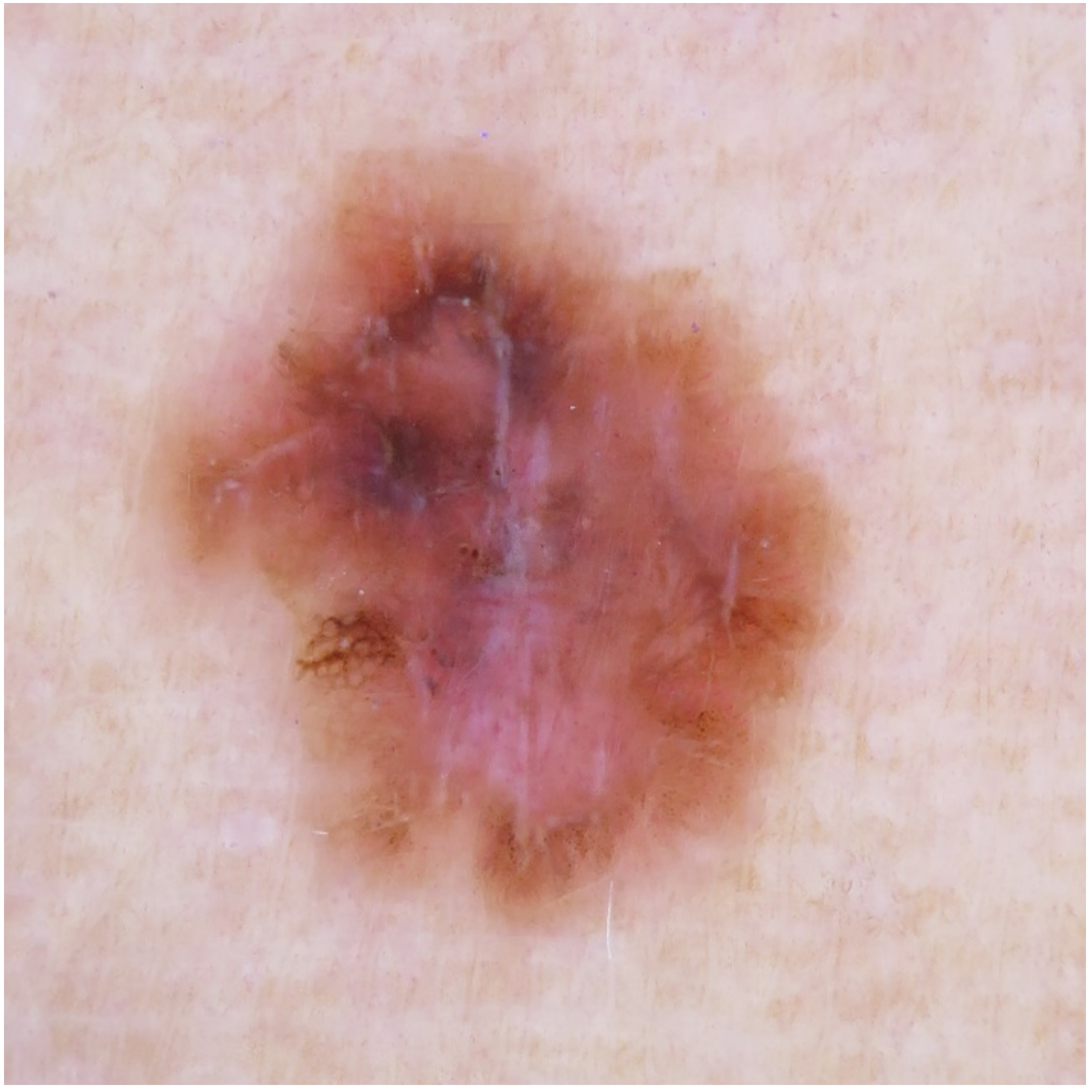

Location: Lower extremity

Invasive) Breslow interval: 0.6-0.8 mm

Case number 37

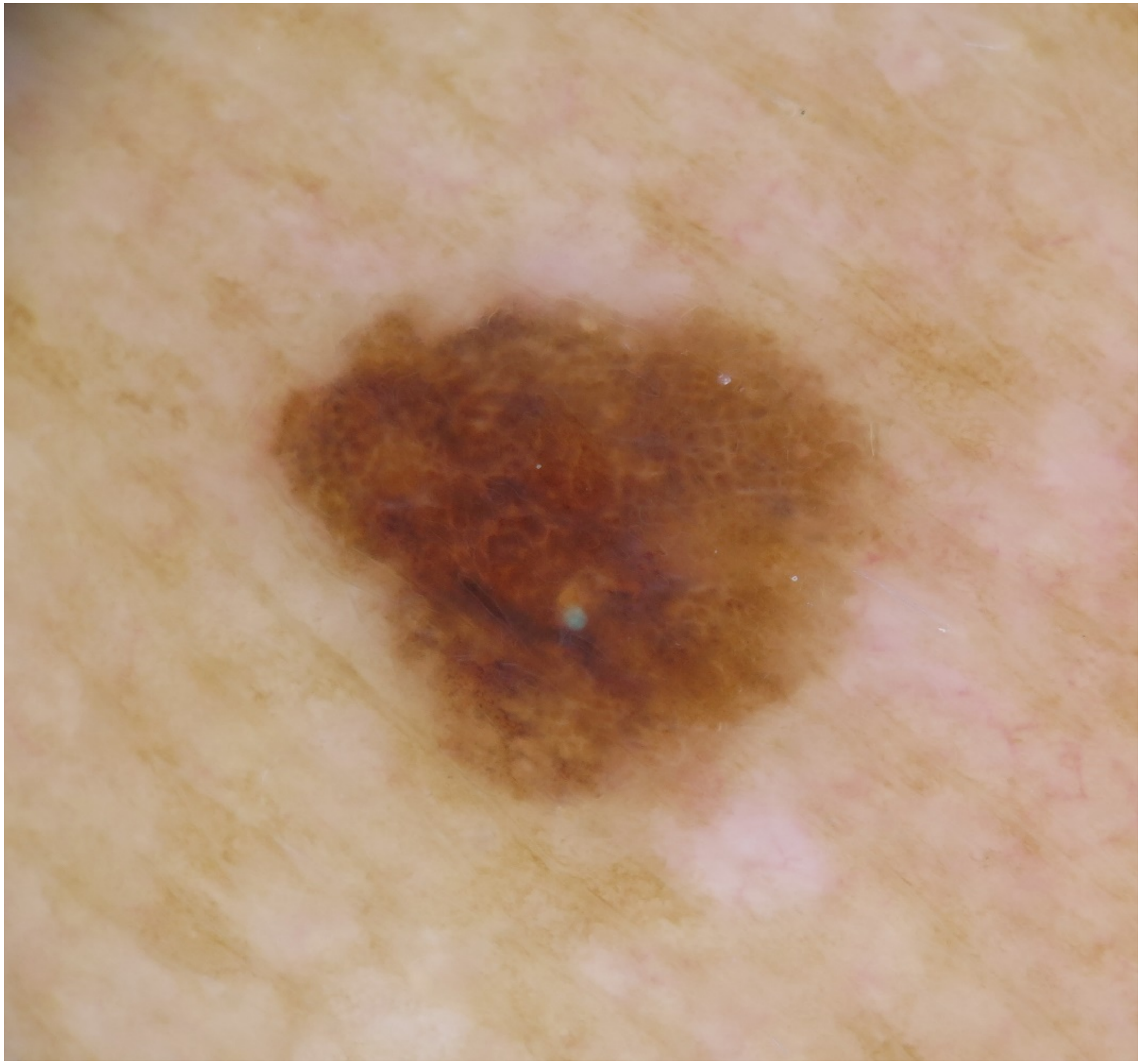

Location: Trunk

Invasive) Breslow interval: 0.1-0.5 mm

Case number 38

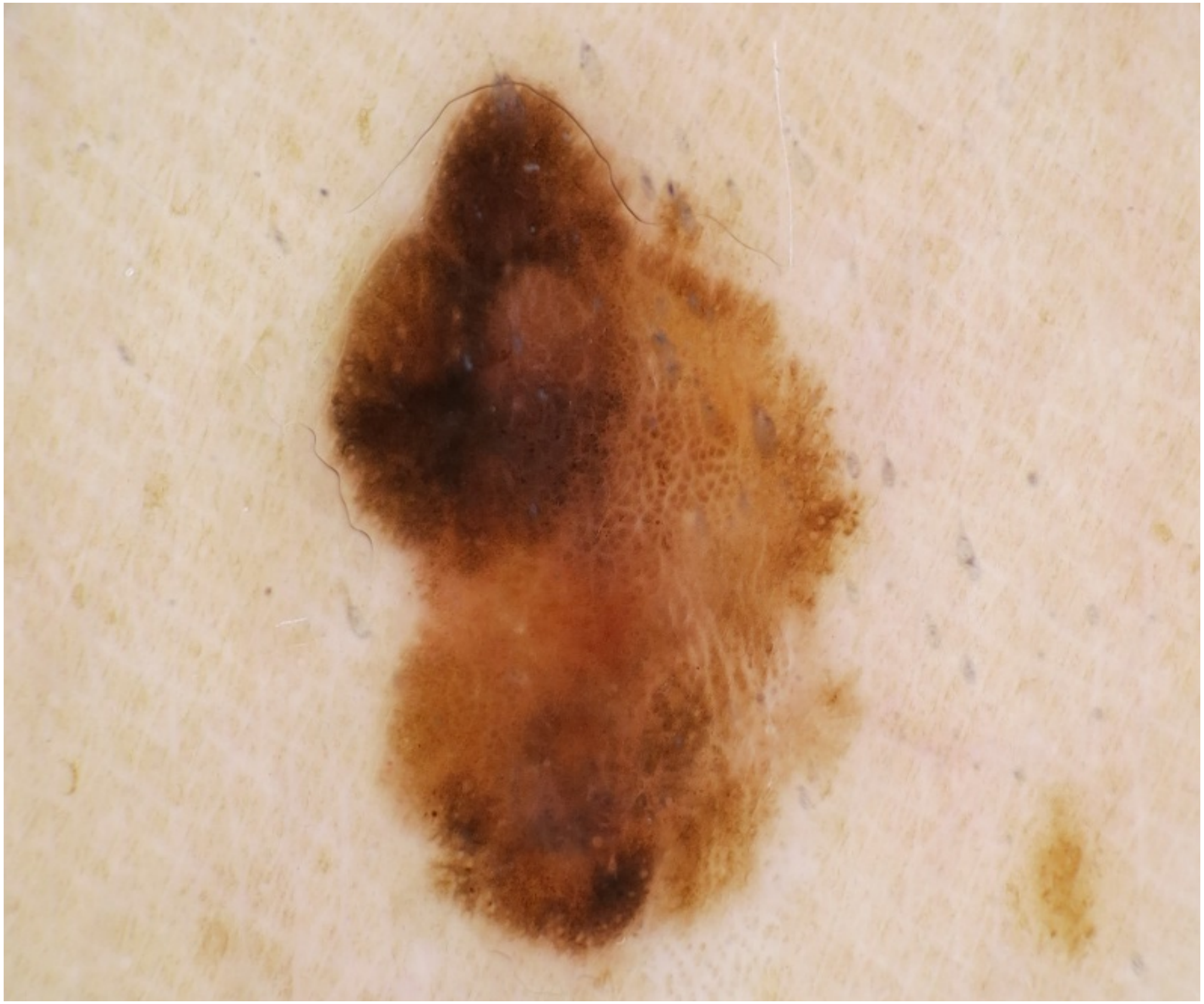

Location: Lower extremity

Invasive) Breslow interval: 0.6-0.8 mm

Case number 39

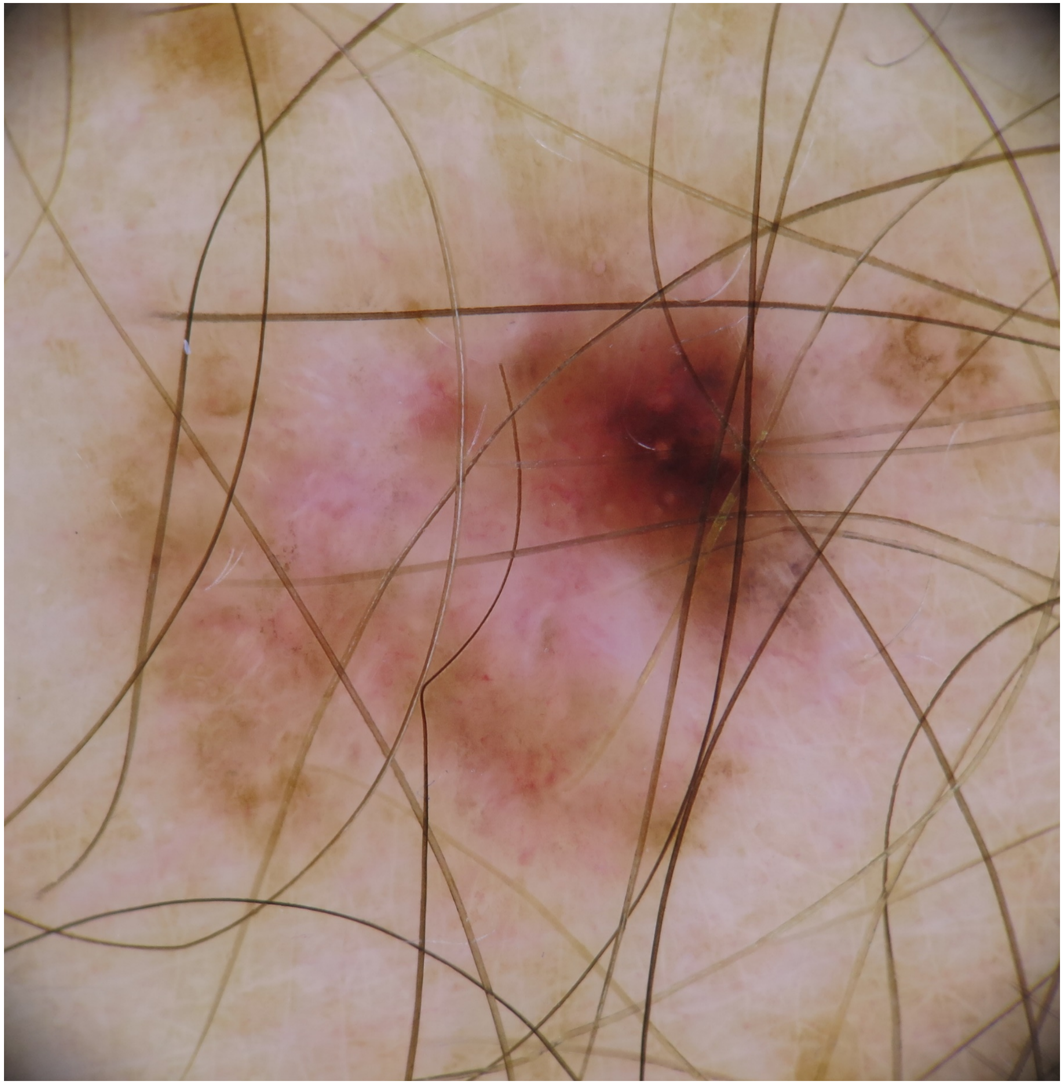

Location: Trunk

Invasive) Breslow interval: 0.6-0.8 mm

Case number 40

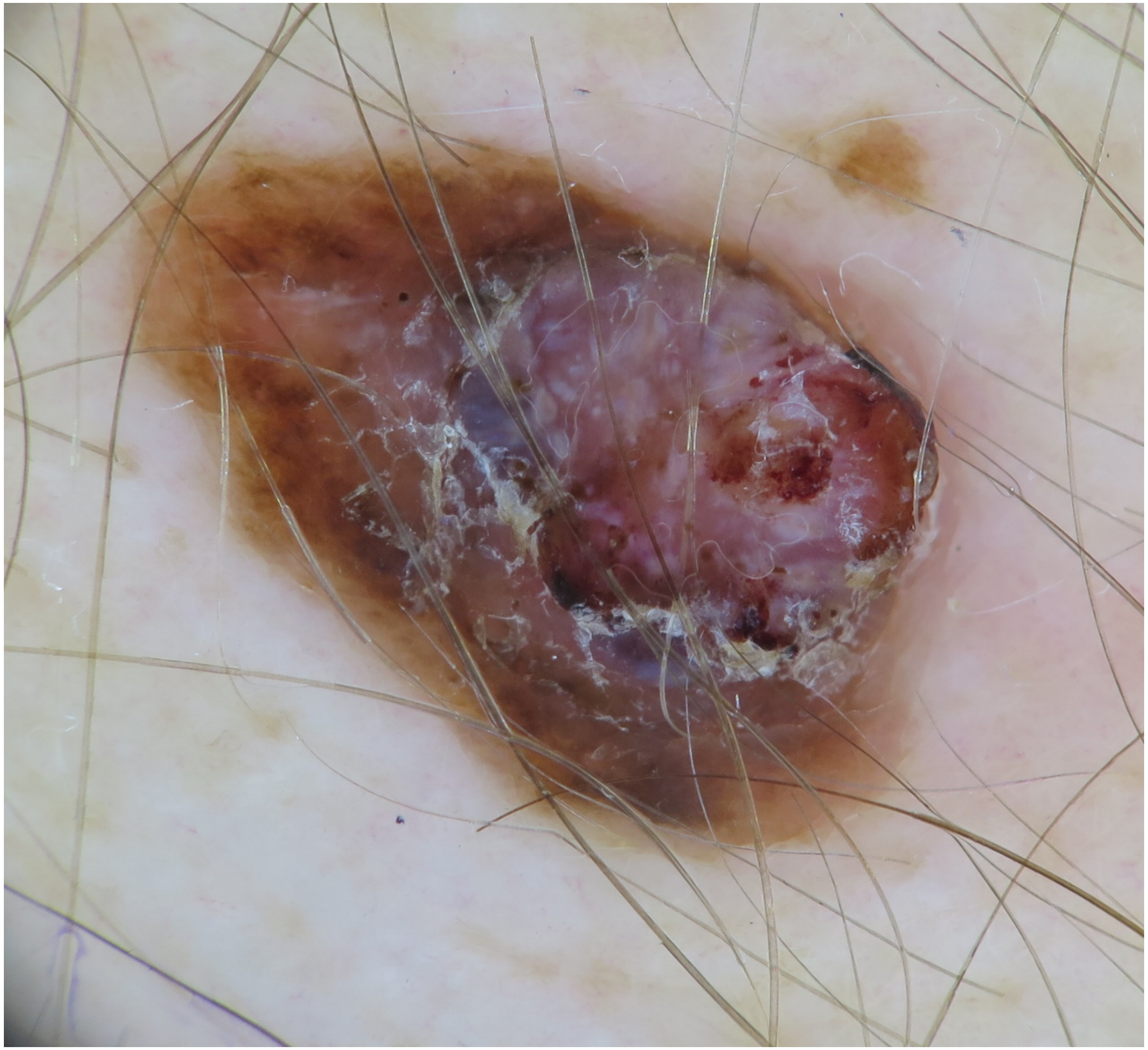

Location: Trunk

Invasive) Breslow interval: 1.1-2.0 mm

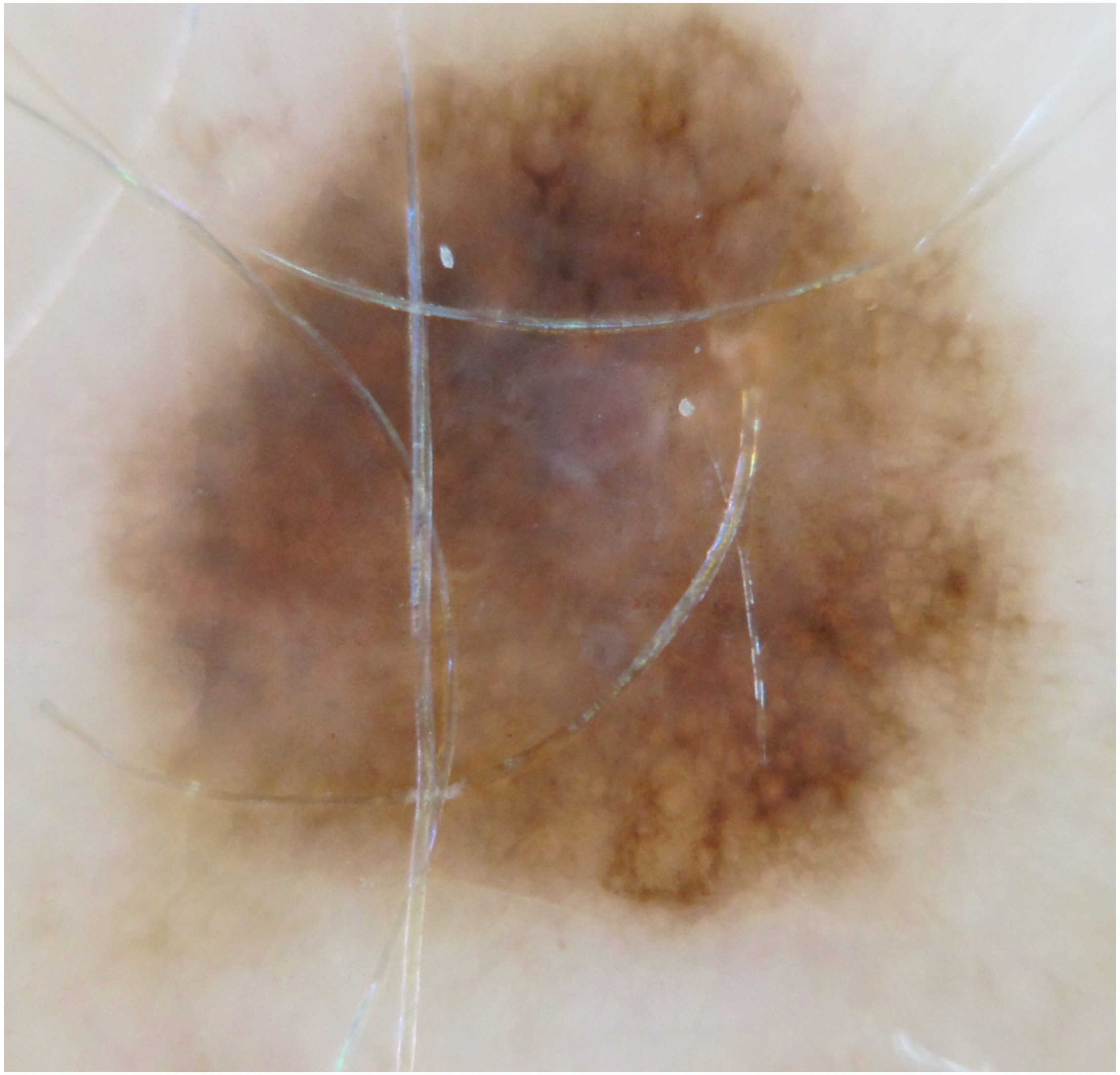

Location: Lower extremity

In situ melanoma

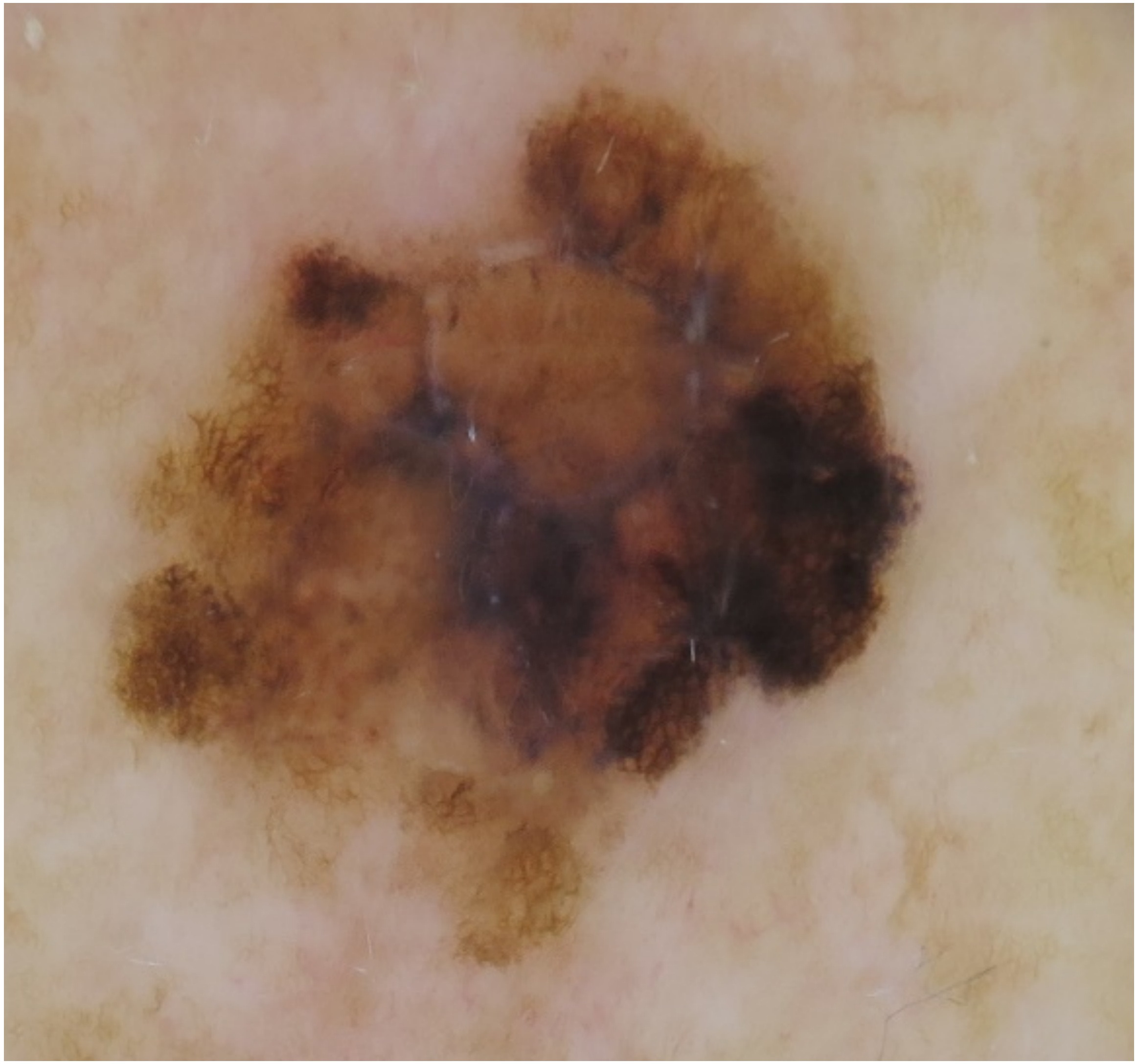

Location: Trunk

In situ melanoma

Case number 43

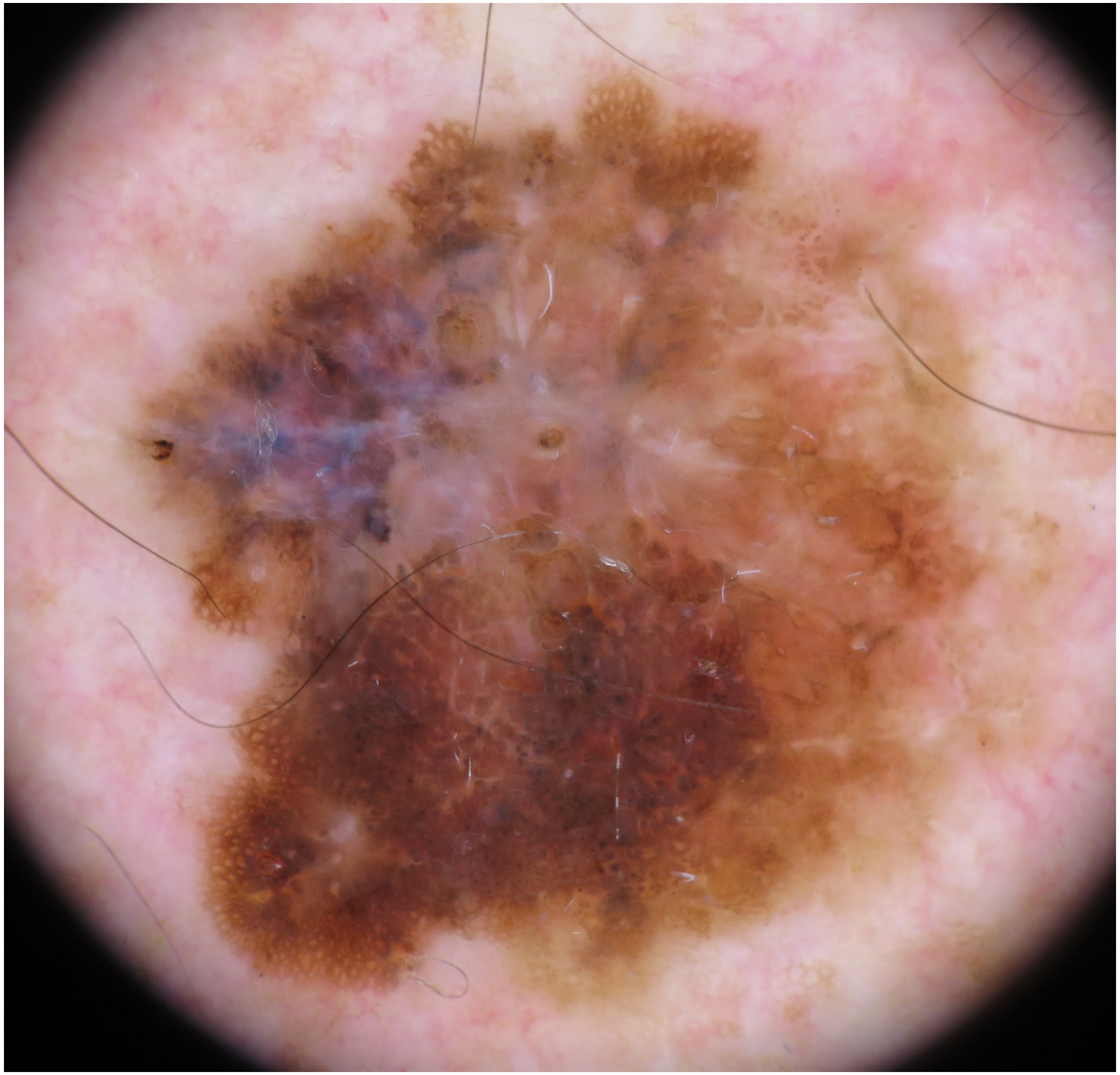

Location: Trunk

Invasive) Breslow interval: 0.1-0.5 mm

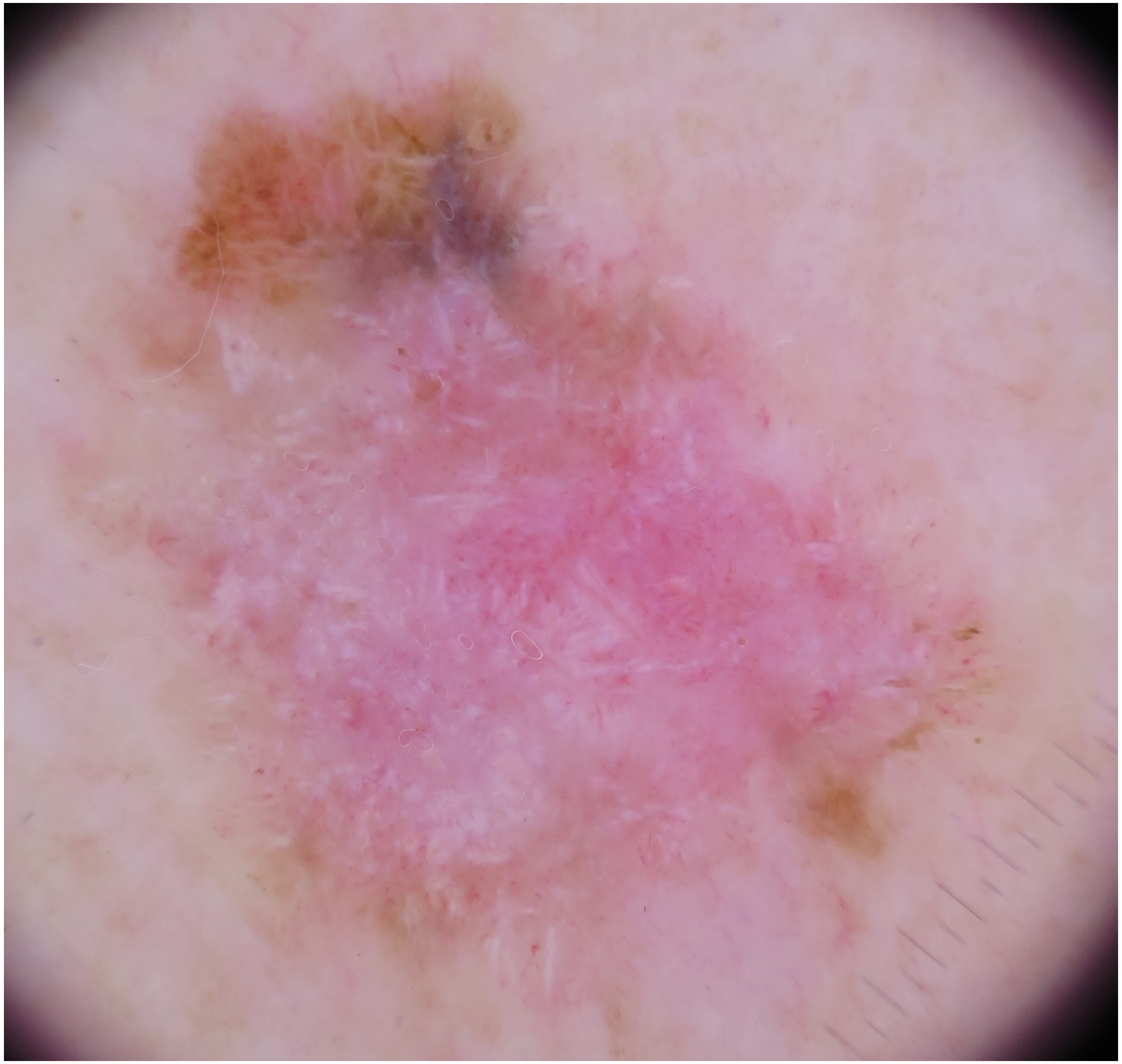

Location: Upper extremity

Invasive) Breslow interval: 1.1-2.0 mm

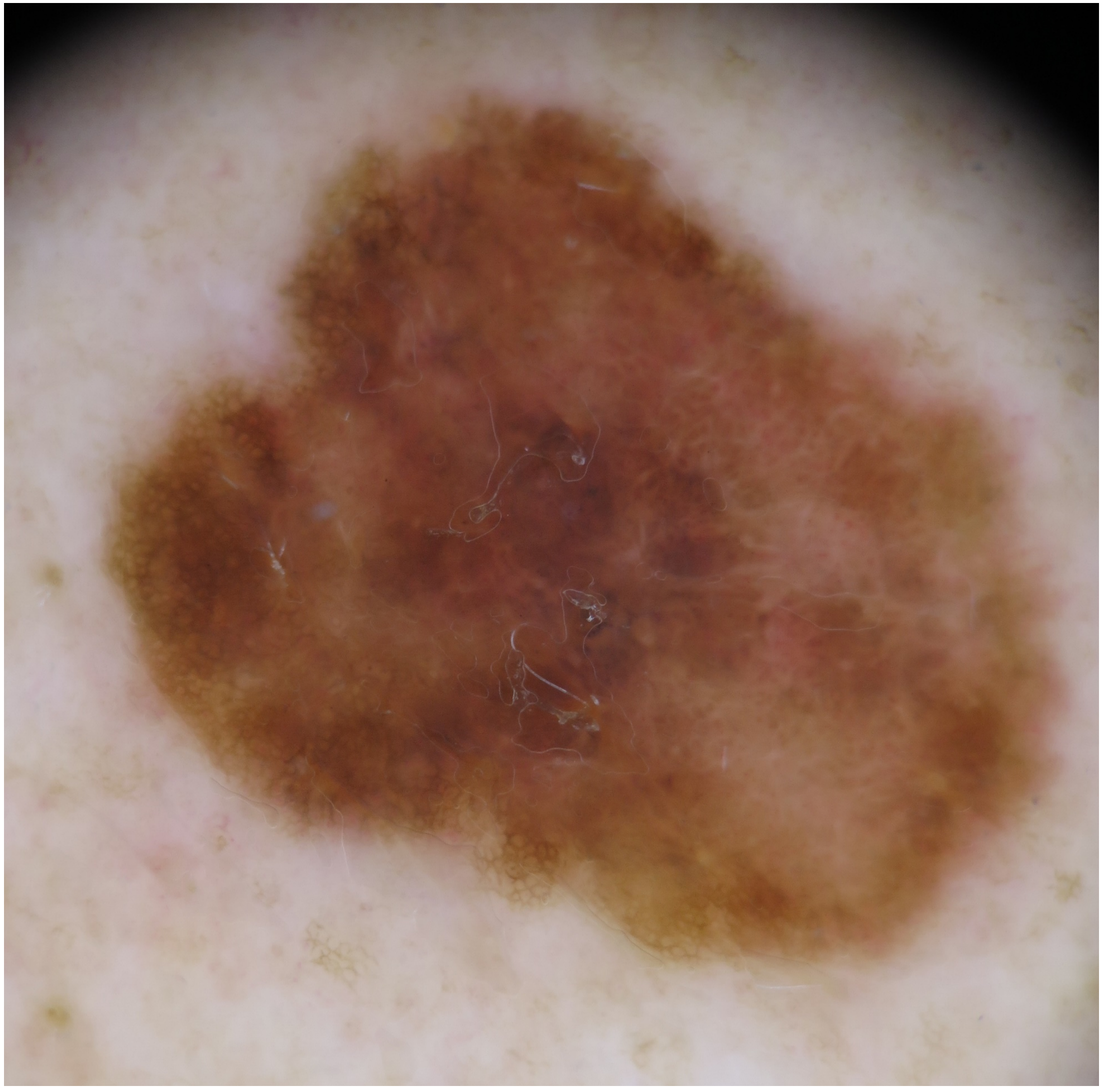

Location: Trunk

In situ melanoma

Case number 46

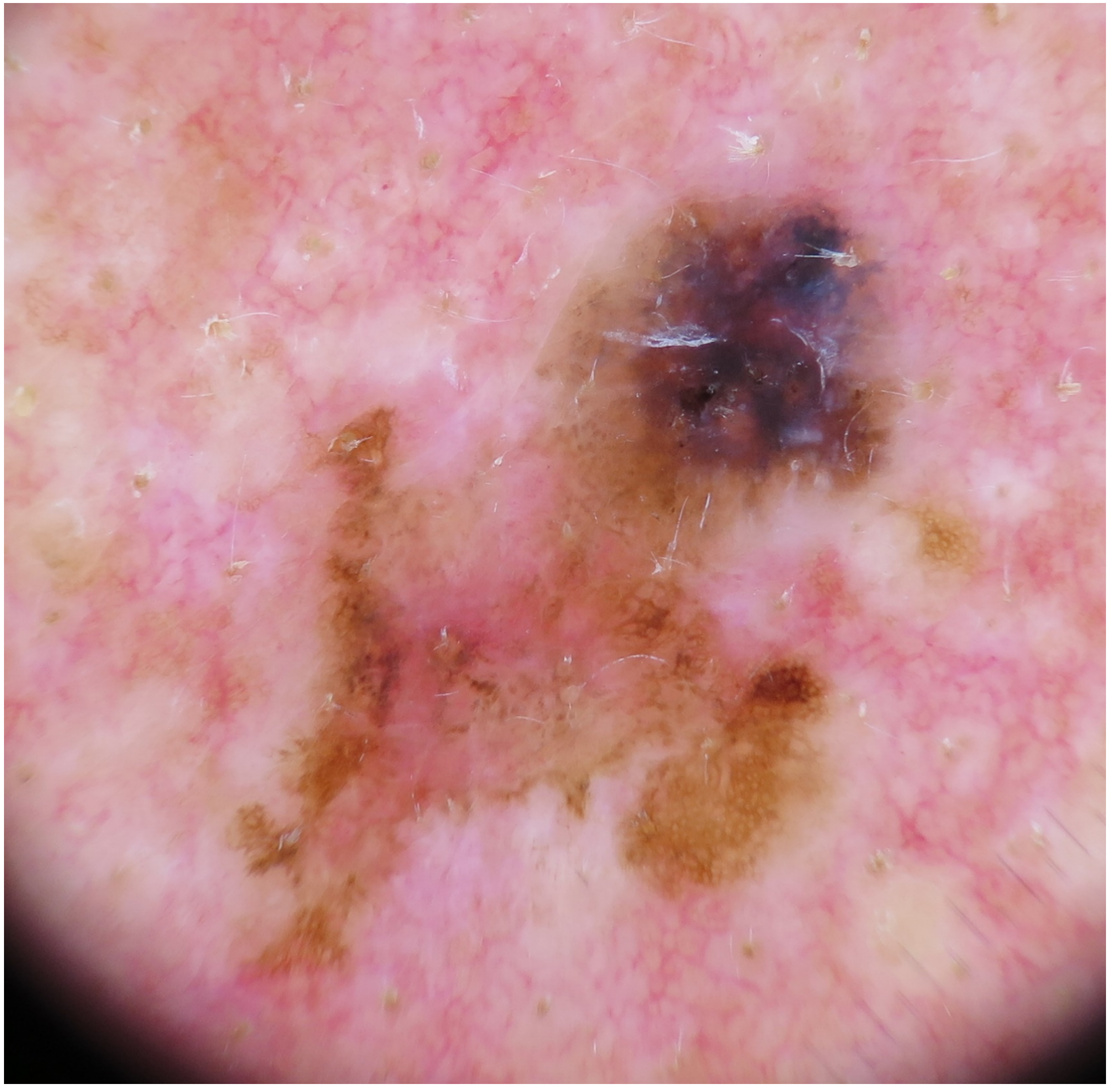

Location: Trunk

Invasive) Breslow interval: 0.9-1.0 mm

Case number 47

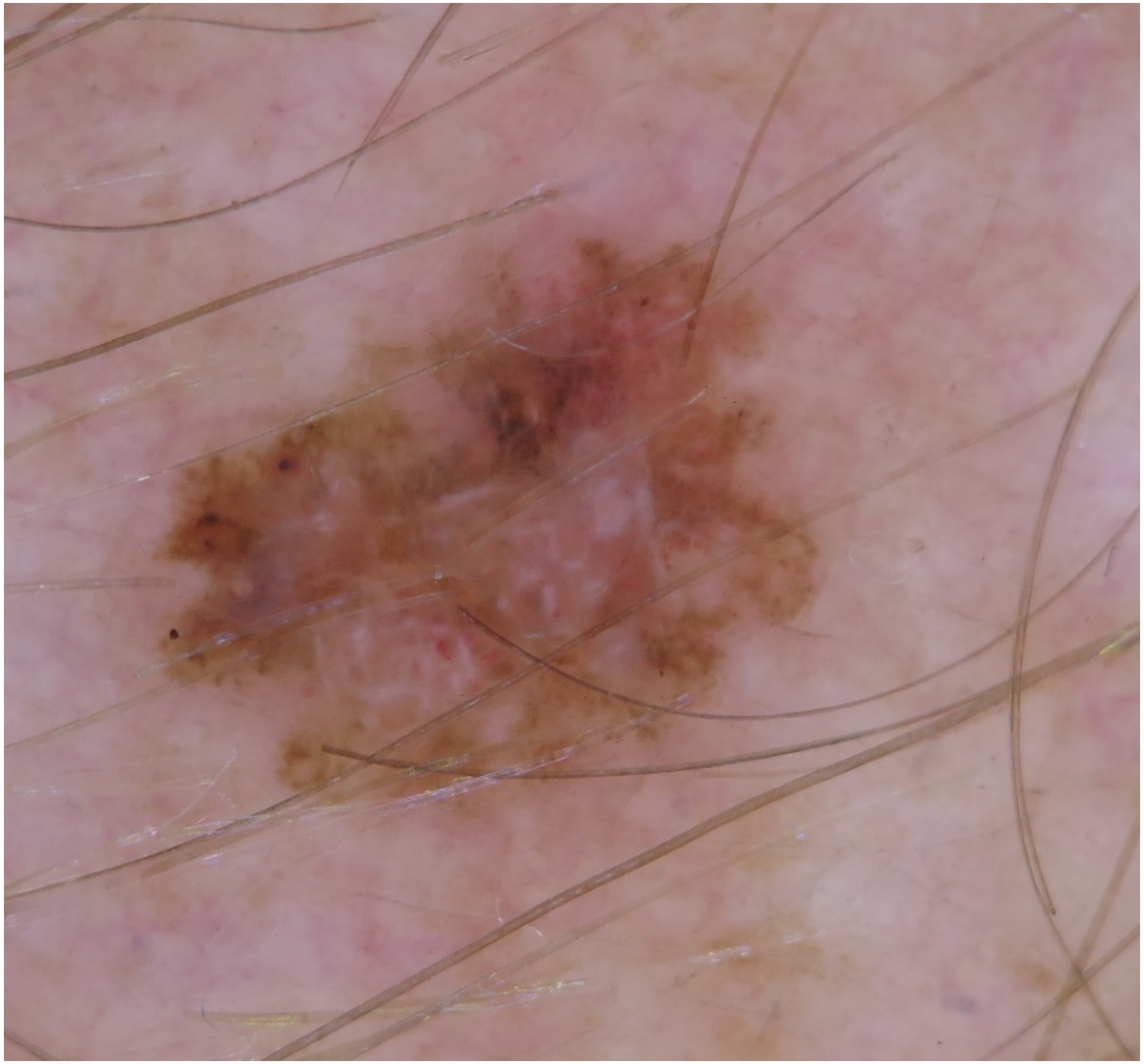

Location: Upper extremity

Invasive) Breslow interval: 0.6-0.8 mm

Case number 48

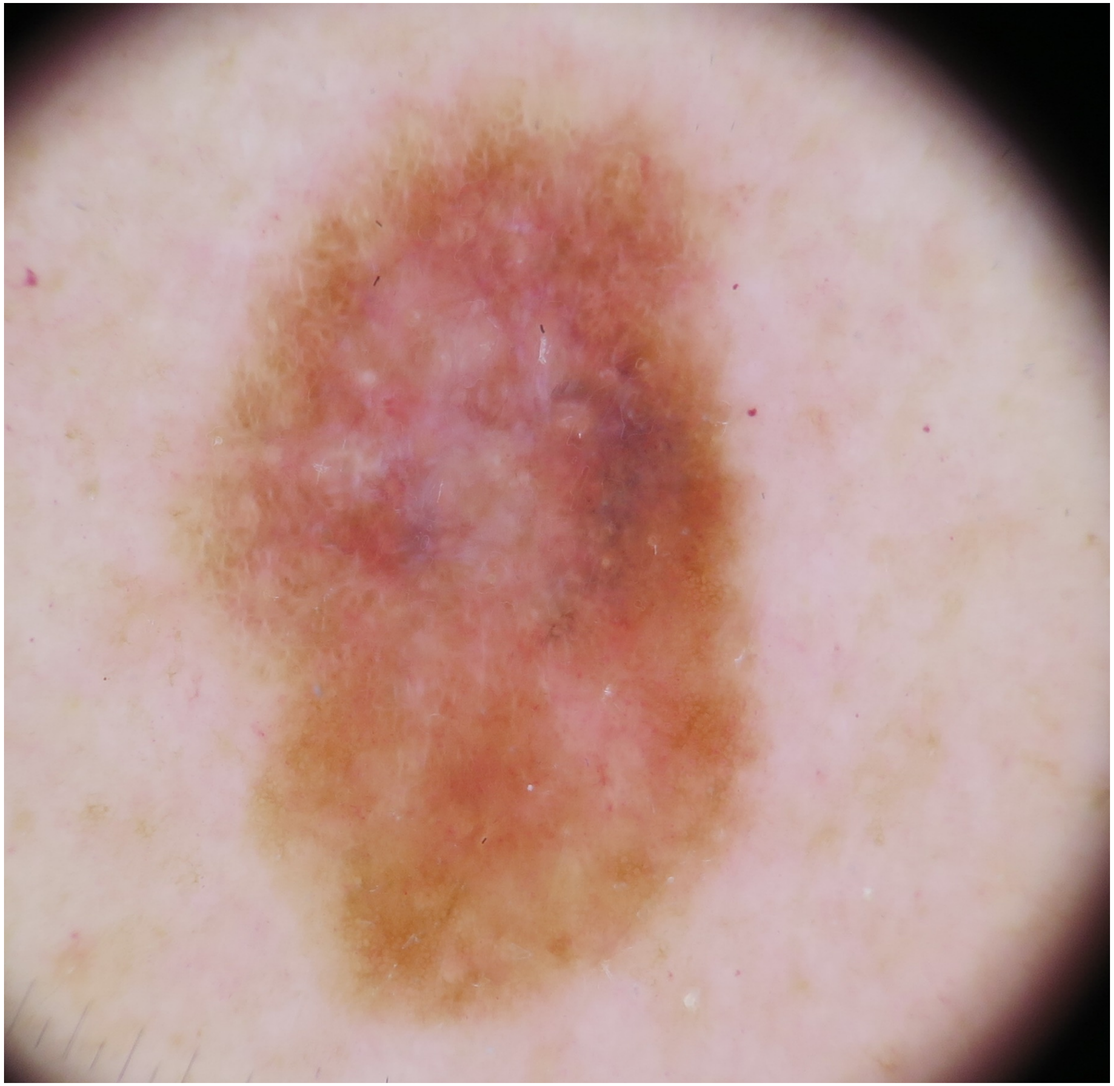

Location: Trunk

Invasive) Breslow interval: 0.1-0.5 mm

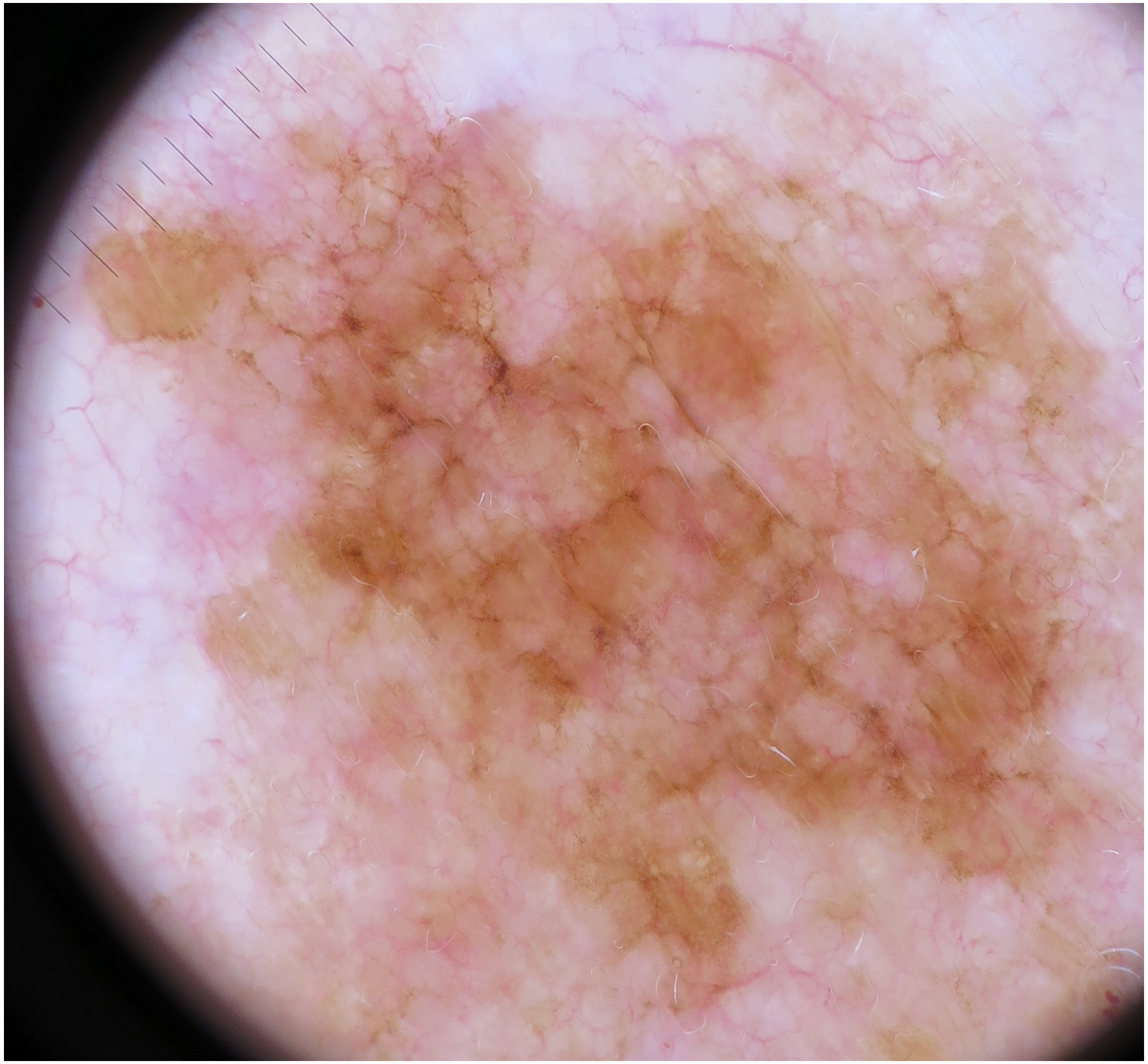

Location: Upper extremity

In situ melanoma

Case number 50

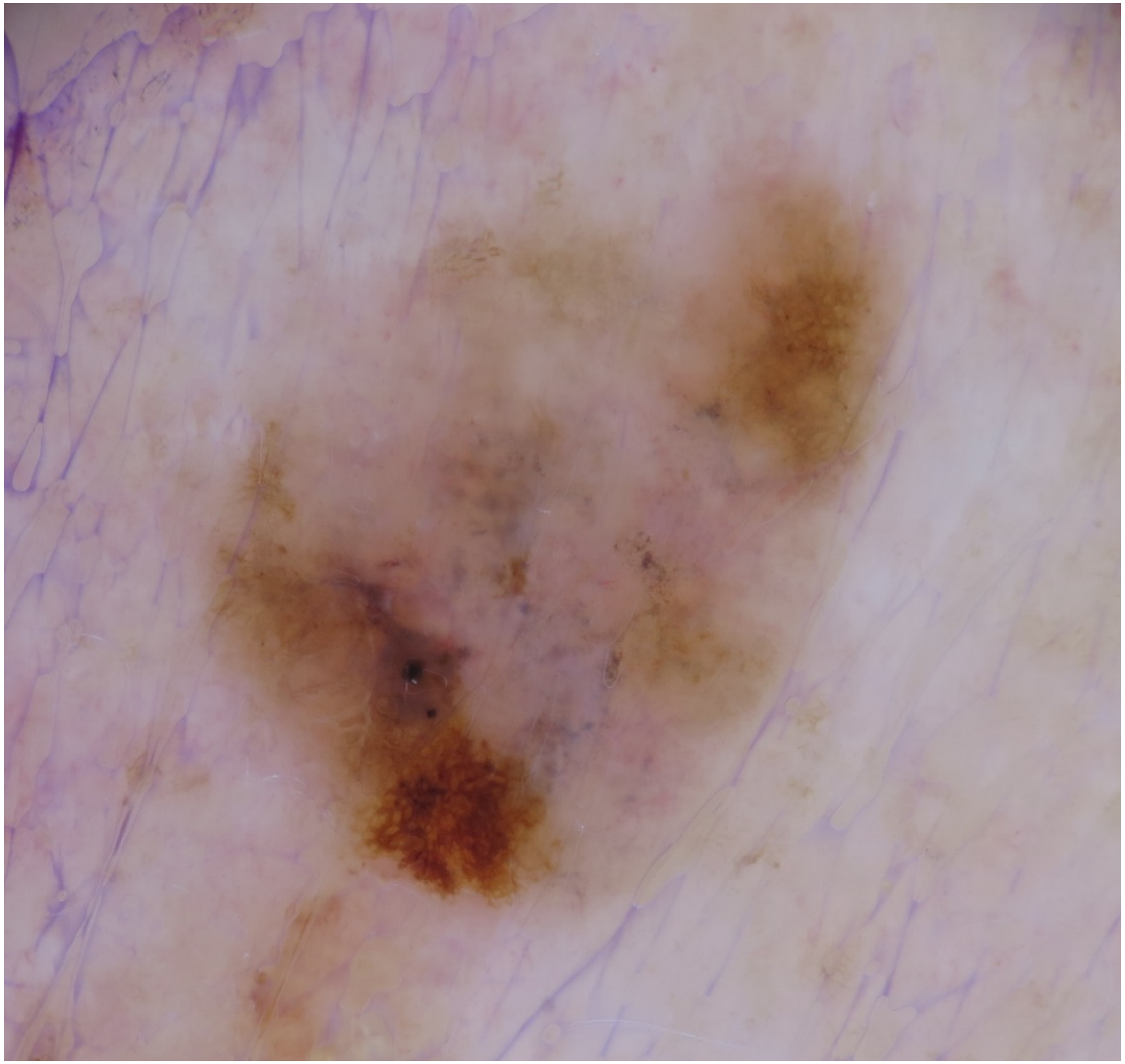

Location: Trunk

In situ melanoma

Case number 51

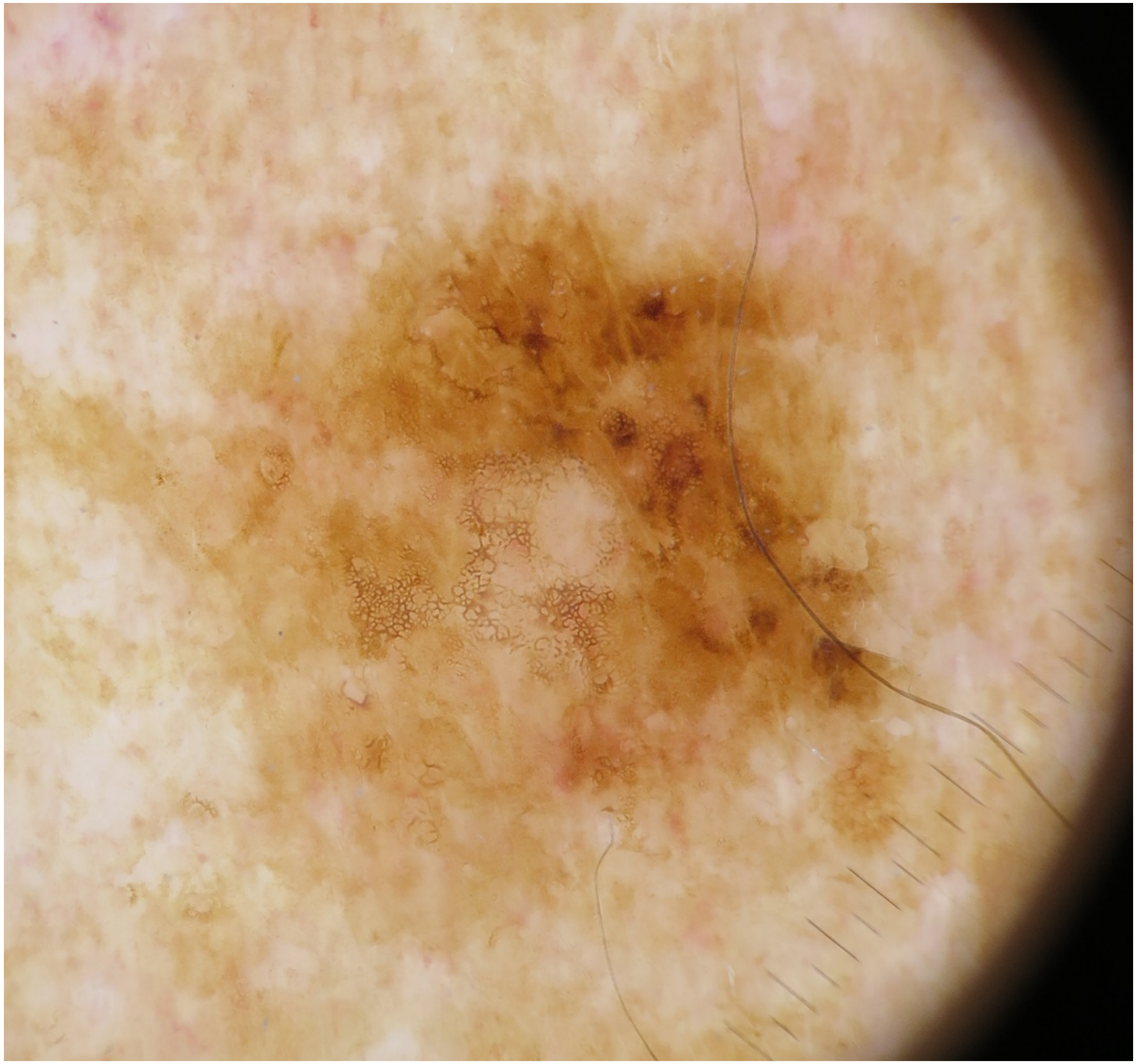

Location: Lower extremity

In situ melanoma

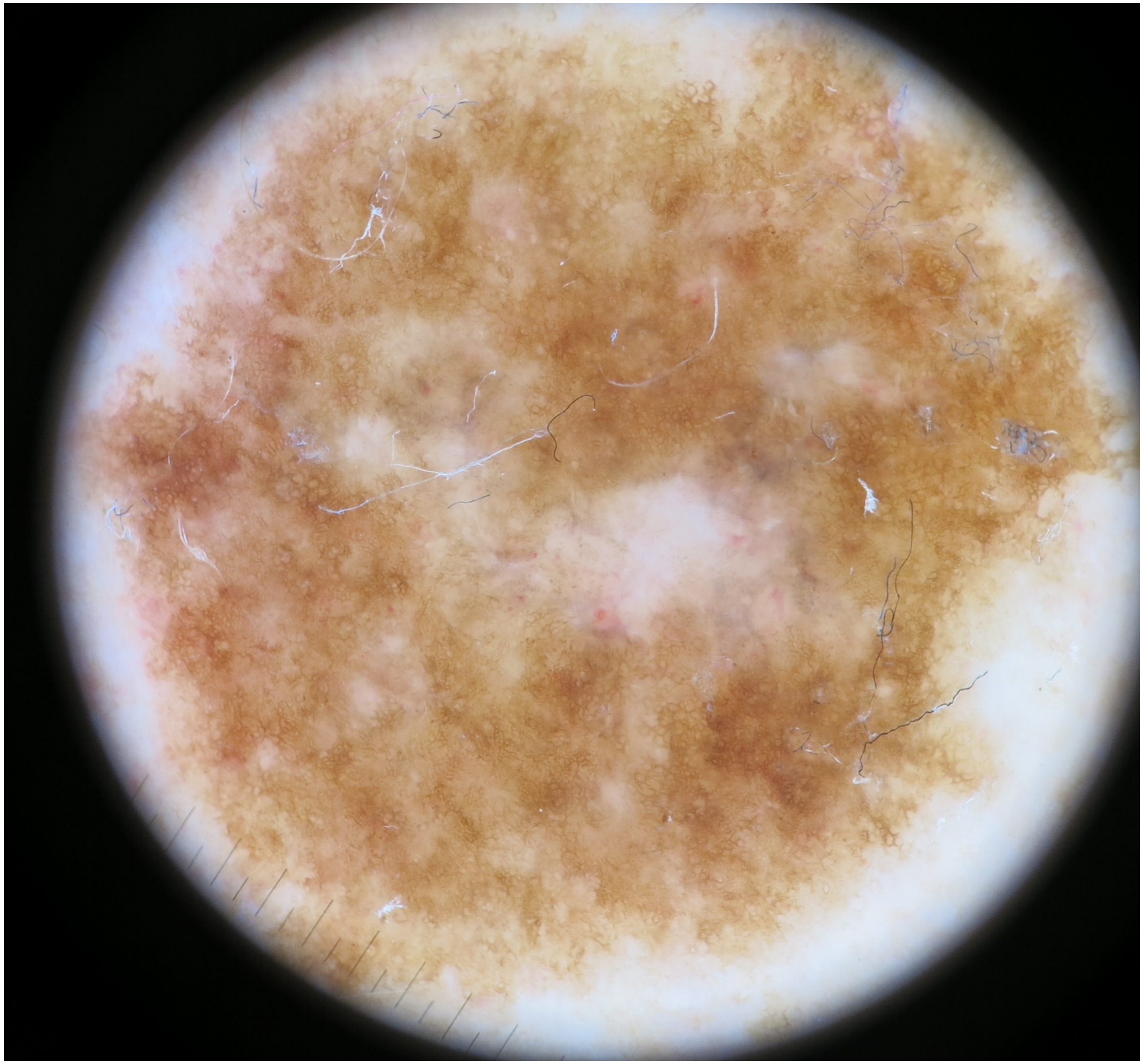

Location: Lower extremity

In situ melanoma

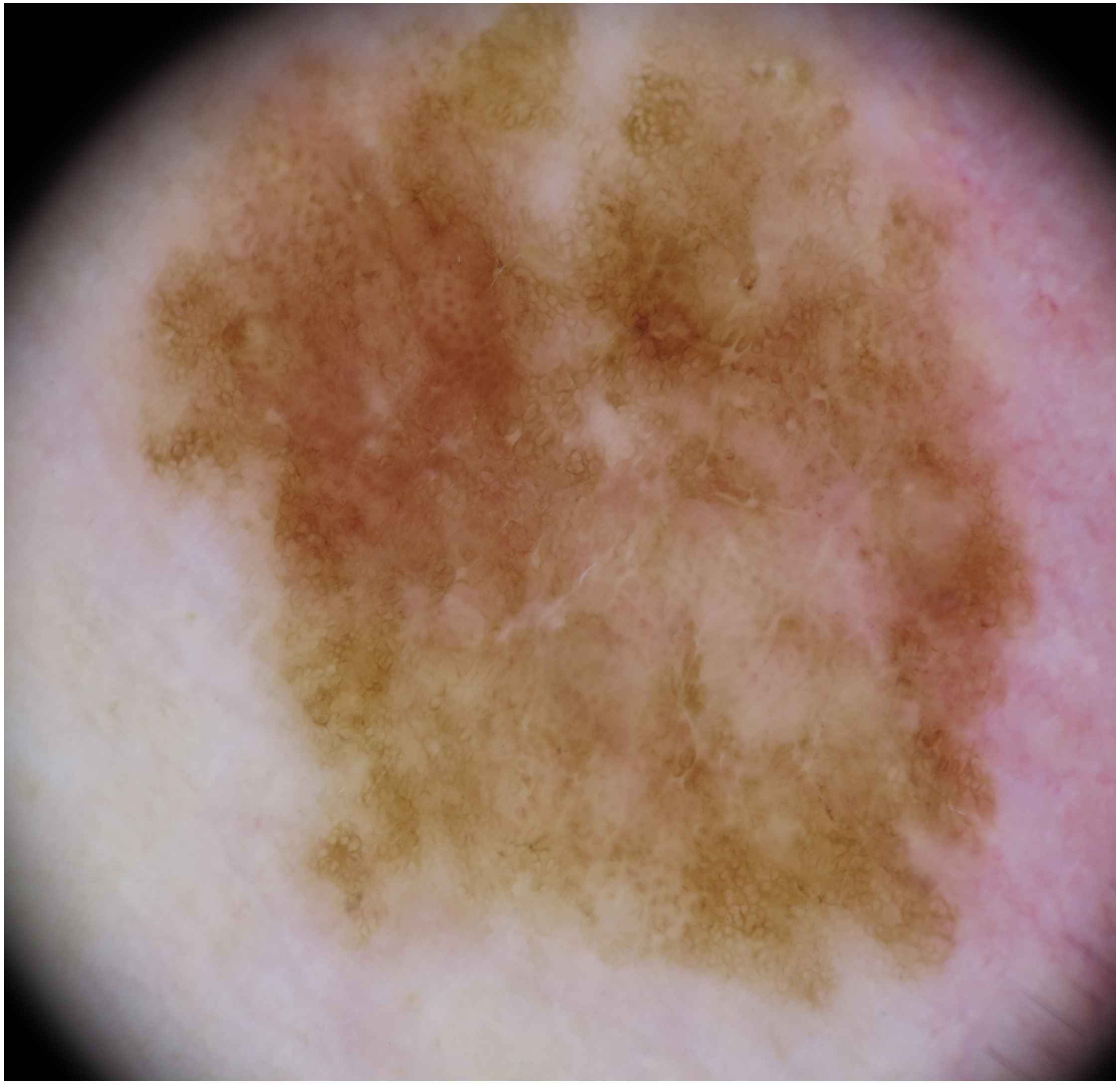

Location: Upper extremity

In situ melanoma

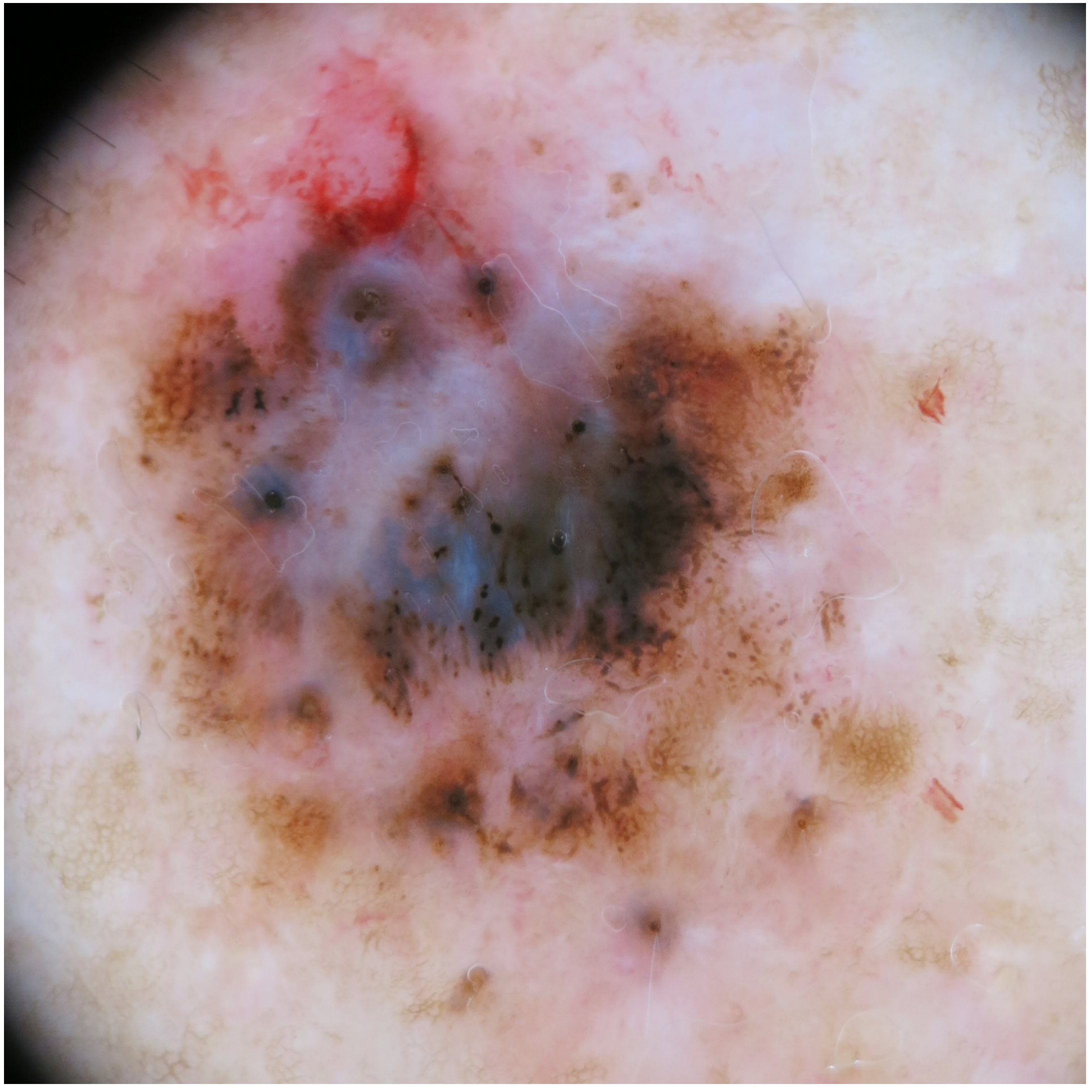

Location: Trunk

Invasive) Breslow interval: 0.6-0.8 mm

Case number 55

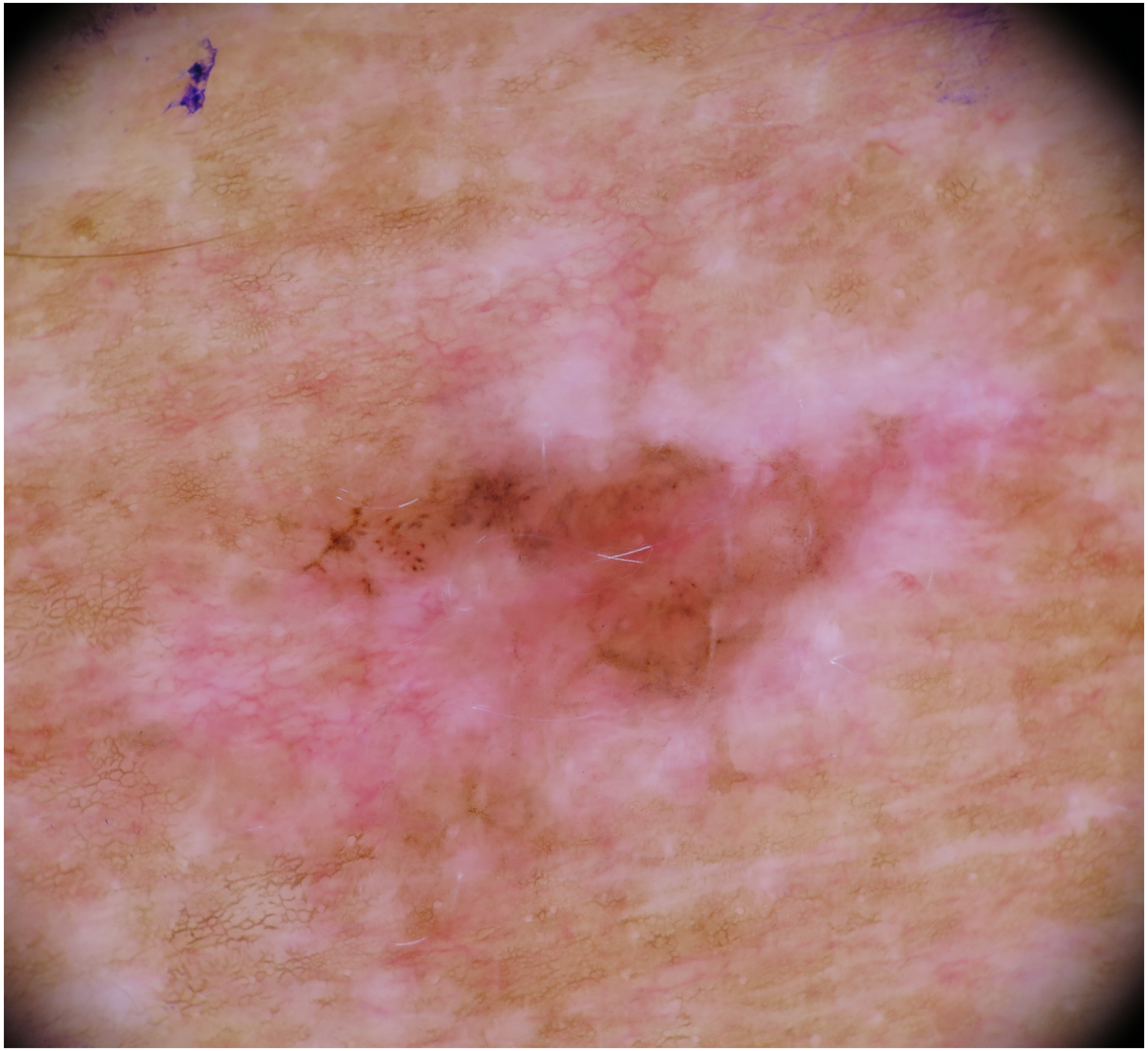

Location: Trunk

Invasive) Breslow interval: 0.1-0.5 mm

Case number 56

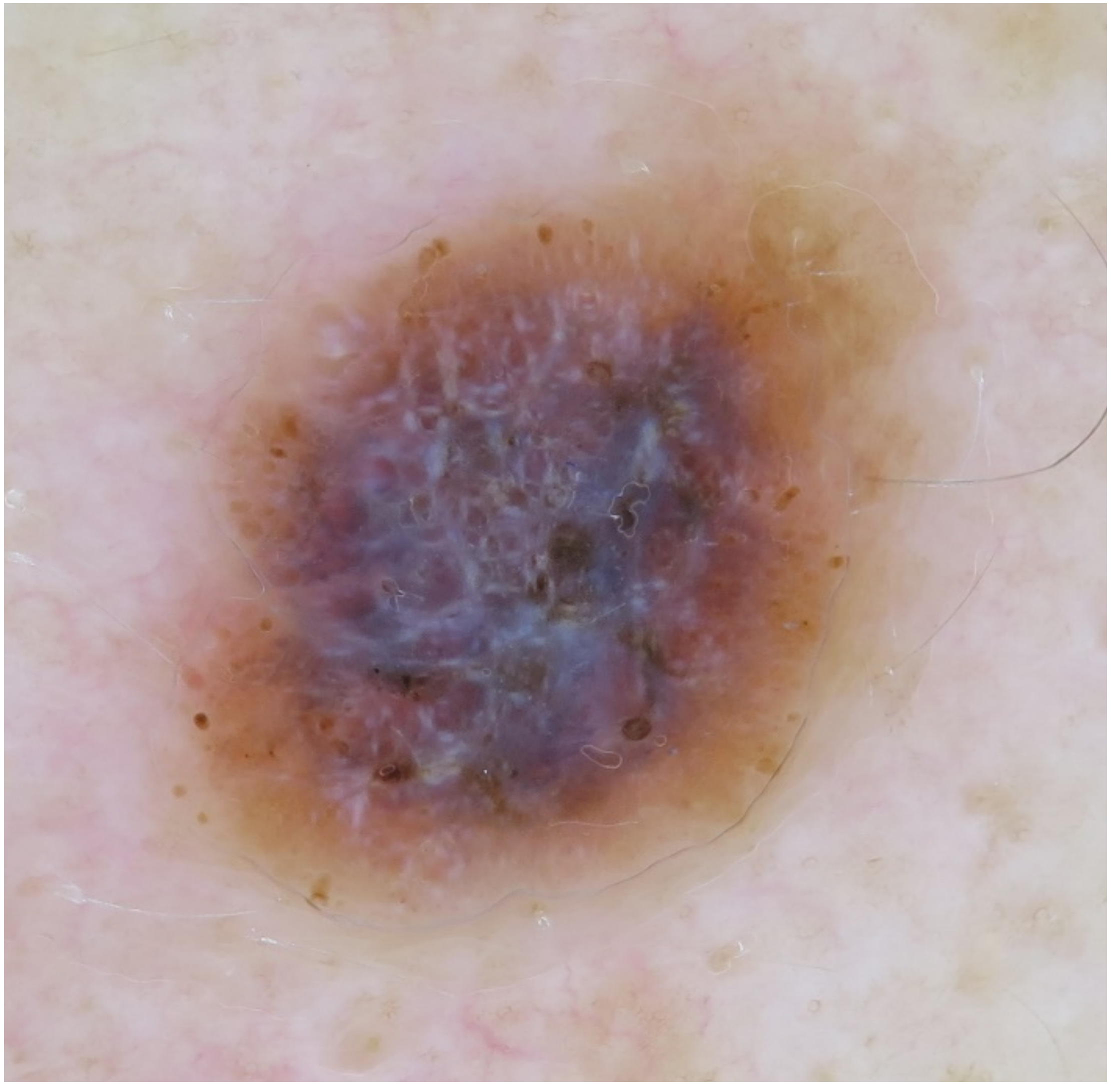

Location: Trunk

Invasive) Breslow interval: 1.1-2.0 mm

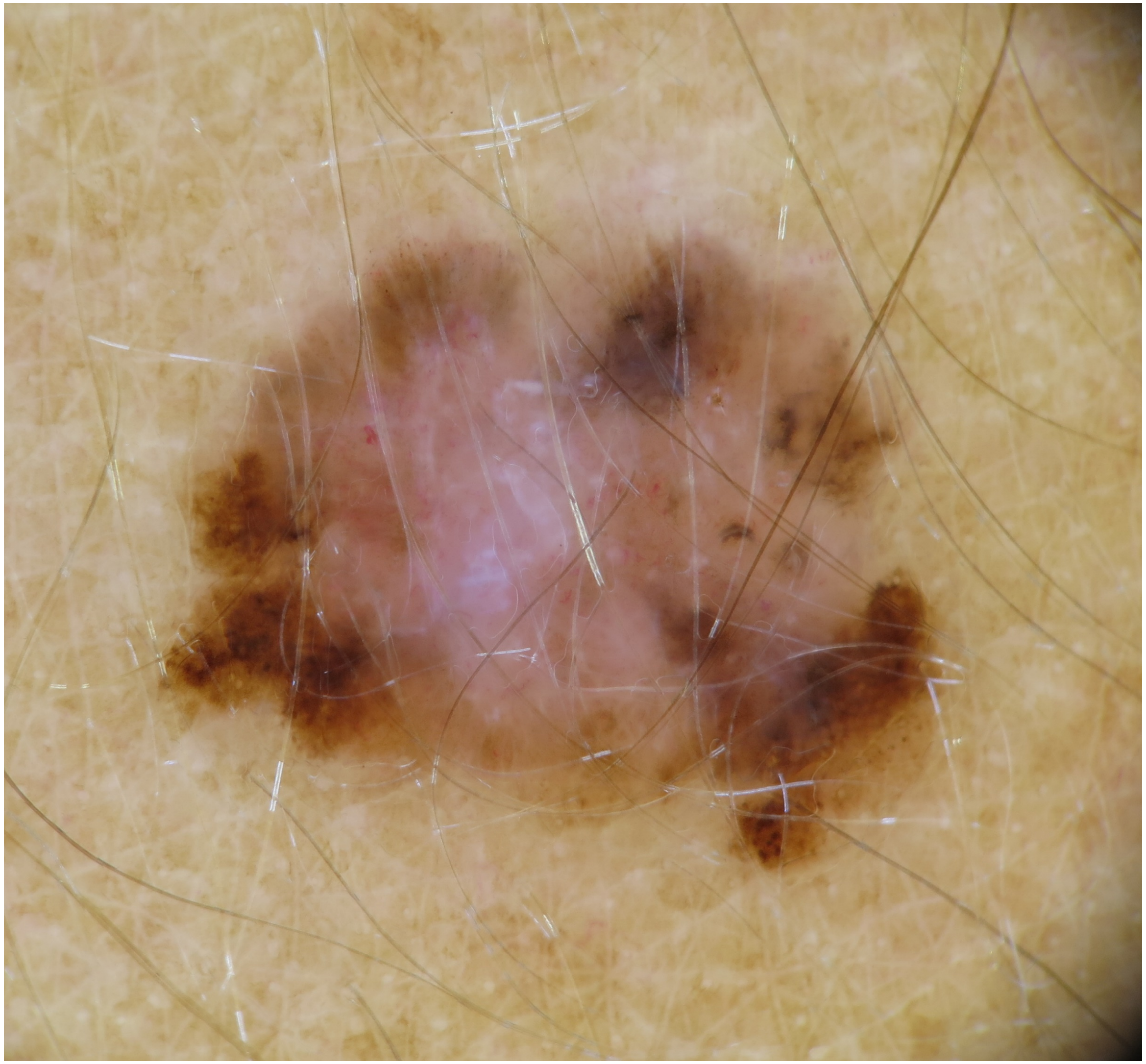

Location: Upper extremity

Invasive) Breslow interval: 0.9-1.0 mm

Case number 58

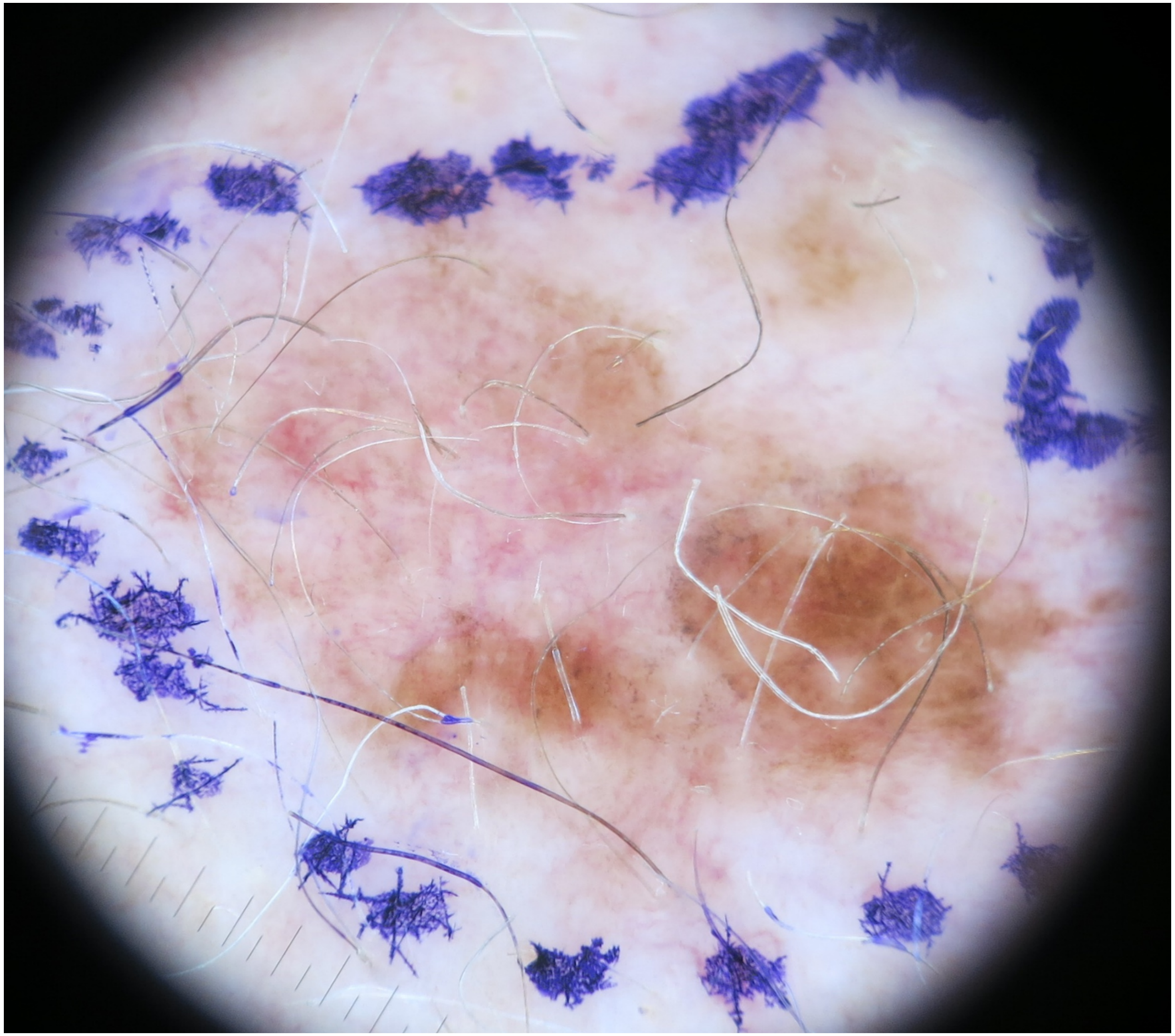

Location: Upper extremity

Invasive) Breslow interval: 0.1-0.5 mm

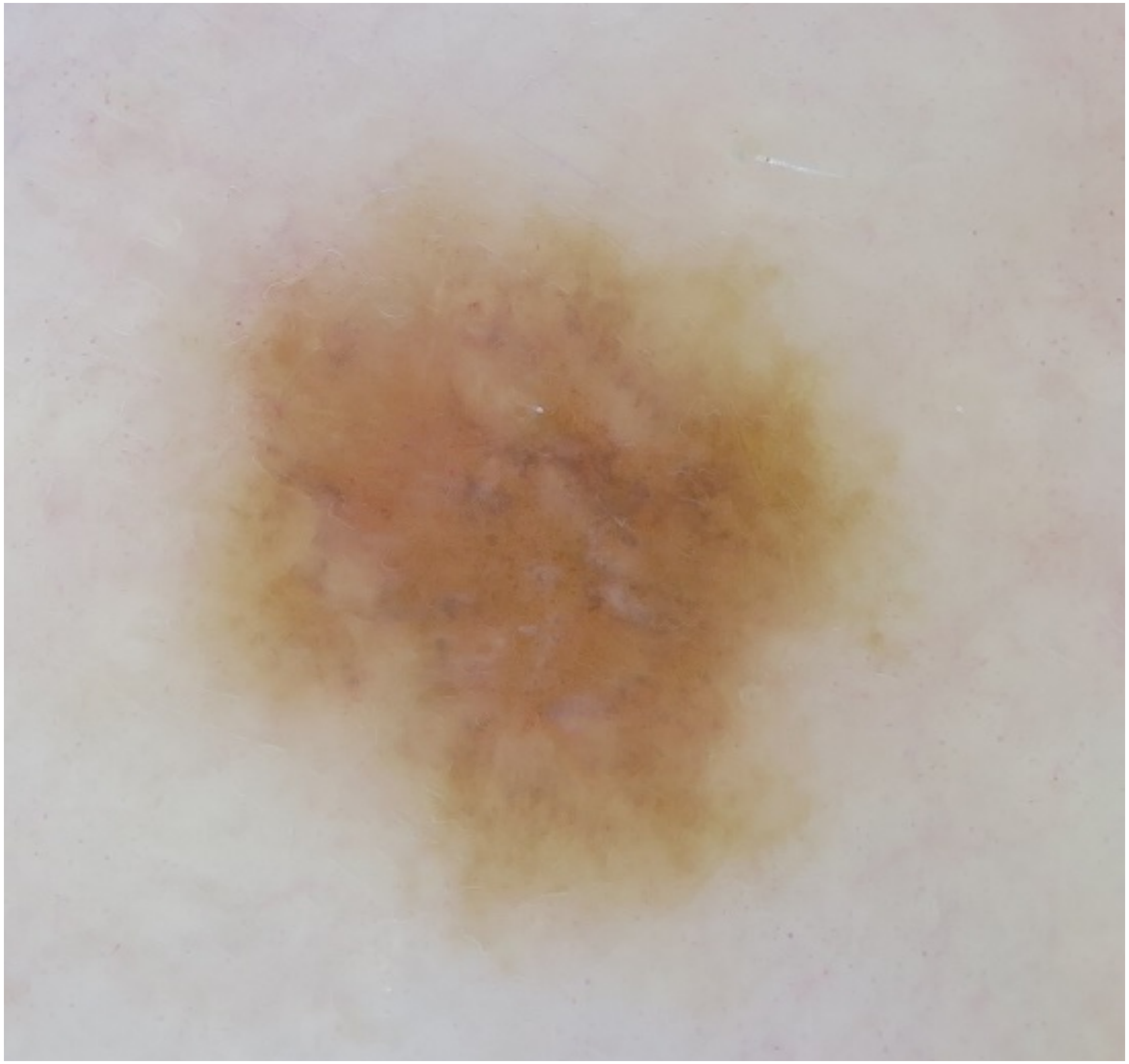

Location: Lower extremity

In situ melanoma

Case number 60

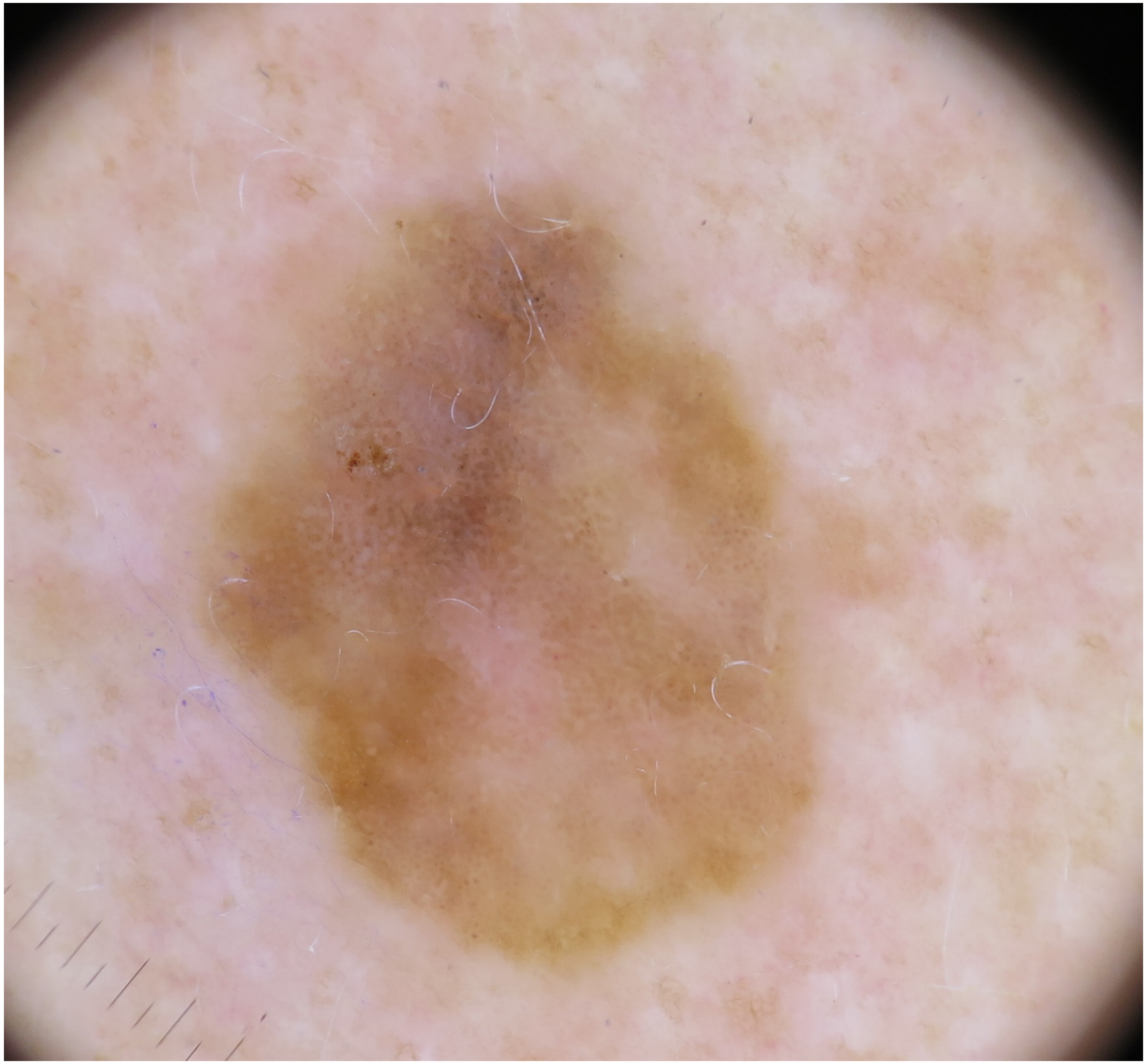

Location: Trunk

In situ melanoma

Case number 61

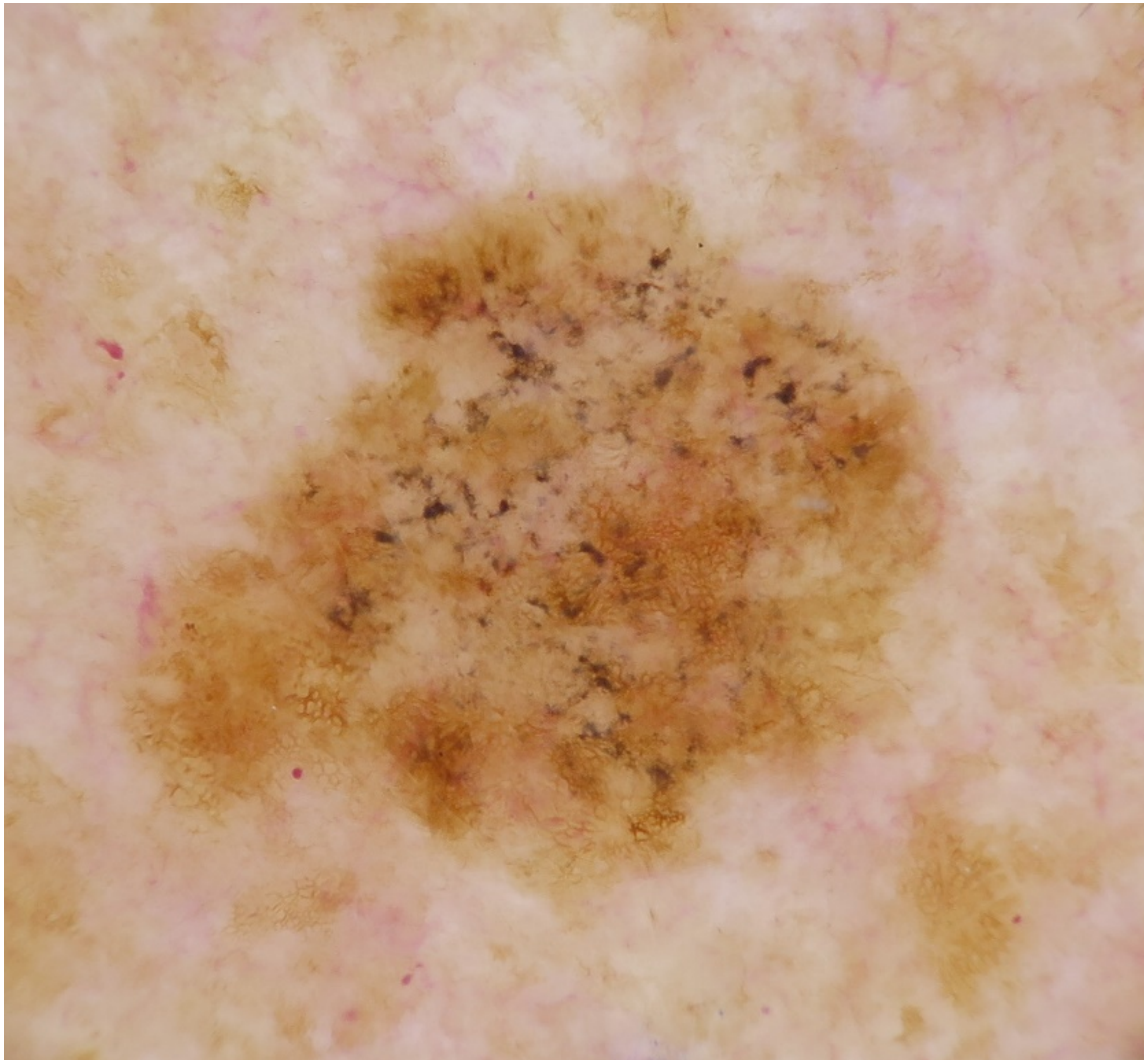

Location: Upper extremity

In situ melanoma

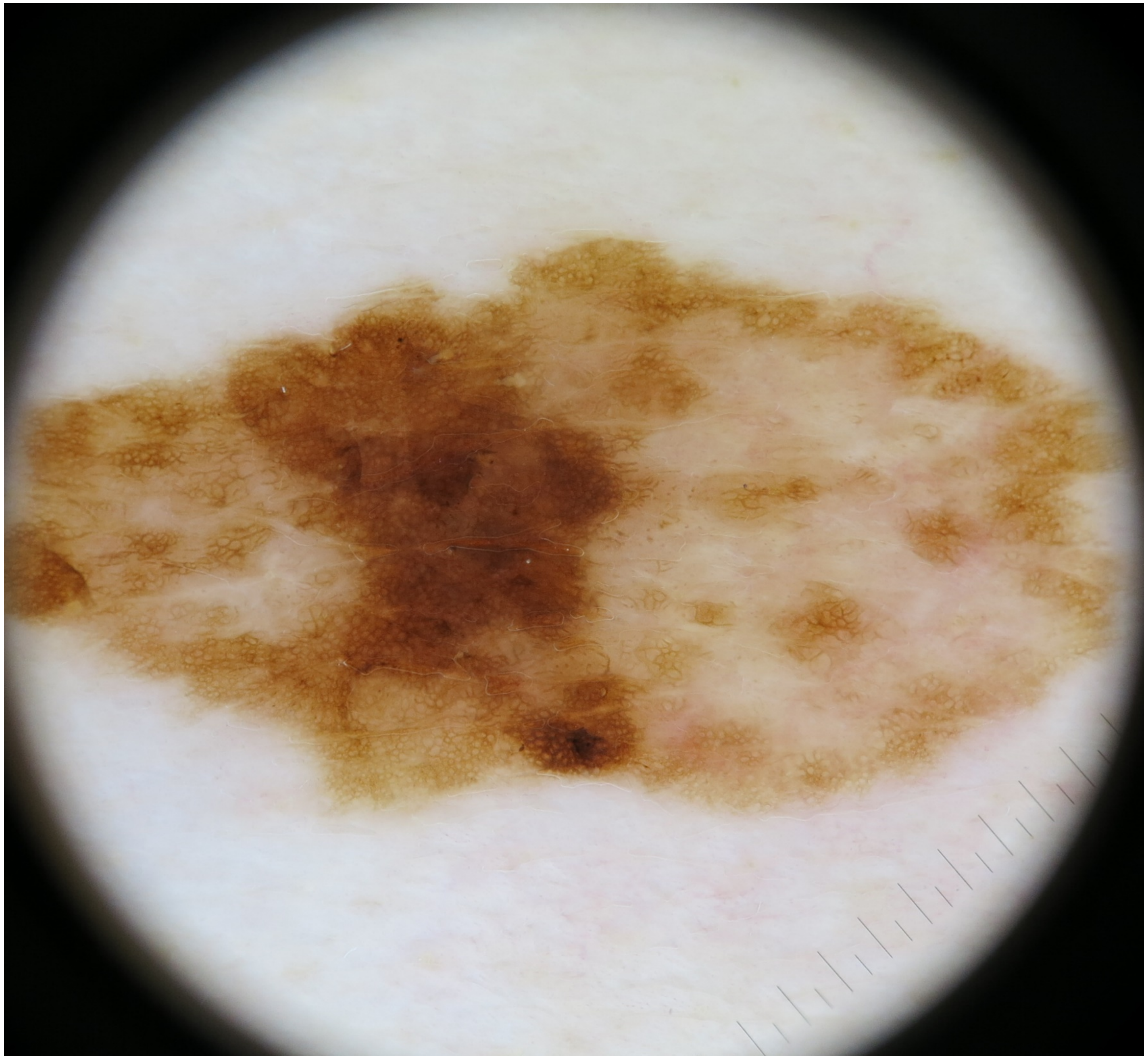

Location: Trunk

In situ melanoma

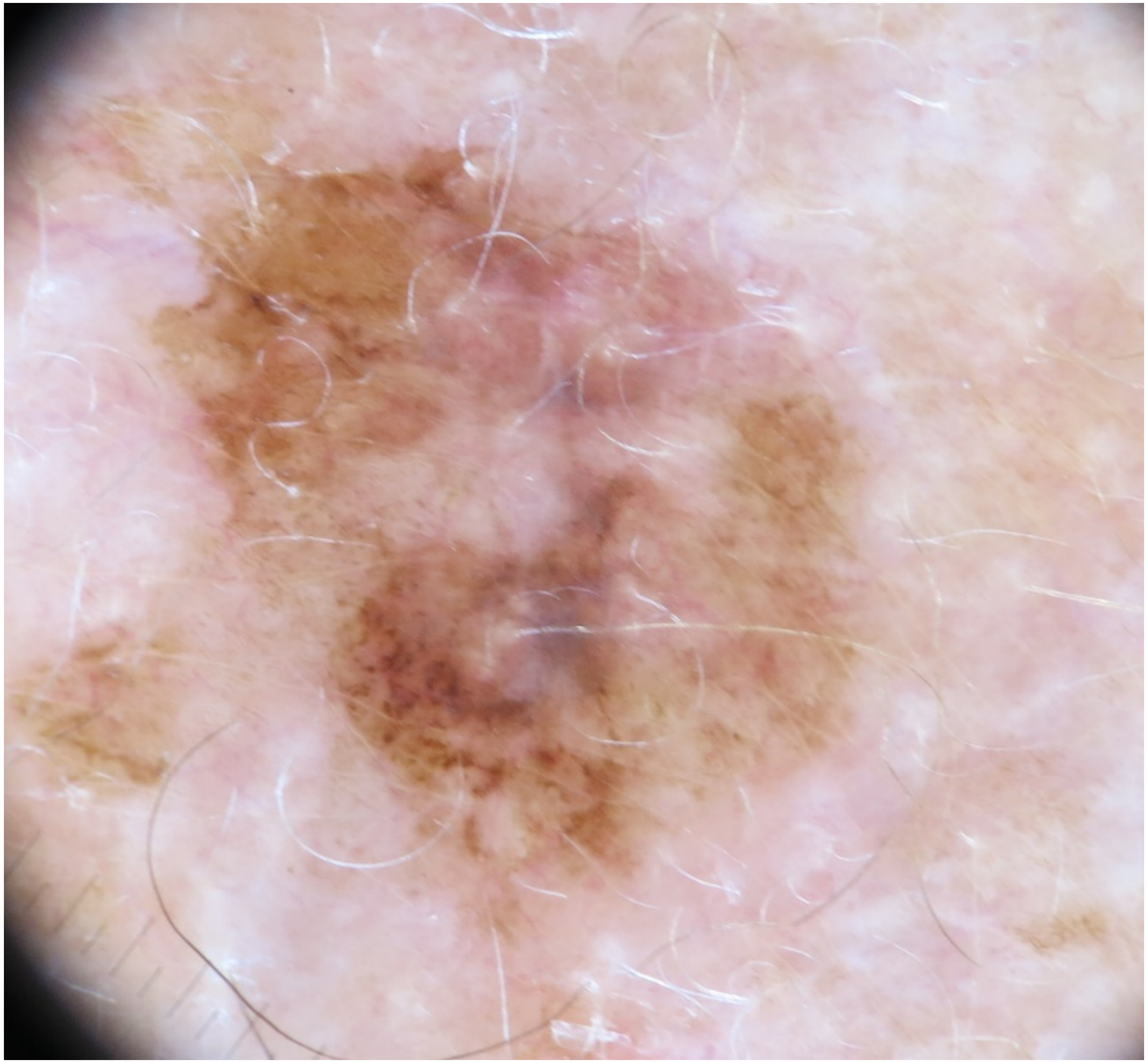

Location: Upper extremity

In situ melanoma

Case number 64

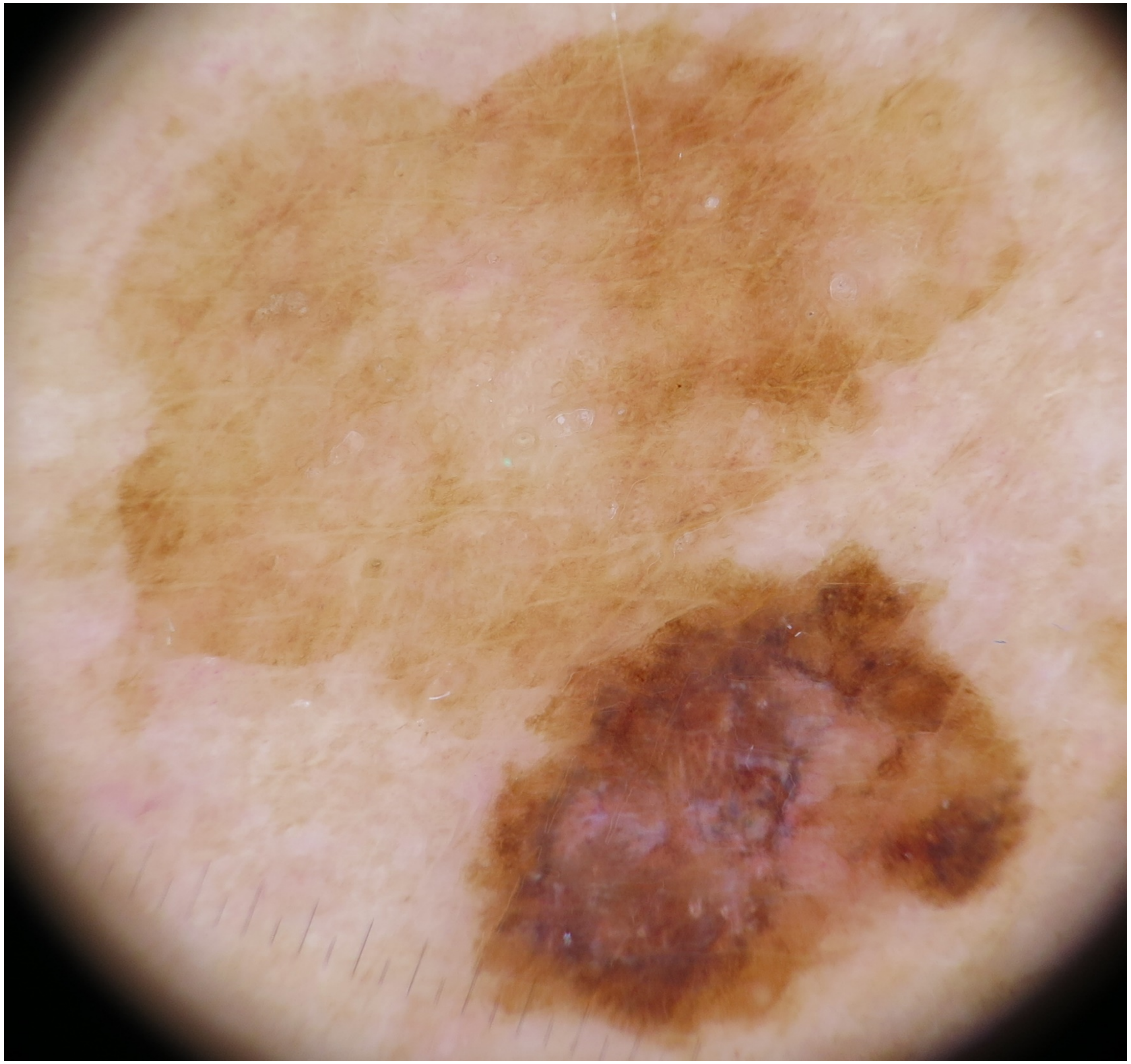

Location: Lower extremity

In situ melanoma

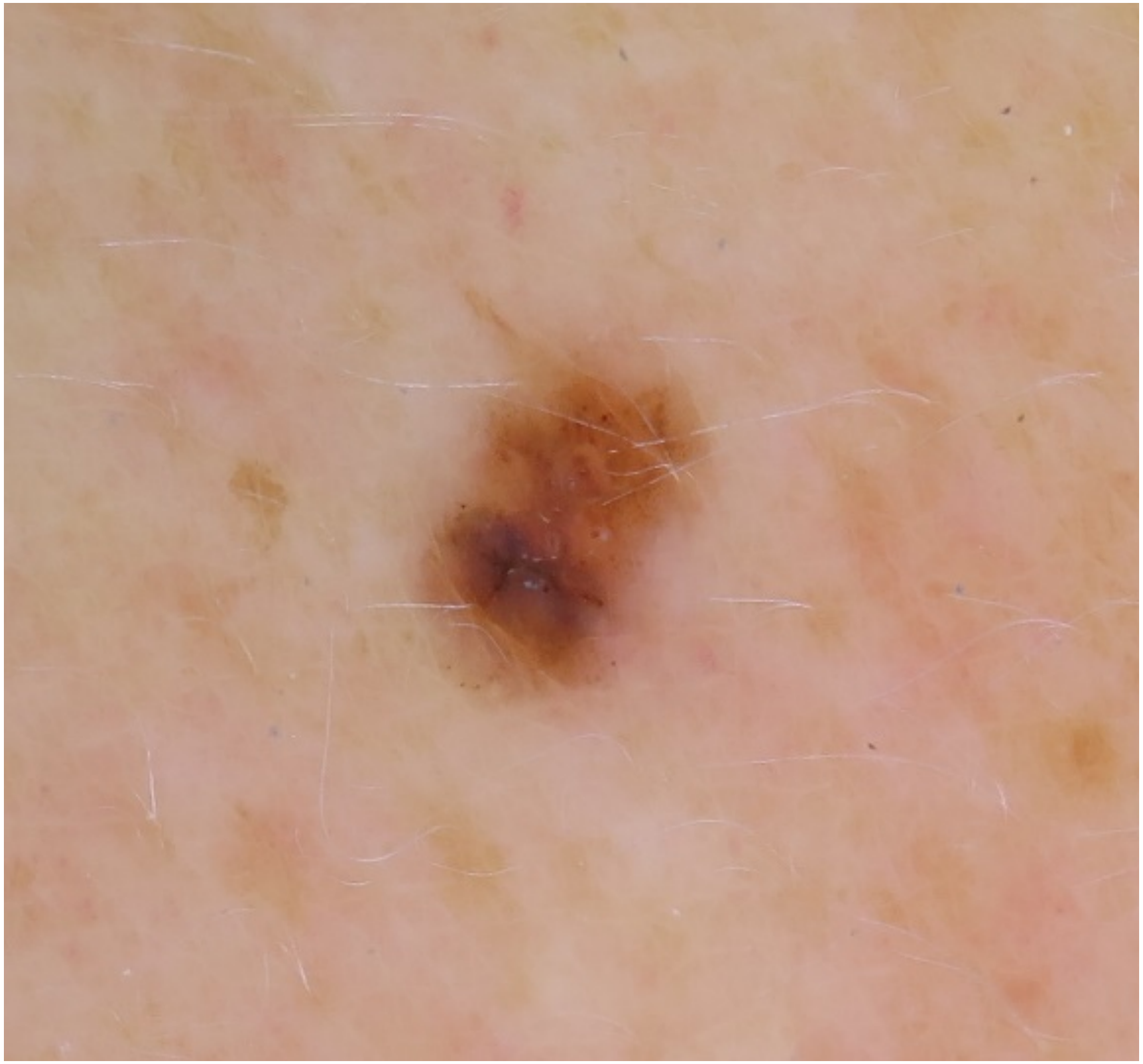

Location: Upper extremity

Invasive) Breslow interval: 0.1-0.5 mm

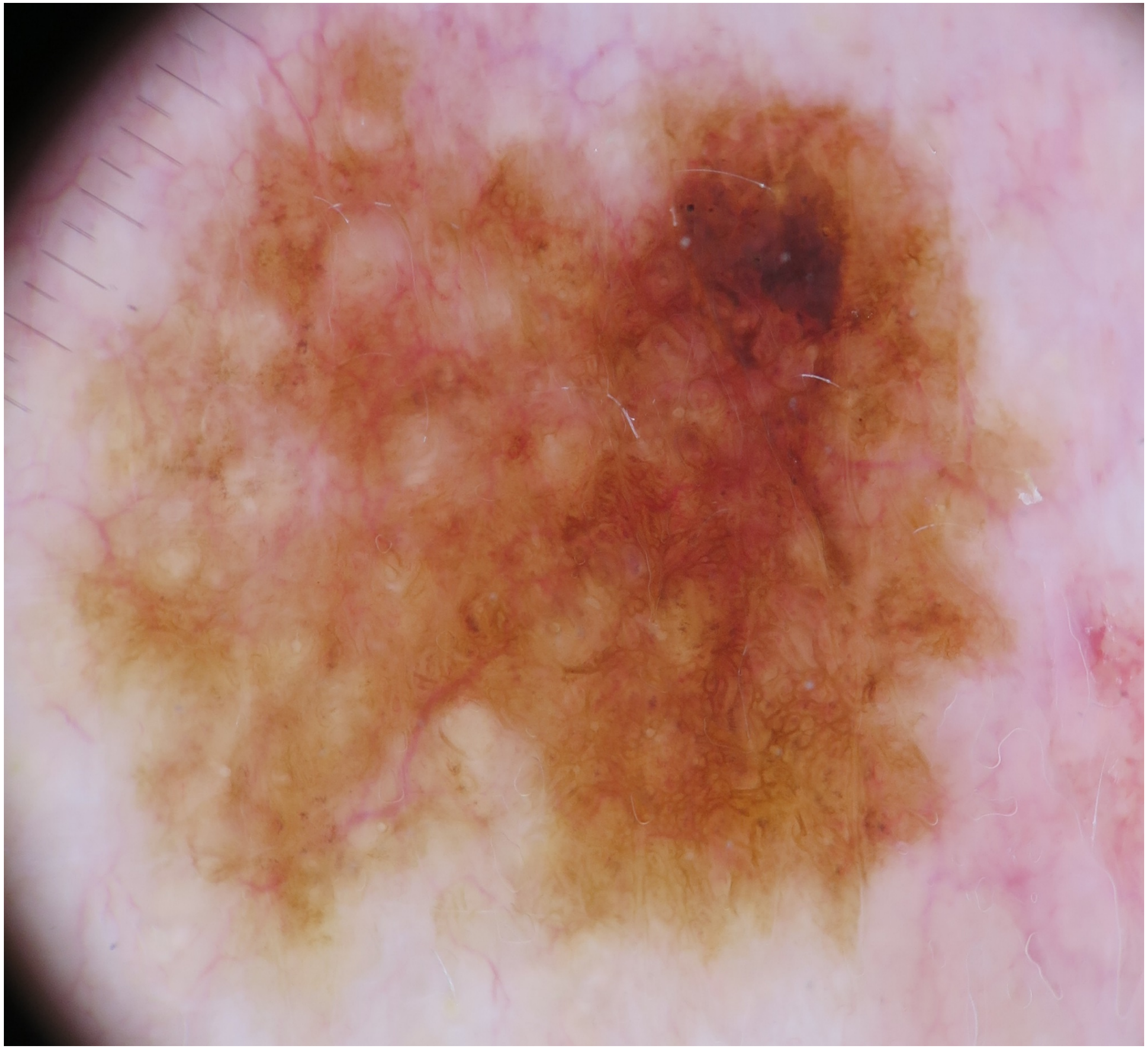

Location: Upper extremity

In situ melanoma

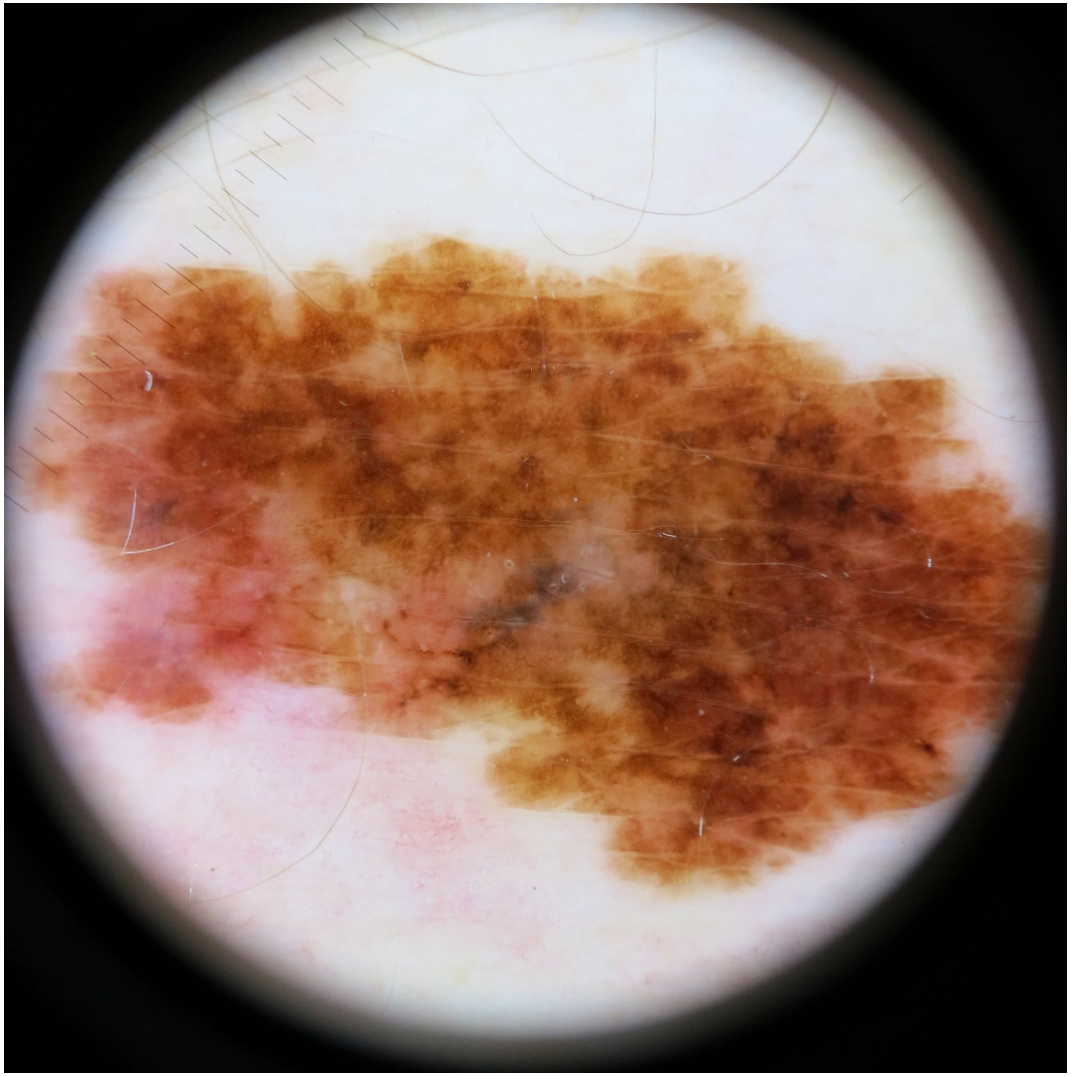

Location: Upper extremity

Invasive) Breslow interval: 0.1-0.5 mm

Case number 68

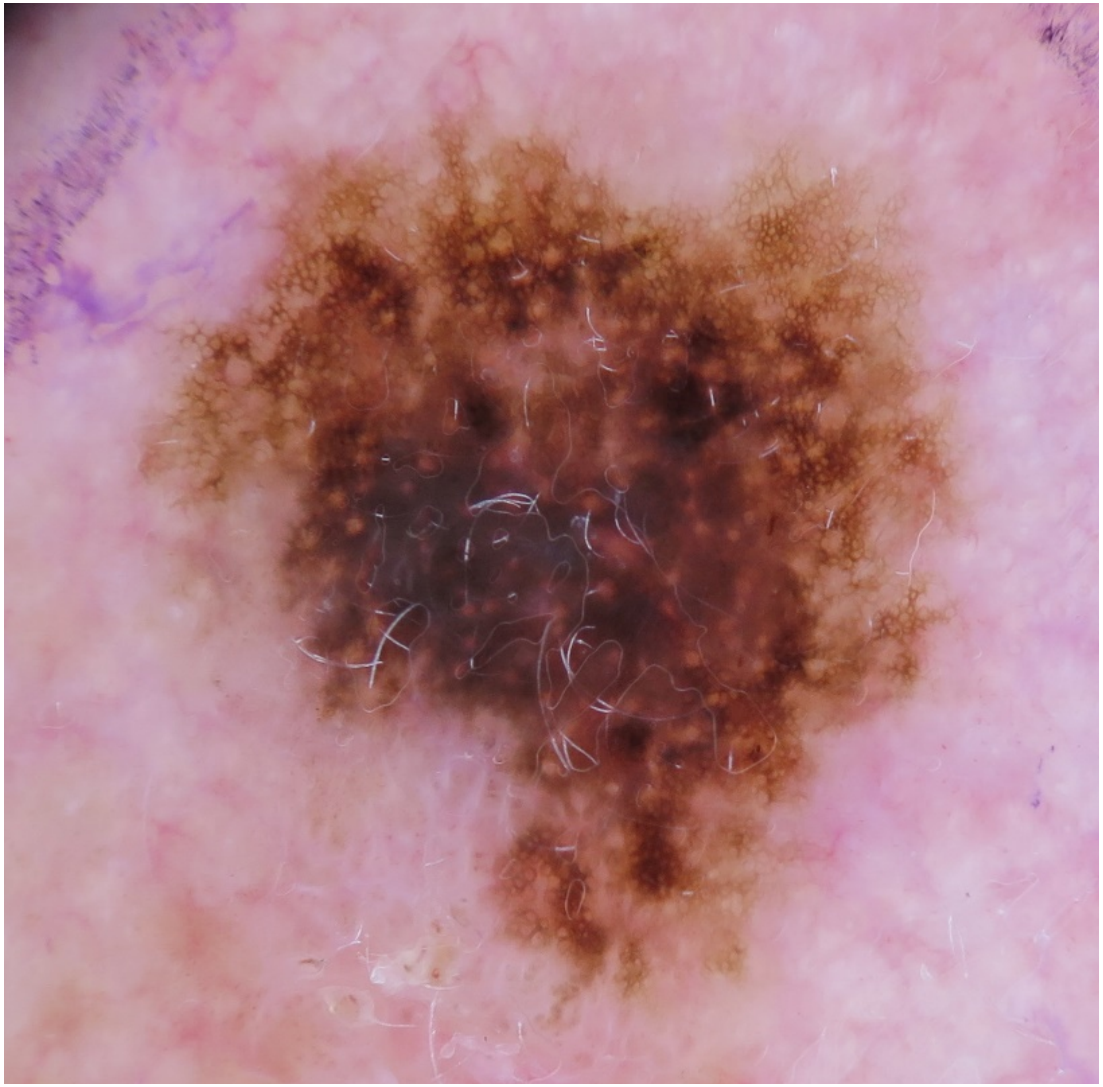

Location: Neck

In situ melanoma

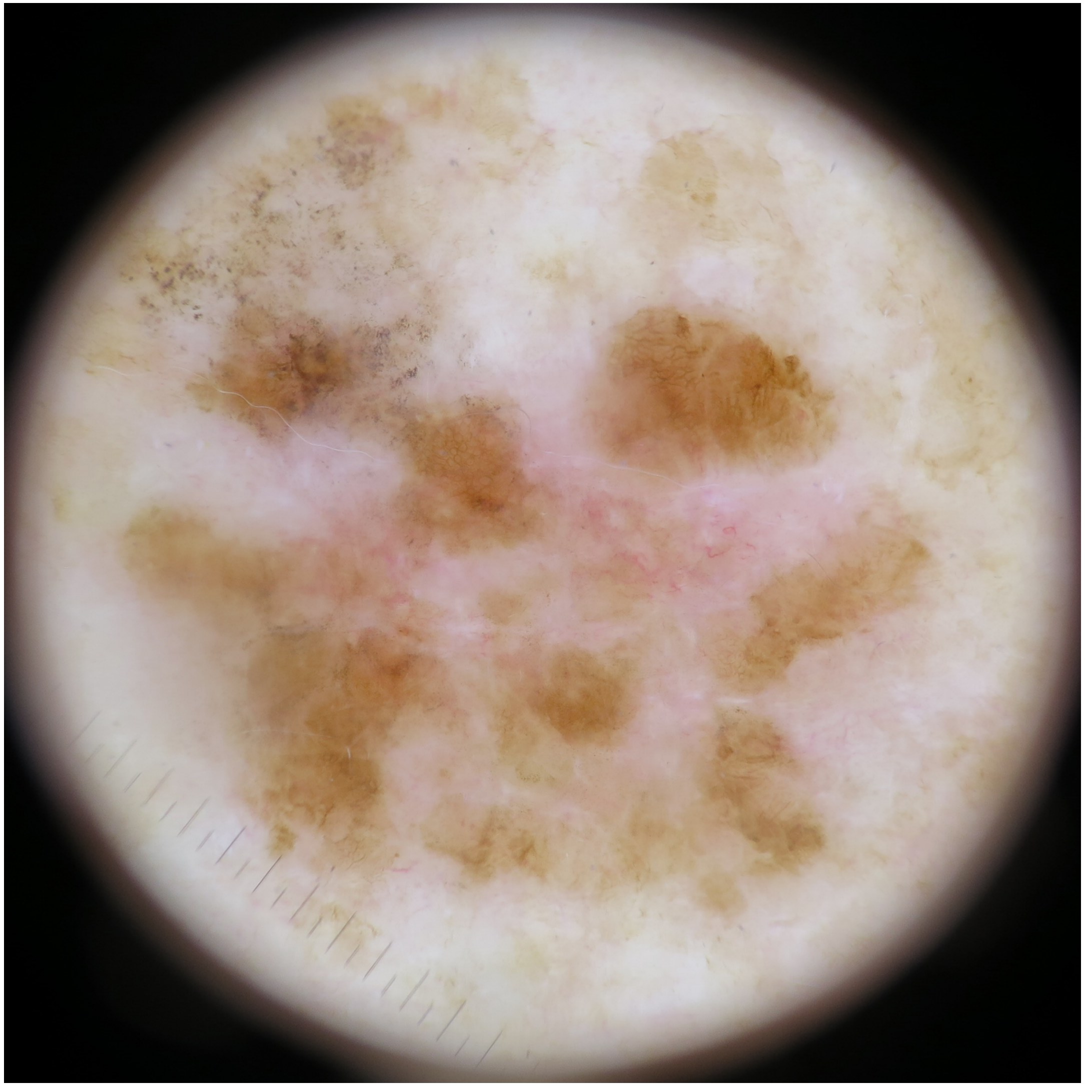

Location: Trunk

In situ melanoma

Case number 70

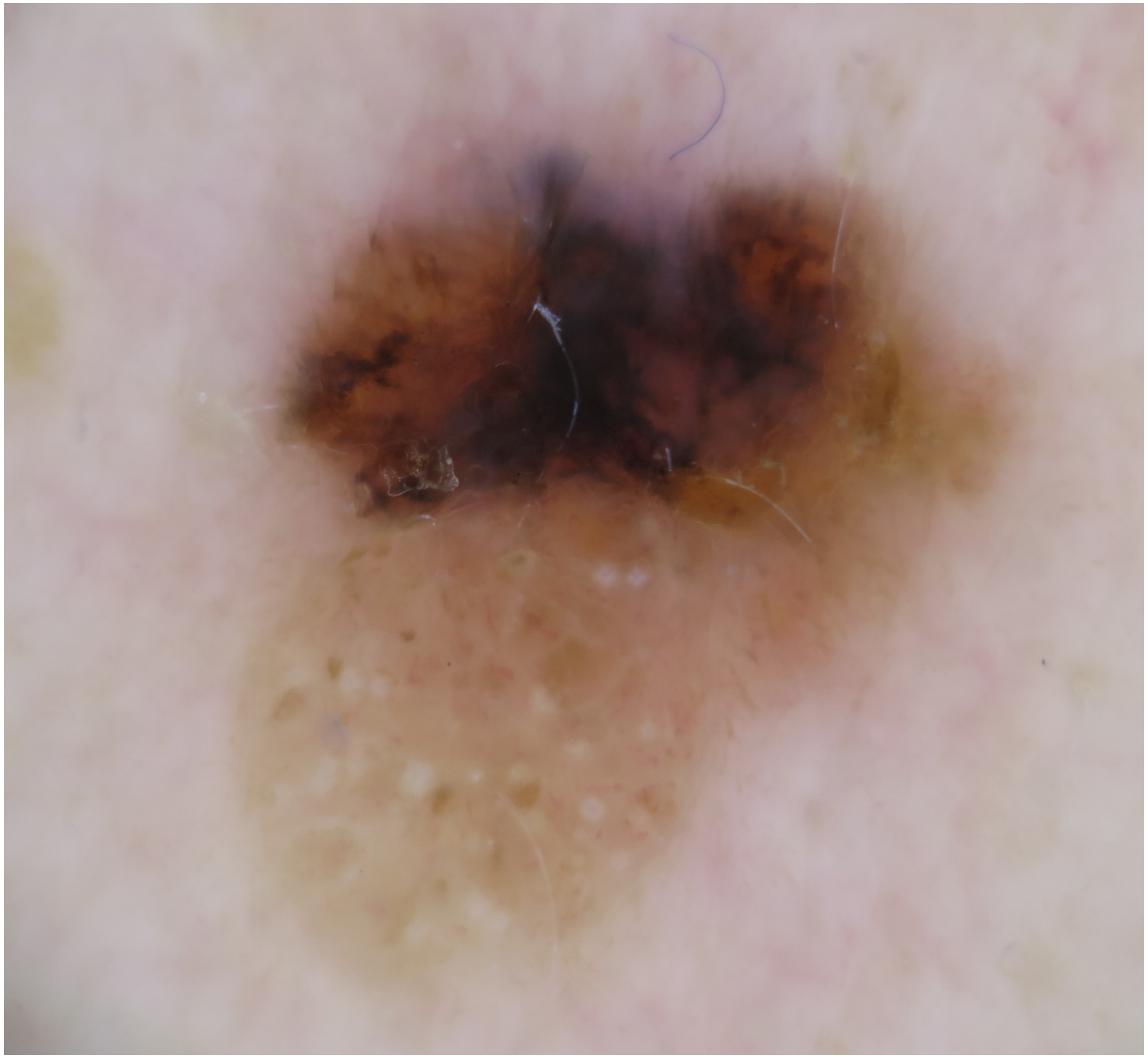

Location: Trunk

In situ melanoma

Case number 71

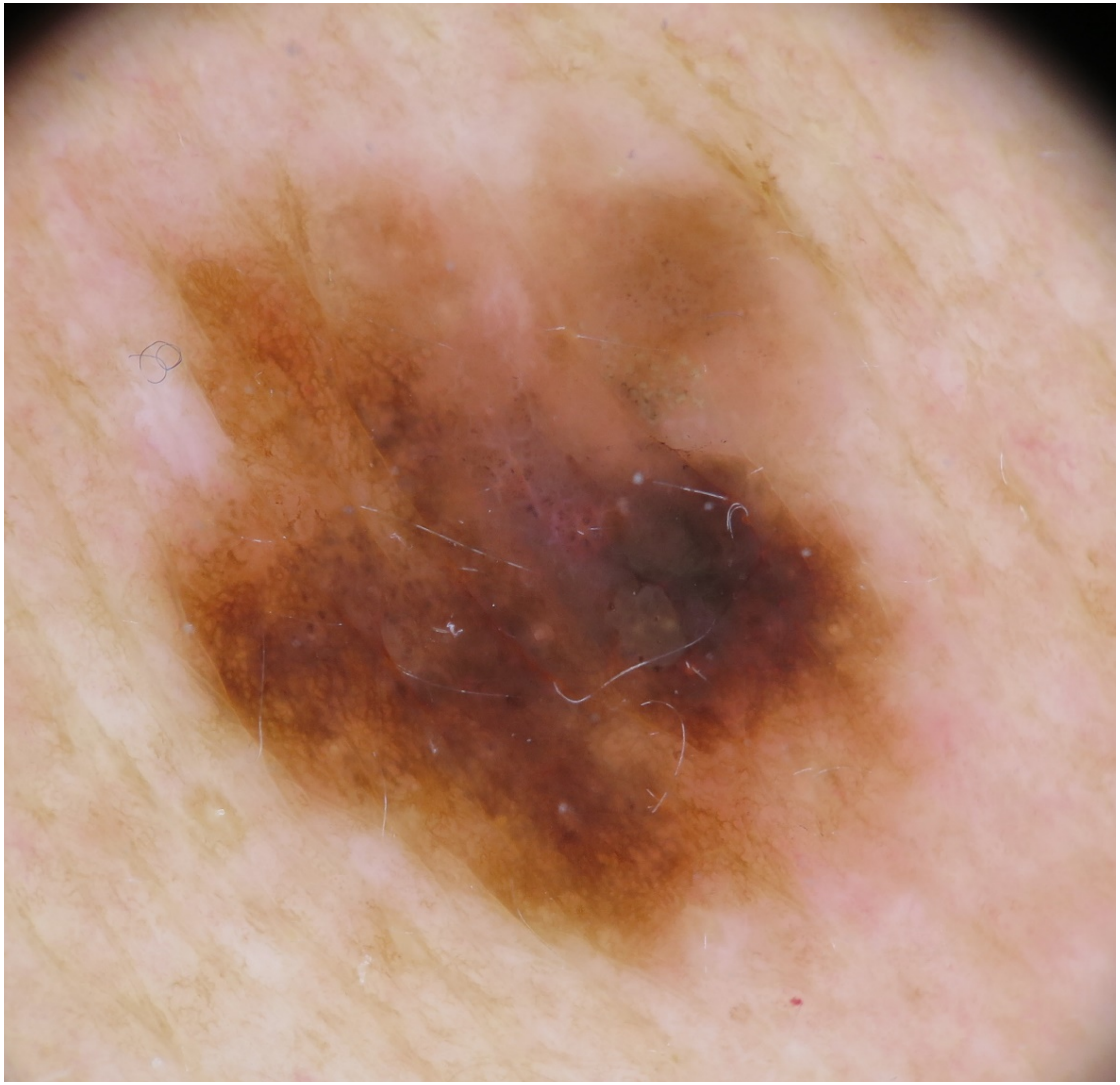

Location: Trunk

In situ melanoma

Case number 72

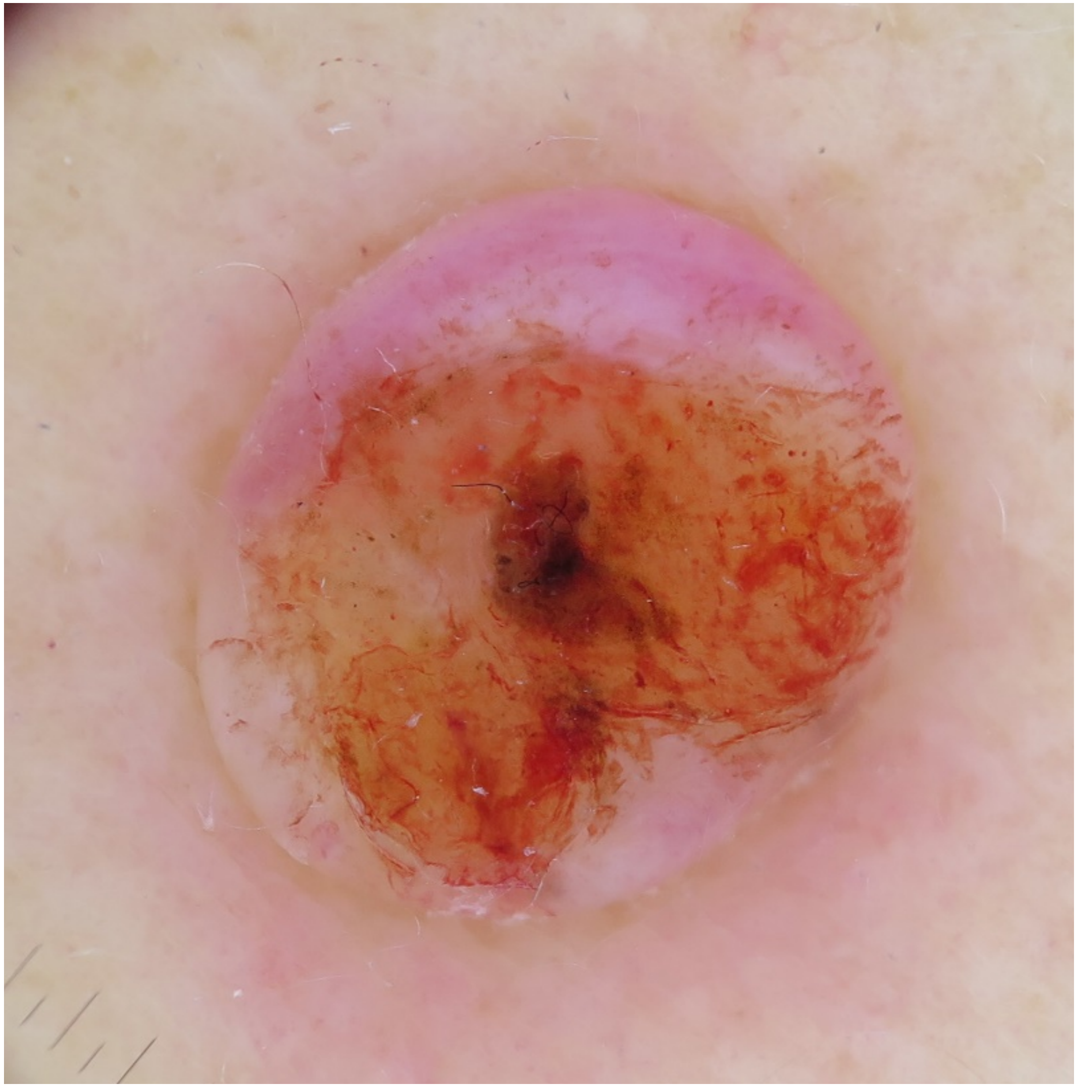

Location: Upper extremity

Invasive) Breslow interval: > 4mm

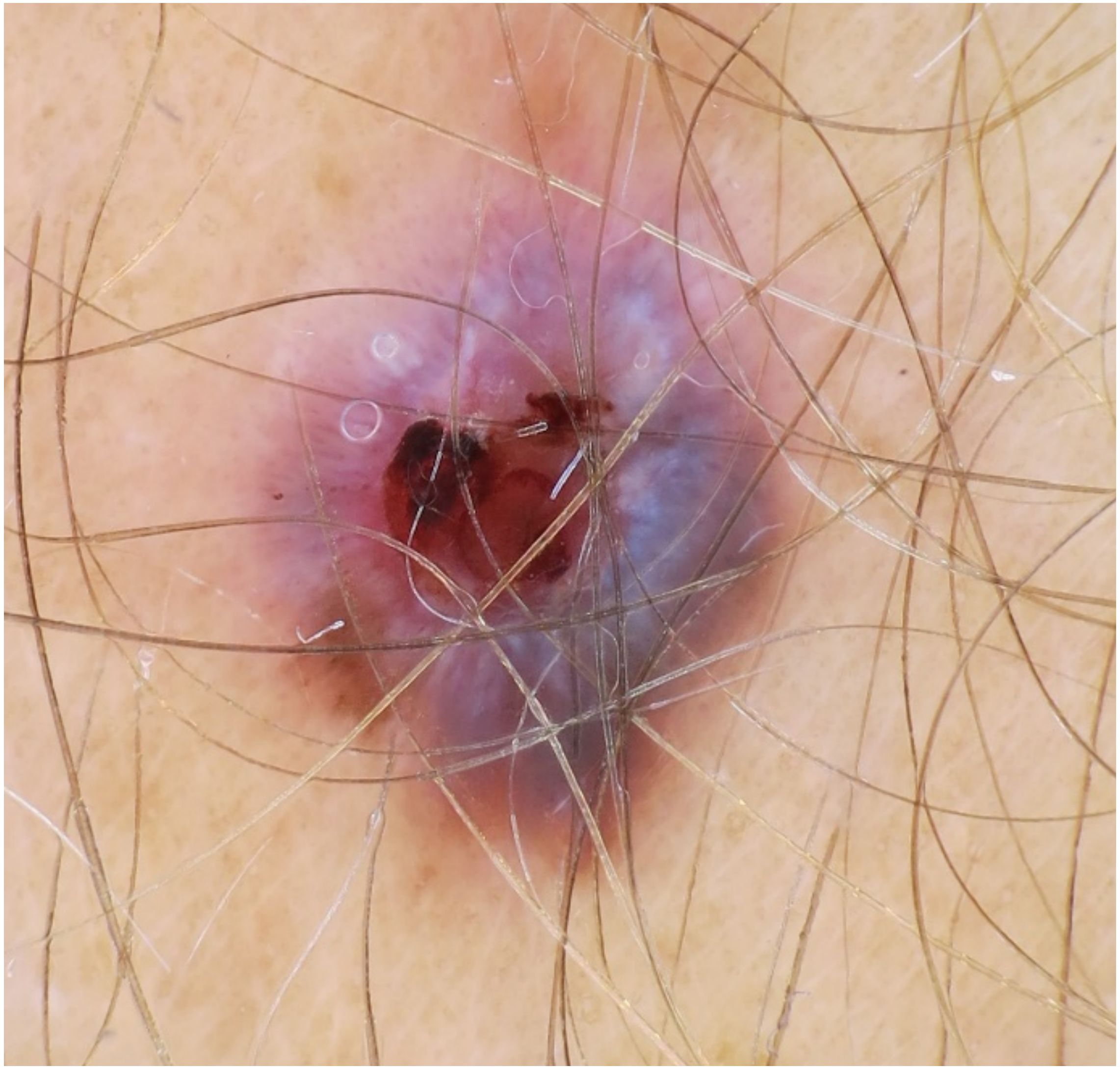

Location: Lower extremity

Invasive) Breslow interval: 0.9-1.0 mm

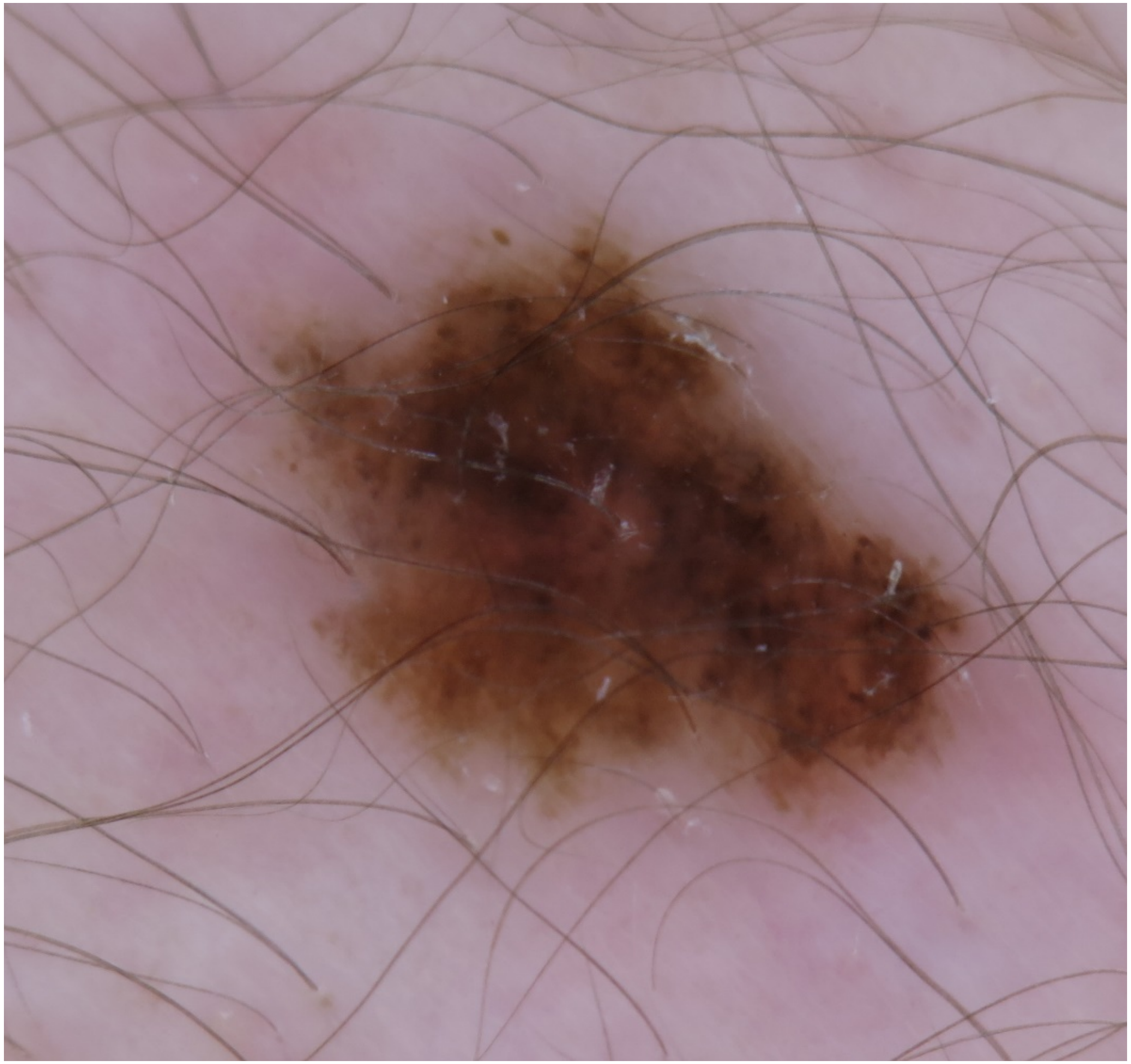

Location: Upper extremity

In situ melanoma

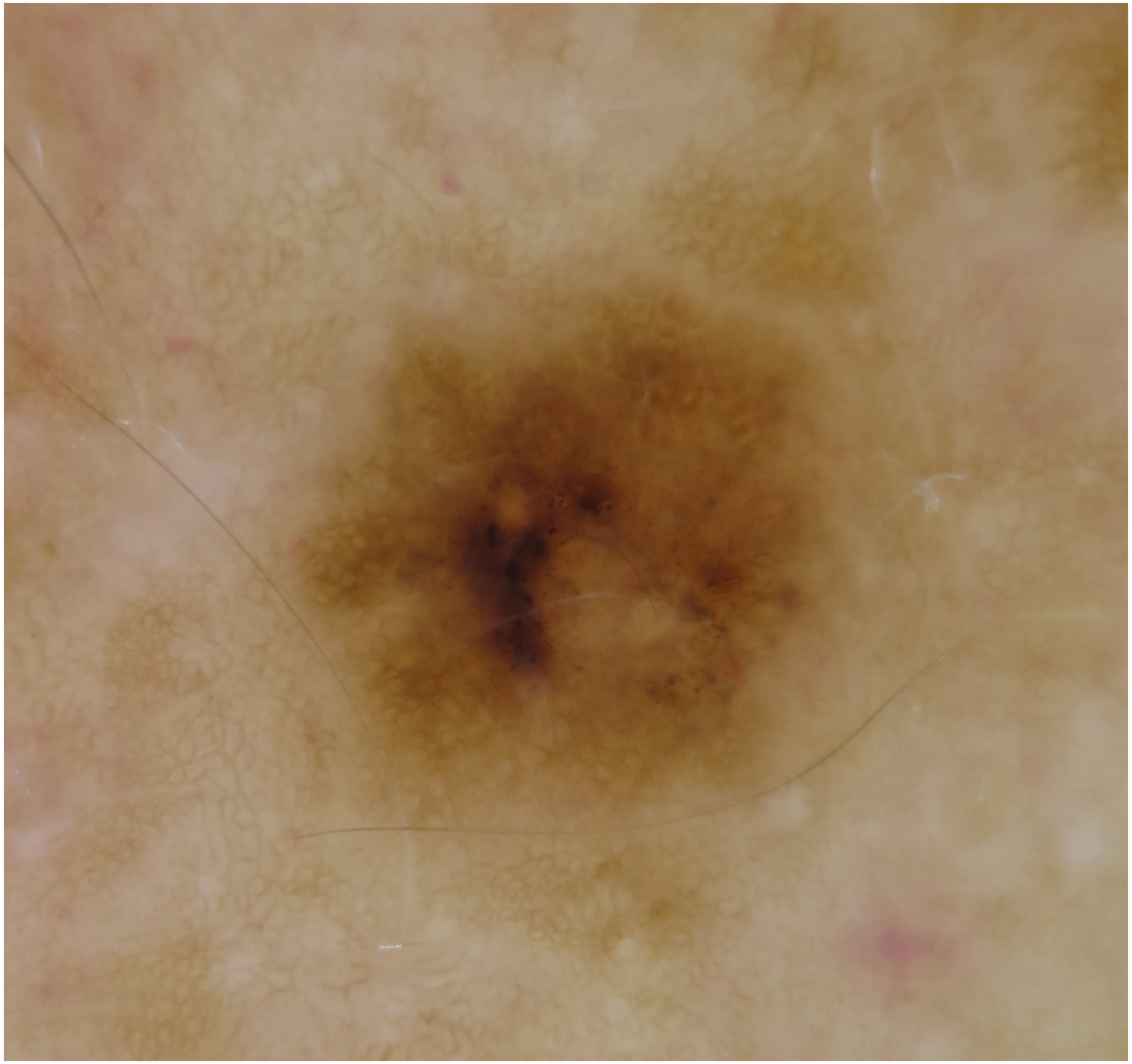

Location: Upper extremity

Invasive) Breslow interval: 0.6-0.8 mm

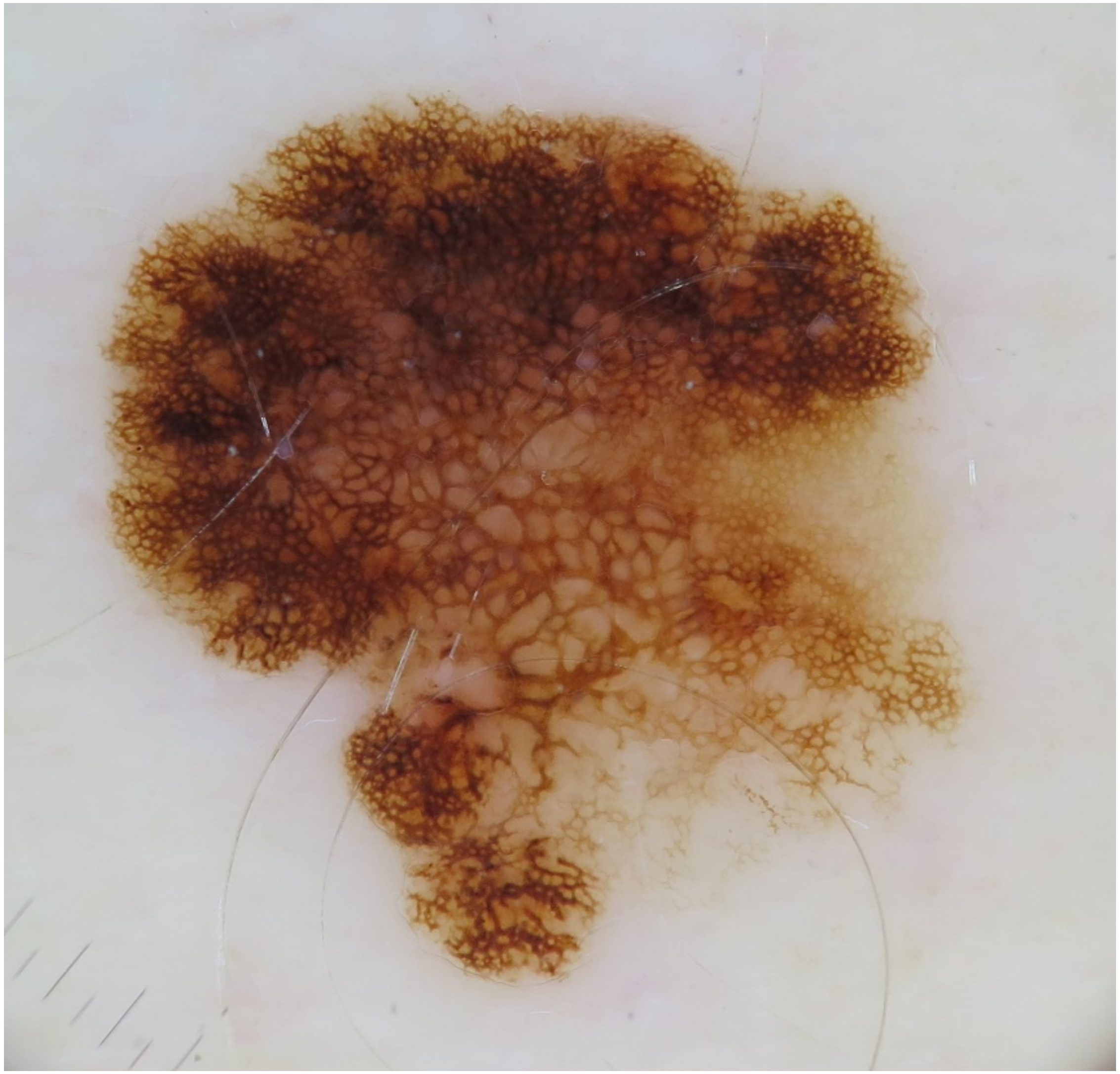

Location: Trunk

In situ melanoma

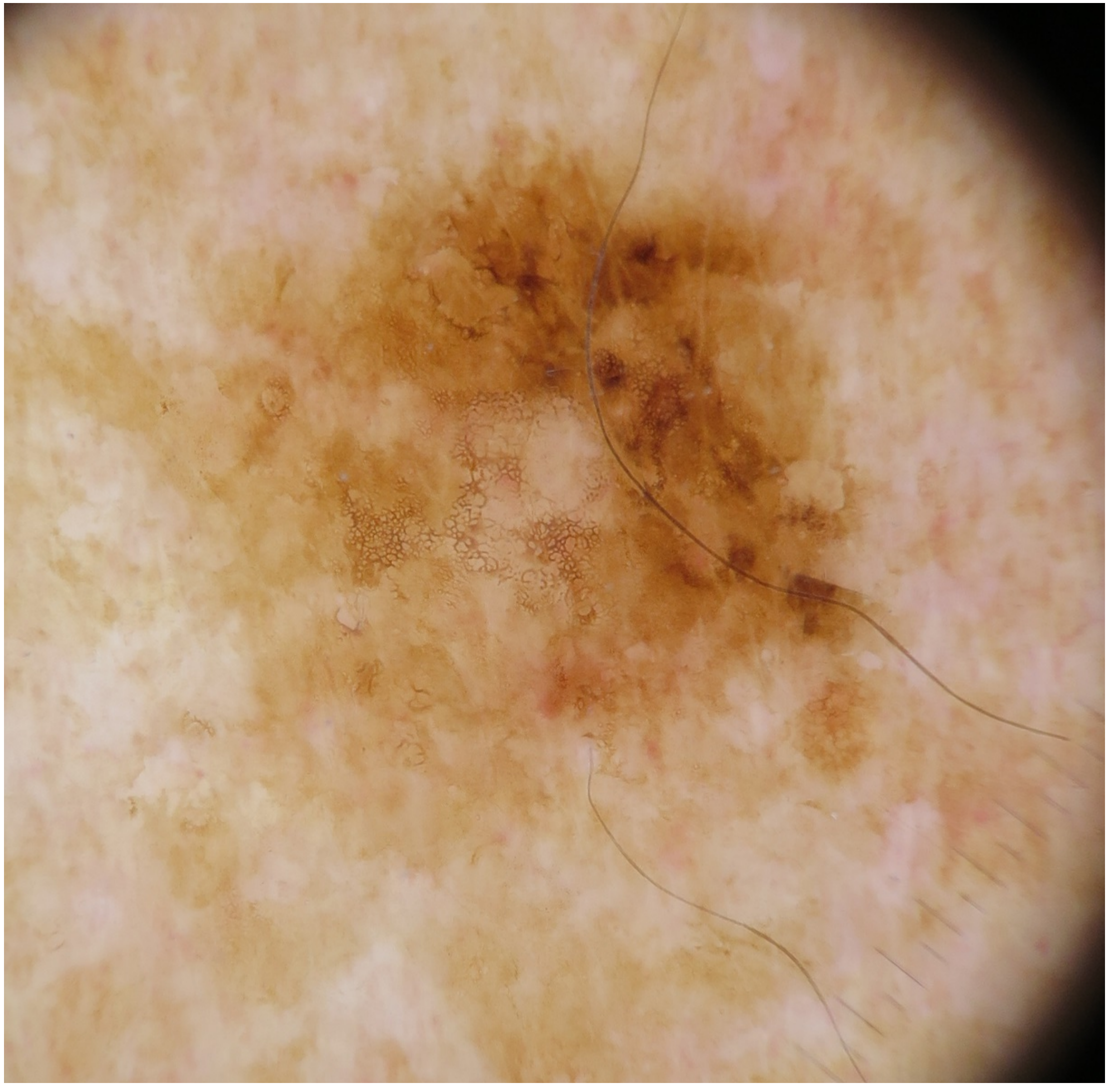

Location: Lower extremity

In situ melanoma

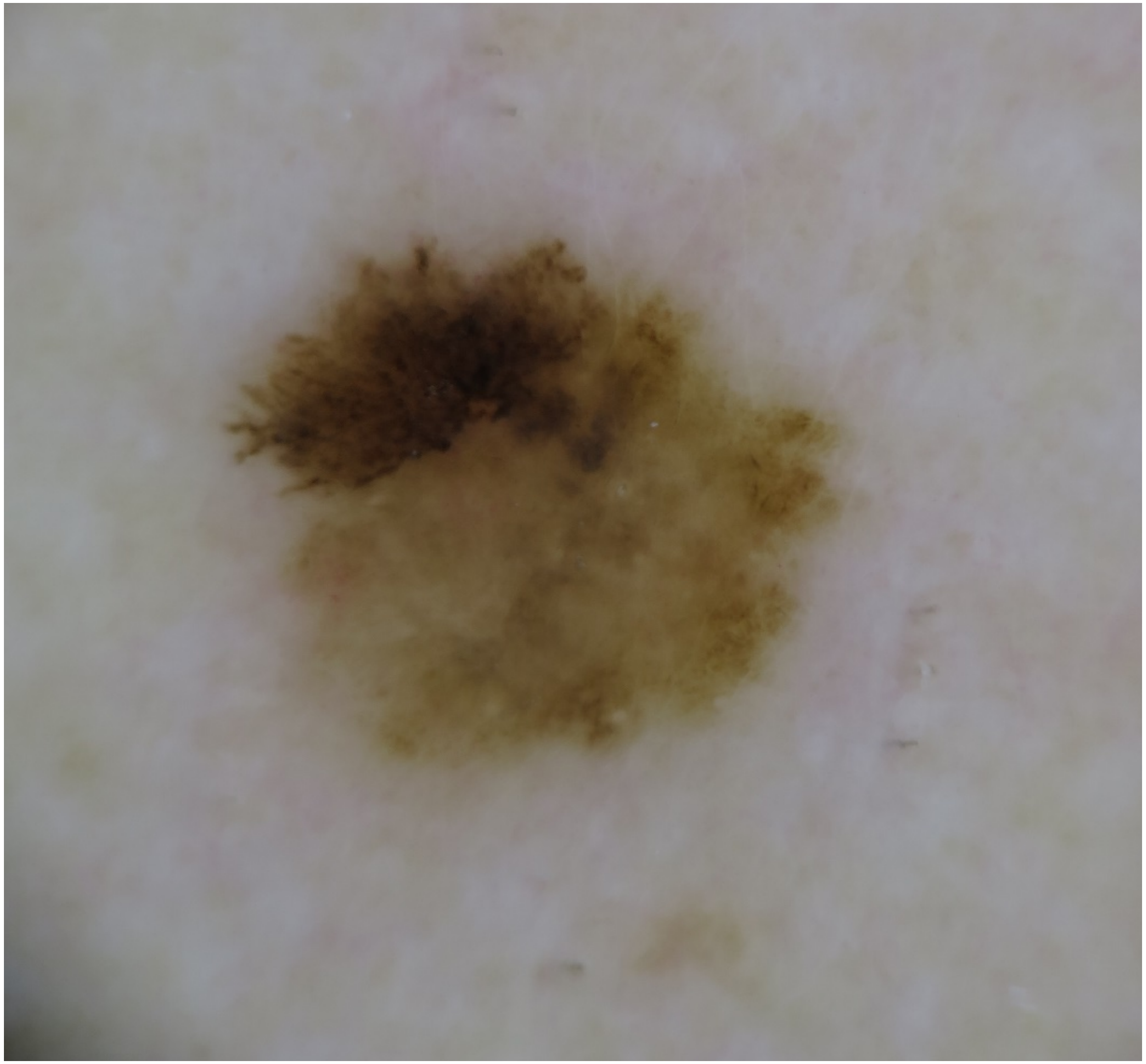

Location: Lower extremity

Invasive) Breslow interval: 0.1-0.5 mm

Case number 79

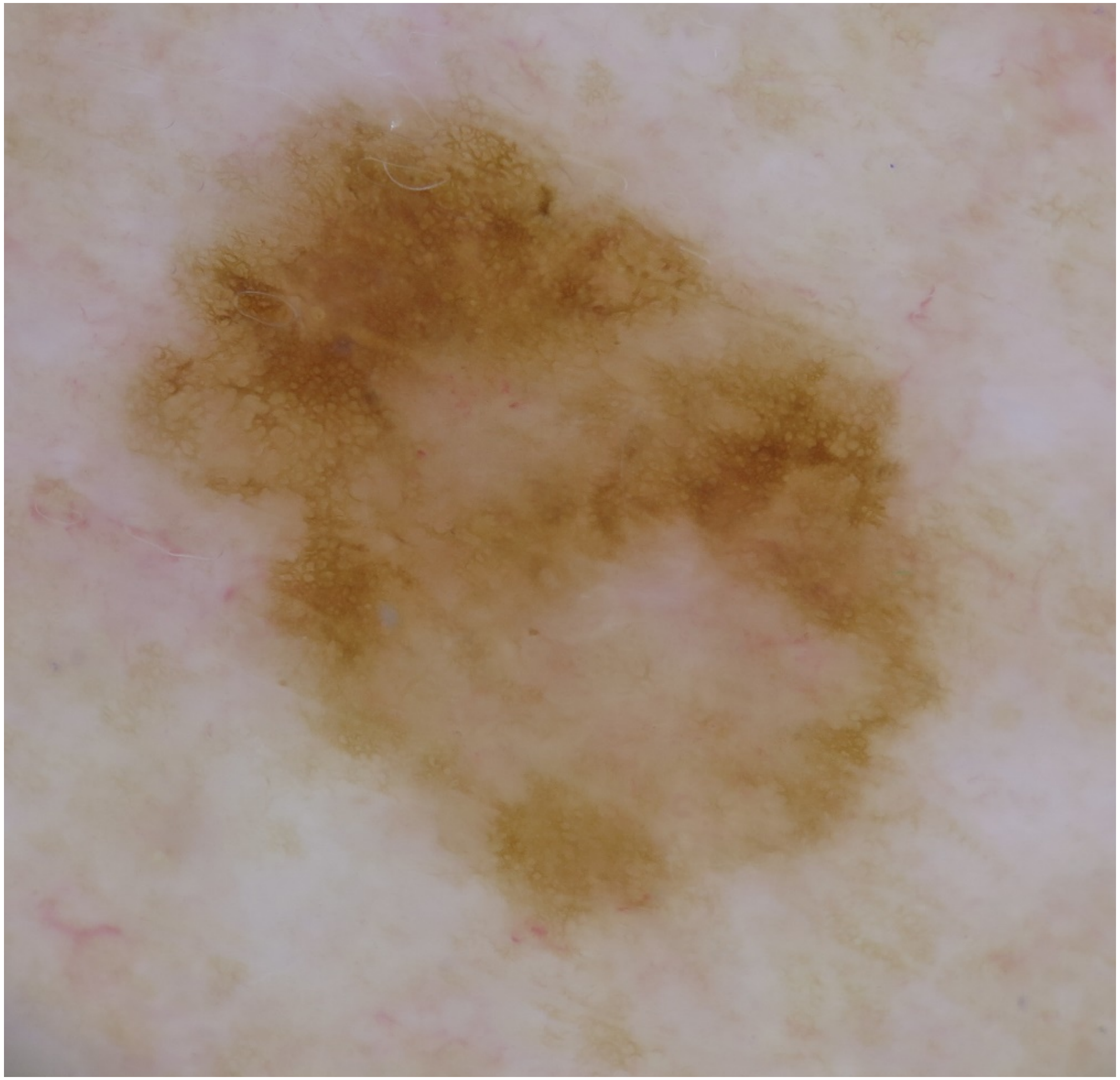

Location: Trunk

In situ melanoma

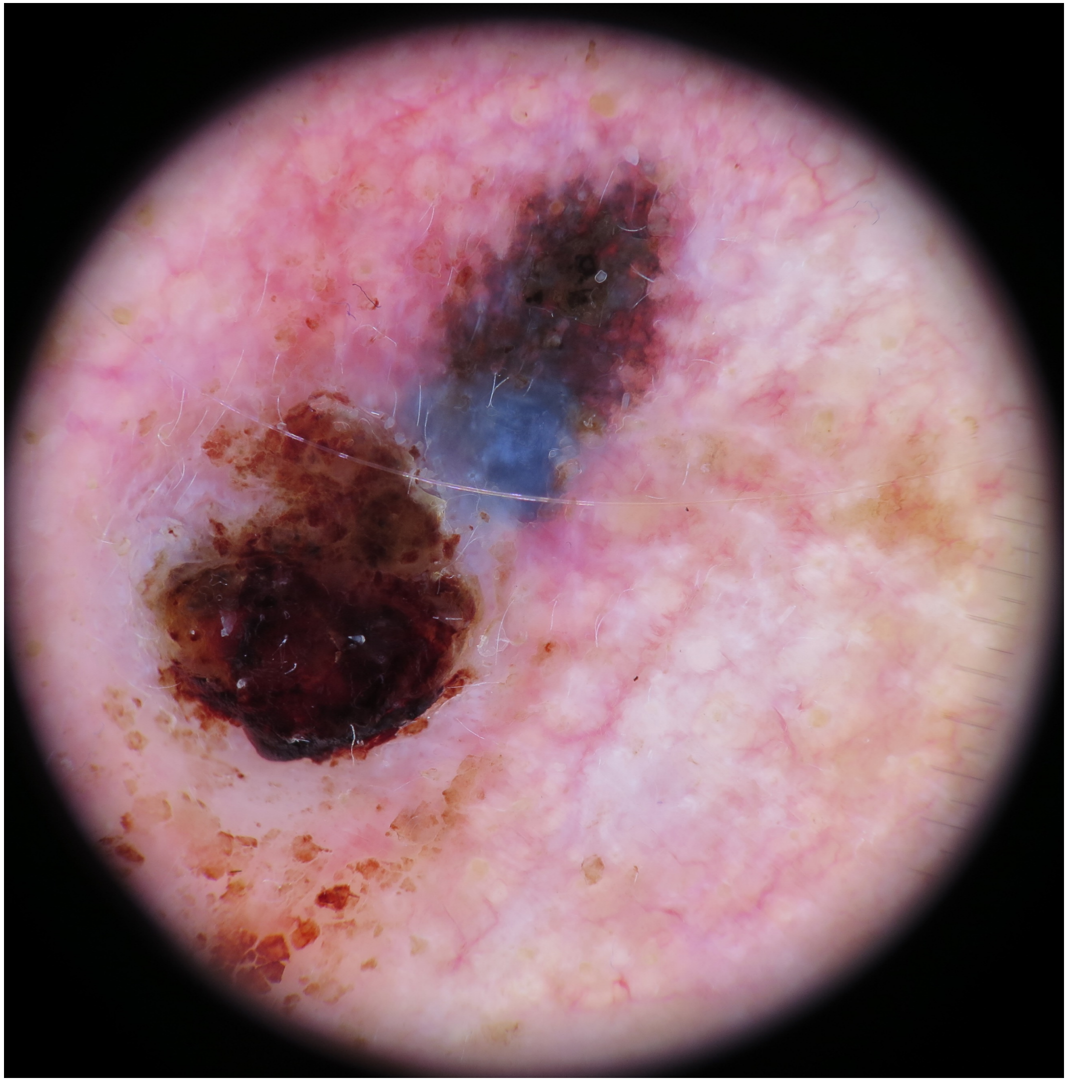

Location: Neck

Invasive) Breslow interval: 2.1-4.0 mm

Case number 81

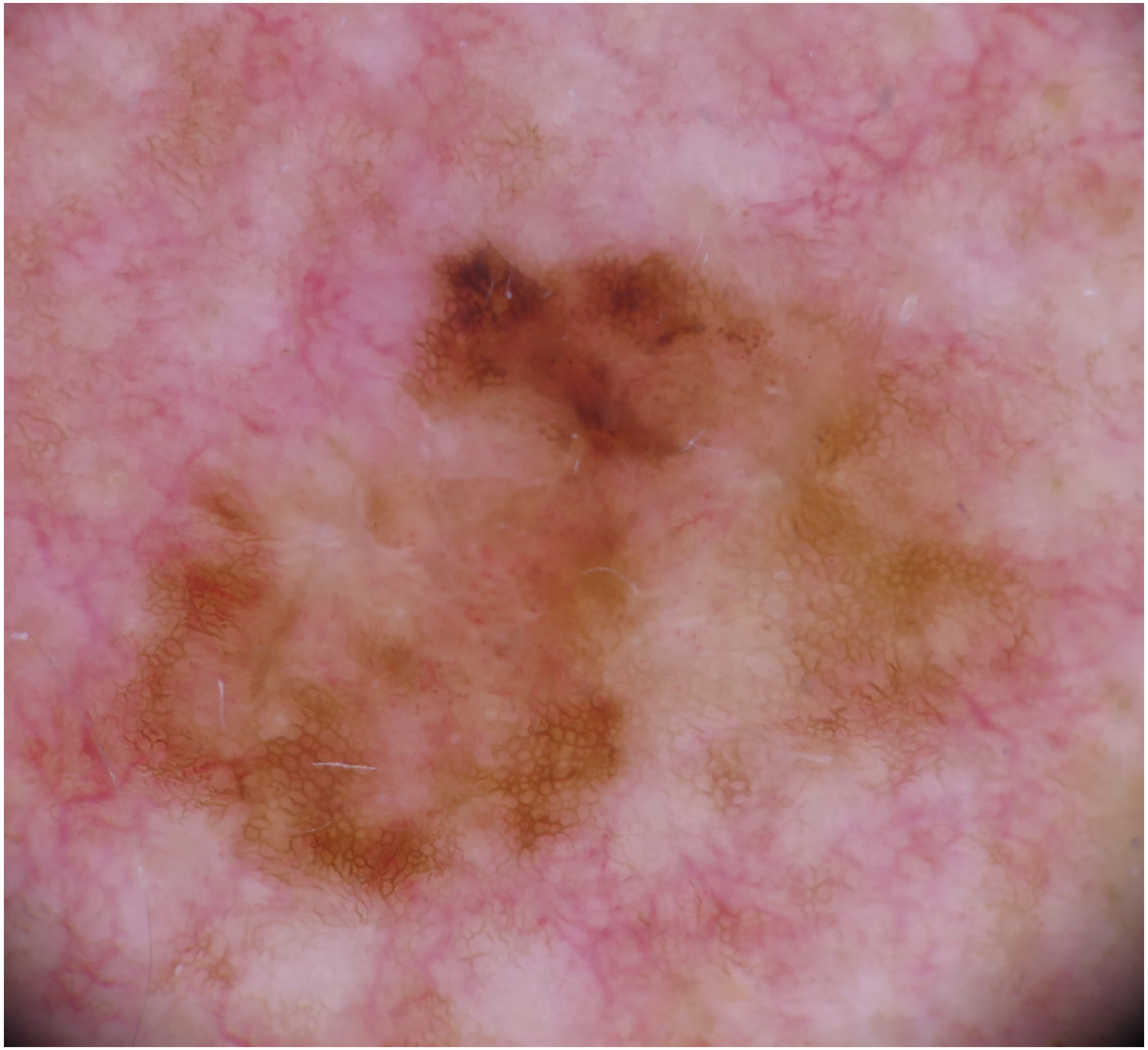

Location: Trunk

In situ melanoma

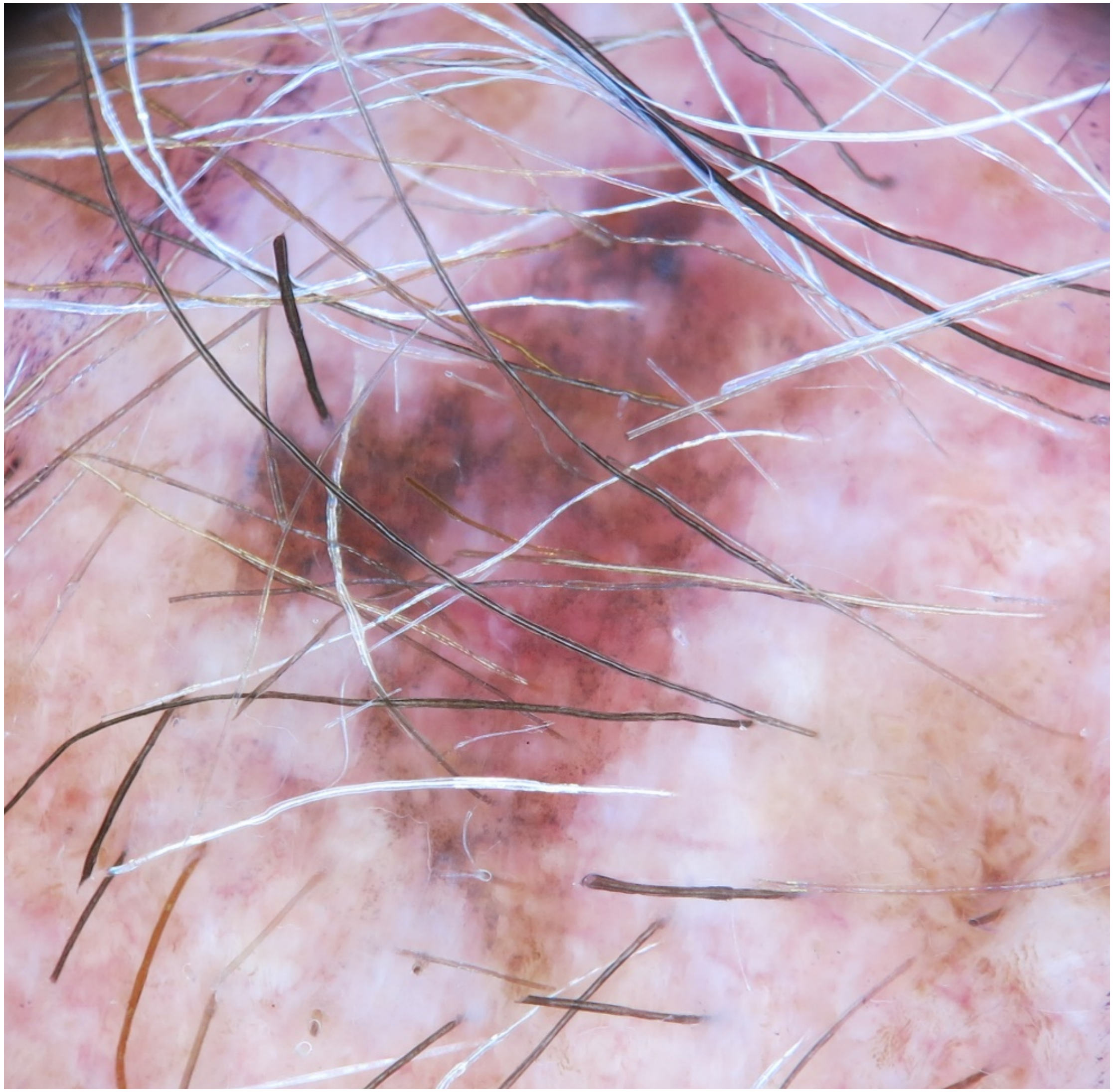

Location: Face

In situ melanoma

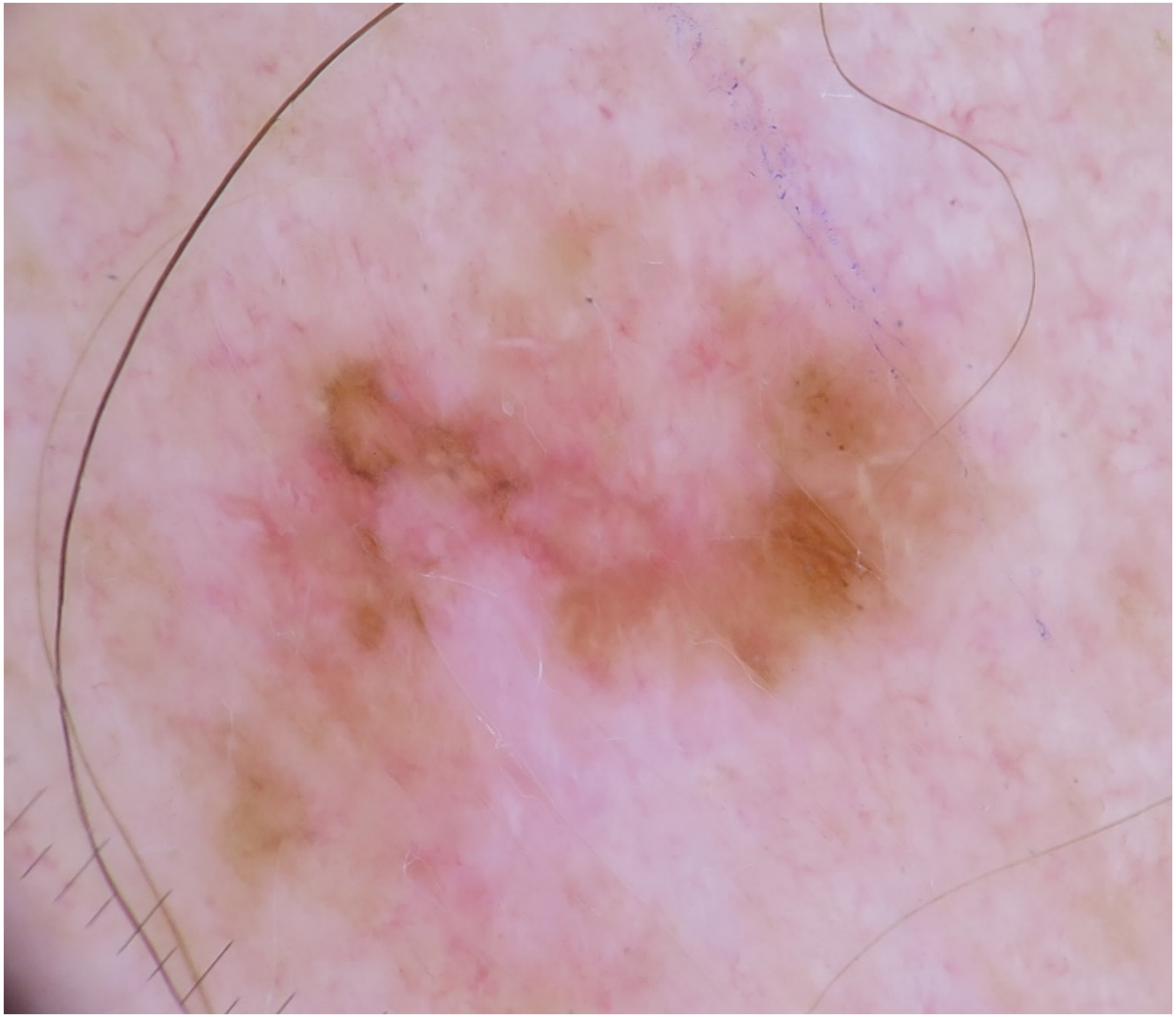

Location: Upper extremity

In situ melanoma

Case number 84

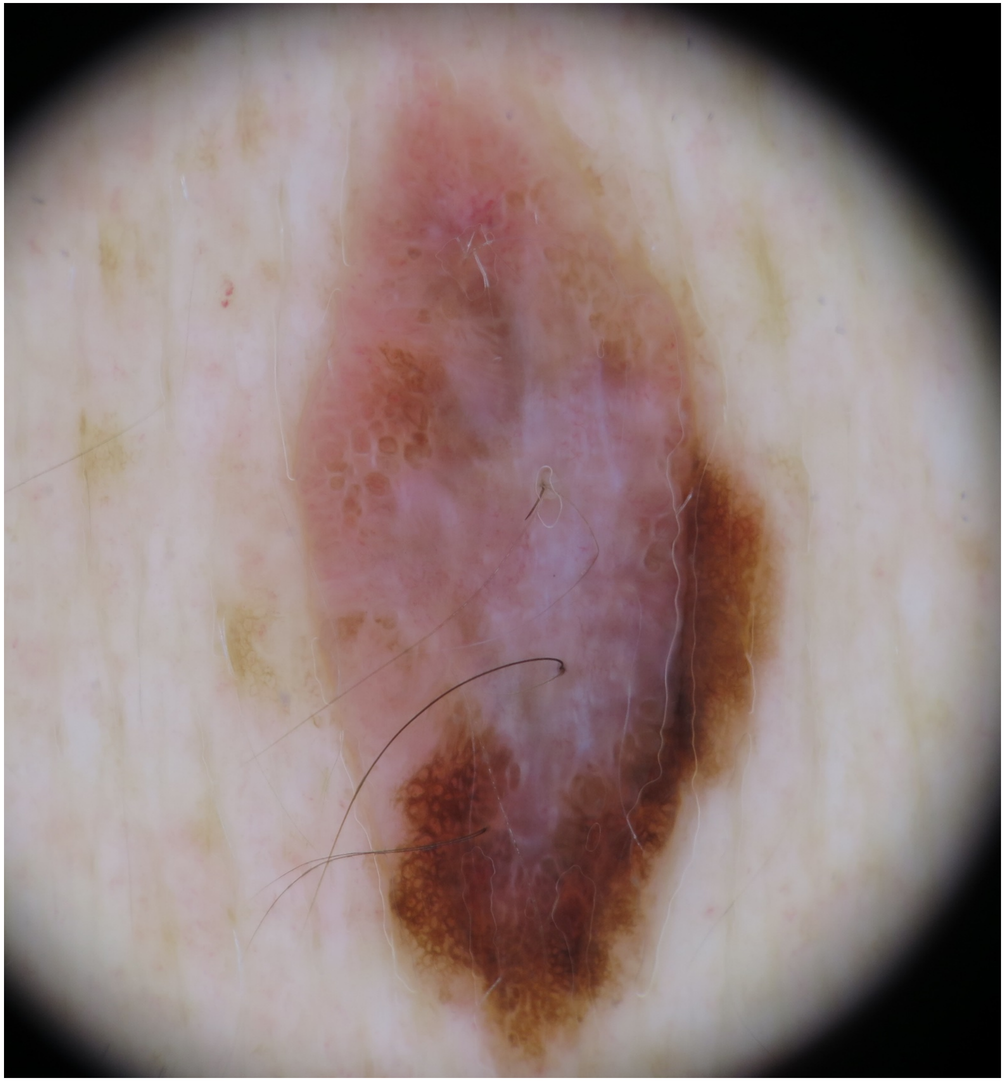

Location: Trunk

Invasive) Breslow interval: 0.1-0.5 mm

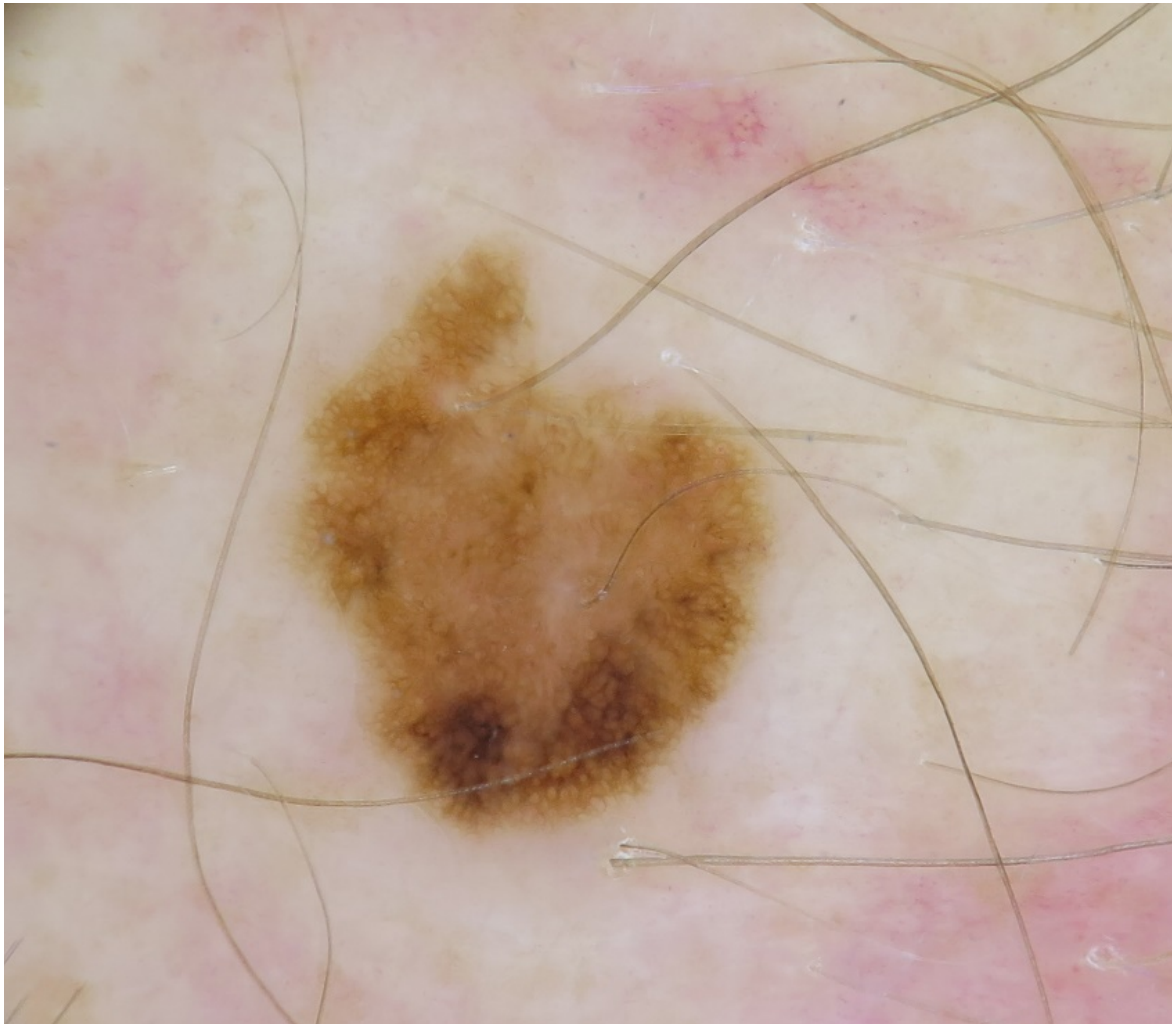

Location: Lower extremity

In situ melanoma

Case number 86

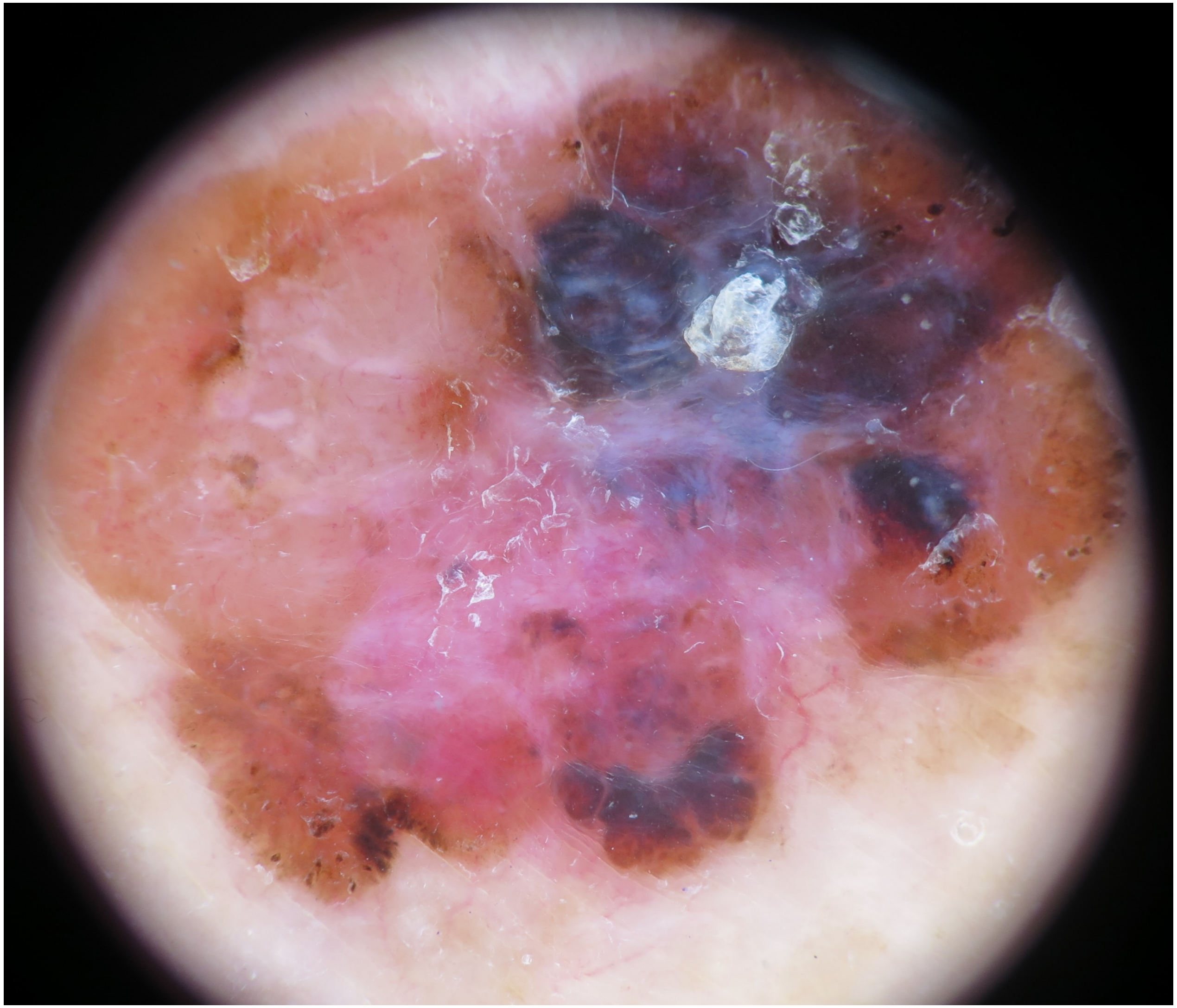

Location: Upper extremity

Invasive) Breslow interval: 2.1-4.0 mm

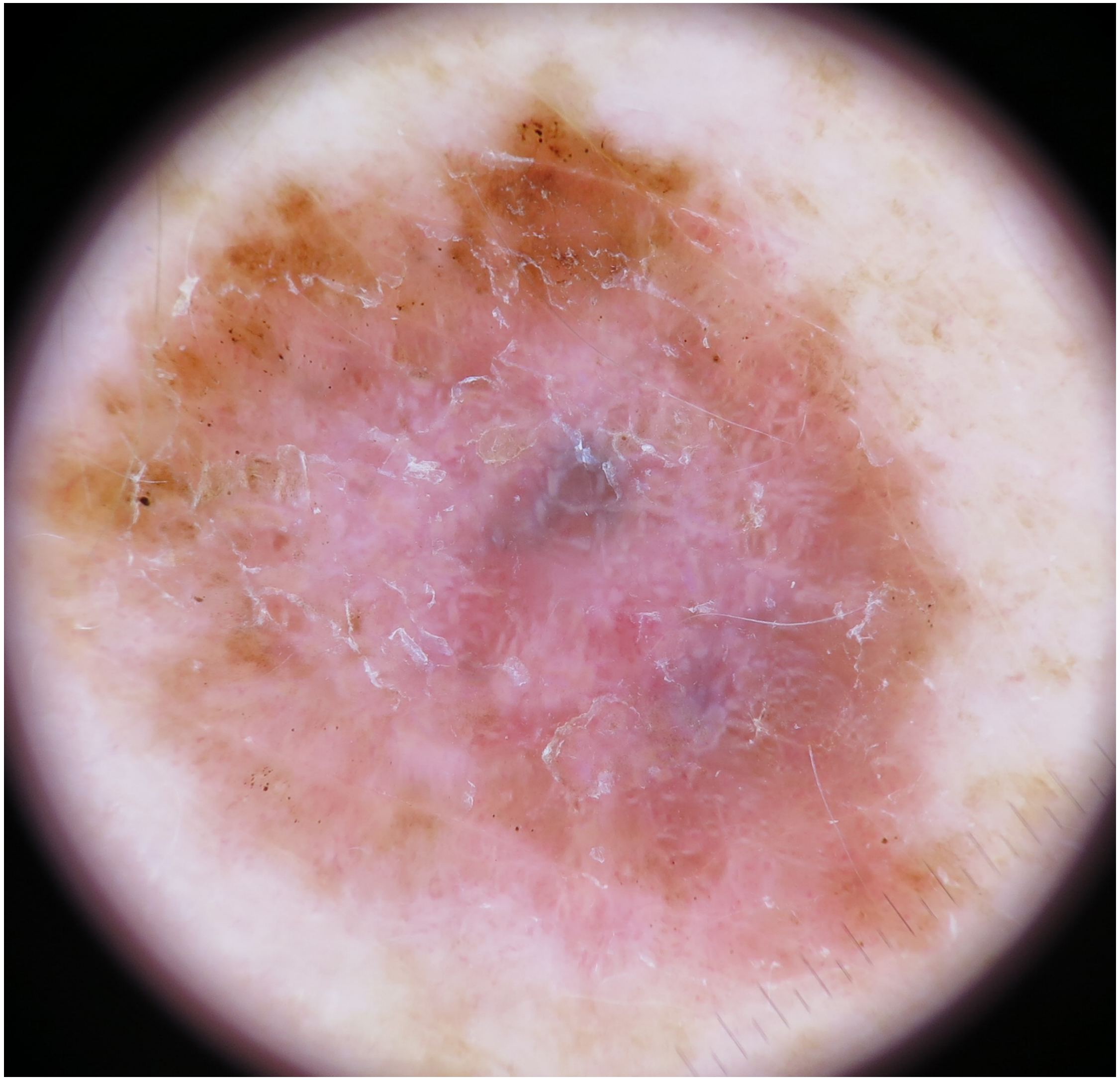

Location: Lower extremity

Invasive) Breslow interval: 1.1-2.0 mm

Case number 88

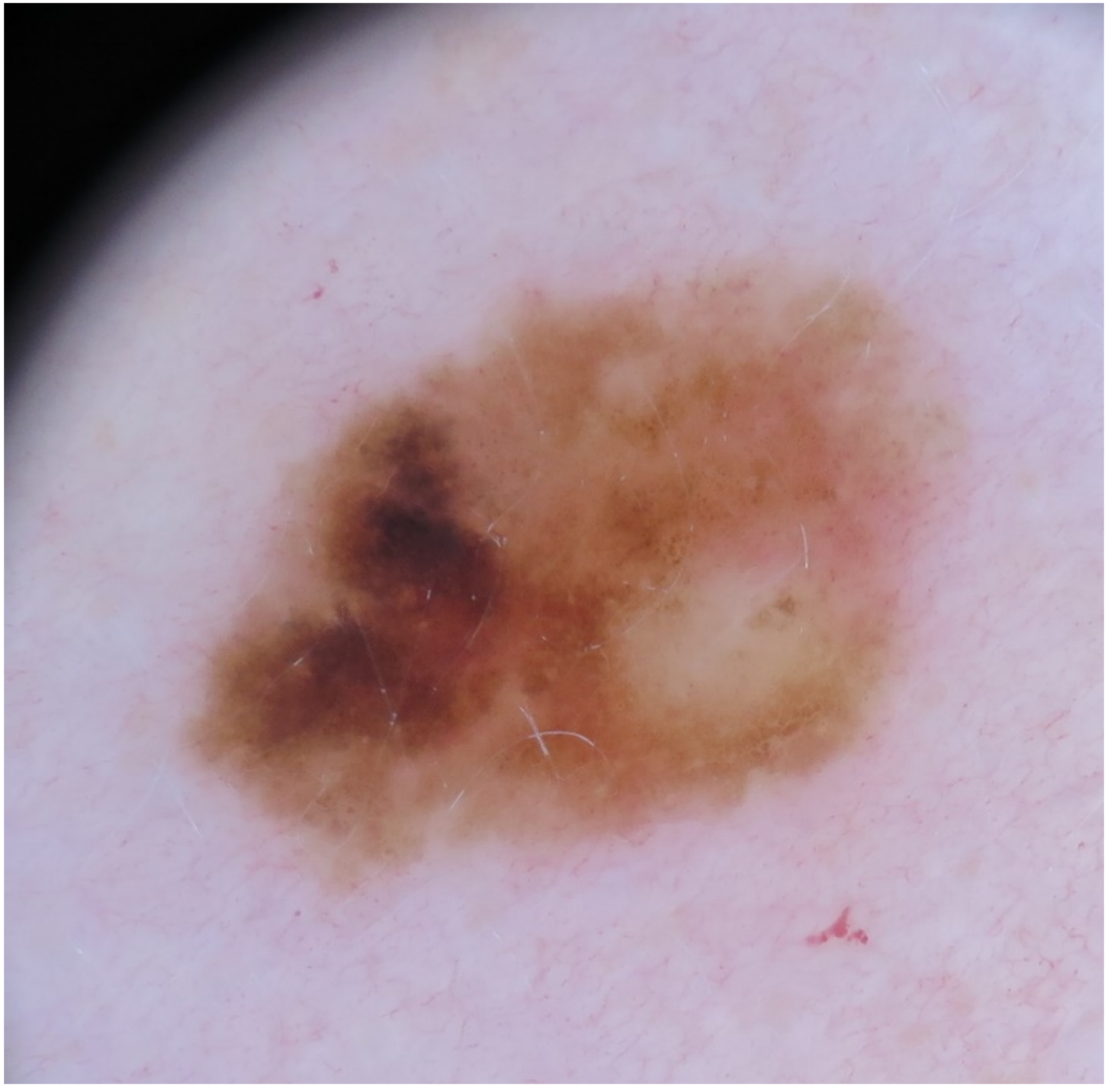

Location: Trunk

Invasive) Breslow interval: 0.6-0.8 mm

Case number 89

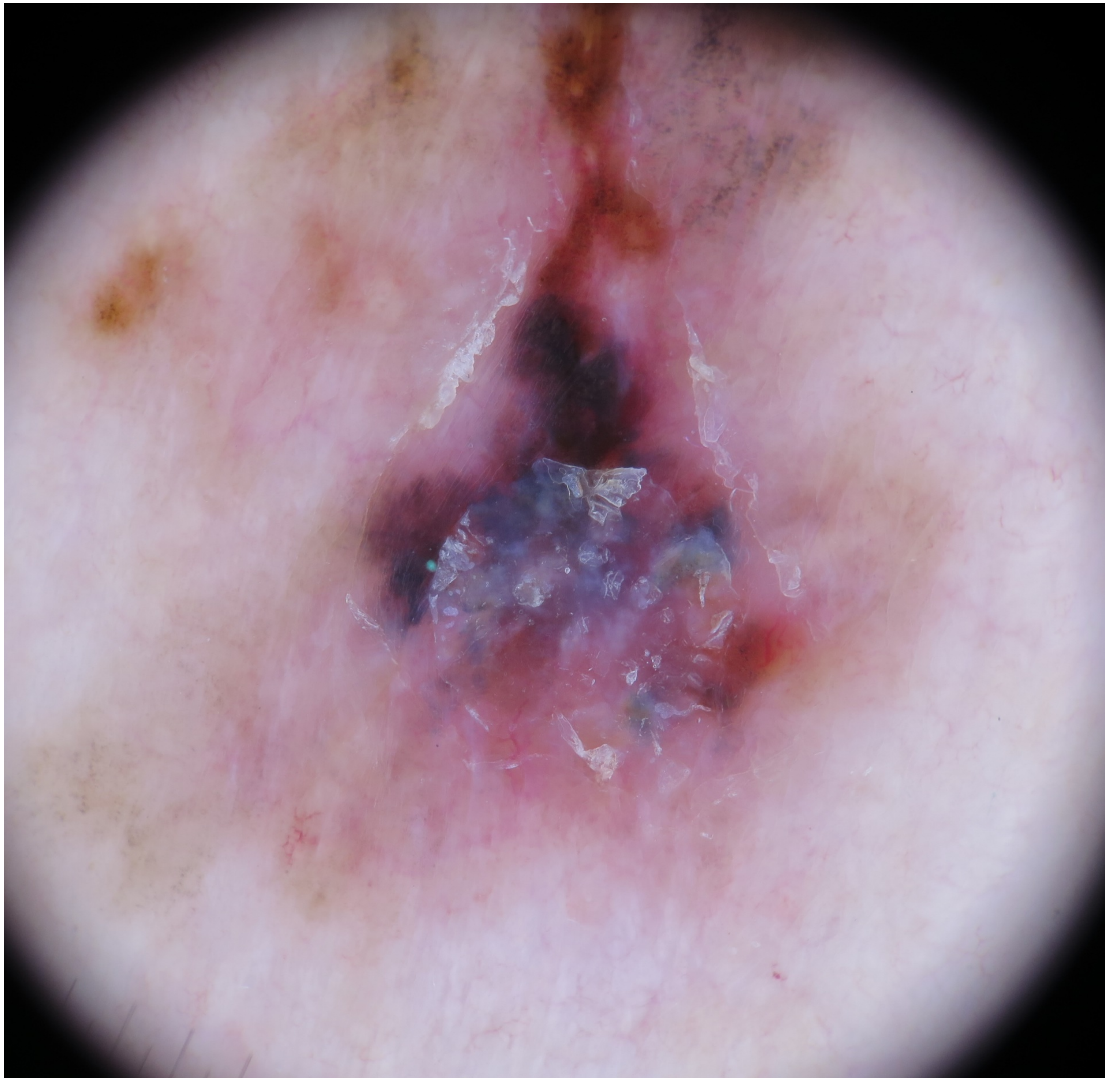

Location: Trunk

Invasive) Breslow interval: 1.1-2.0 mm

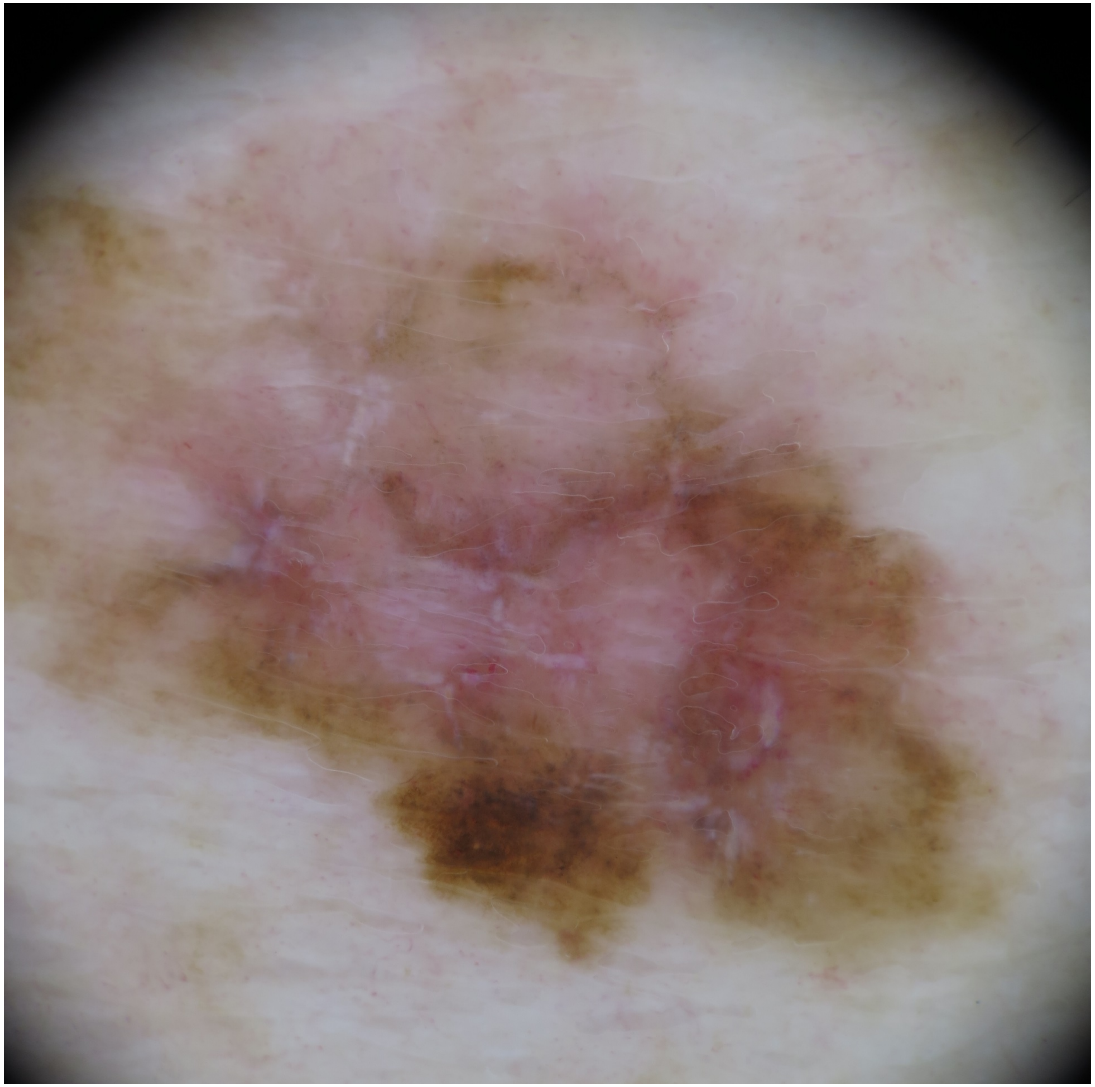

Location: Lower extremity

Invasive) Breslow interval: 0.9-1.0 mm

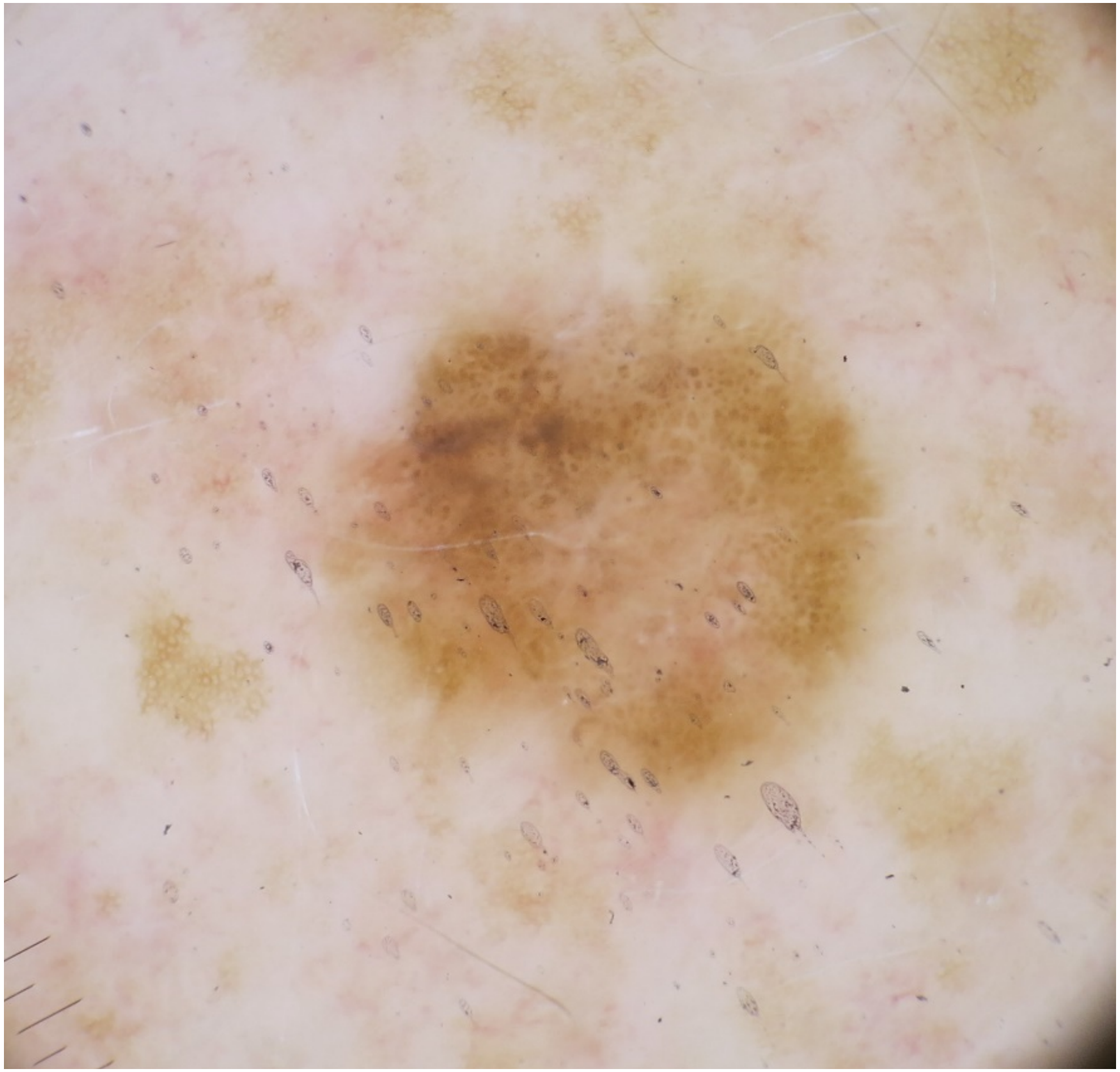

Location: Trunk

In situ melanoma

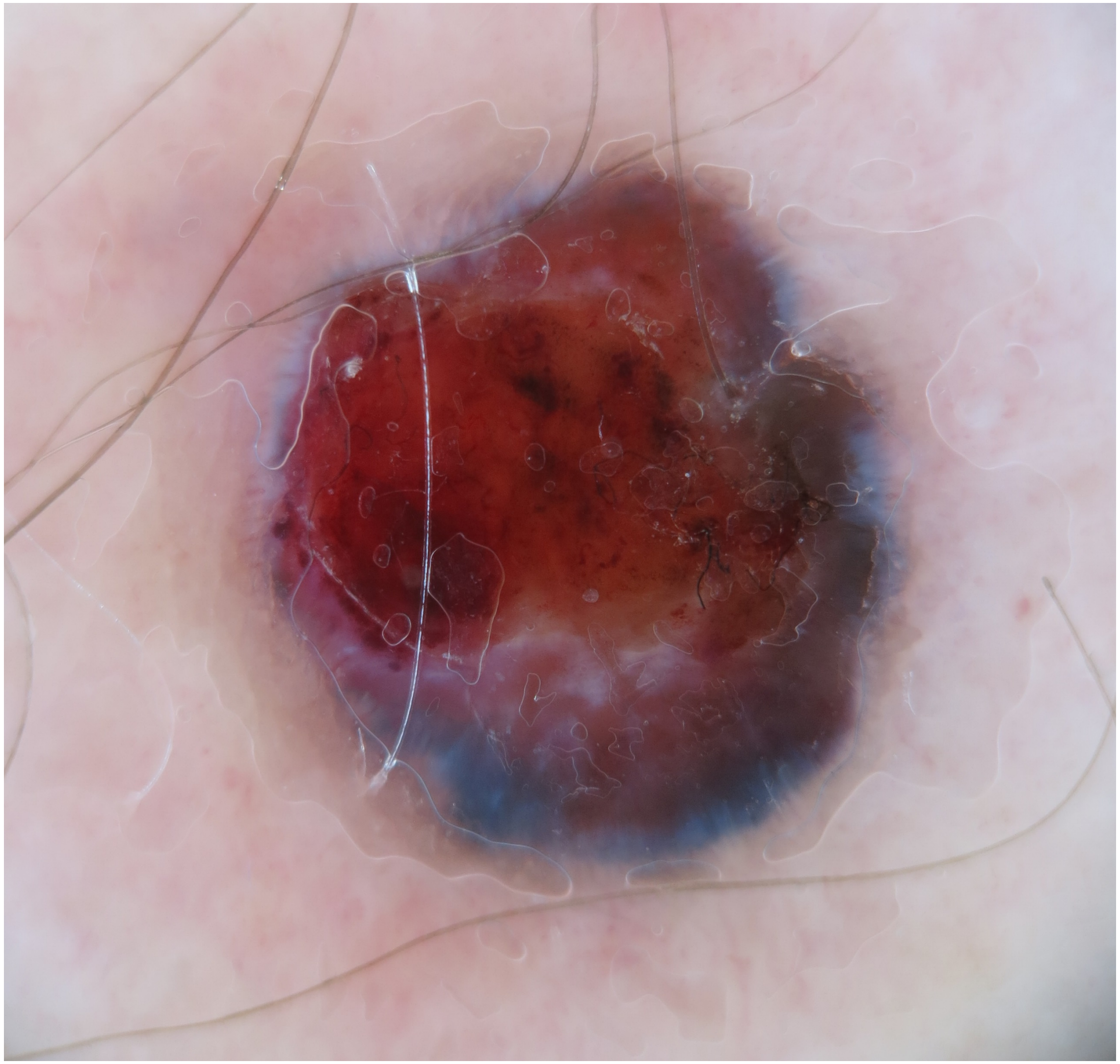

Location: Lower extremity

Invasive) Breslow interval: 1.1-2.0 mm

Case number 93

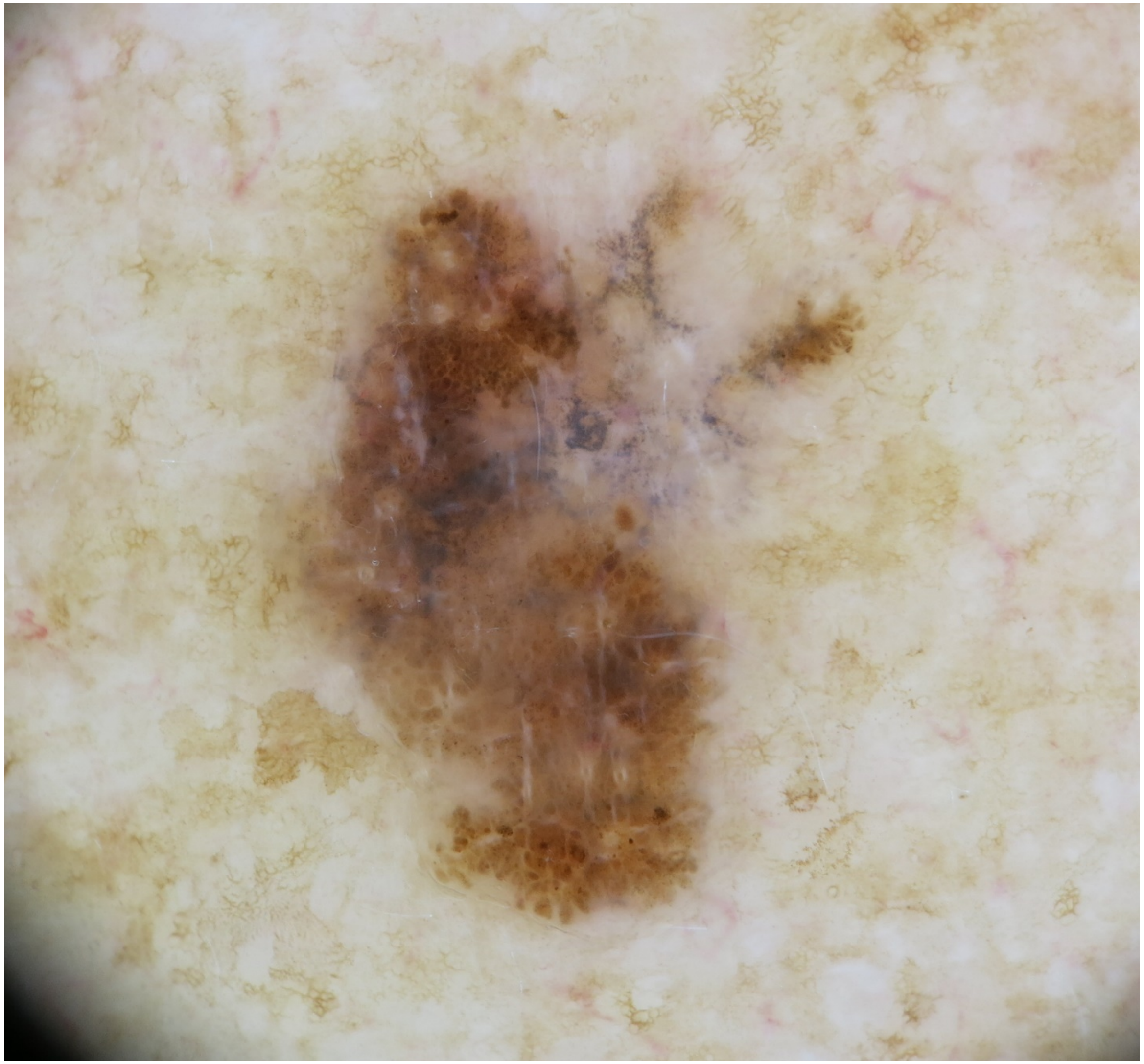

Location: Upper extremity

Invasive) Breslow interval: 0.1-0.5 mm

Case number 94

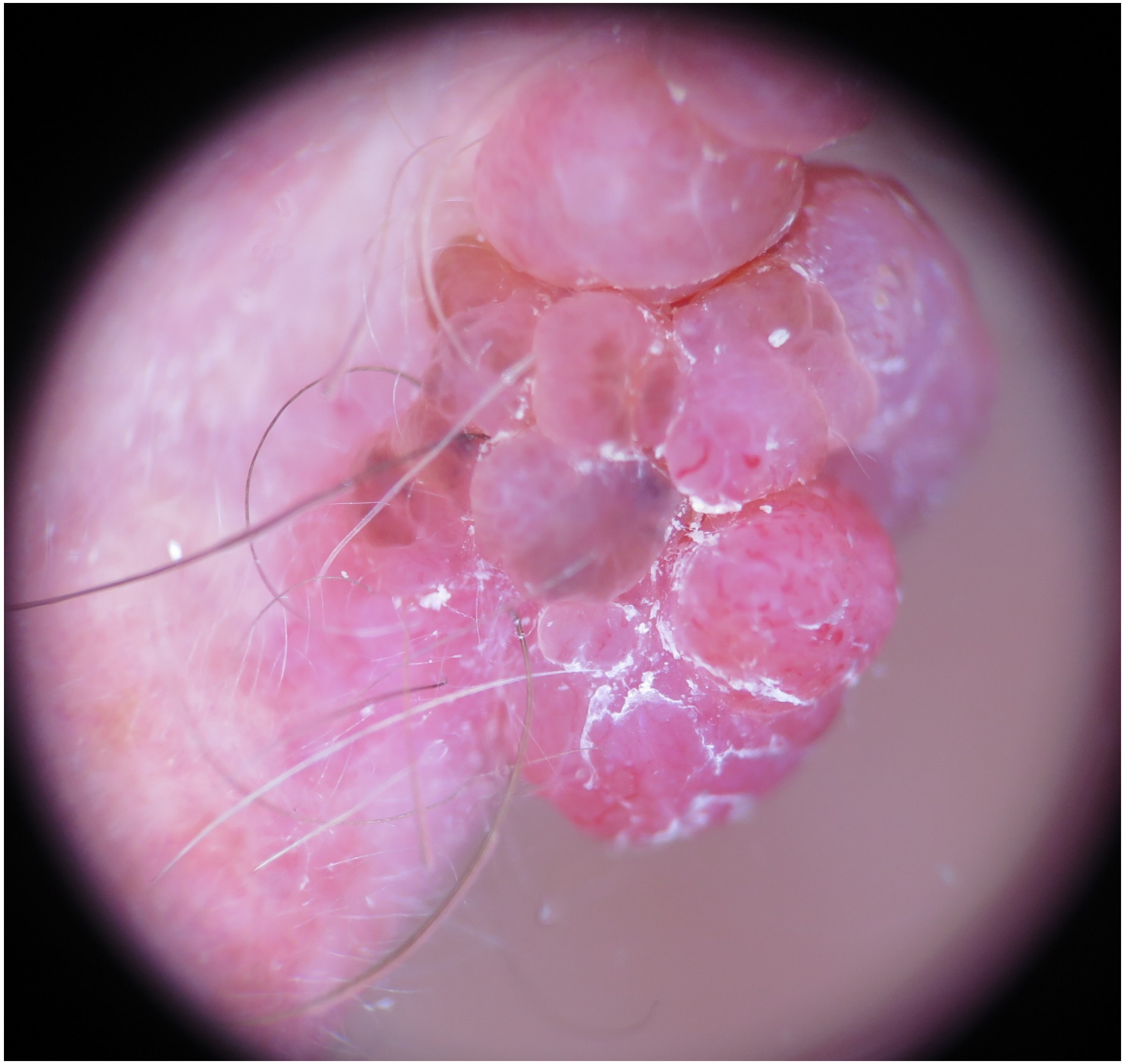

Location: Ear

Invasive) Breslow interval: > 4mm

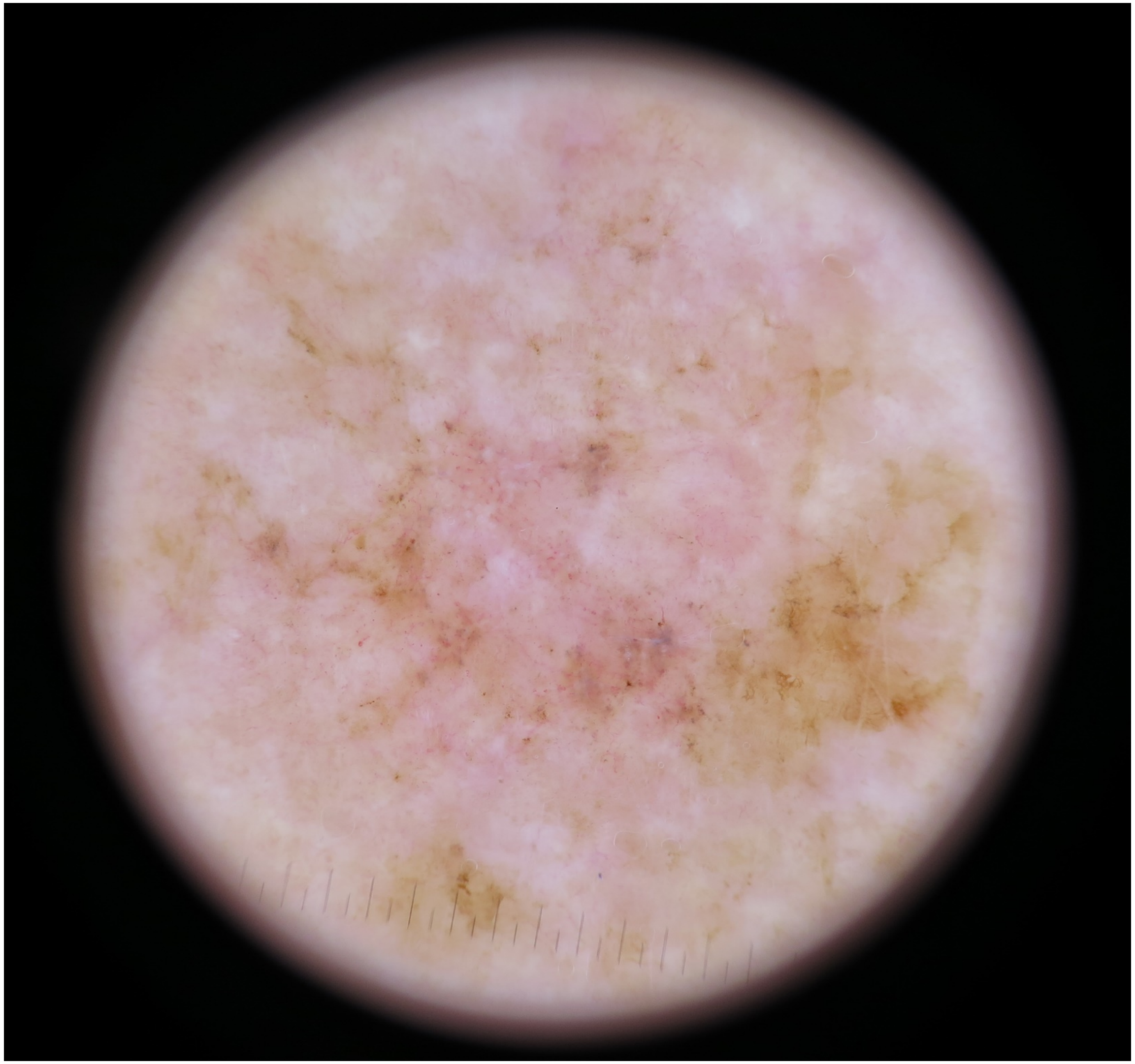

Location: Upper extremity

In situ melanoma

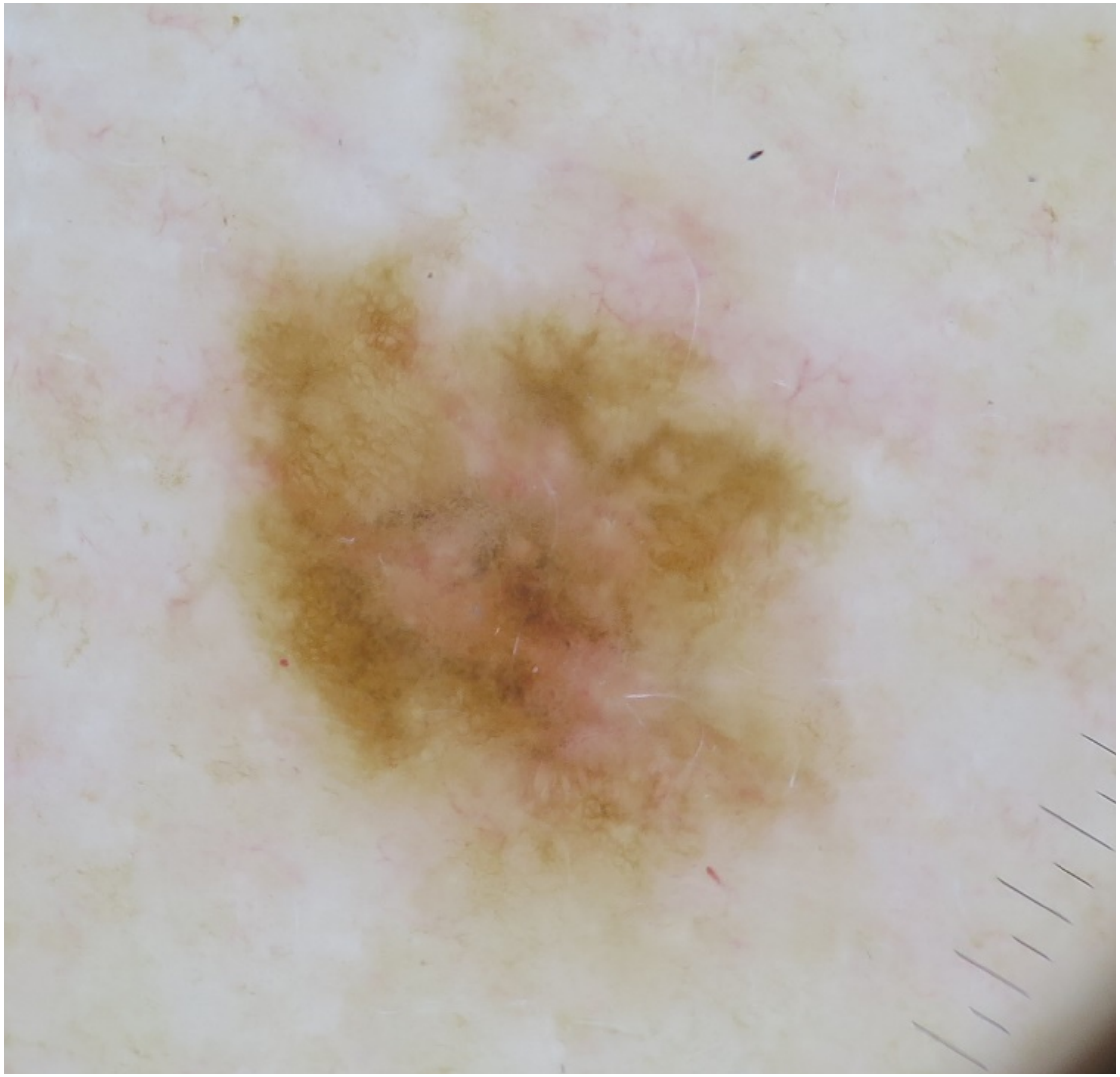

Location: Trunk

In situ melanoma

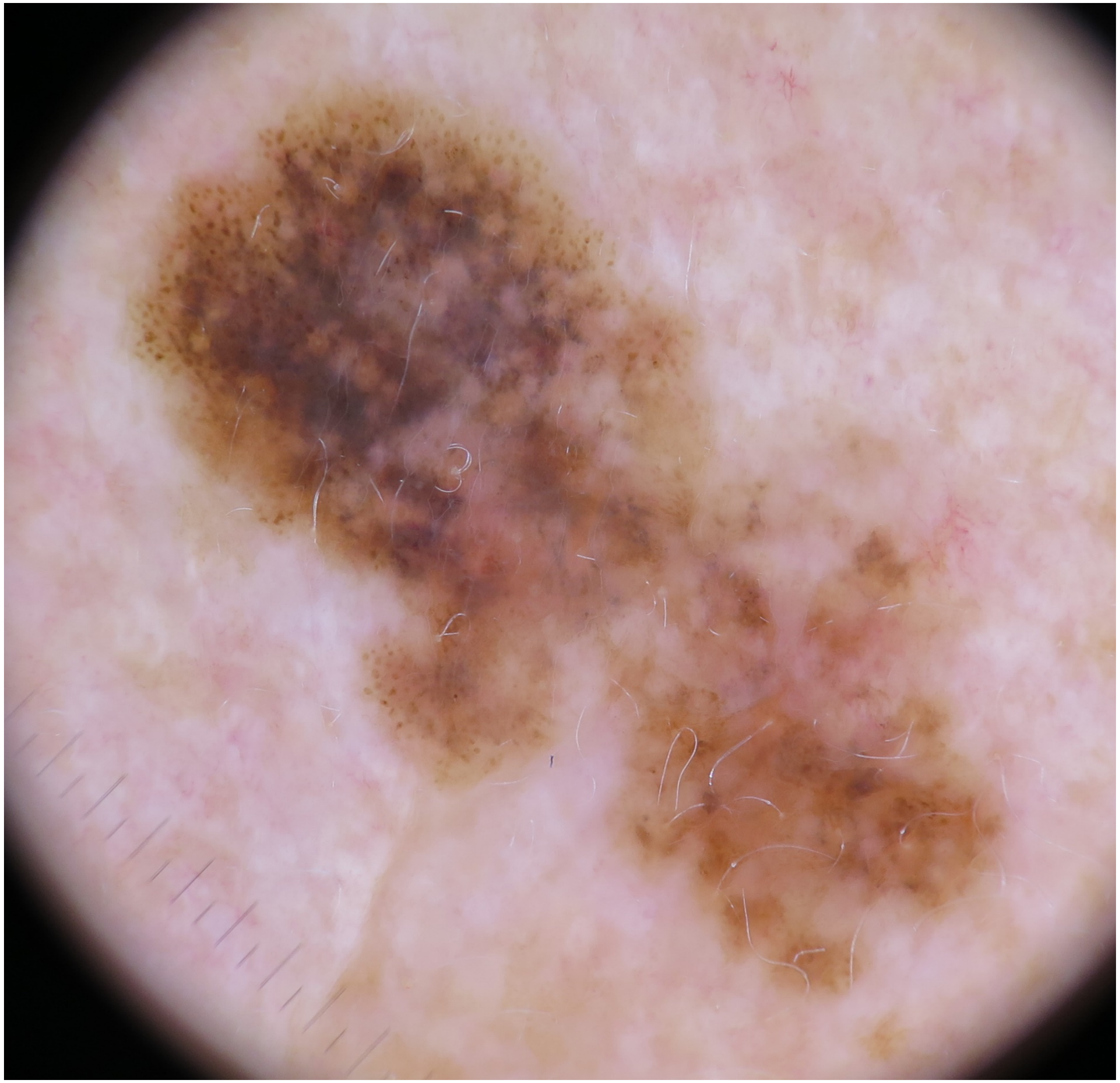

Location: Neck

Invasive) Breslow interval: 0.6-0.8 mm

Case number 98

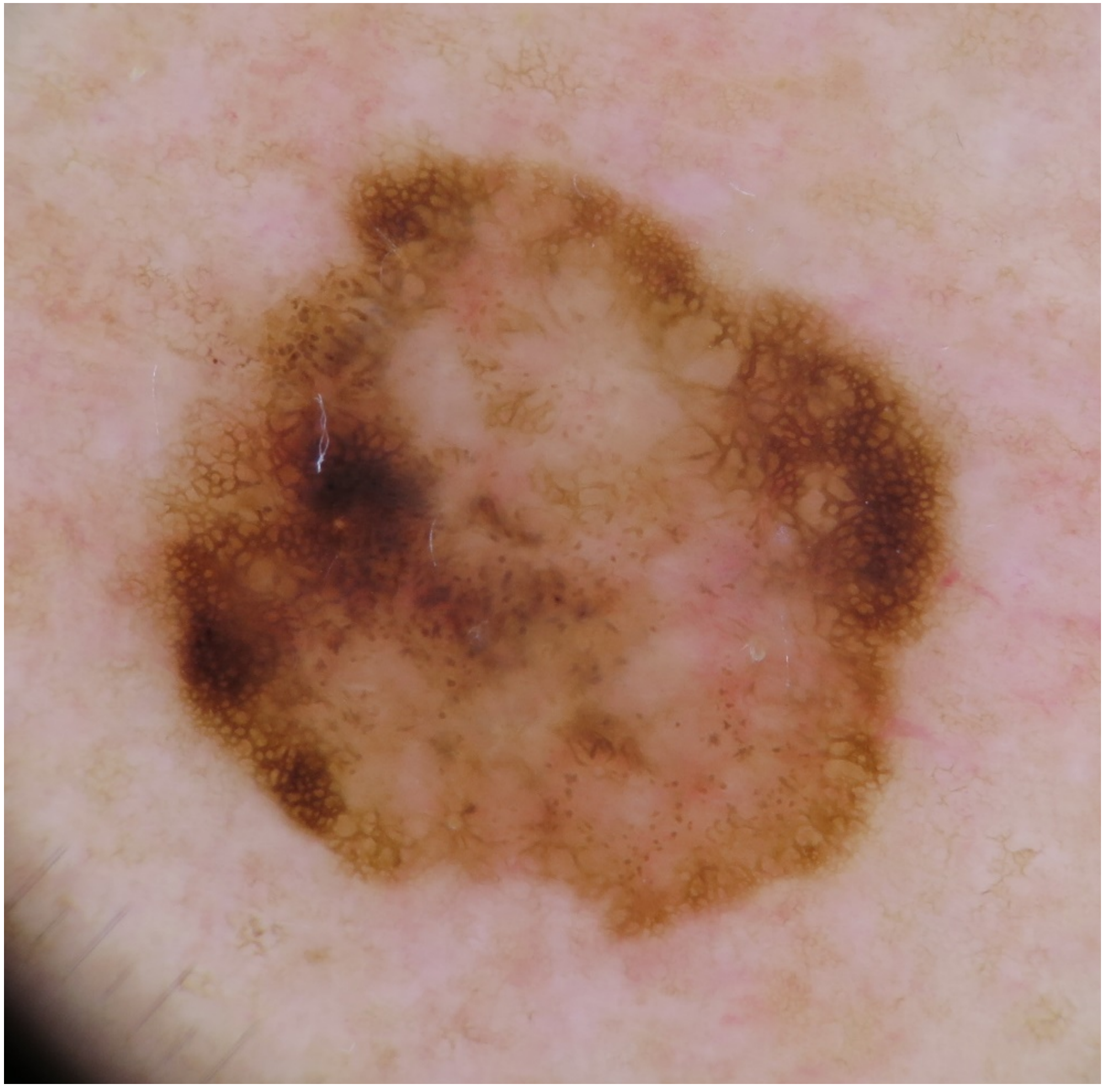

Location: Trunk

In situ melanoma

Case number 99

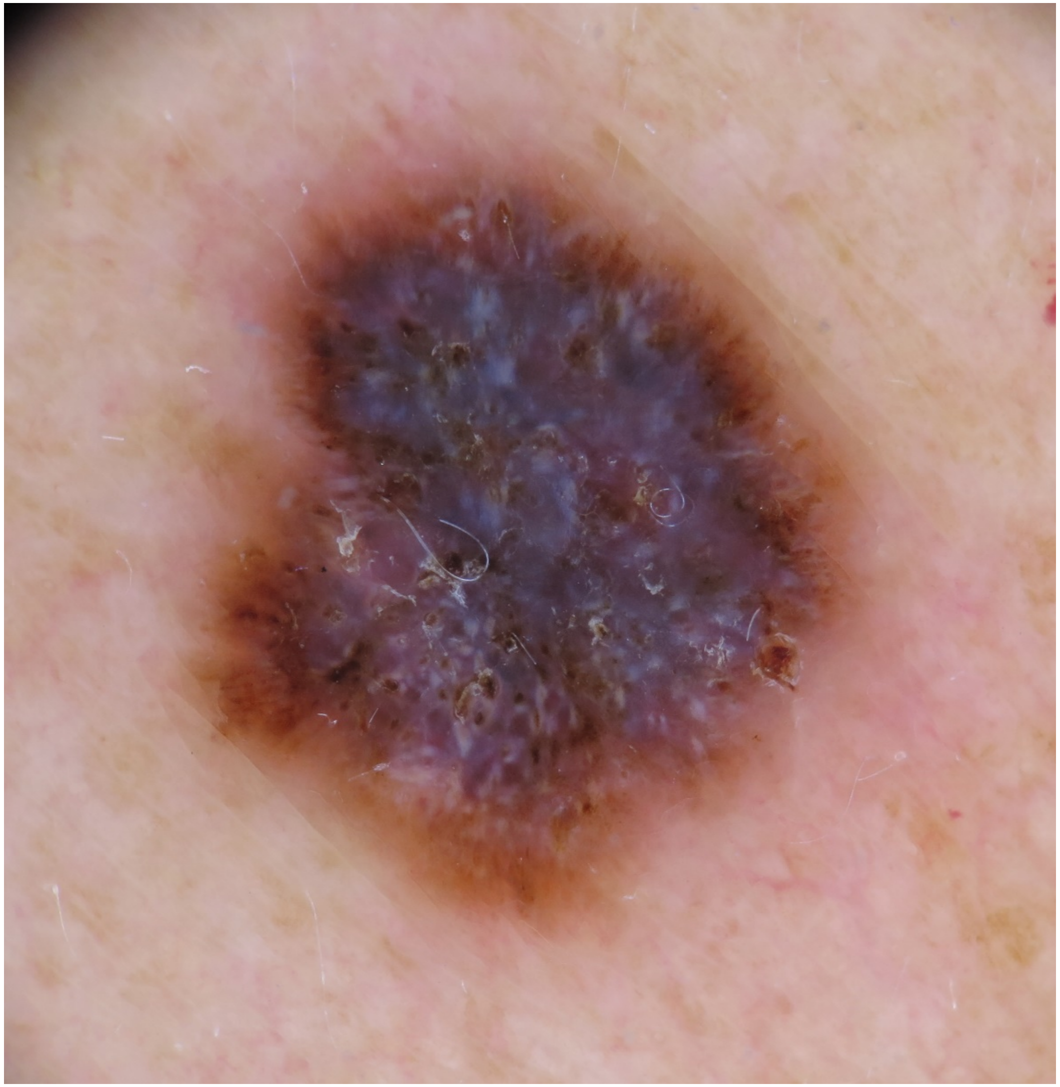

Location: Upper extremity

Invasive) Breslow interval: 1.1-2.0 mm

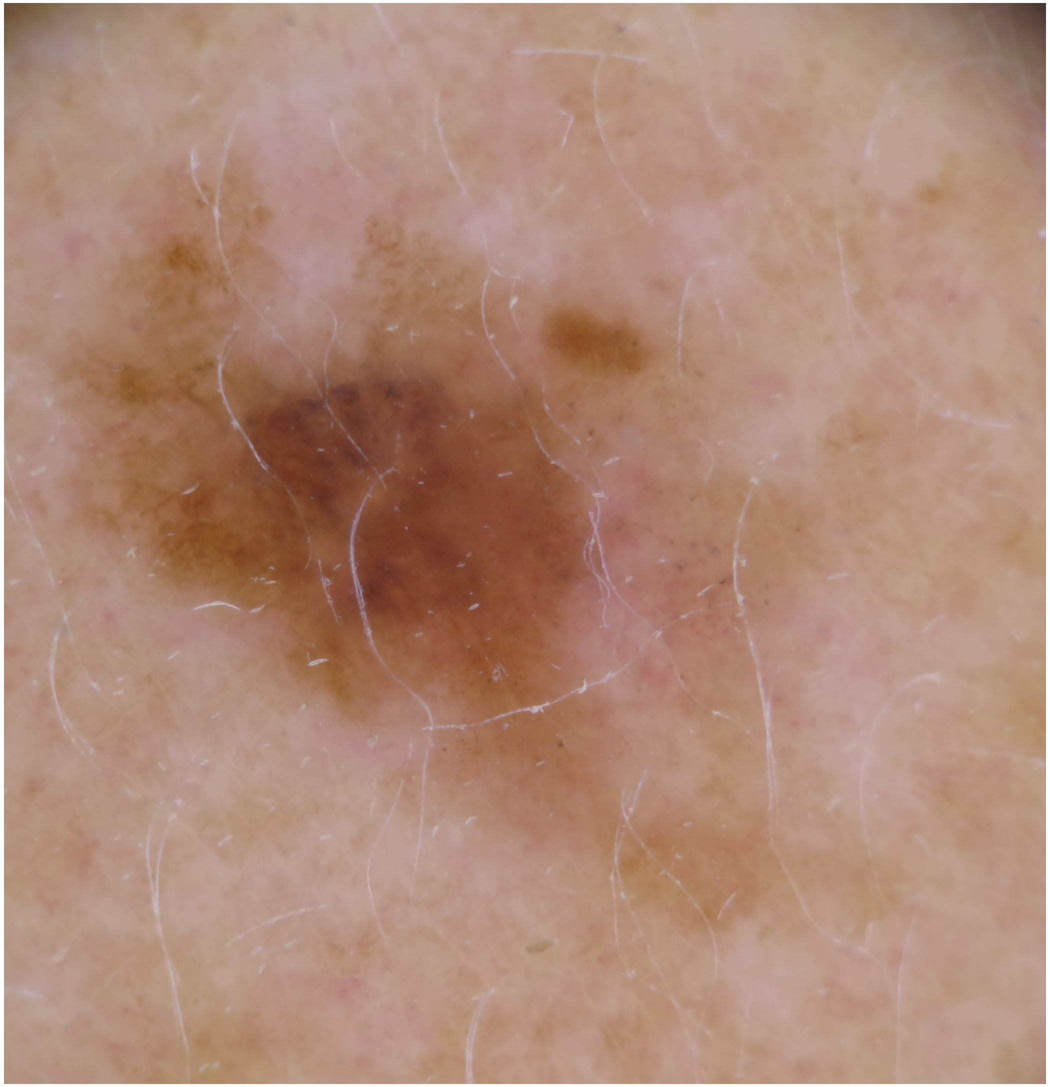

Location: Upper extremity

In situ melanoma

Case number 101

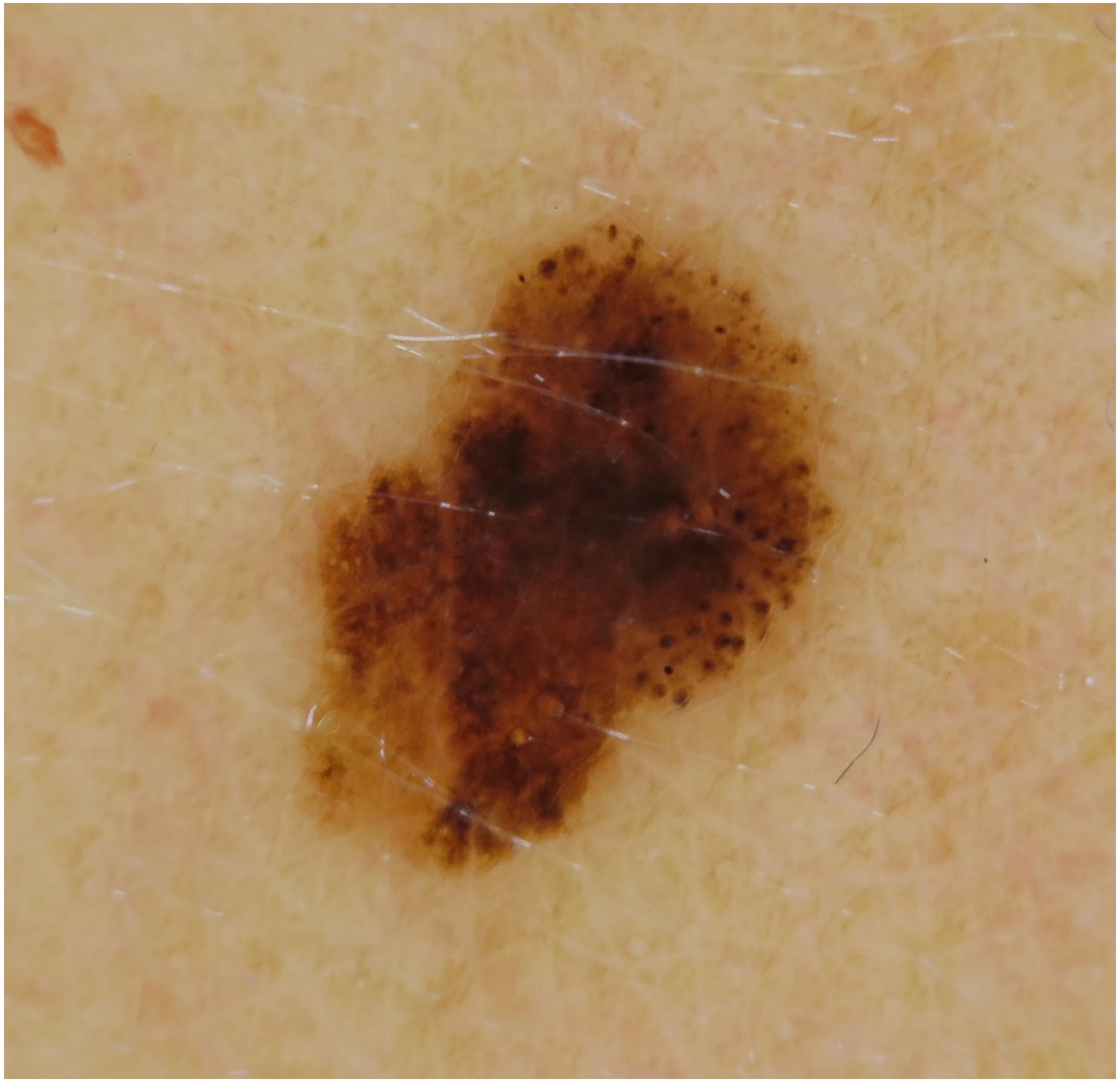

Location: Upper extremity

In situ melanoma

Case number 102

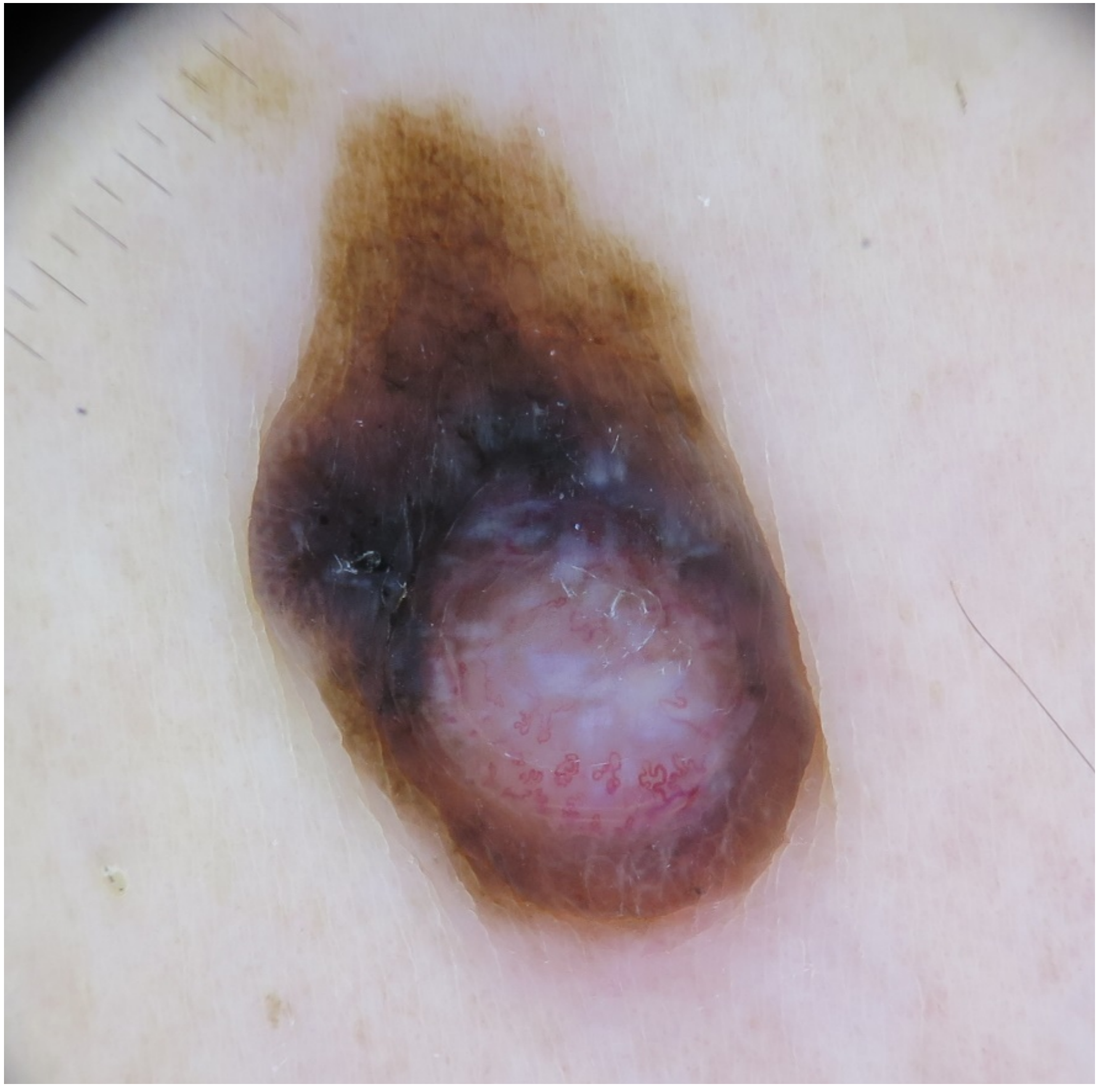

Location: Lower extremity

Invasive) Breslow interval: 2.1-4.0 mm

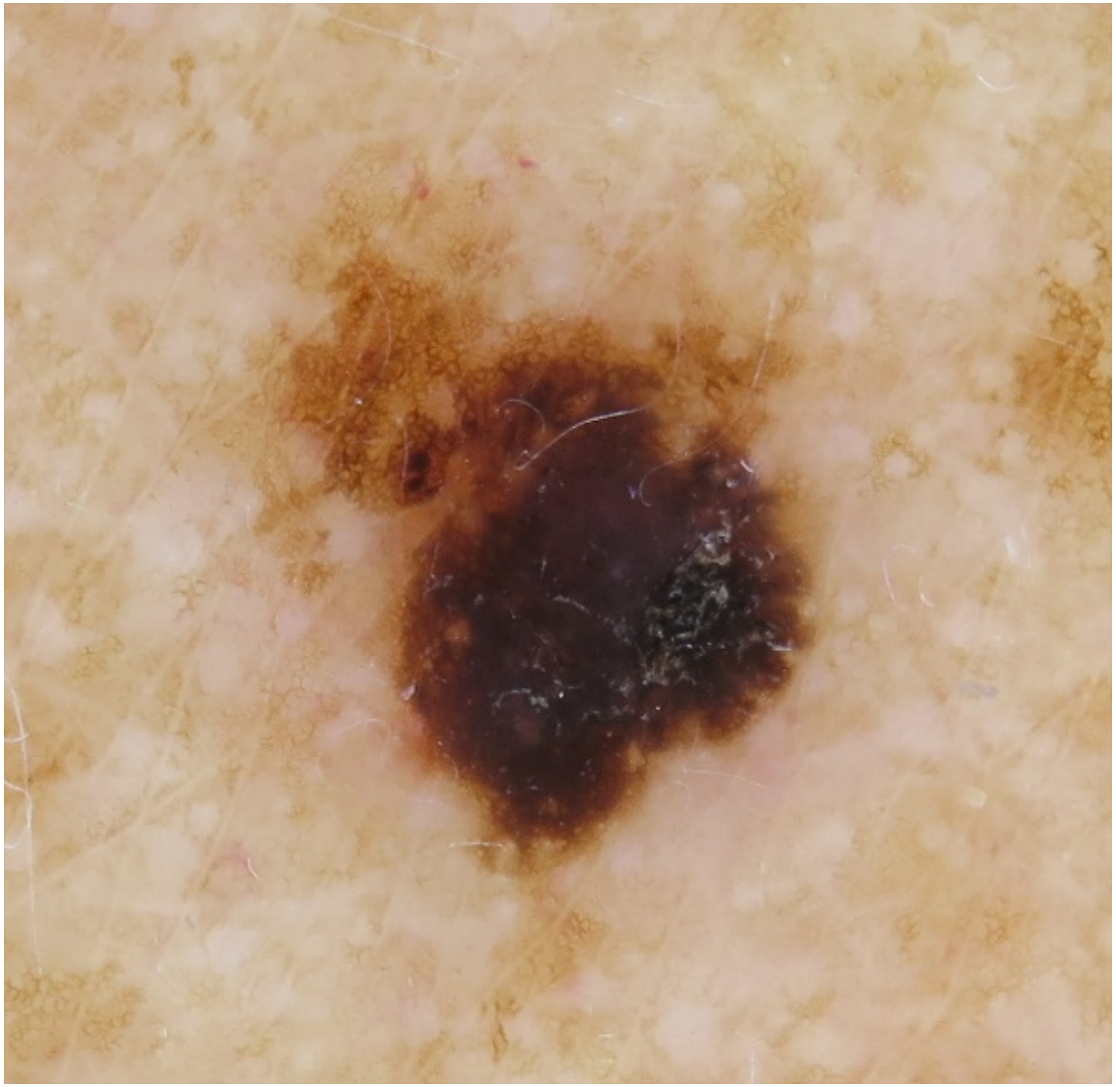

Location: Upper extremity

Invasive) Breslow interval: 0.6-0.8 mm

Case number 104

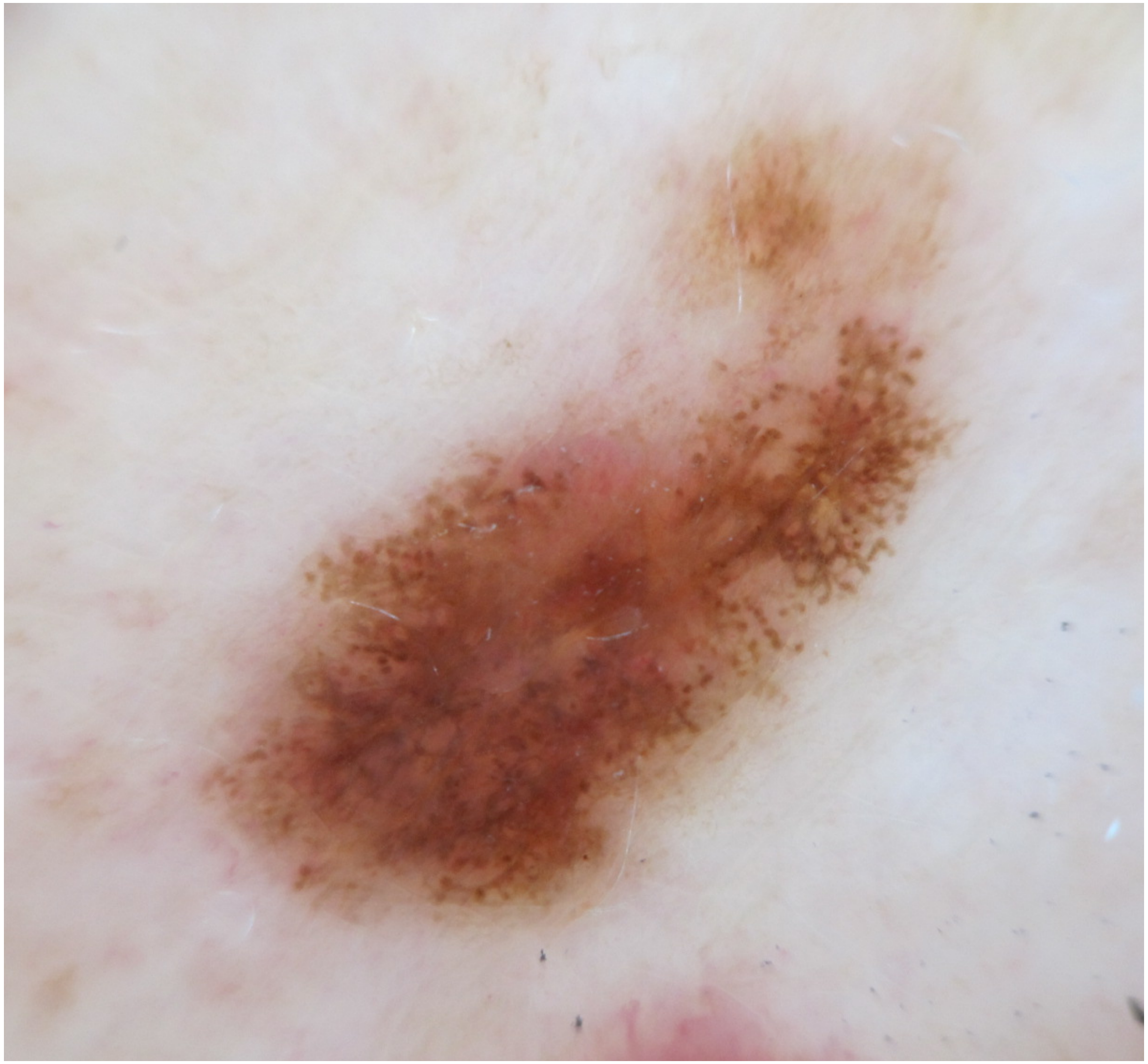

Location: Trunk

In situ melanoma

Case number 105

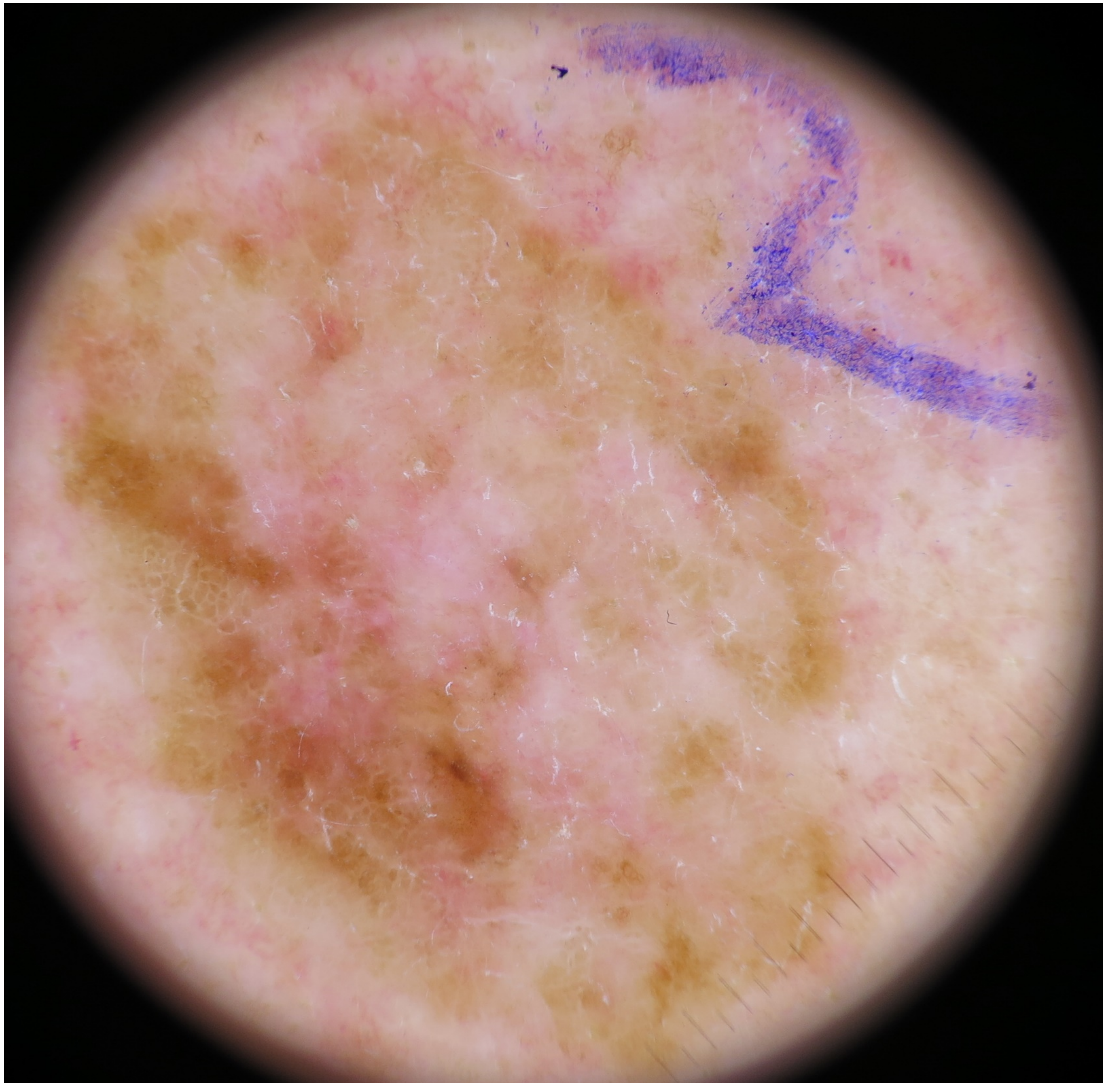

Location: Upper extremity

Invasive) Breslow interval: 0.1-0.5 mm

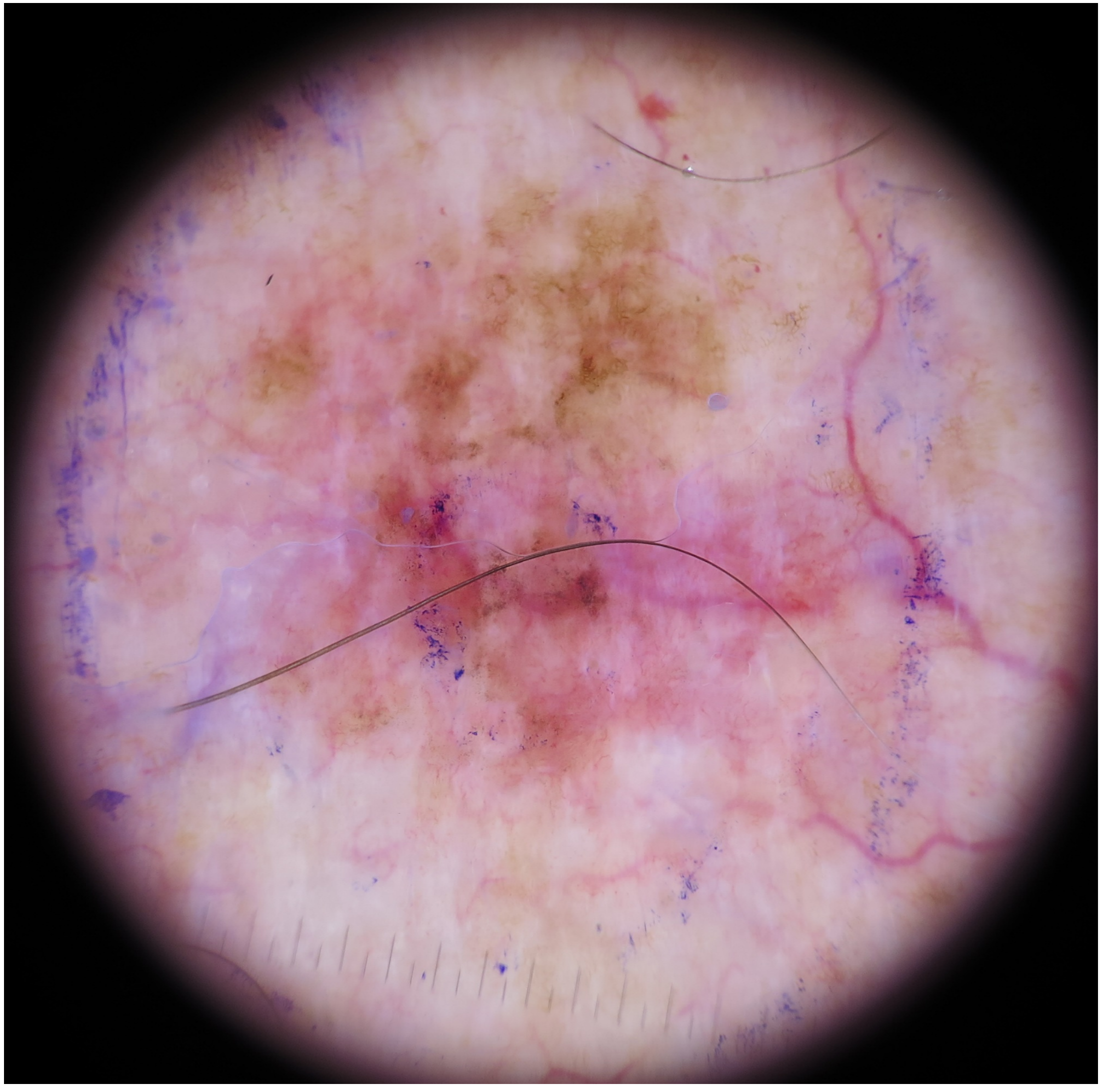

Location: Trunk

In situ melanoma

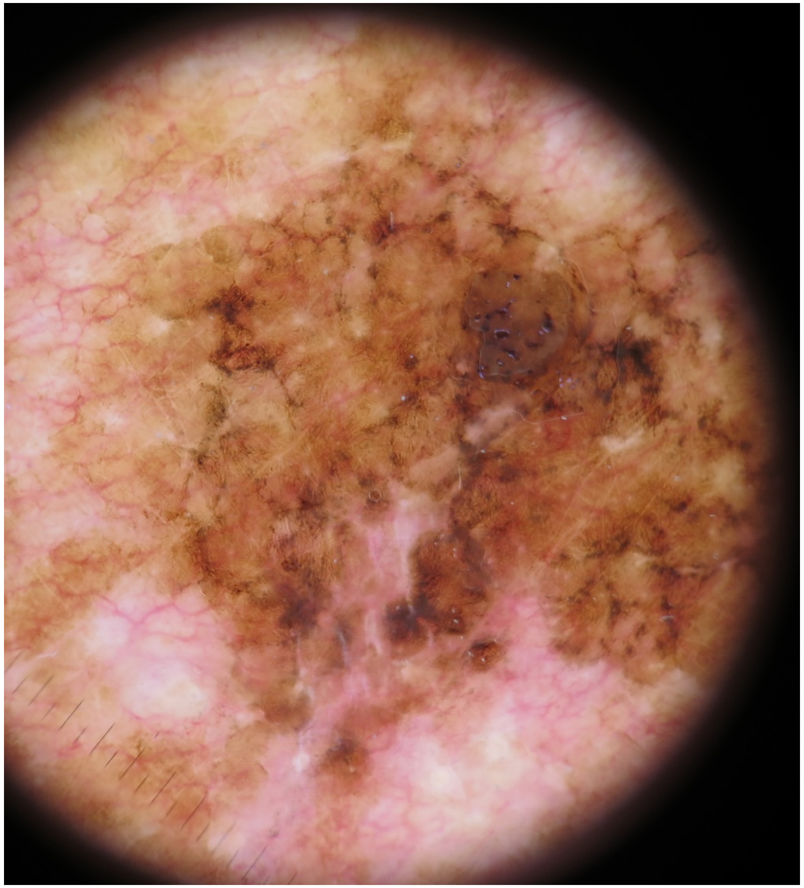

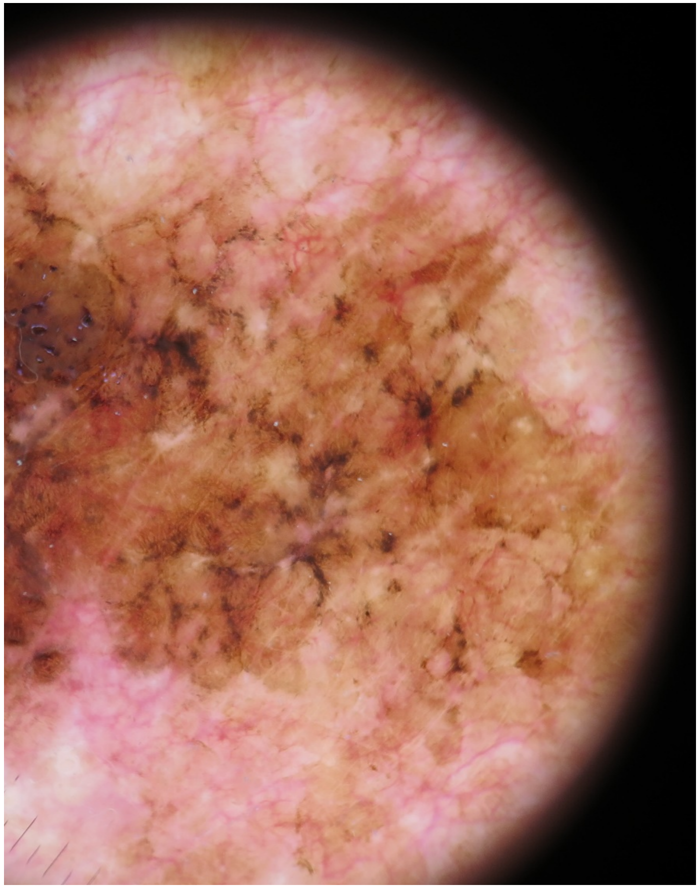

Location: Trunk

In situ melanoma

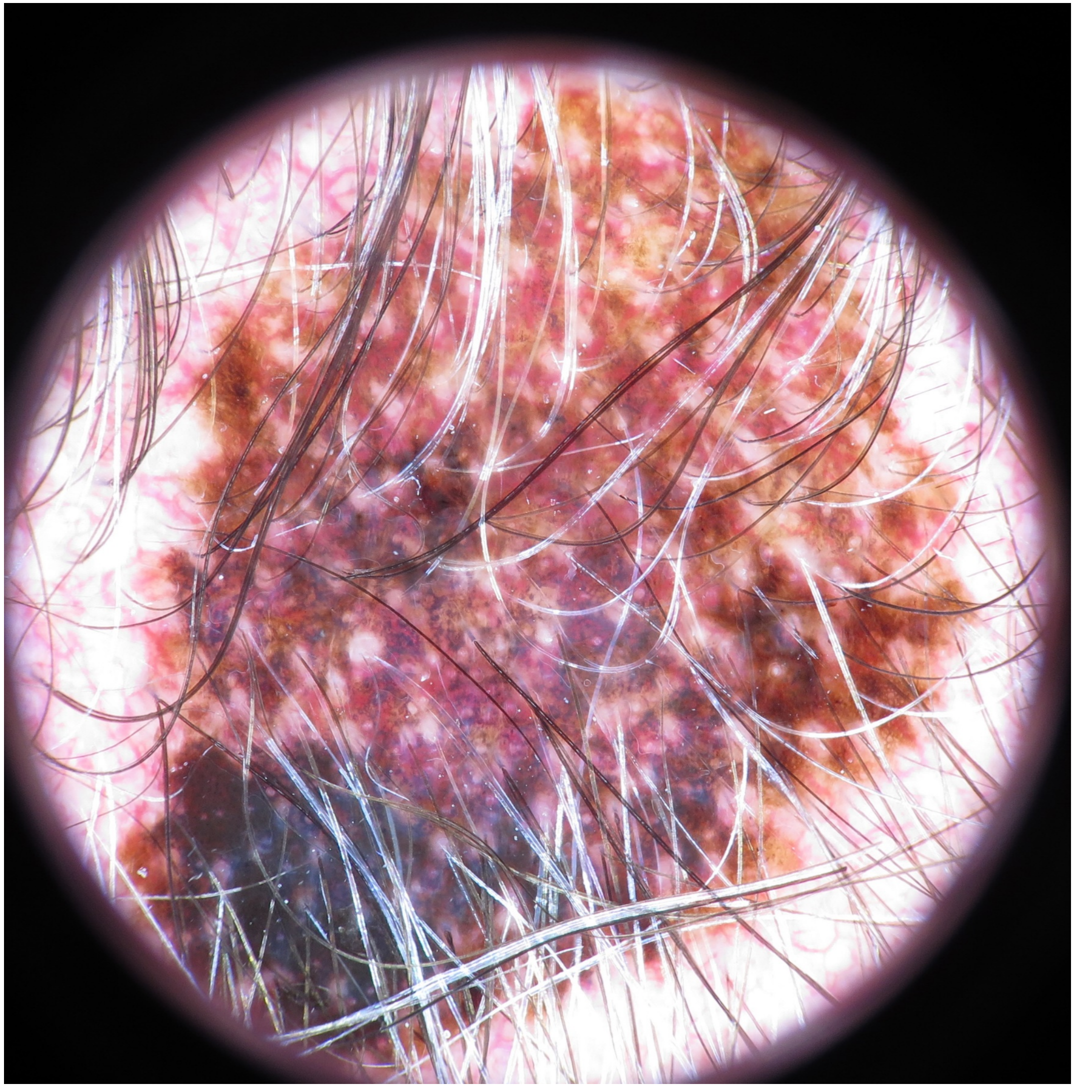

Location: Scalp

In situ melanoma

Case number 109

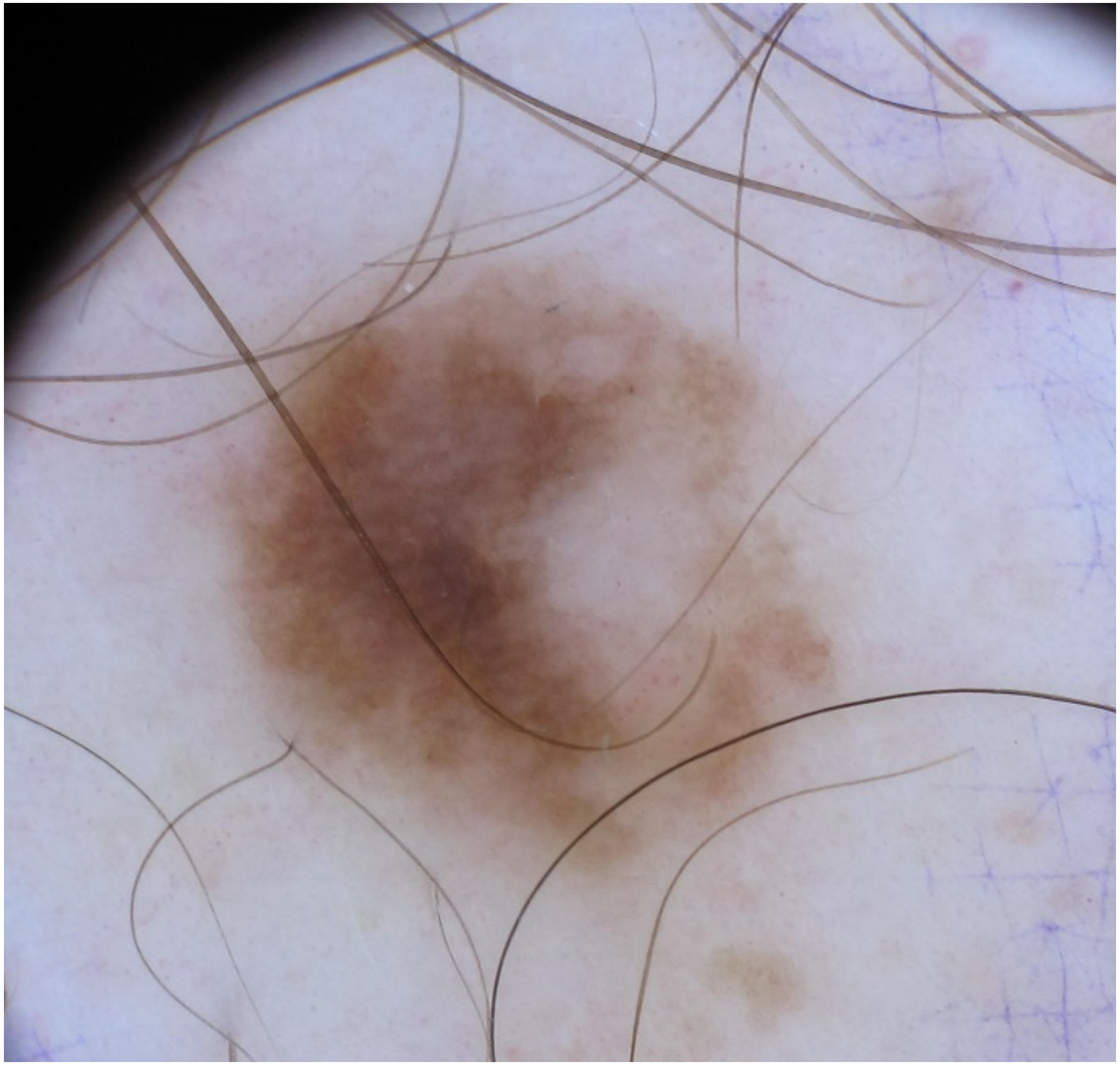

Location: Trunk

Invasive) Breslow interval: 0.6-0.8 mm

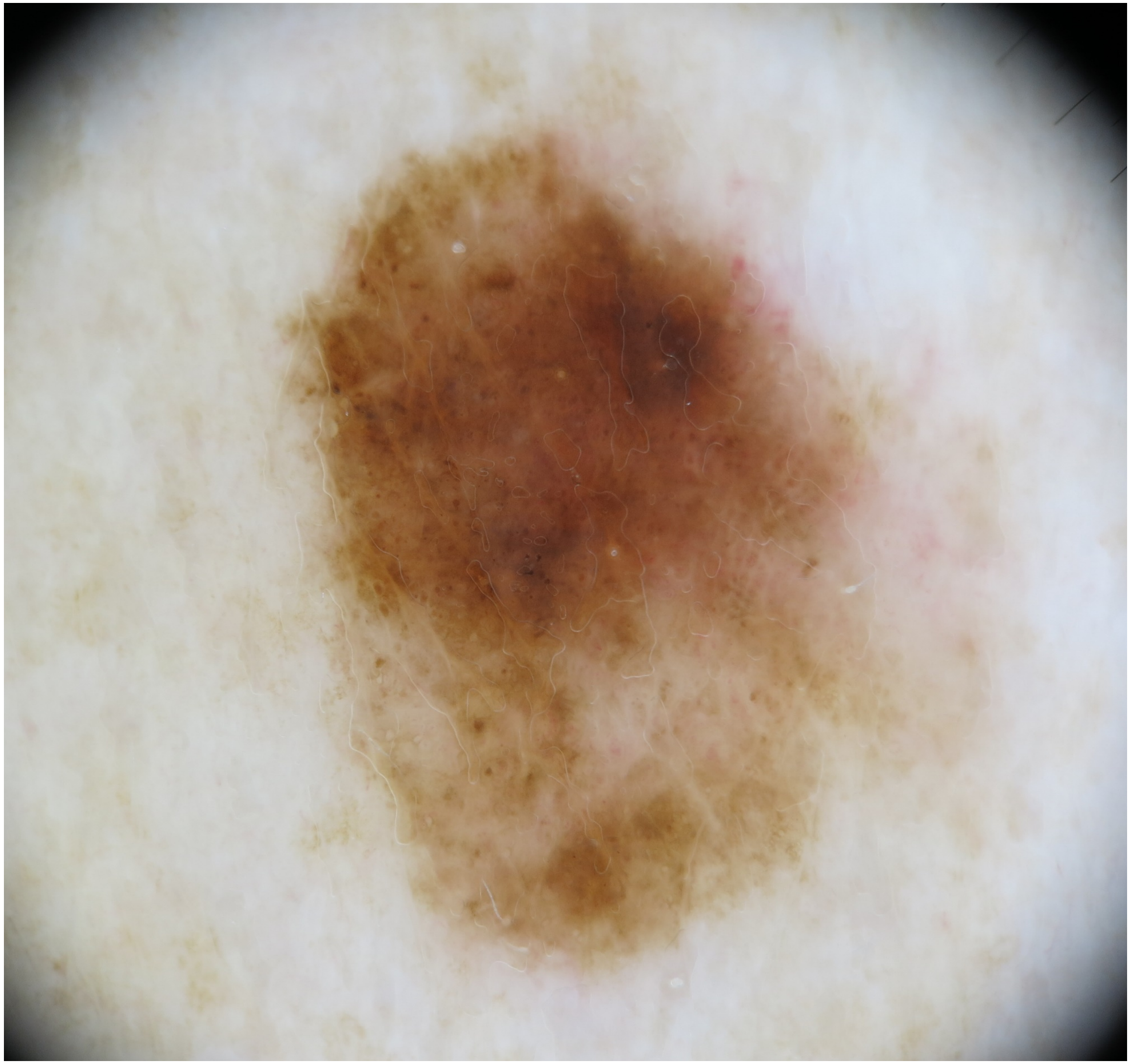

Location: Upper extremity

In situ melanoma

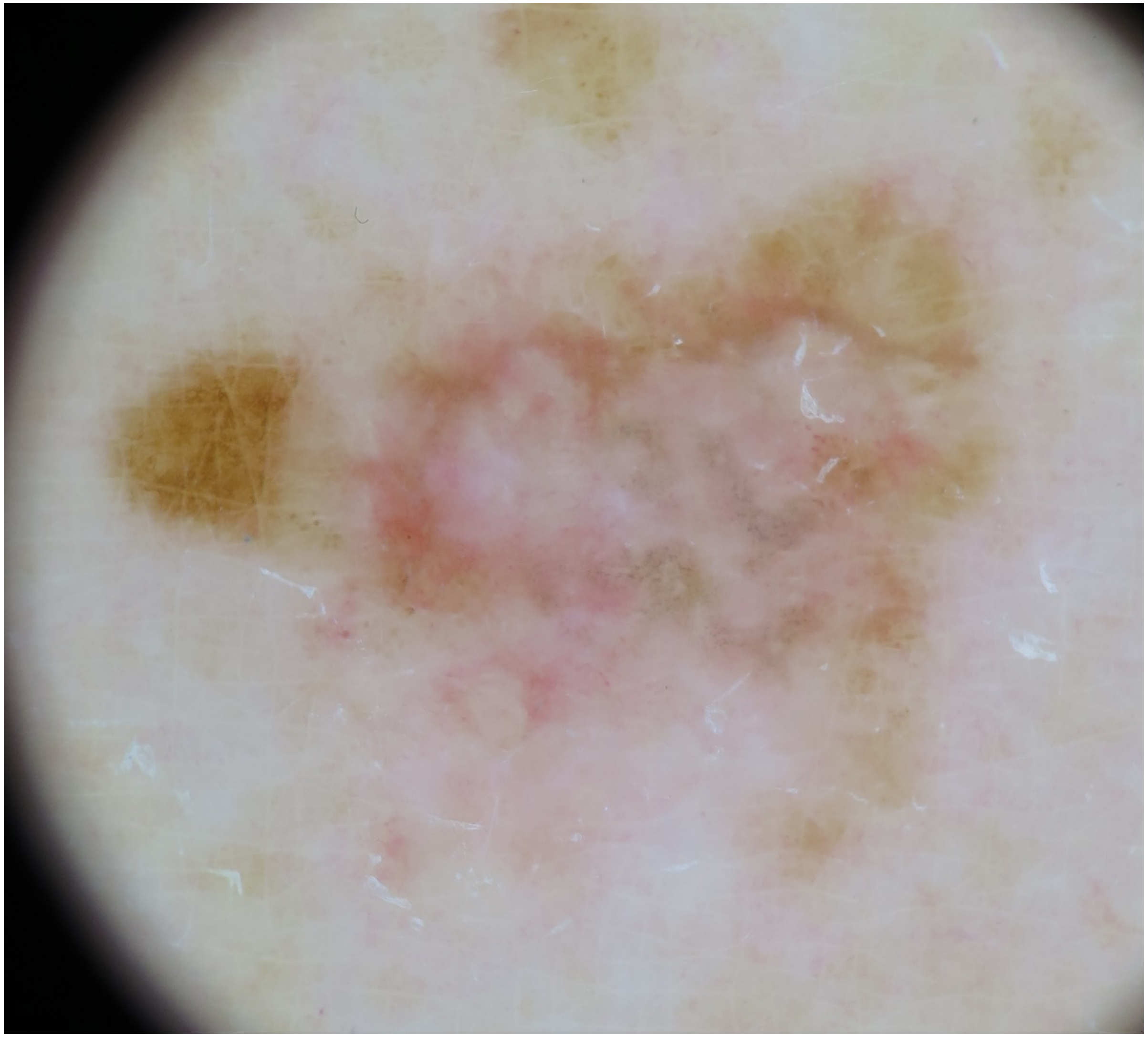

Location: Upper extremity

Invasive) Breslow interval: 0.1-0.5 mm

Case number 112

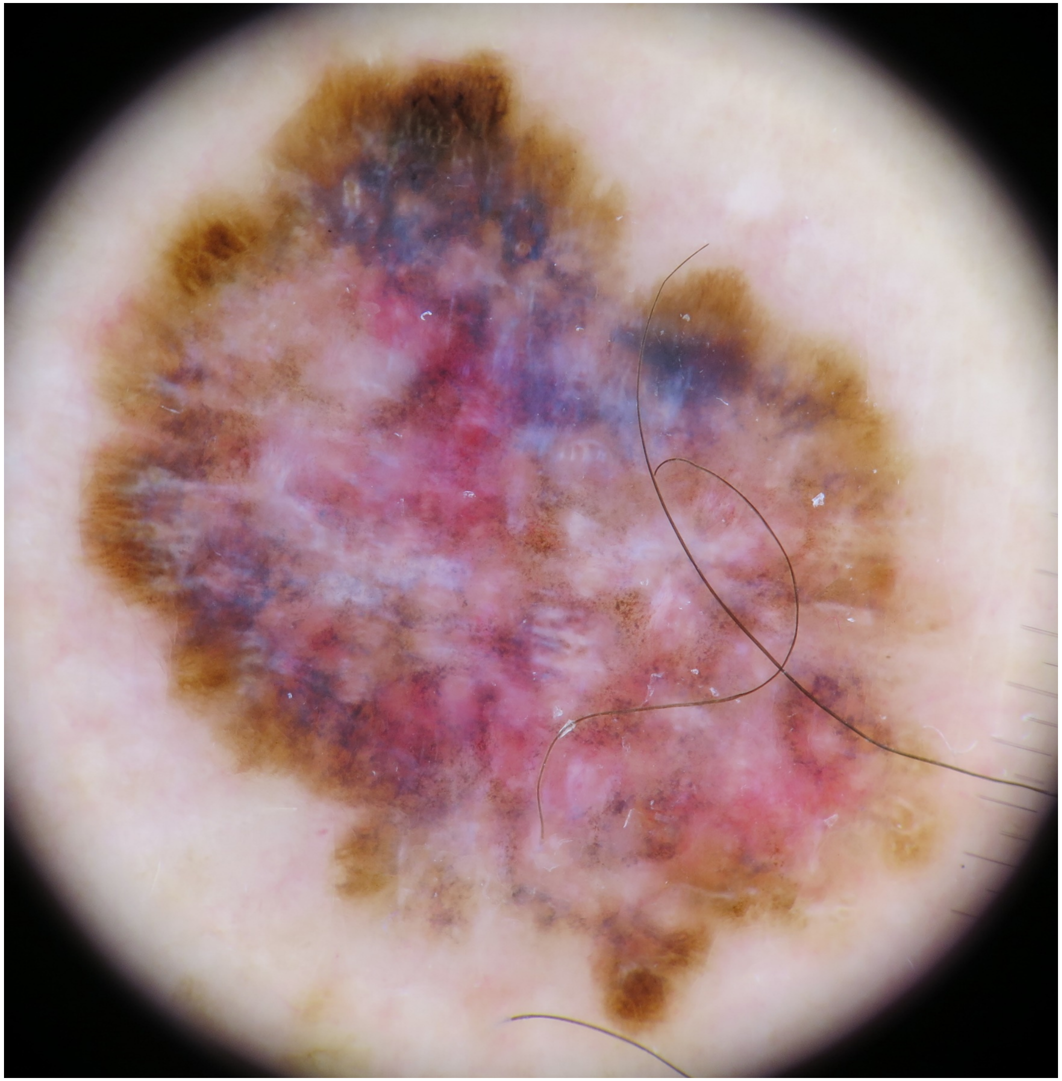

Location: Upper extremity

Invasive) Breslow interval: 0.1-0.5 mm

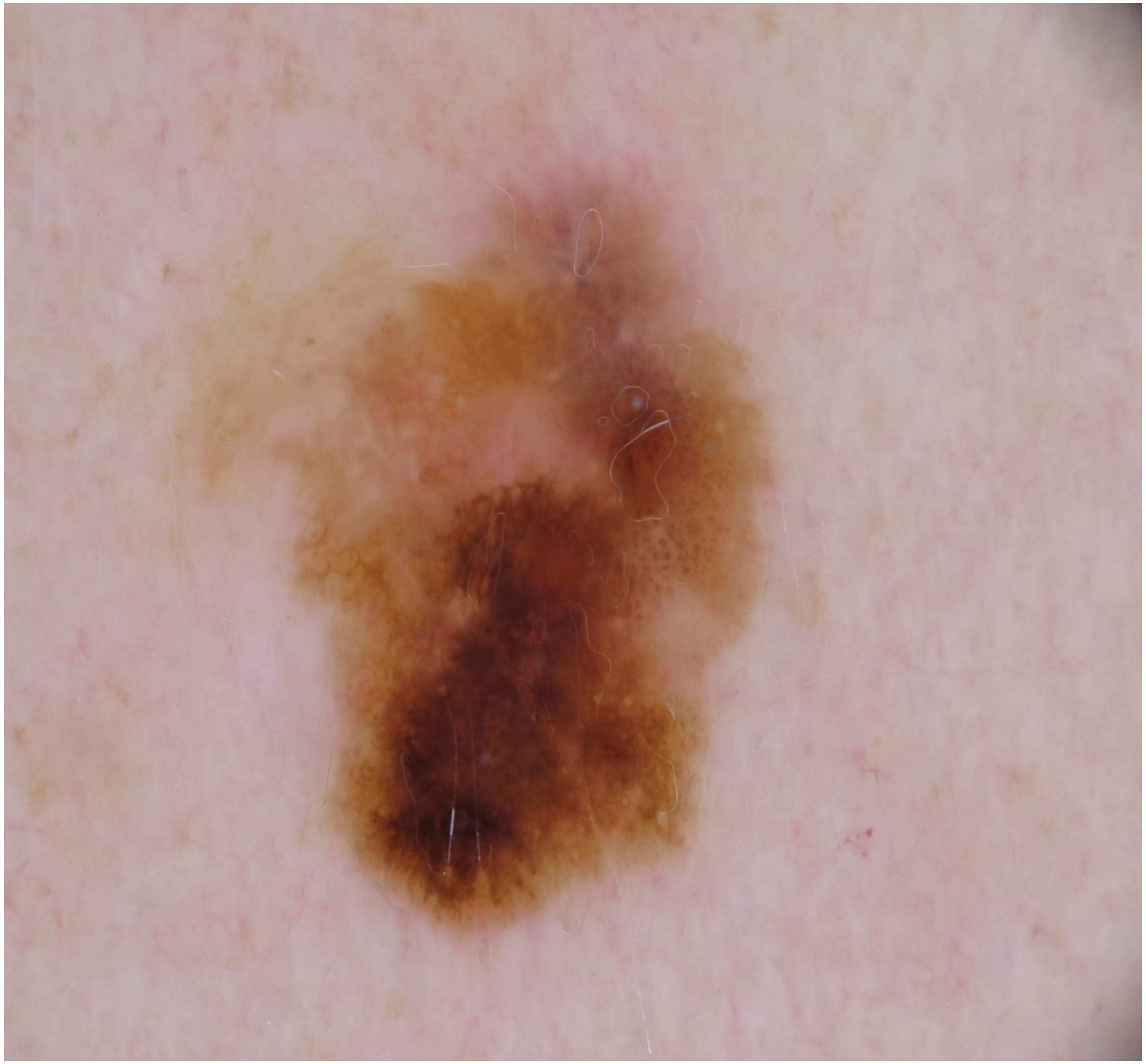

Location: Upper extremity

Invasive) Breslow interval: 0.1-0.5 mm

Case number 114

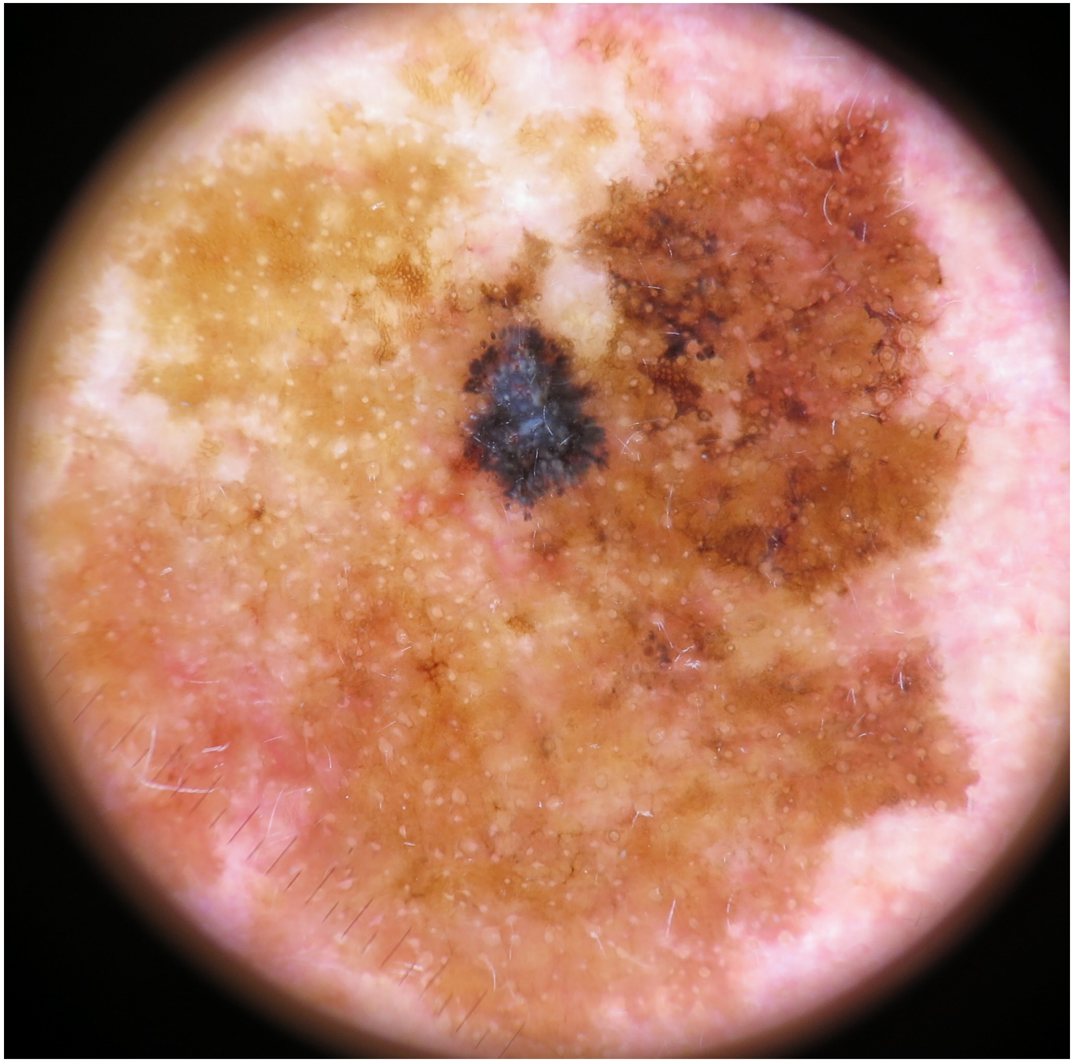

Location: Face

Invasive) Breslow interval: 0.6-0.8 mm

Case number 115

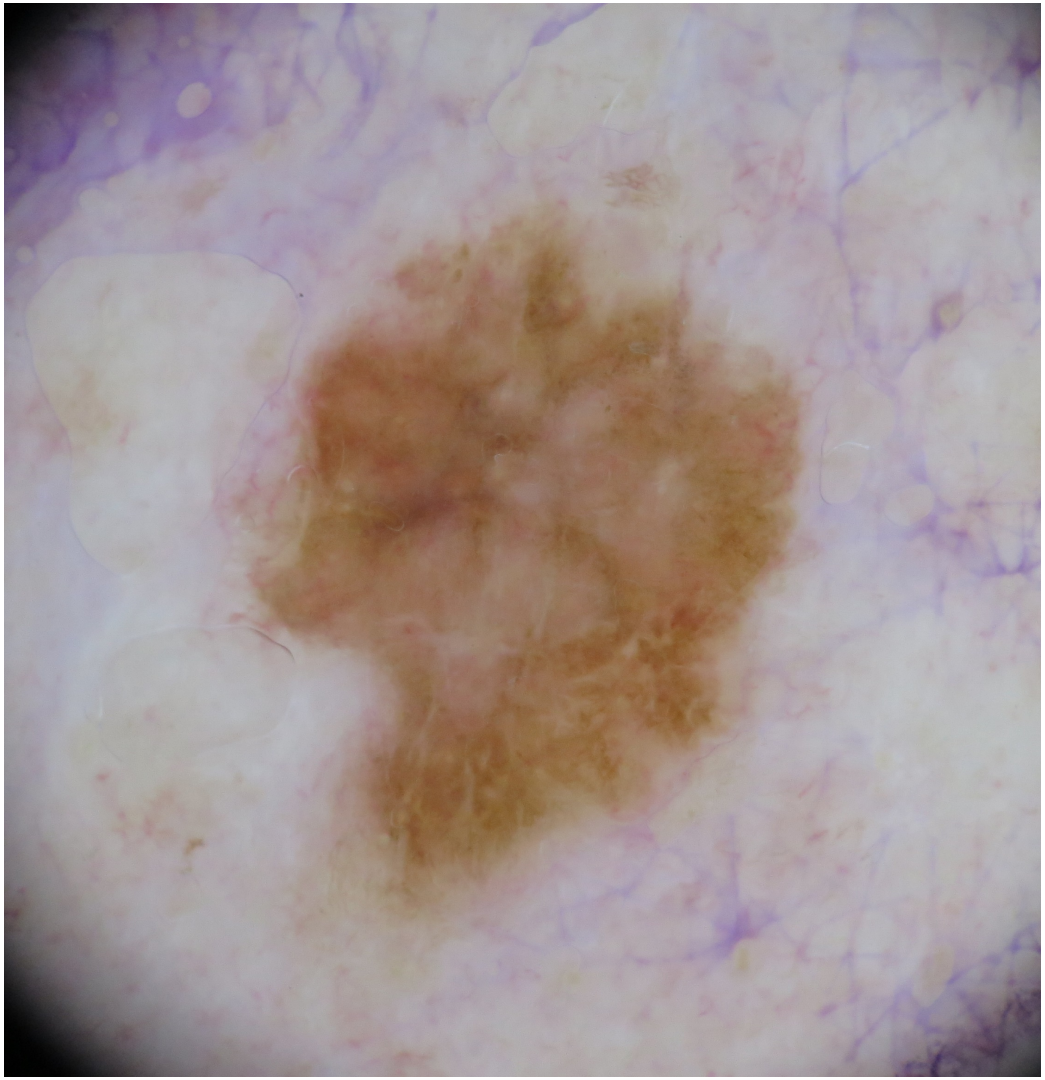

Location: Trunk

In situ melanoma

Case number 116

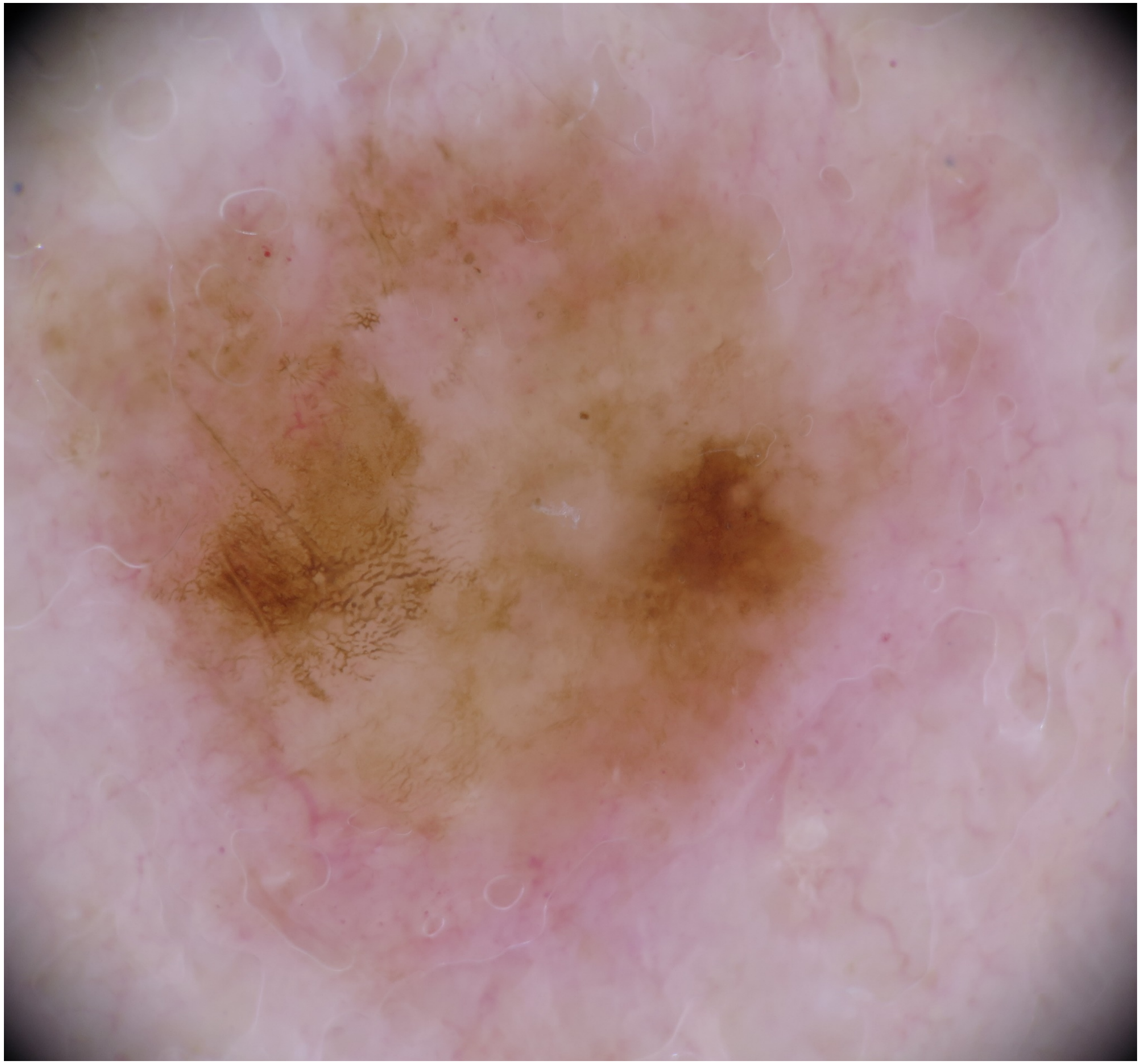

Location: Upper extremity

Invasive) Breslow interval: 0.1-0.5 mm

Case number 117

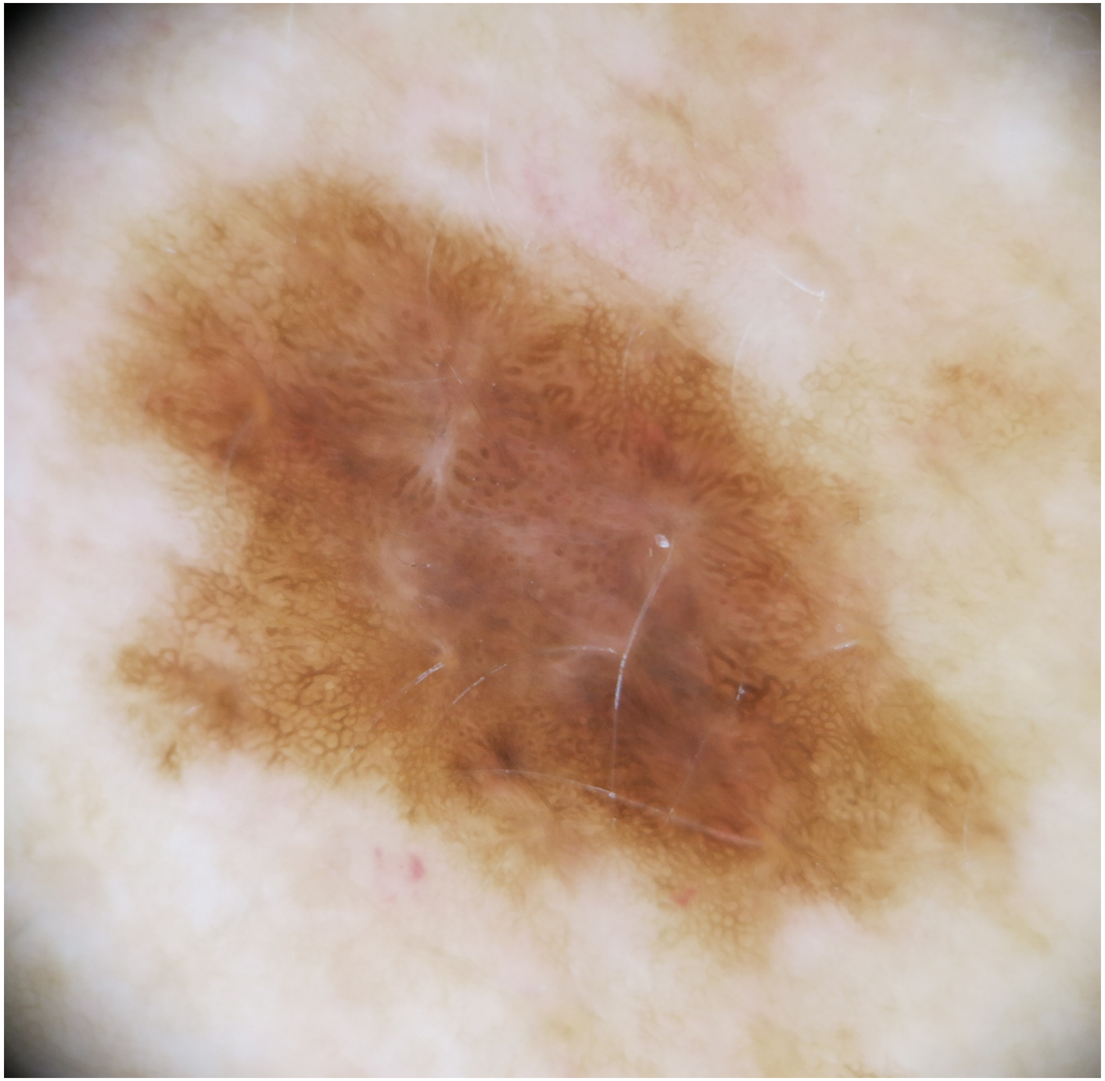

Location: Trunk

In situ melanoma

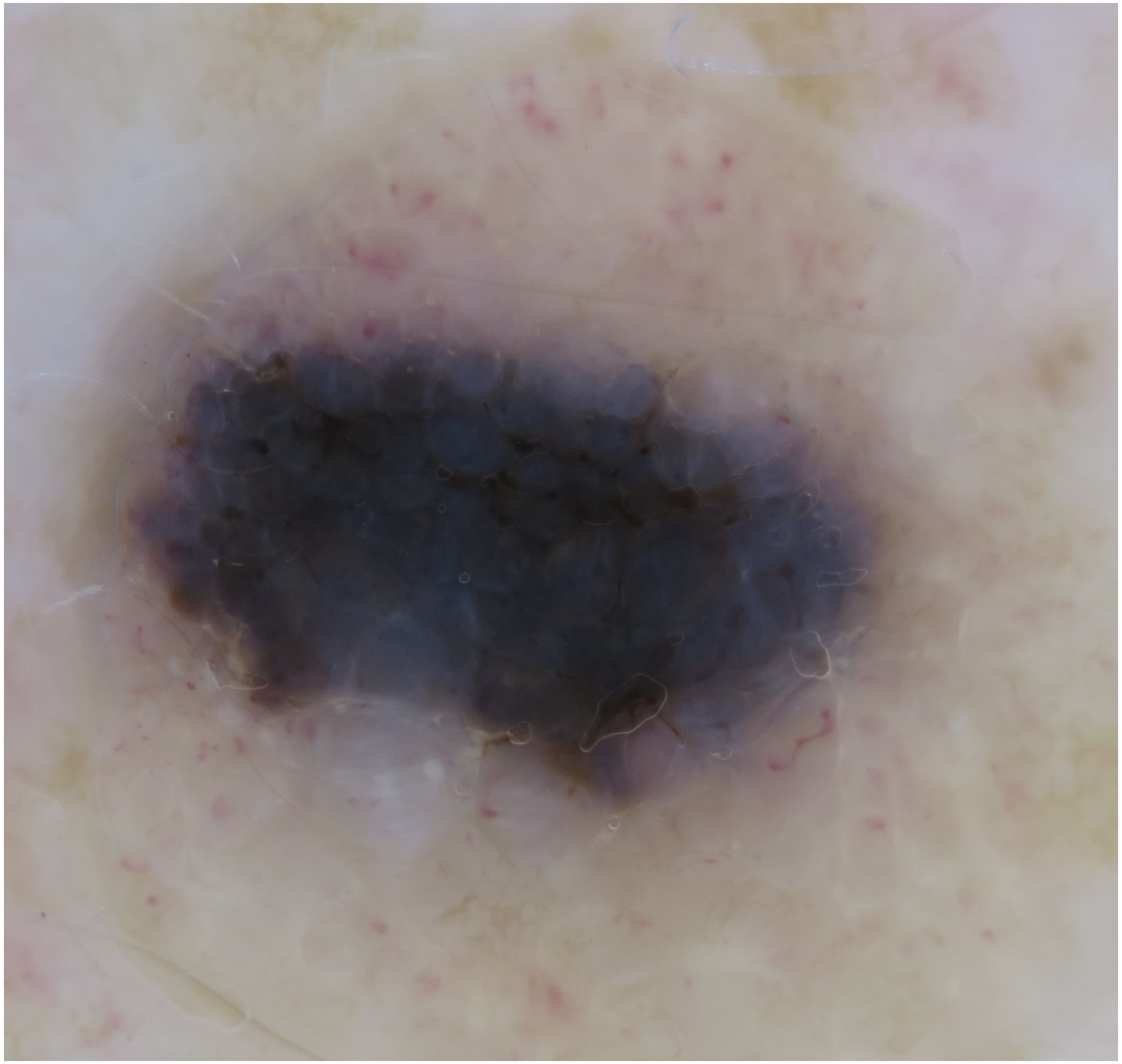

Location: Upper extremity

Invasive) Breslow interval: 2.1-4.0 mm

Case number 119

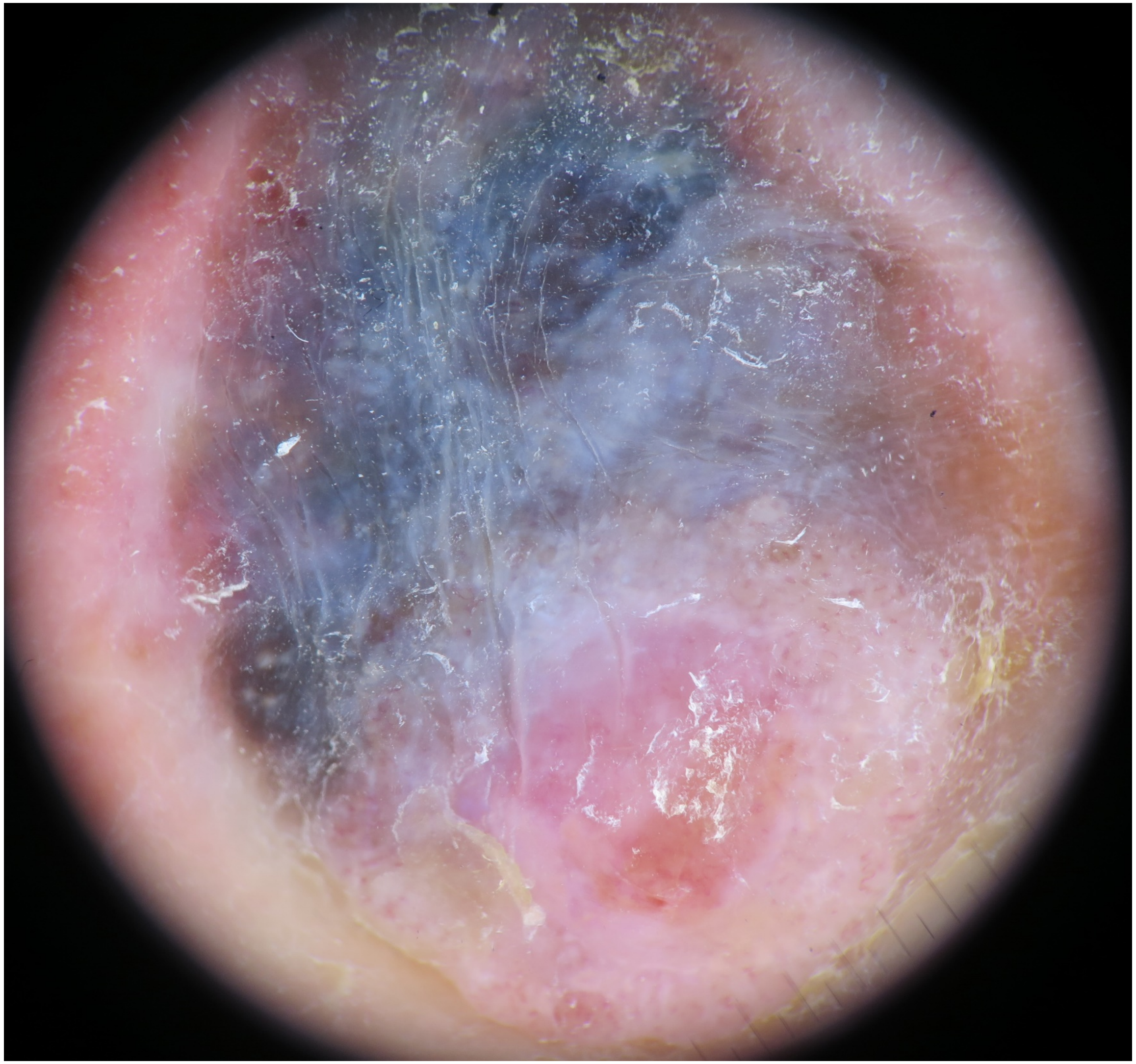

Location: Lower extremity

Invasive) Breslow interval: > 4mm

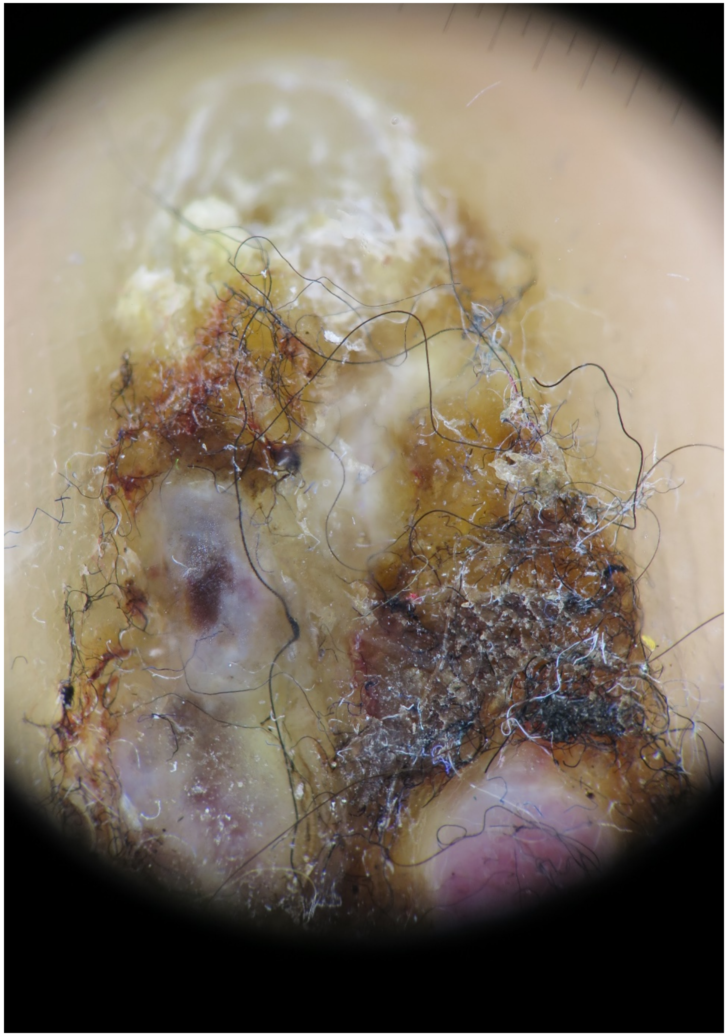

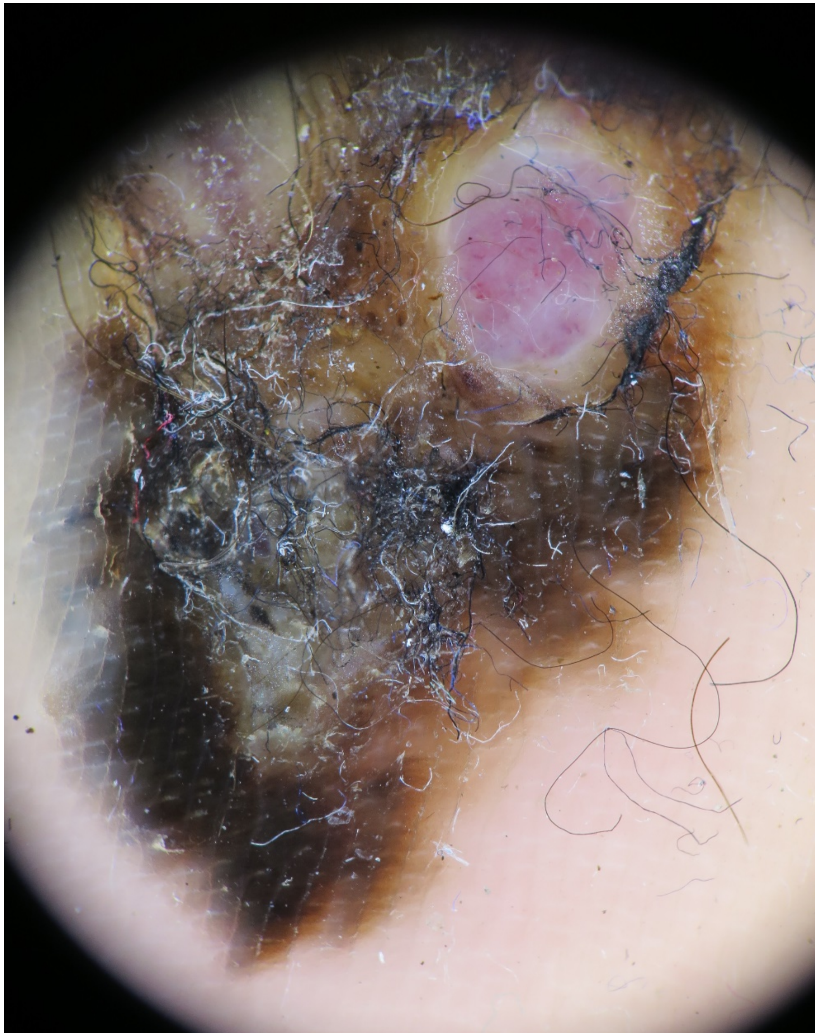

Location: Lower extremity

Invasive) Breslow interval: 2.1-4.0 mm

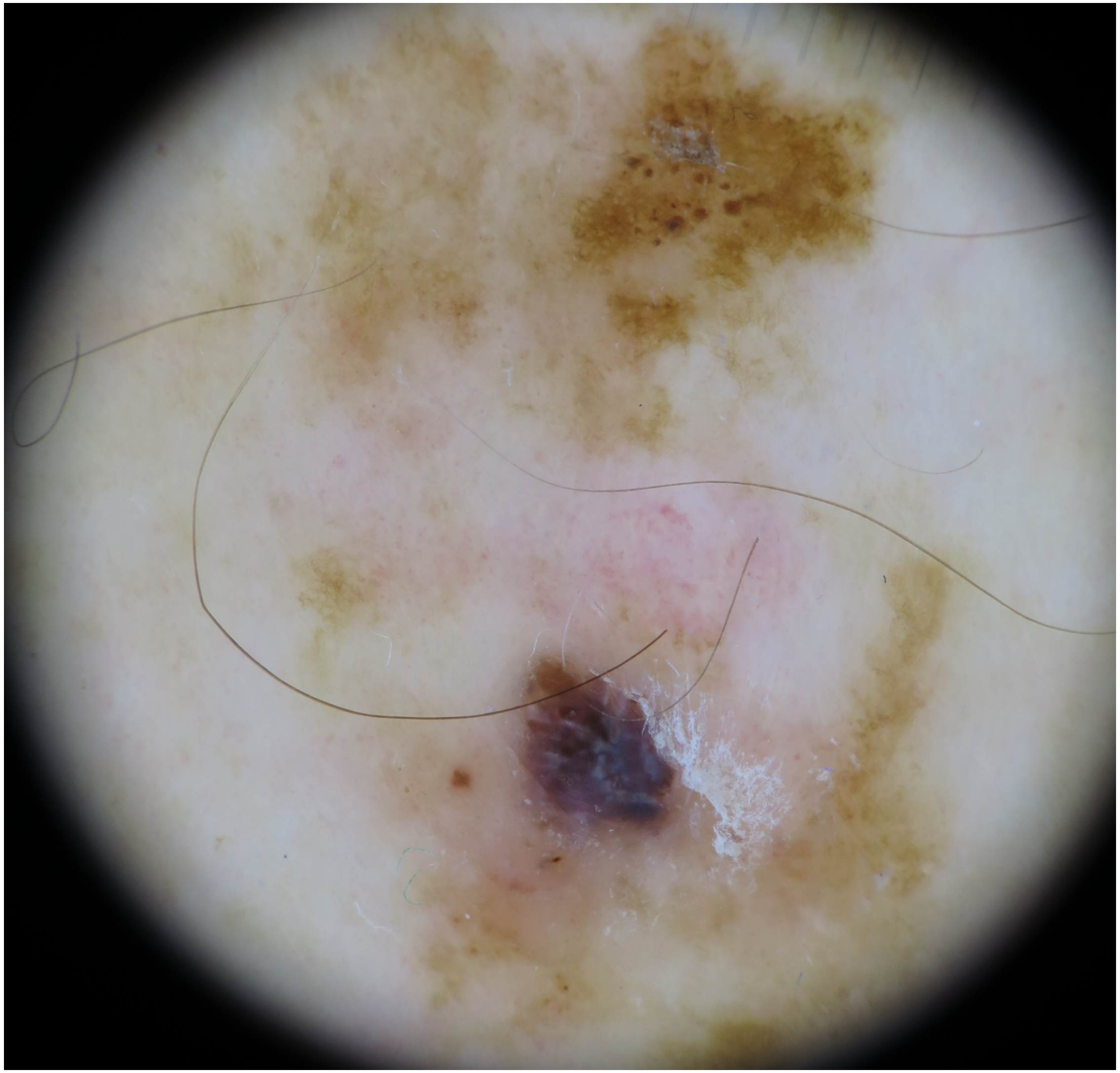

Location: Lower extremity

Invasive) Breslow interval: 1.1-2.0 mm

Case number 122

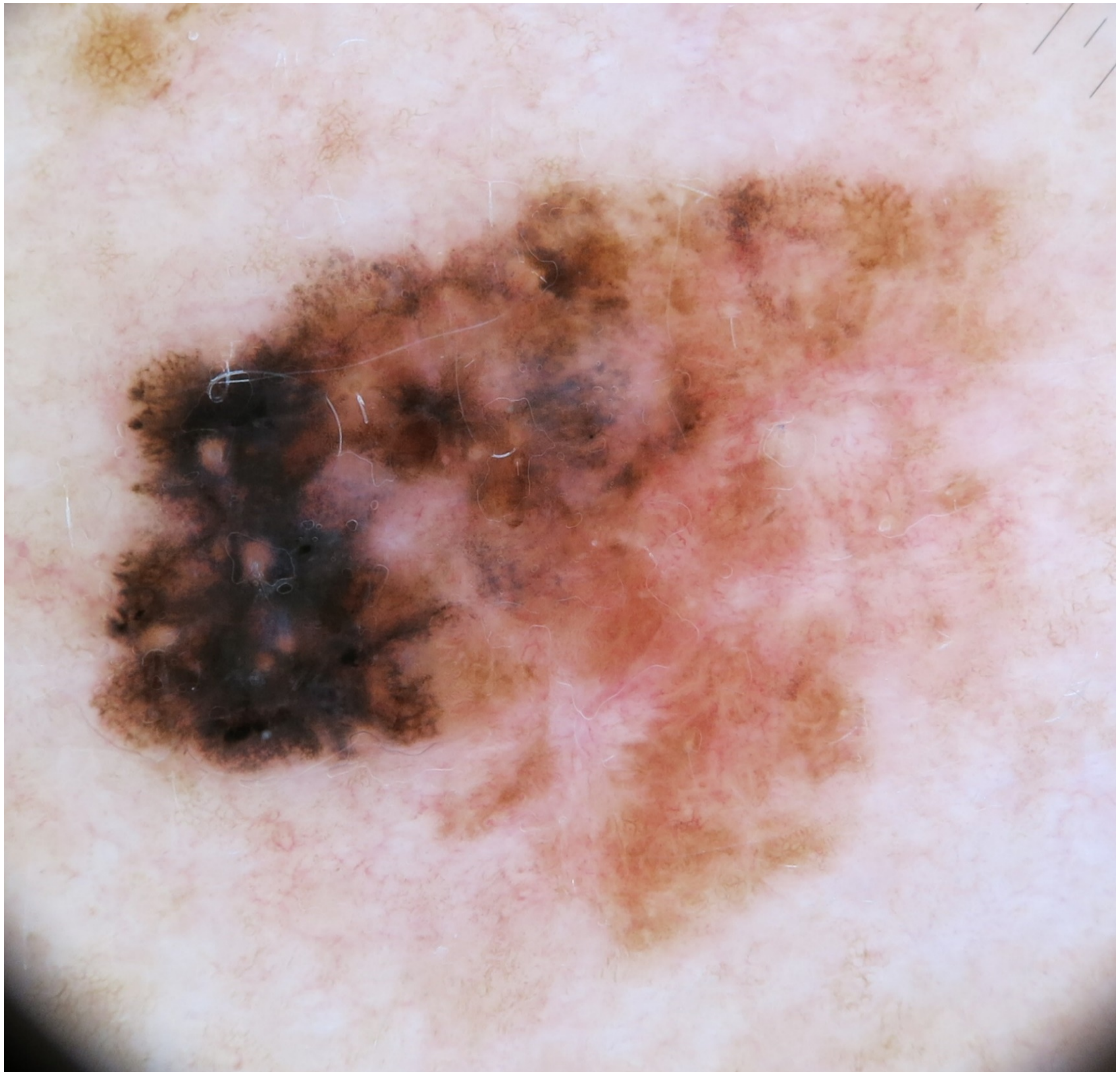

Location: Trunk

Invasive) Breslow interval: 0.1-0.5 mm

Case number 123

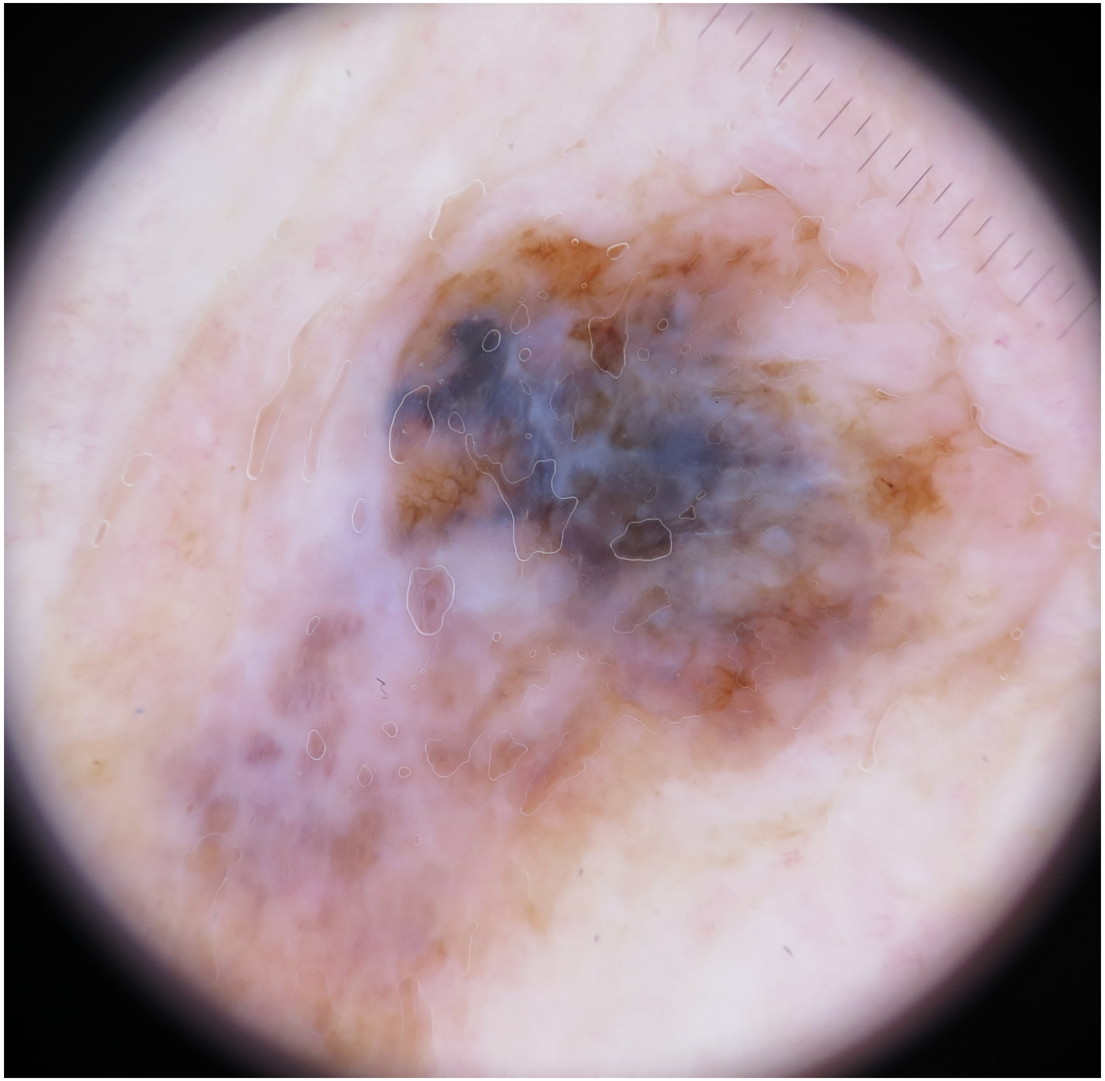

Location: Trunk

Invasive) Breslow interval: 2.1-4.0 mm

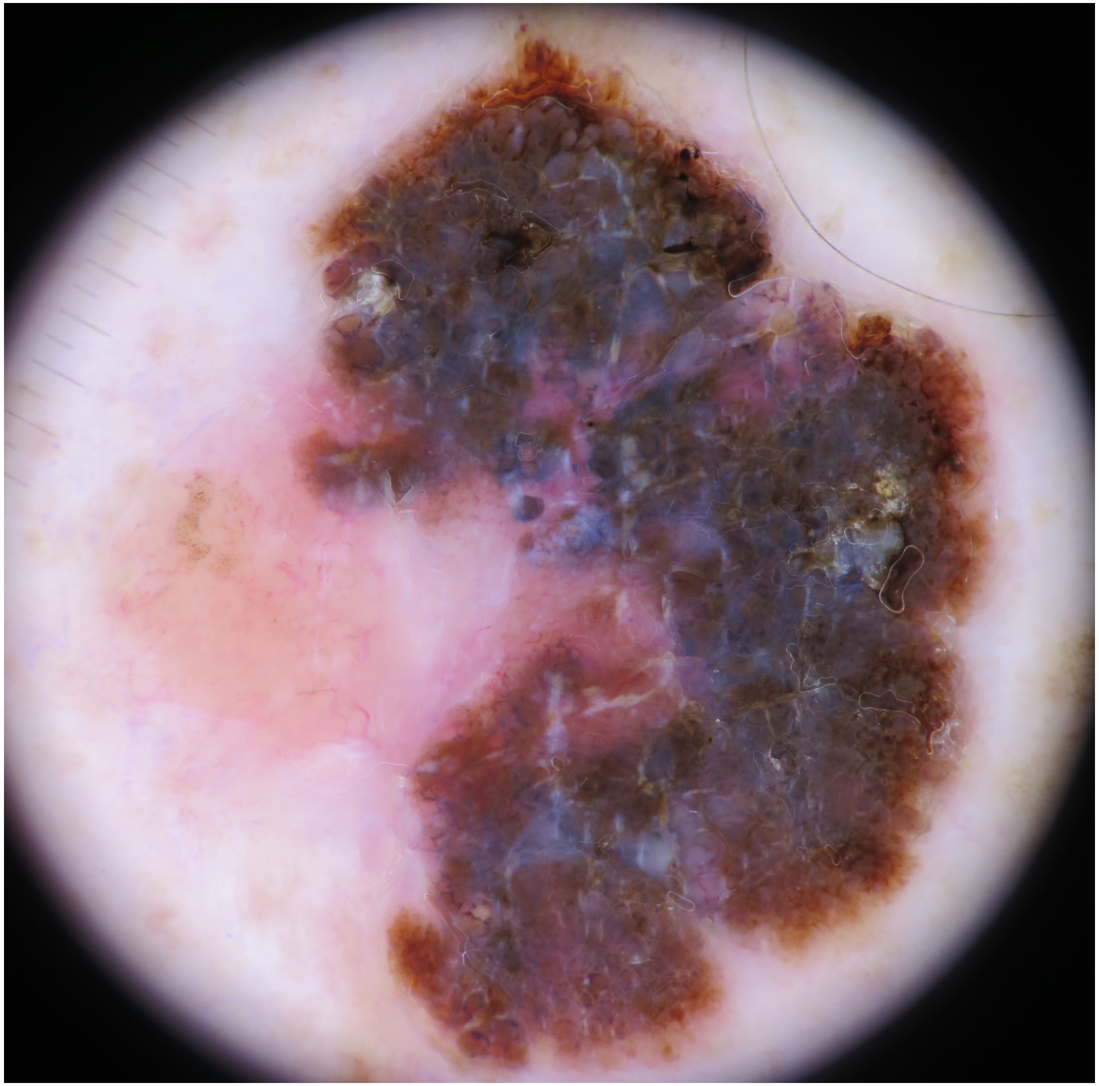

Location: Trunk

Invasive) Breslow interval: 1.1-2.0 mm

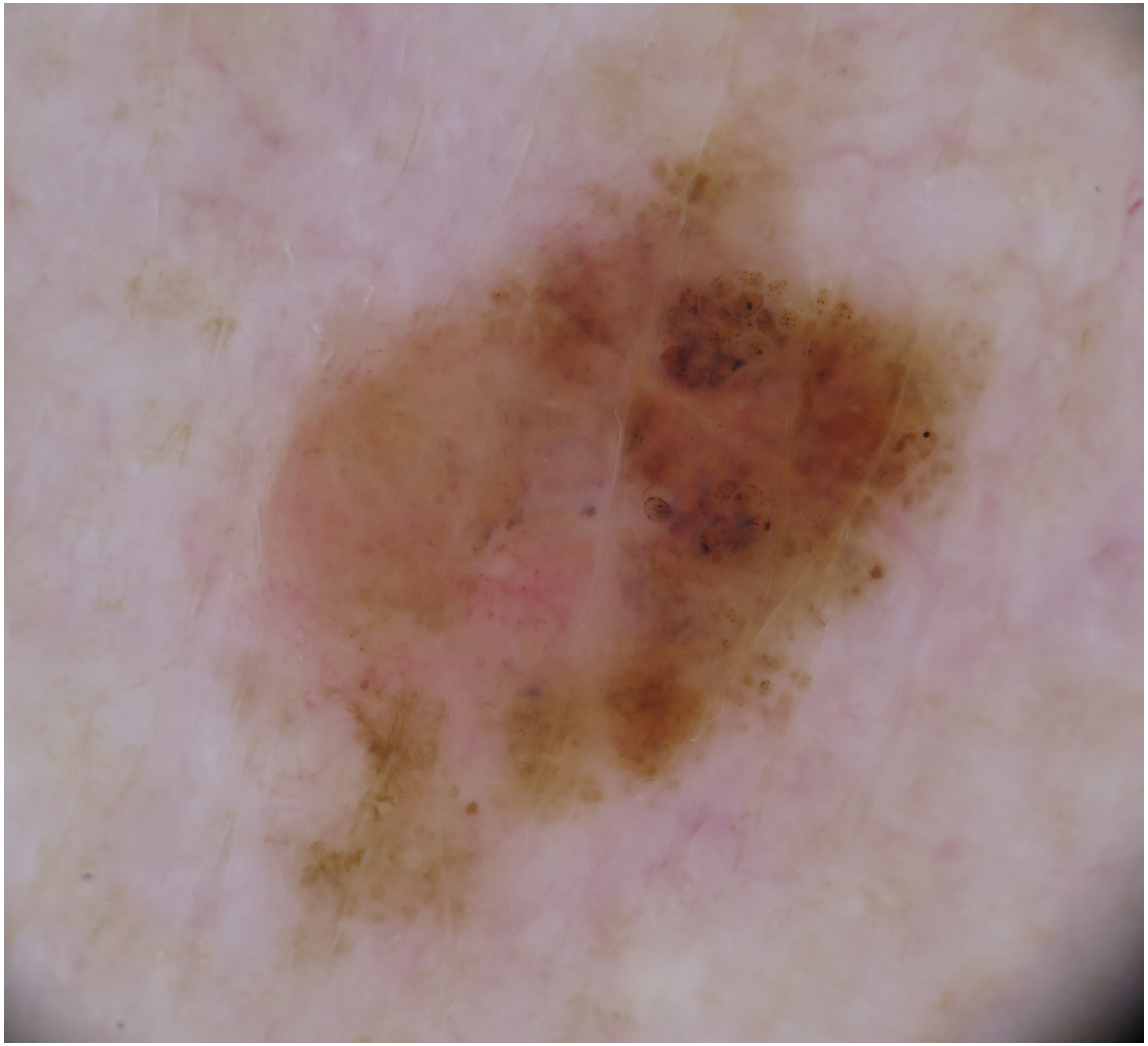

Location: Upper extremity

Invasive) Breslow interval: 0.6-0.8 mm

Case number 126

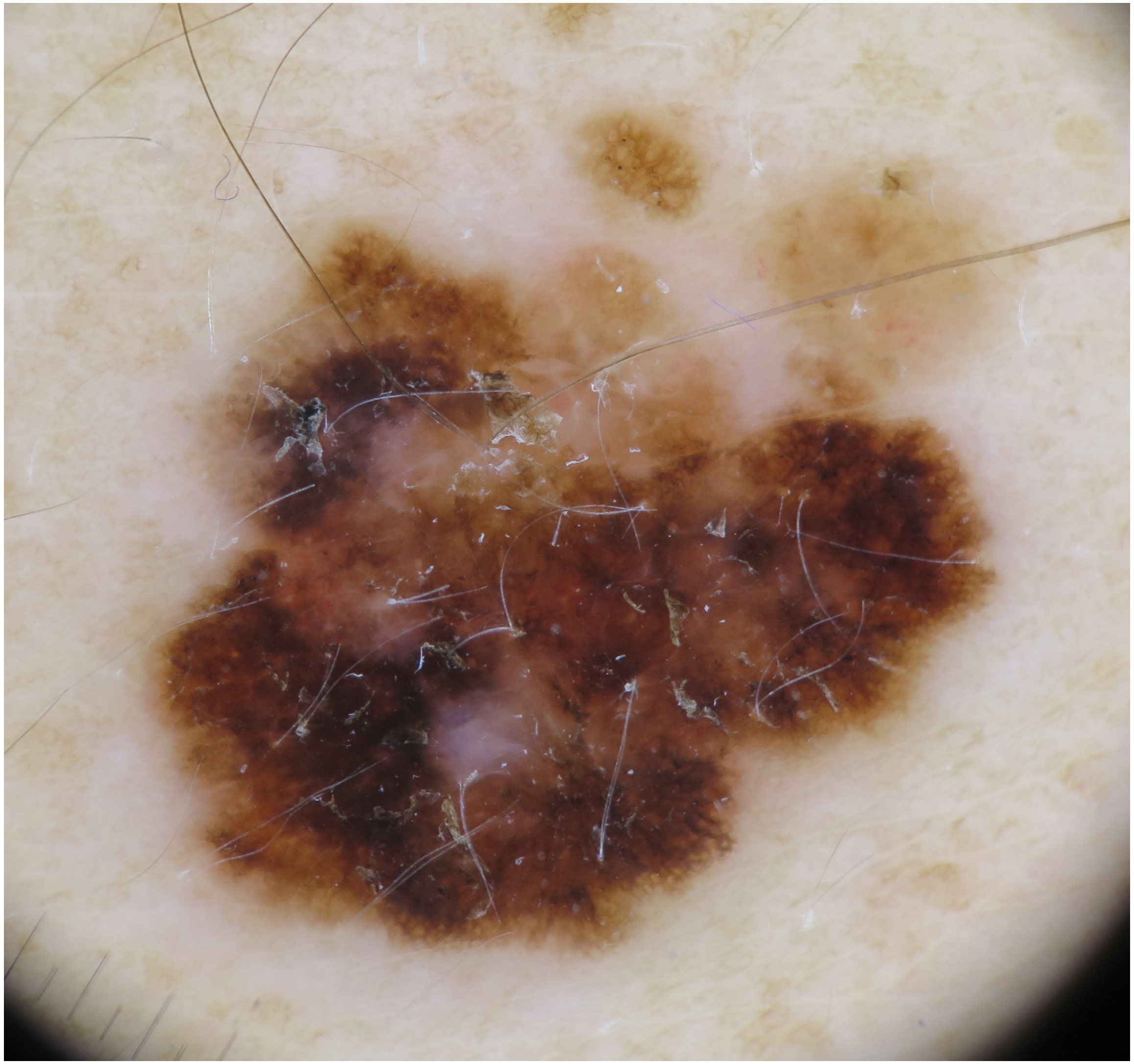

Location: Trunk

Invasive) Breslow interval: 0.1-0.5 mm

Case number 127

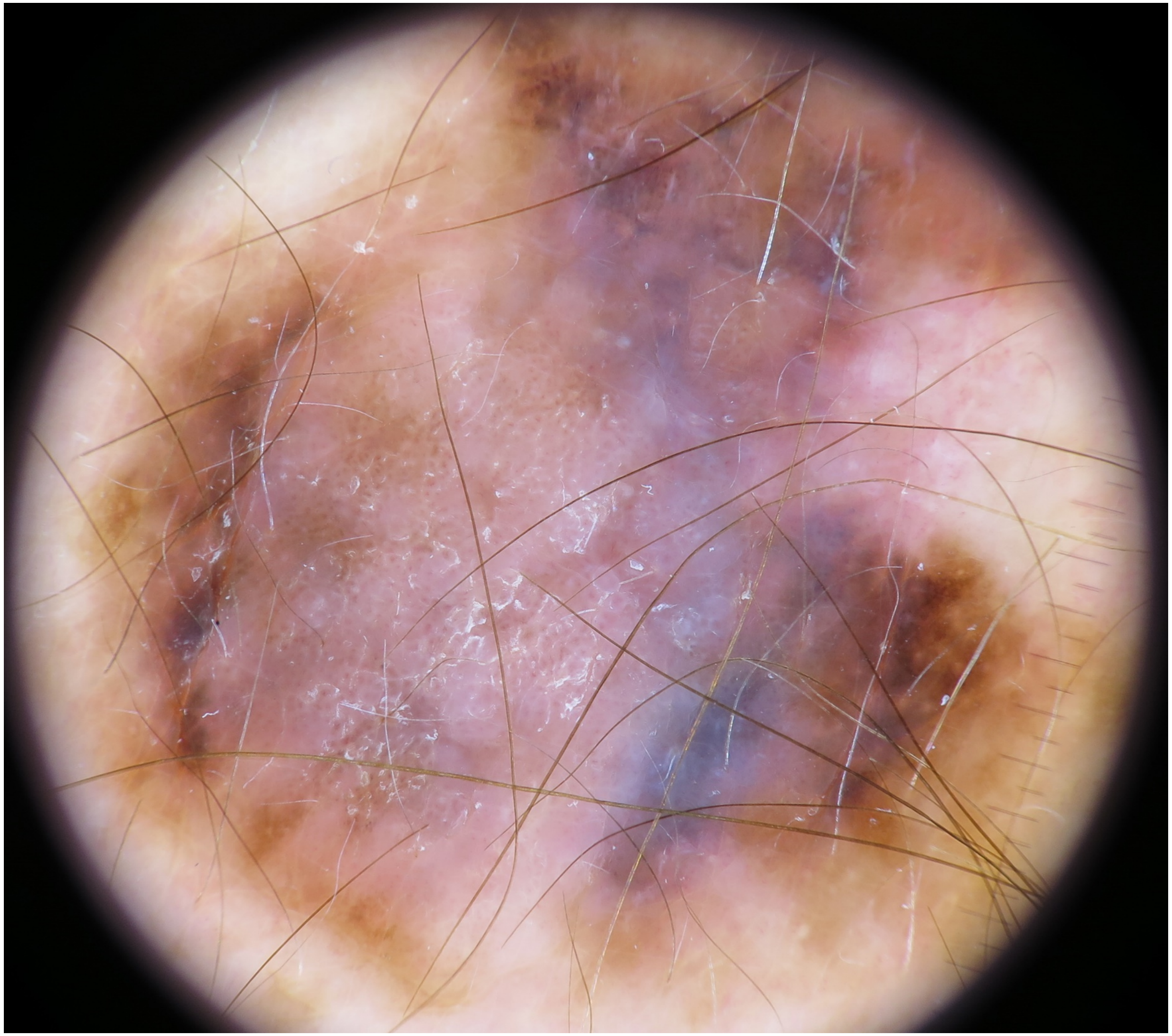

Location: Upper extremity

Invasive) Breslow interval: 2.1-4.0 mm

Case number 128

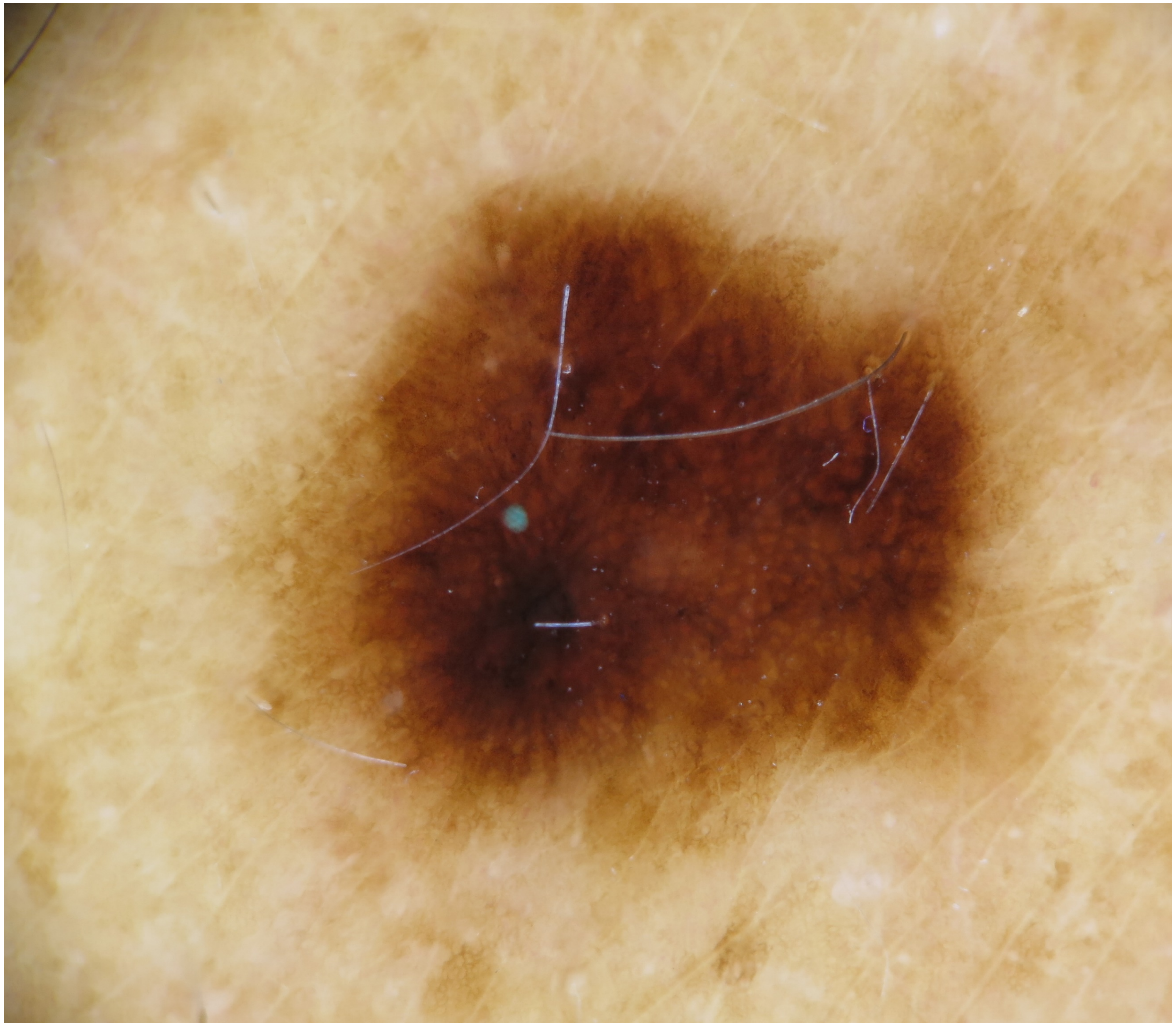

Location: Lower extremity

In situ melanoma

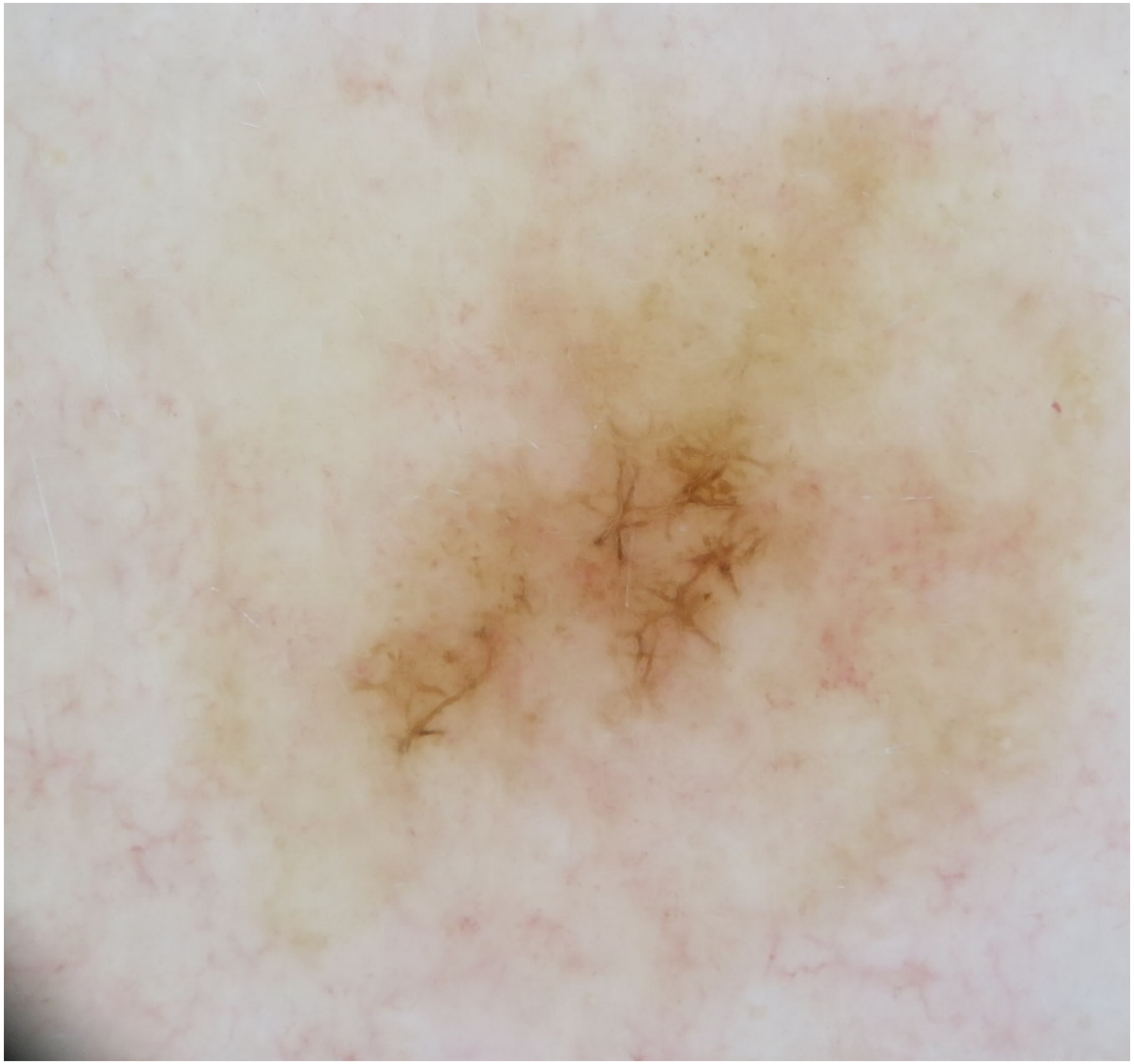

Location: Upper extremity

In situ melanoma

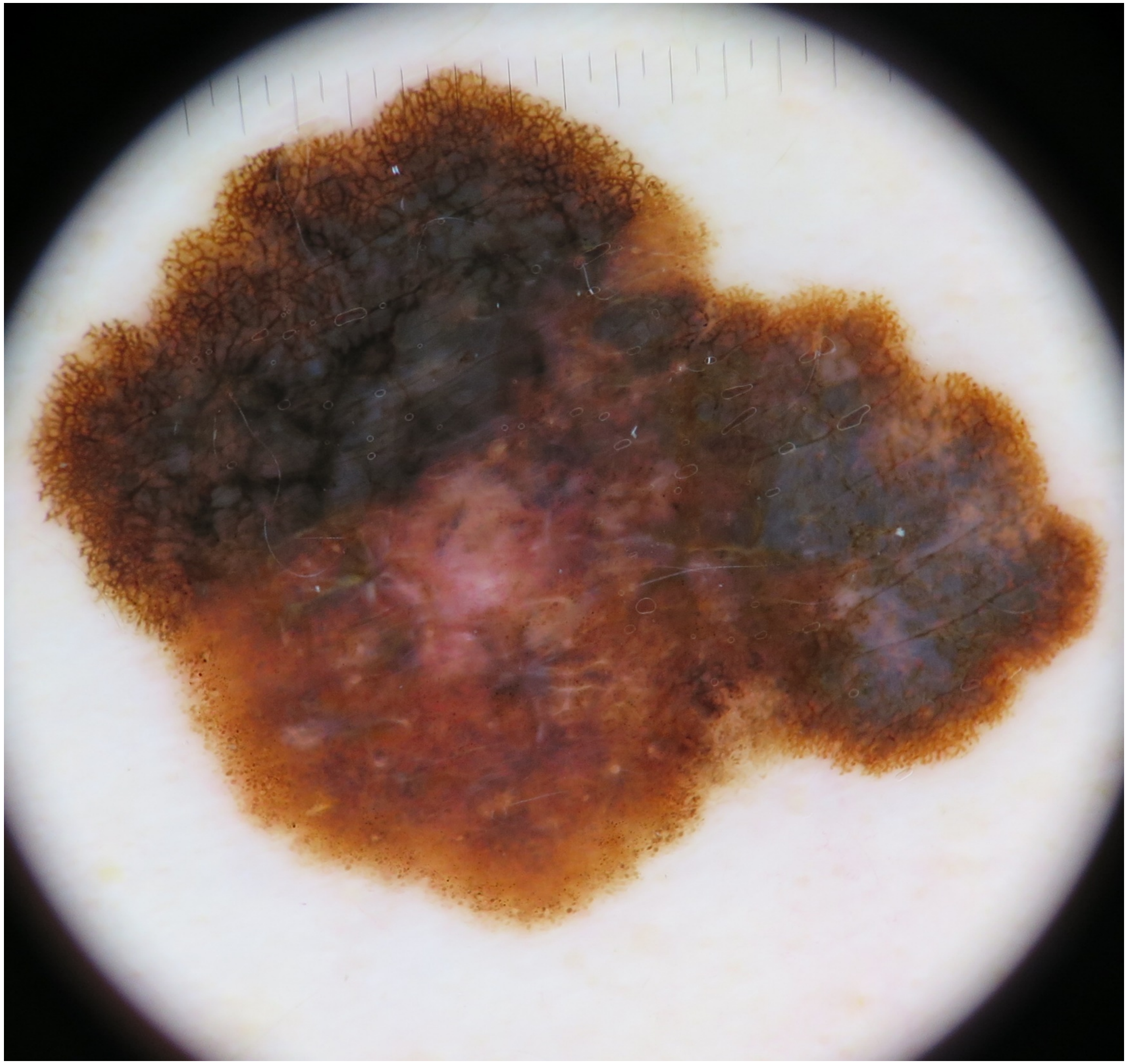

Location: Trunk

Invasive) Breslow interval: 0.6-0.8 mm

Case number 131

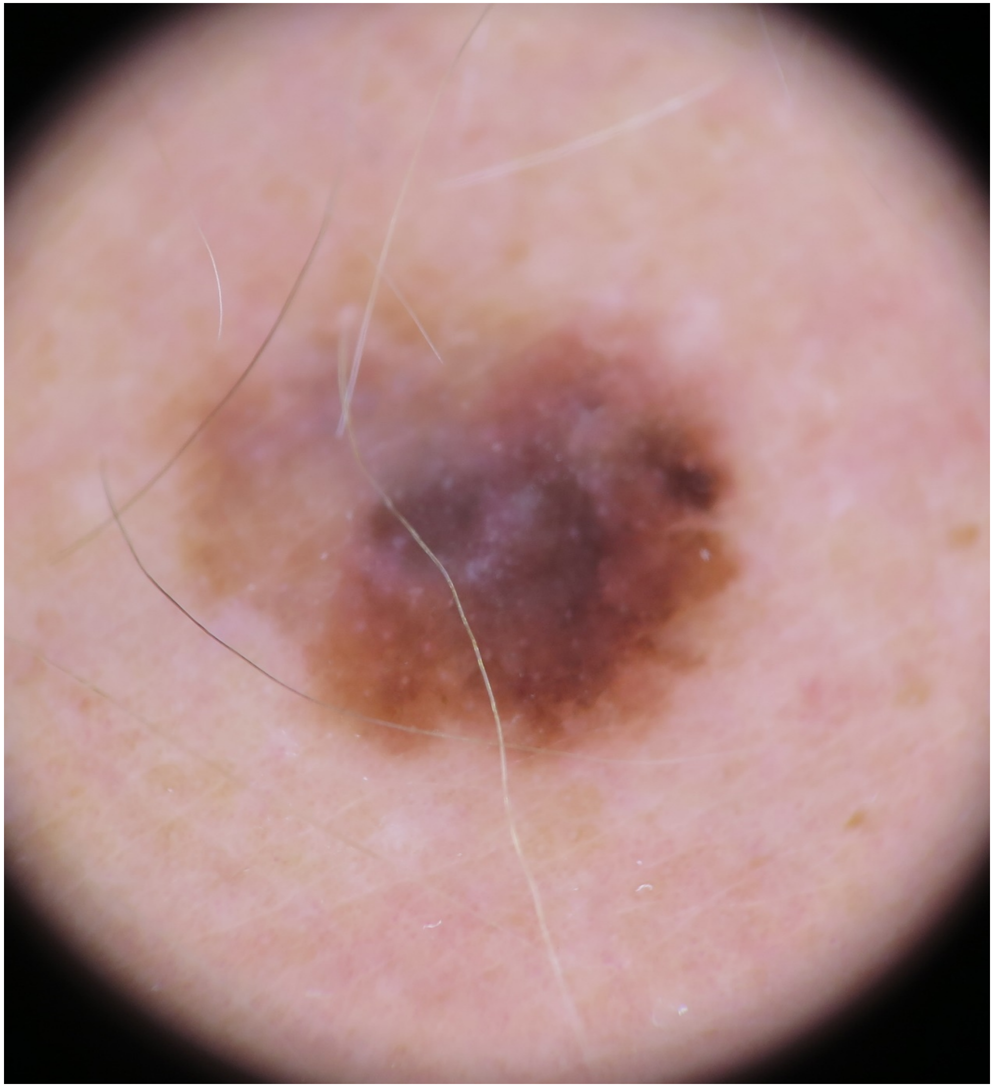

Location: Lower extremity

Invasive) Breslow interval: 0.1-0.5 mm

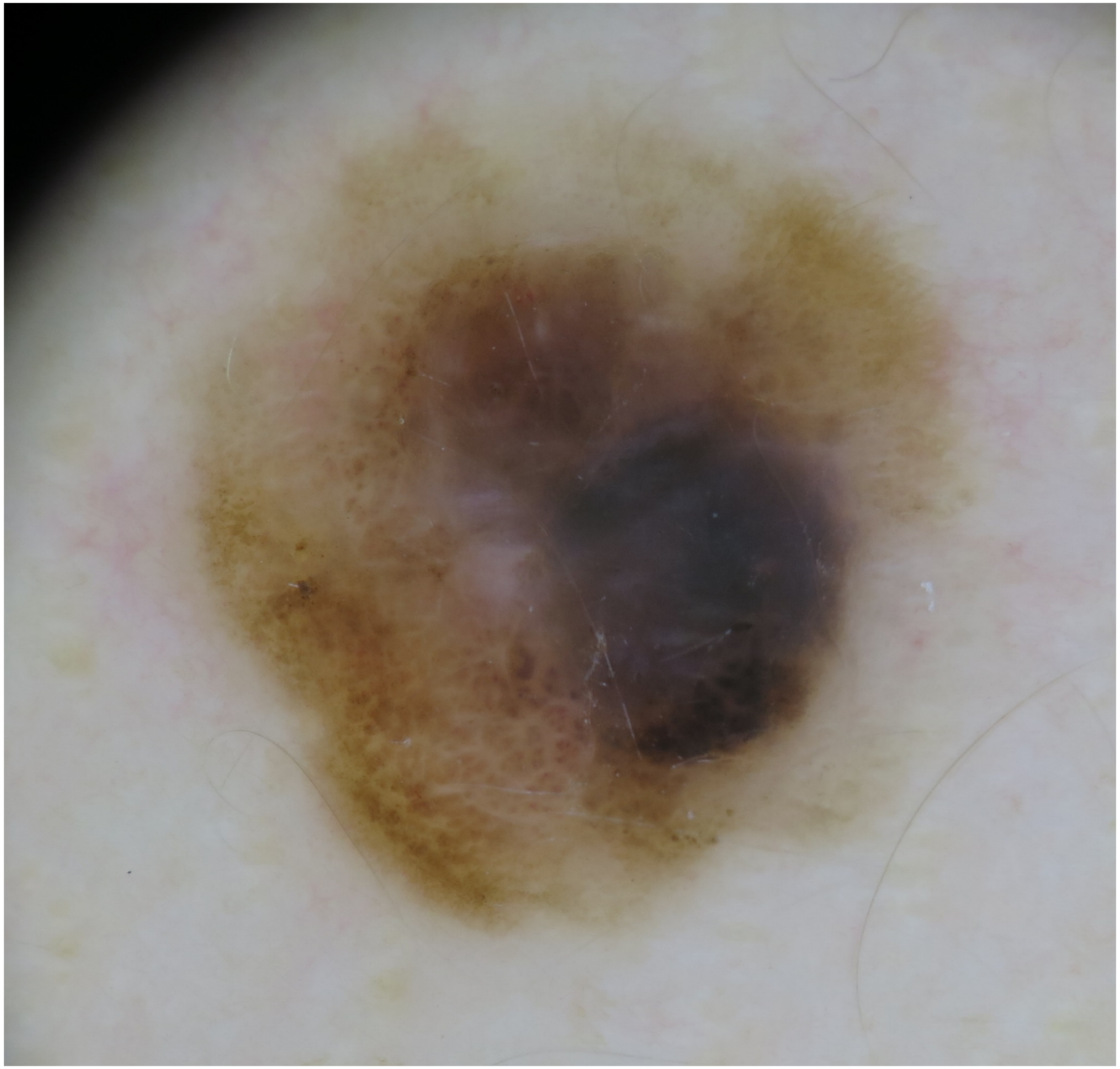

Location: Upper extremity

Invasive) Breslow interval: 1.1-2.0 mm

Case number 133

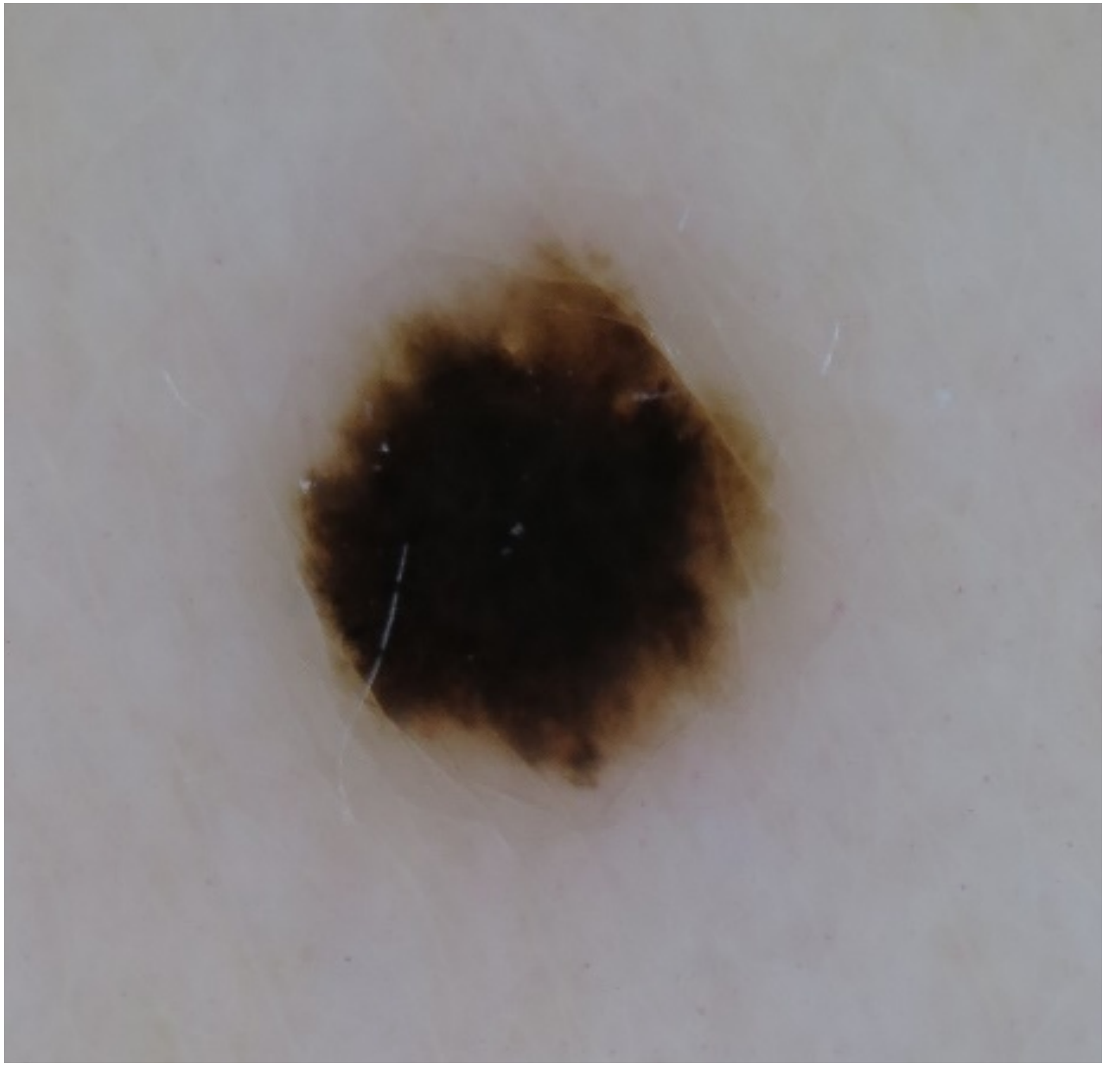

Location: Lower extremity

Invasive) Breslow interval: 0.1-0.5 mm

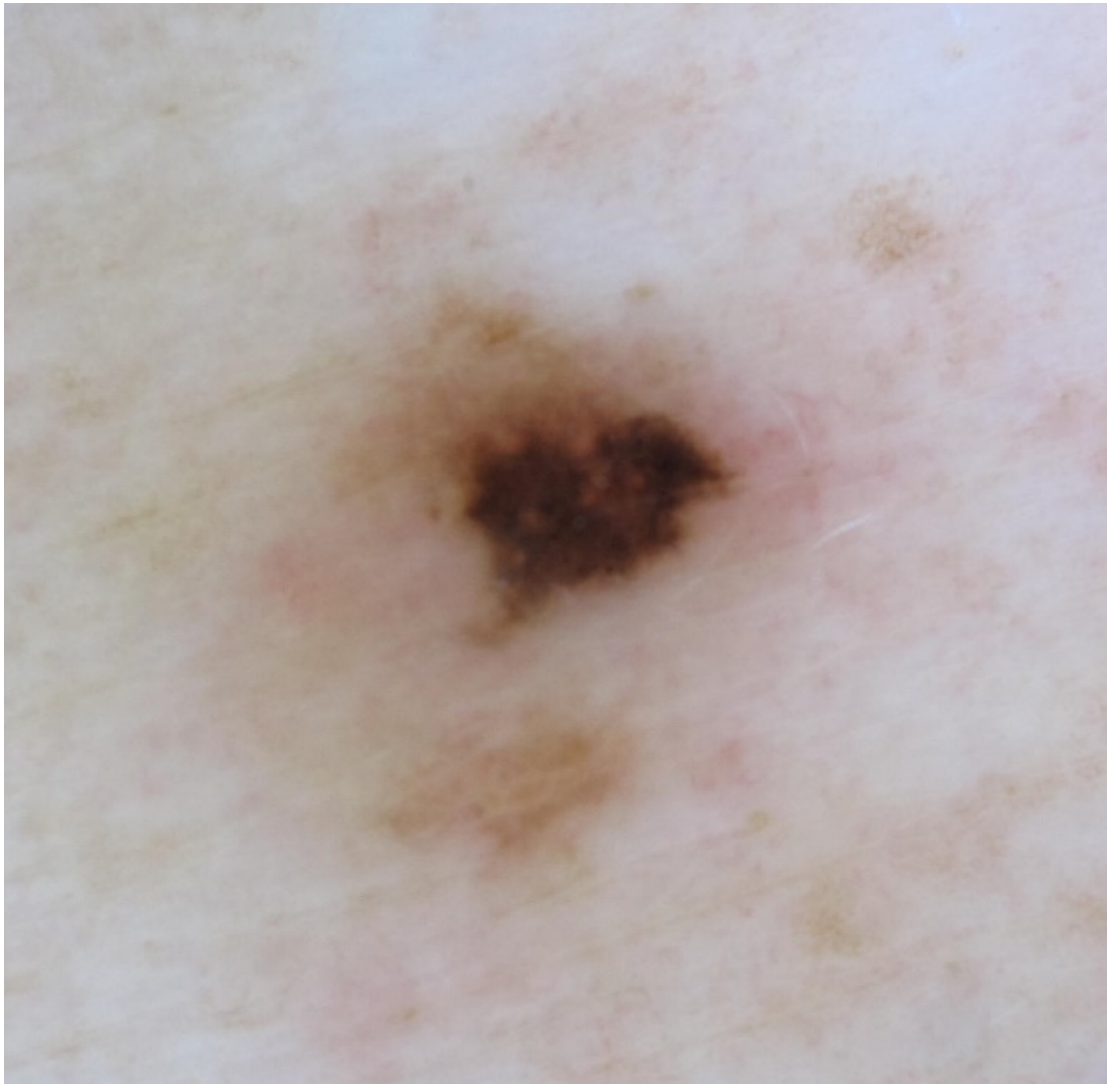

Location: Trunk

In situ melanoma

Case number 135

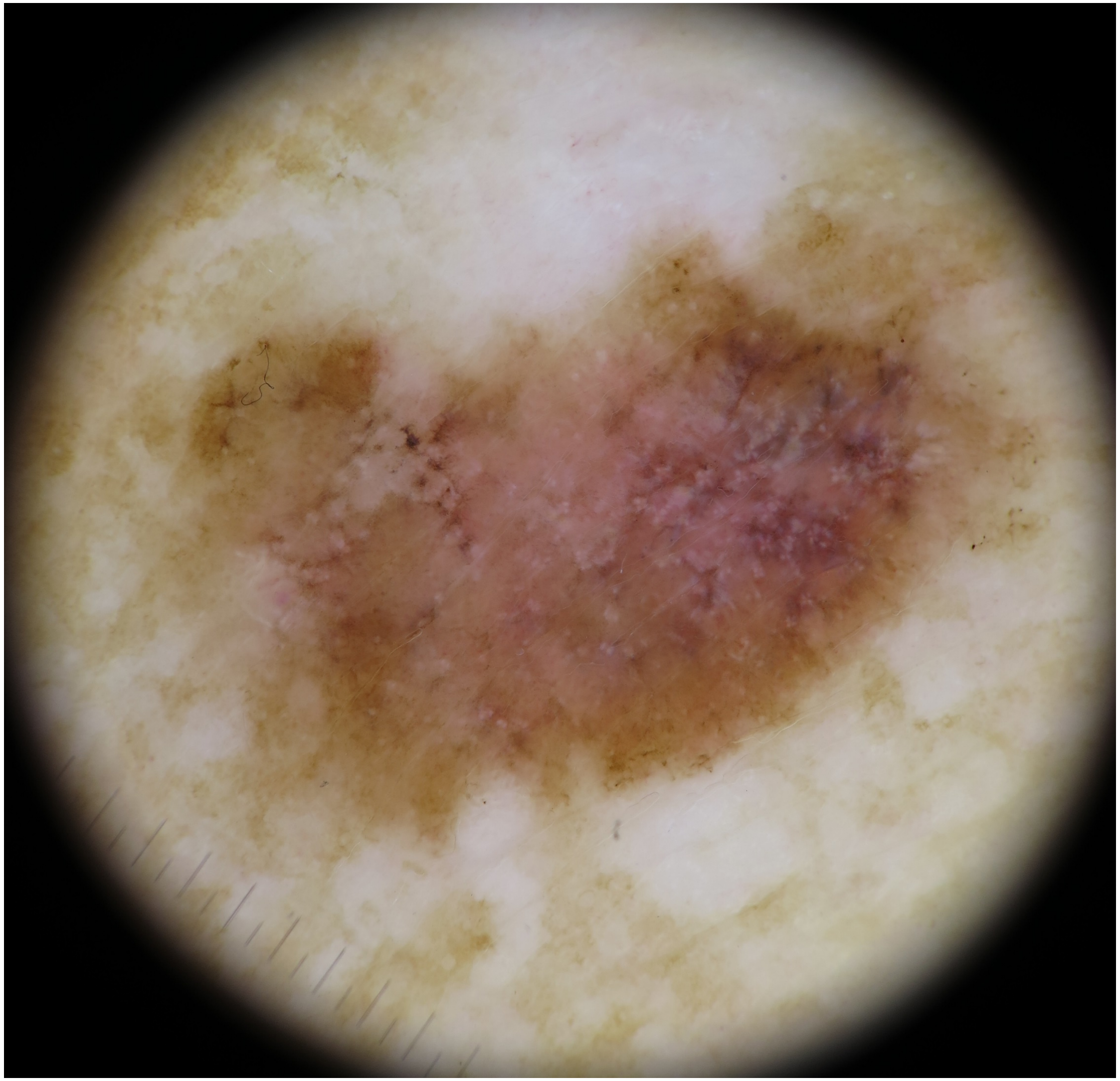

Location: Lower extremity

Invasive) Breslow interval: 0.6-0.8 mm

Case number 136

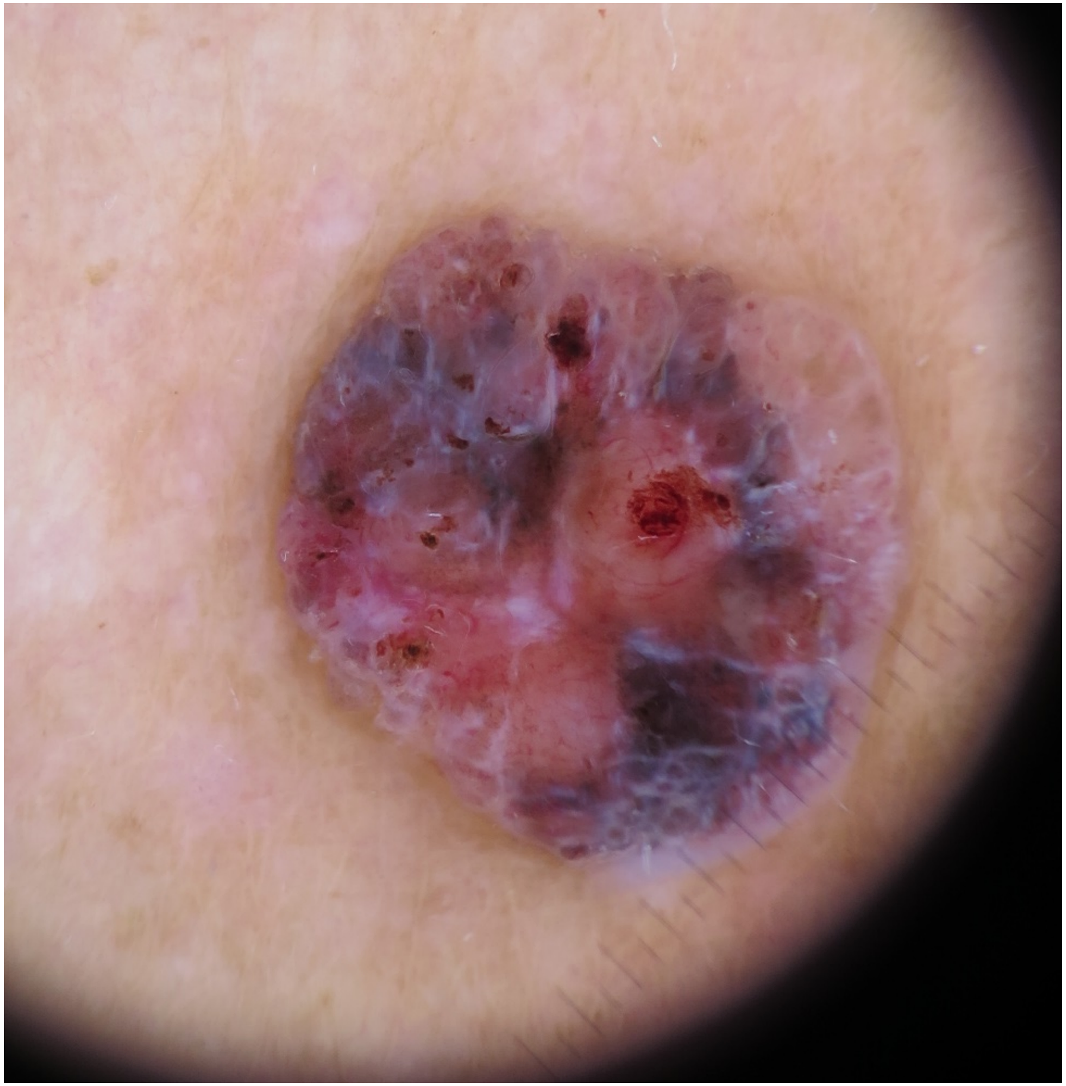

Location: Lower extremity

Invasive) Breslow interval: > 4mm

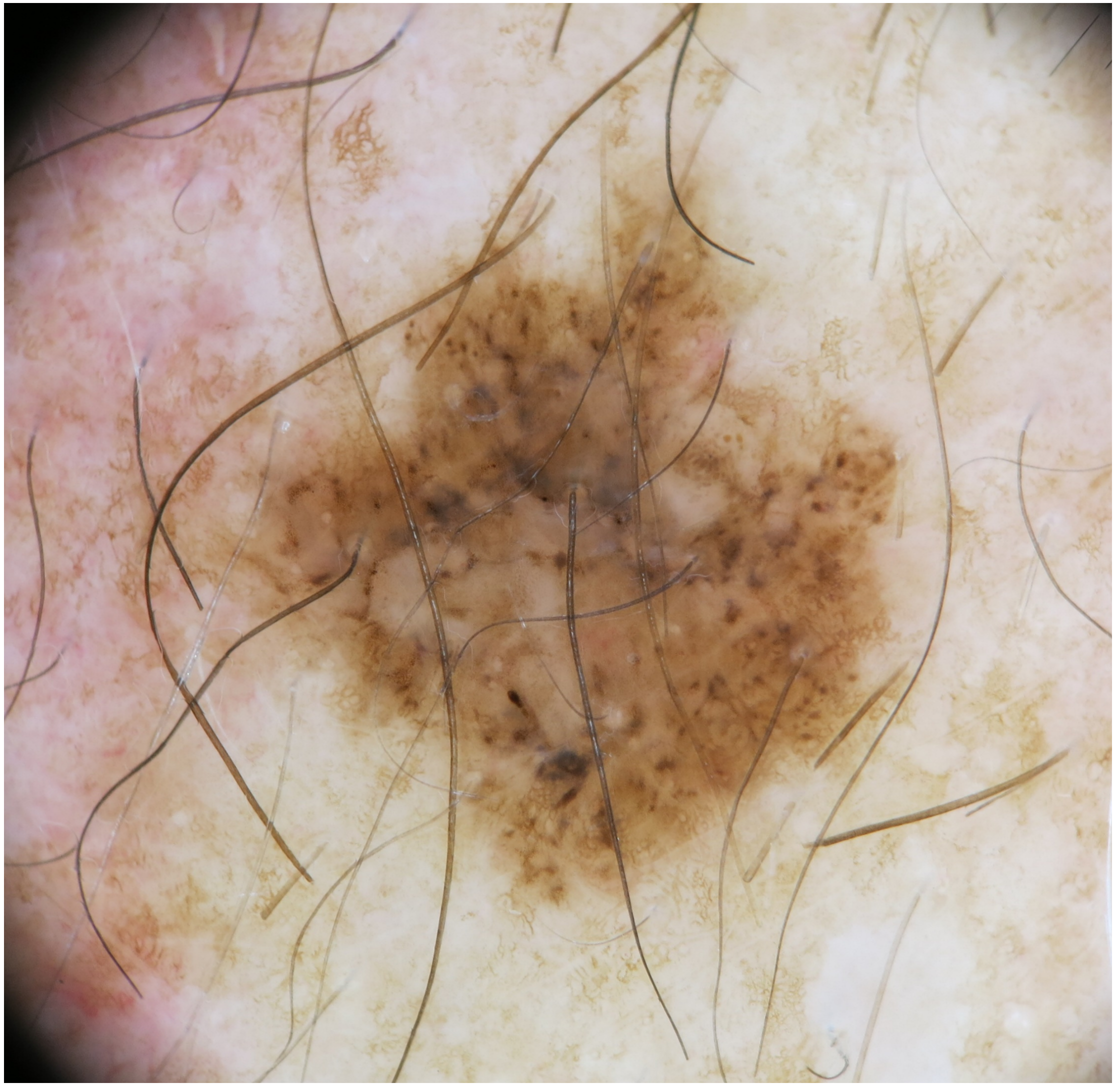

Location: Upper extremity

In situ melanoma

Case number 138

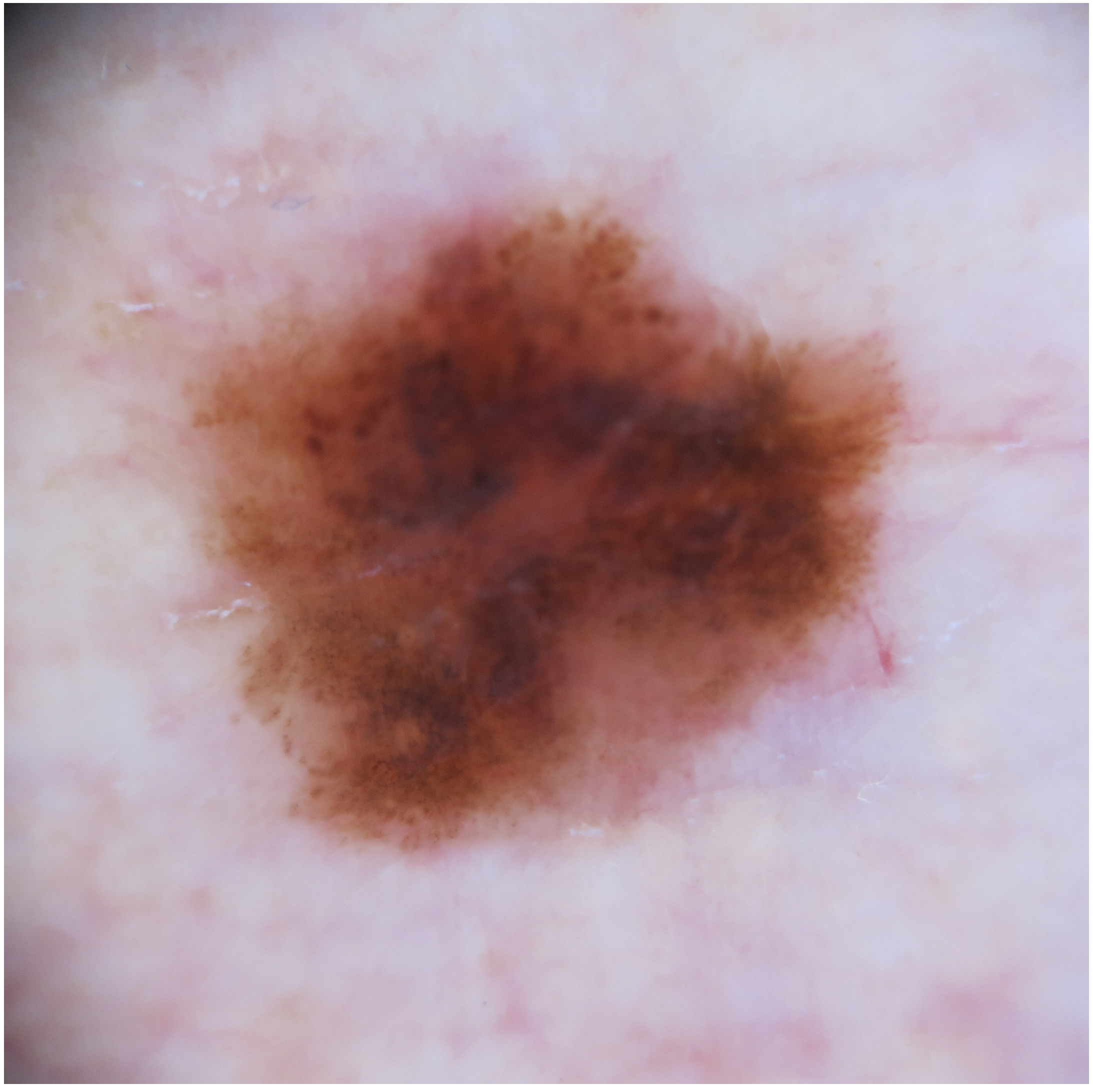

Location: Neck

Invasive) Breslow interval: 0.6-0.8 mm

Case number 139

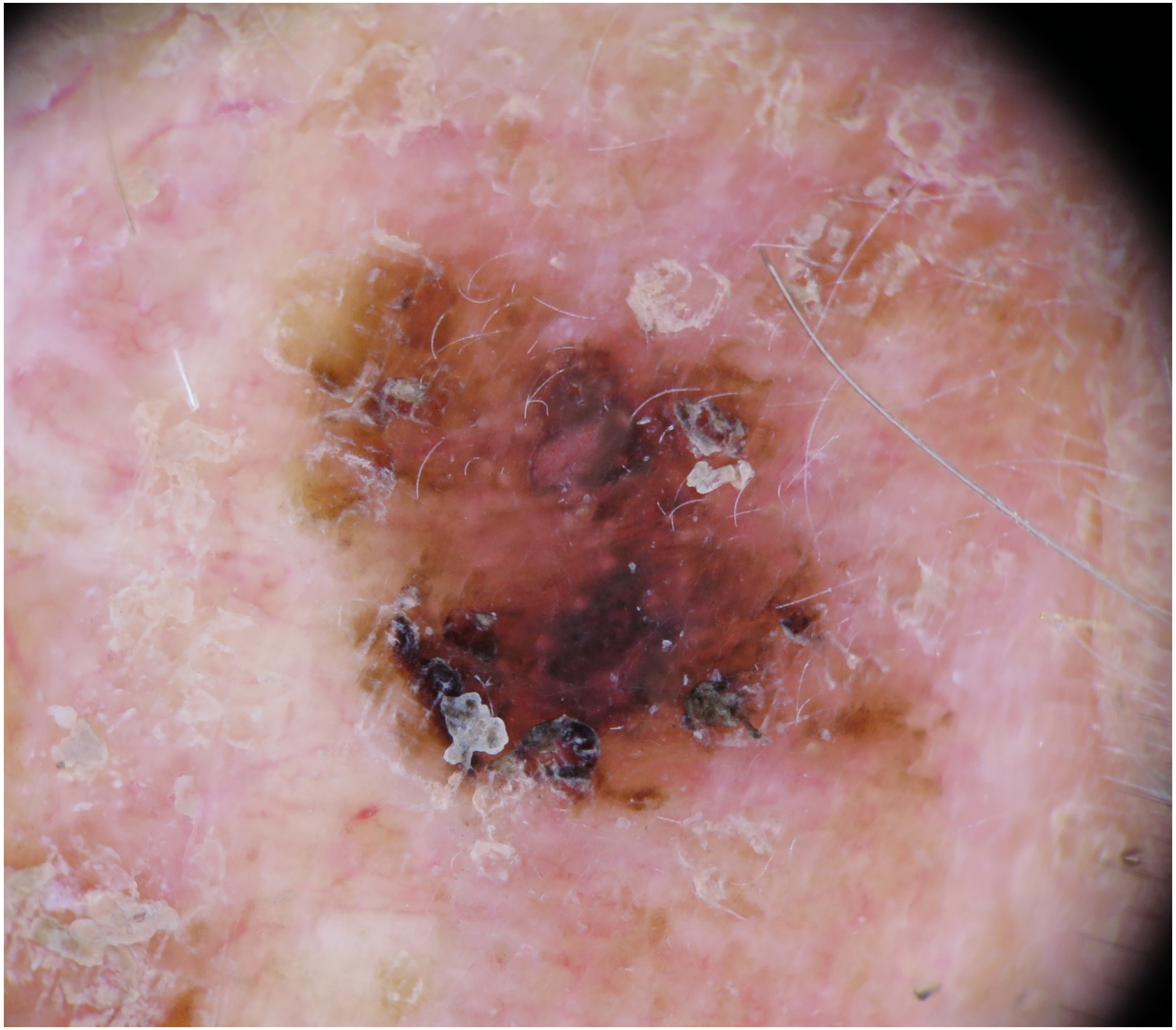

Location: Face

Invasive) Breslow interval: 0.1-0.5 mm

Case number 140

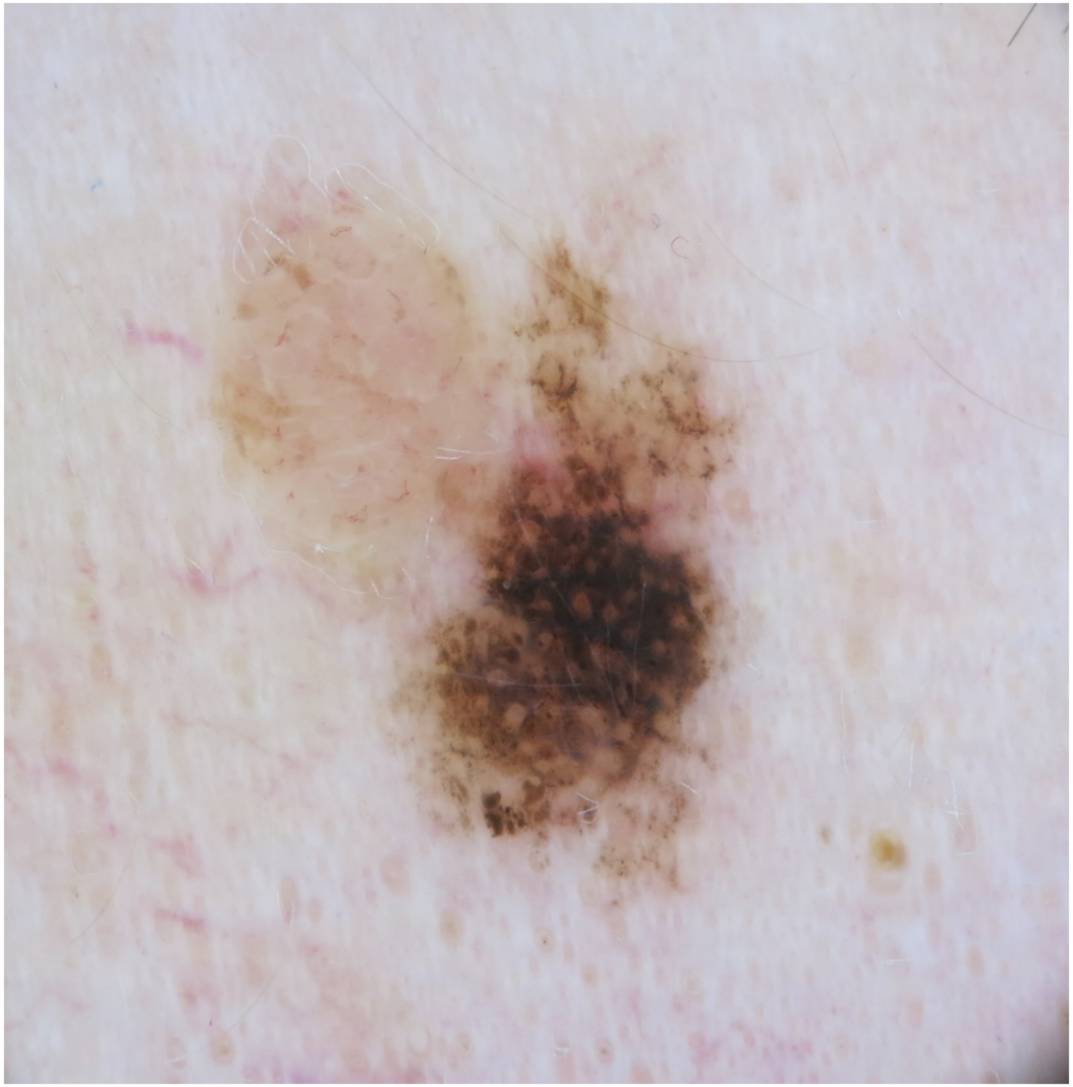

Location: Face

Invasive) Breslow interval: 0.6-0.8 mm

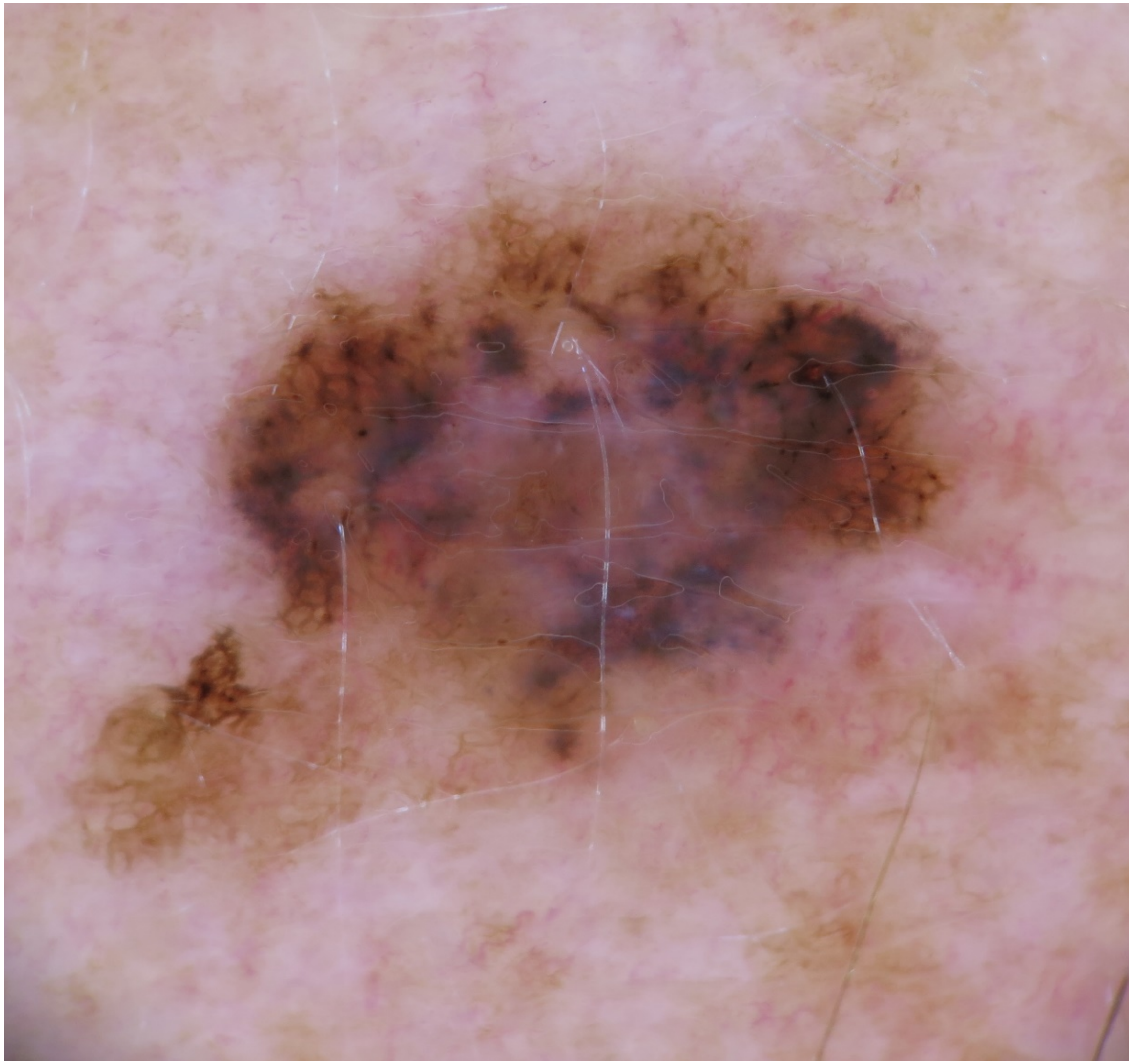

Location: Trunk

In situ melanoma

Case number 142

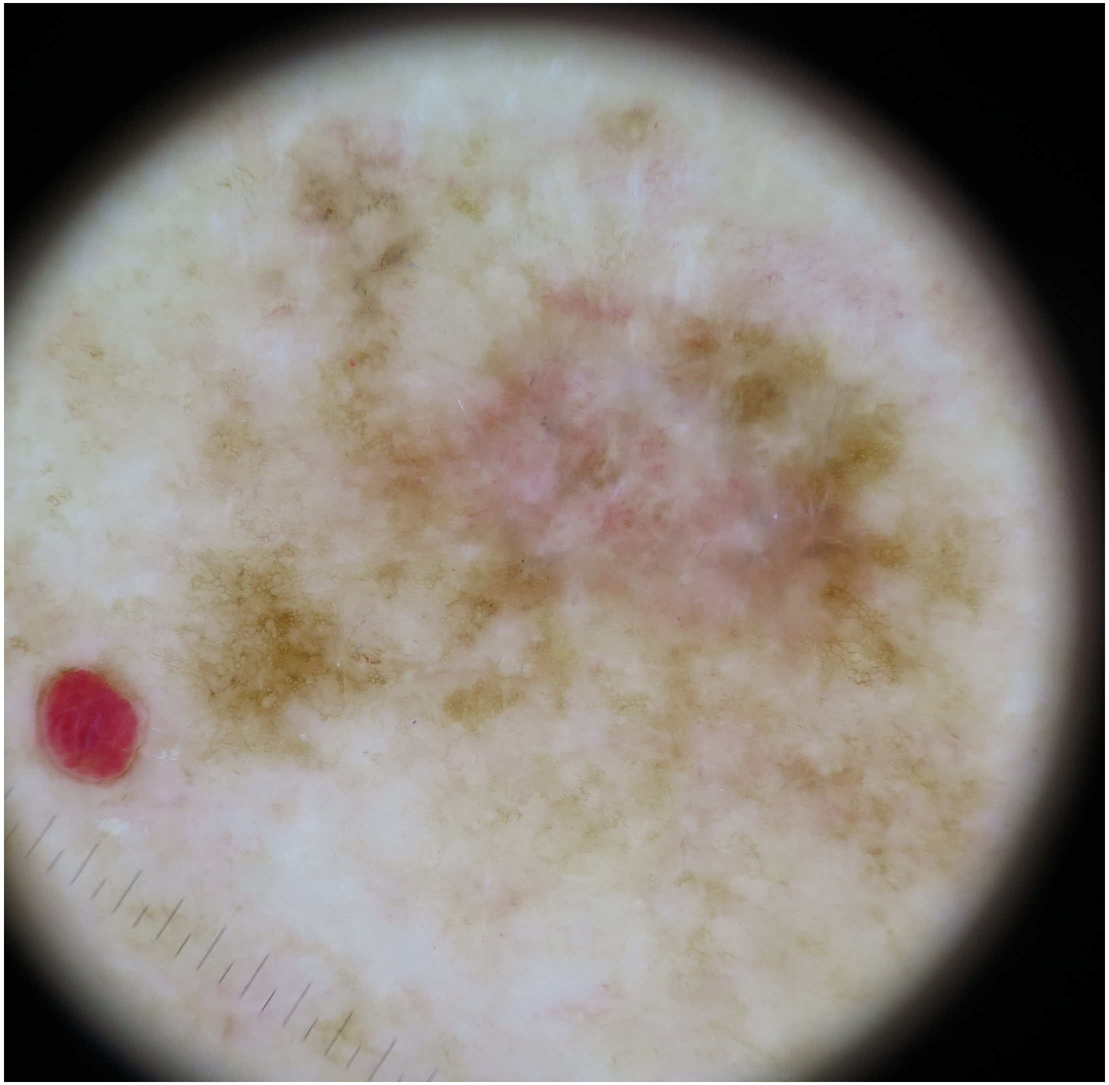

Location: Trunk

Invasive) Breslow interval: 0.1-0.5 mm

Case number 143

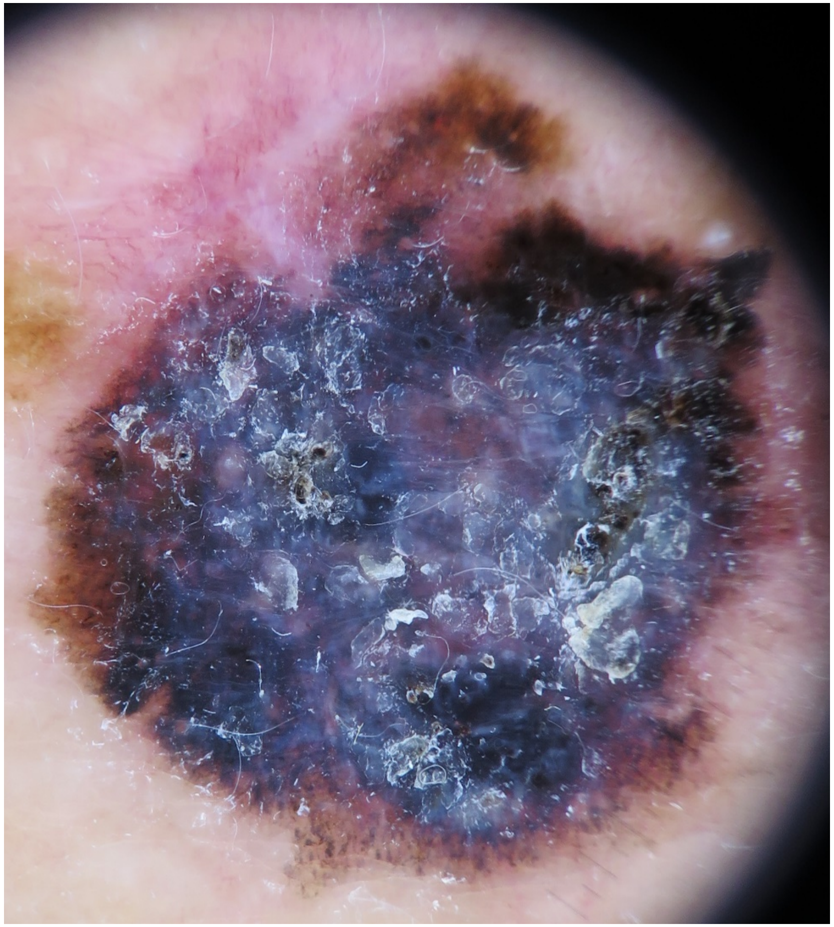

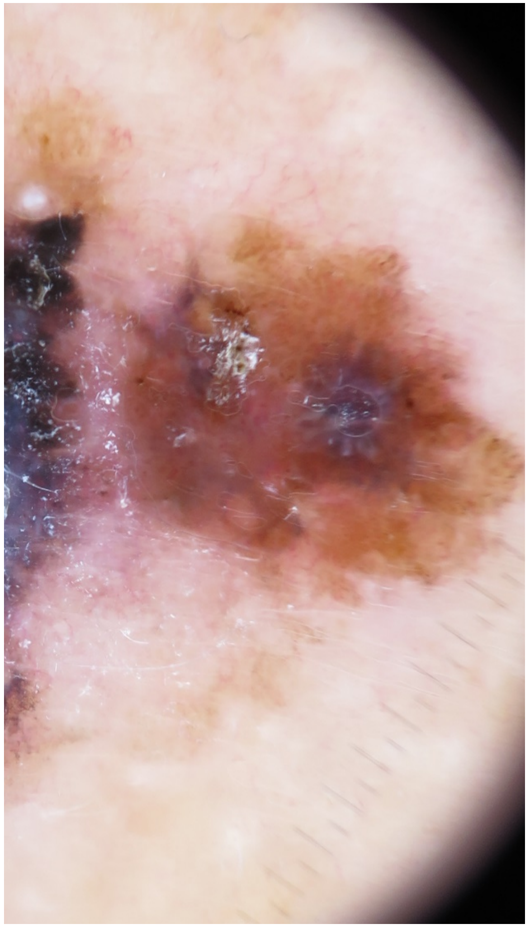

Location: Upper extremity

Invasive) Breslow interval: 0.9-1.0 mm

Case number 144

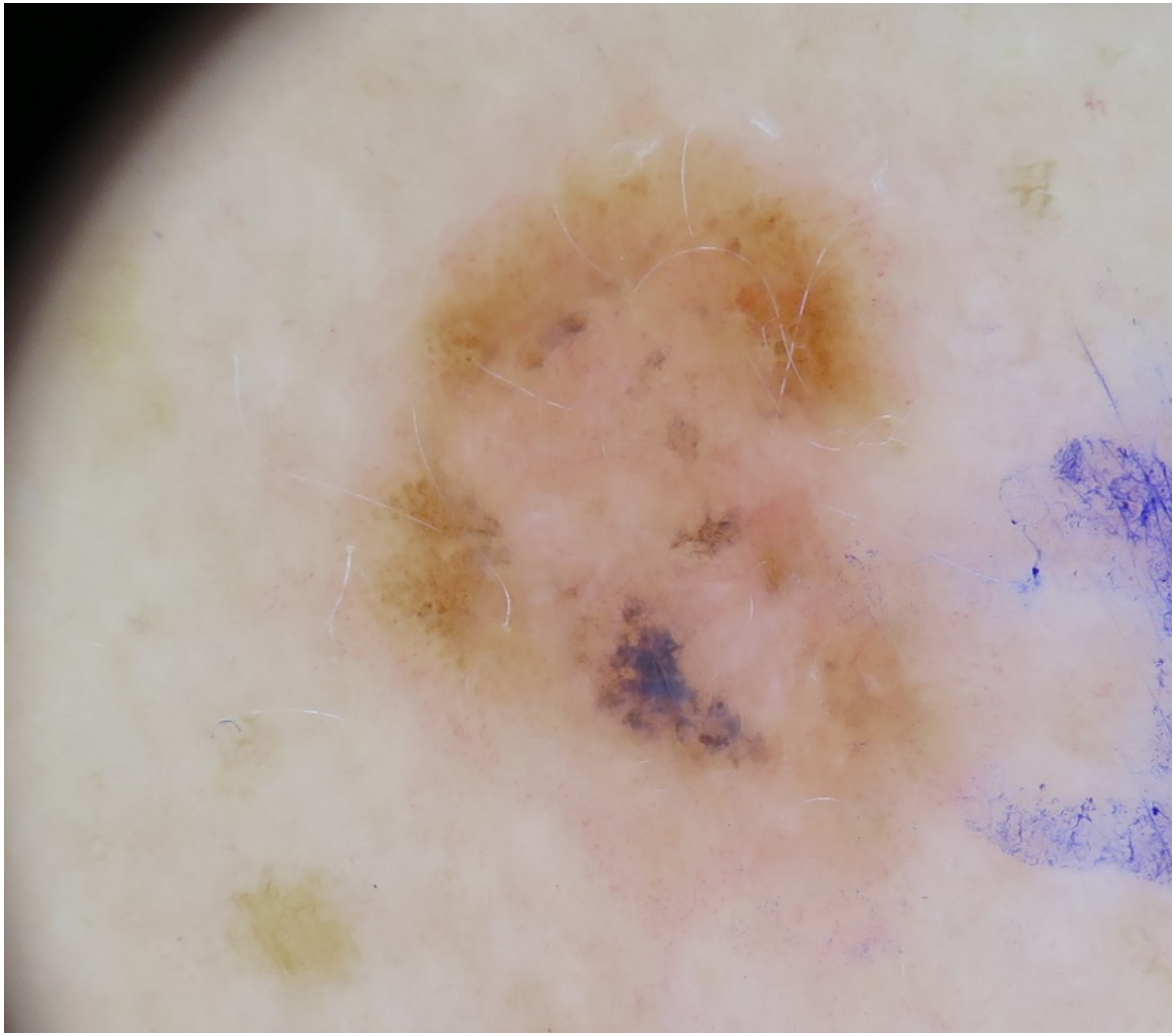

Location: Trunk

Invasive) Breslow interval: 0.9-1.0 mm

Case number 145

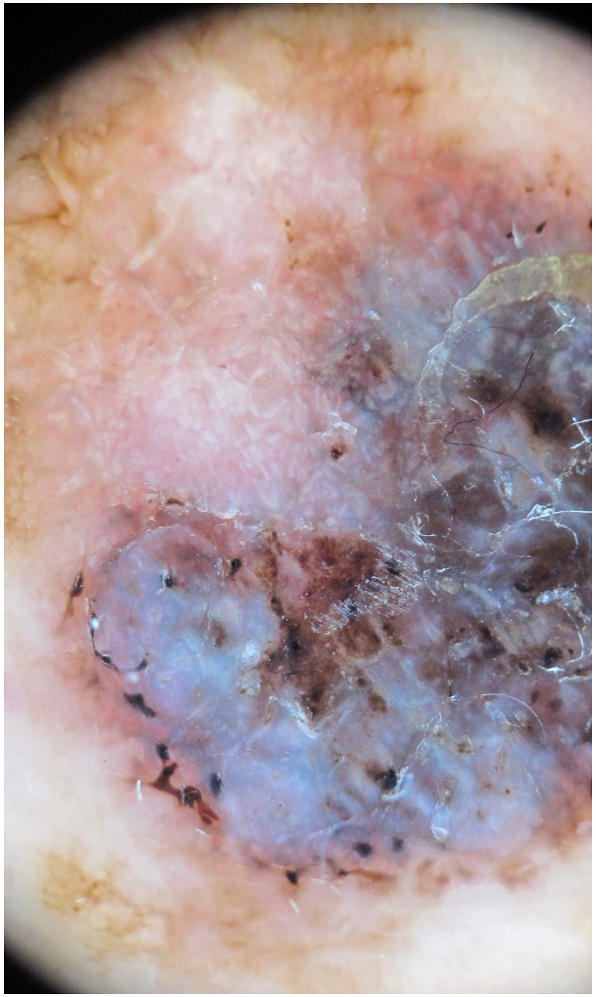

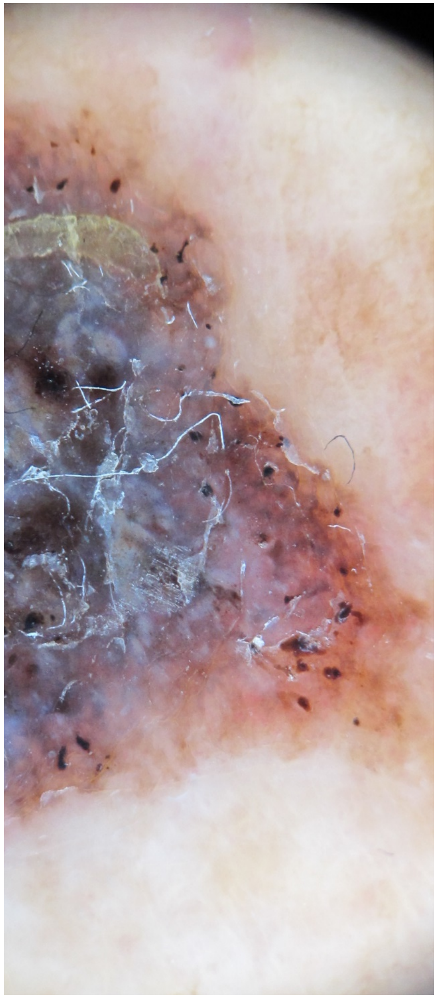

Location: Lower extremity

Invasive) Breslow interval: 0.9-1.0 mm

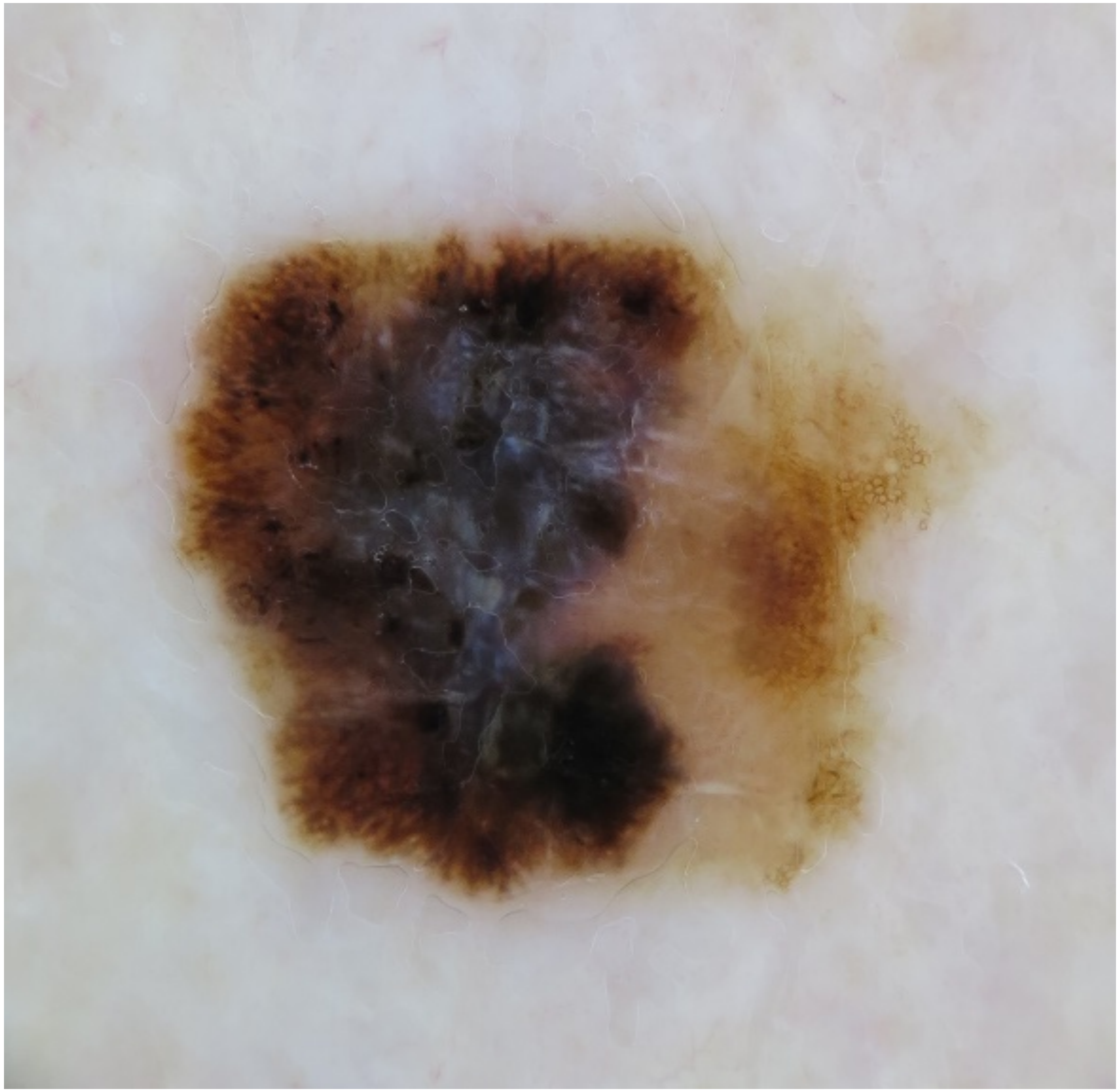

Location: Lower extremity

Invasive) Breslow interval: 0.6-0.8 mm

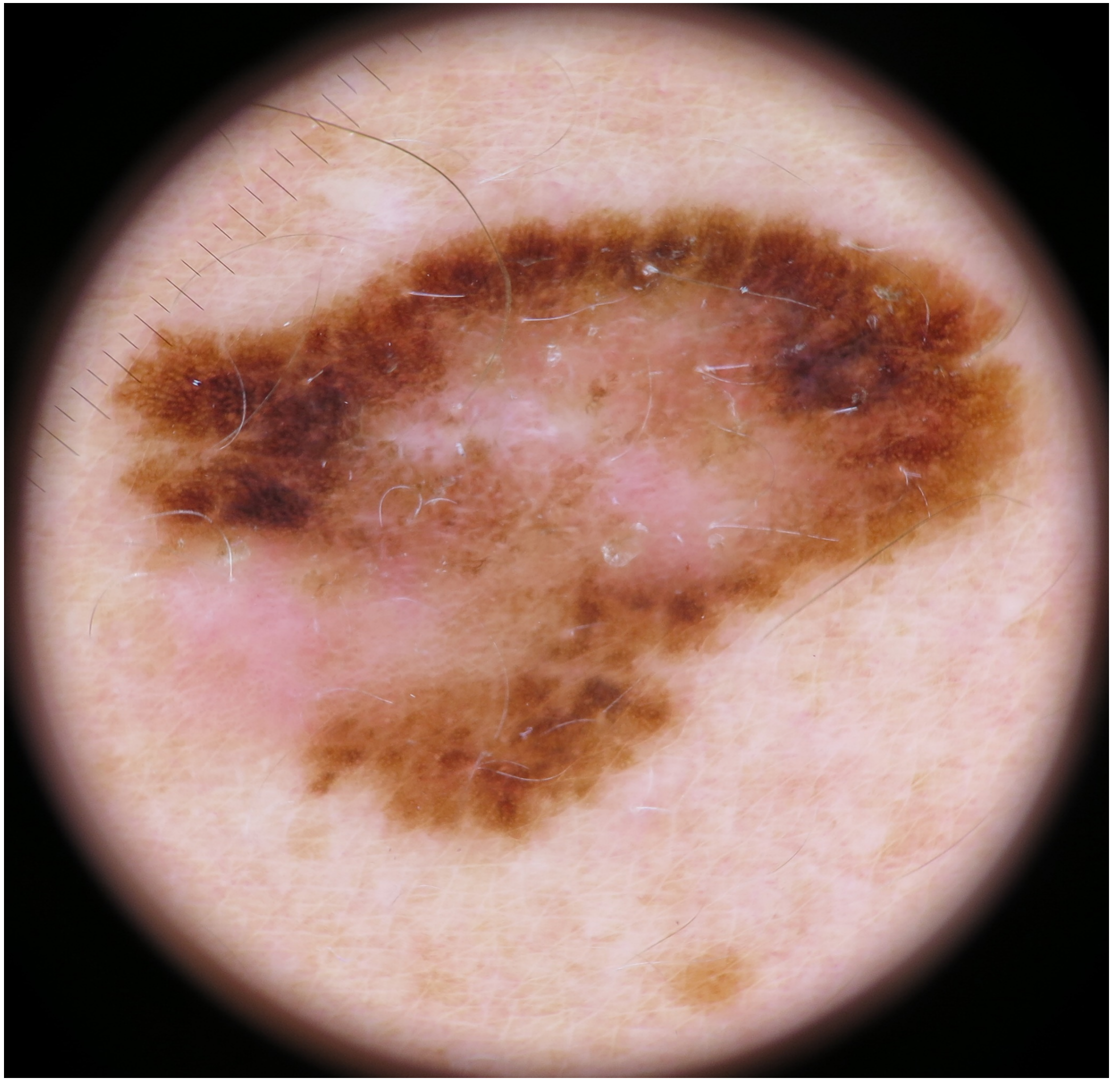

Location: Trunk

In situ melanoma

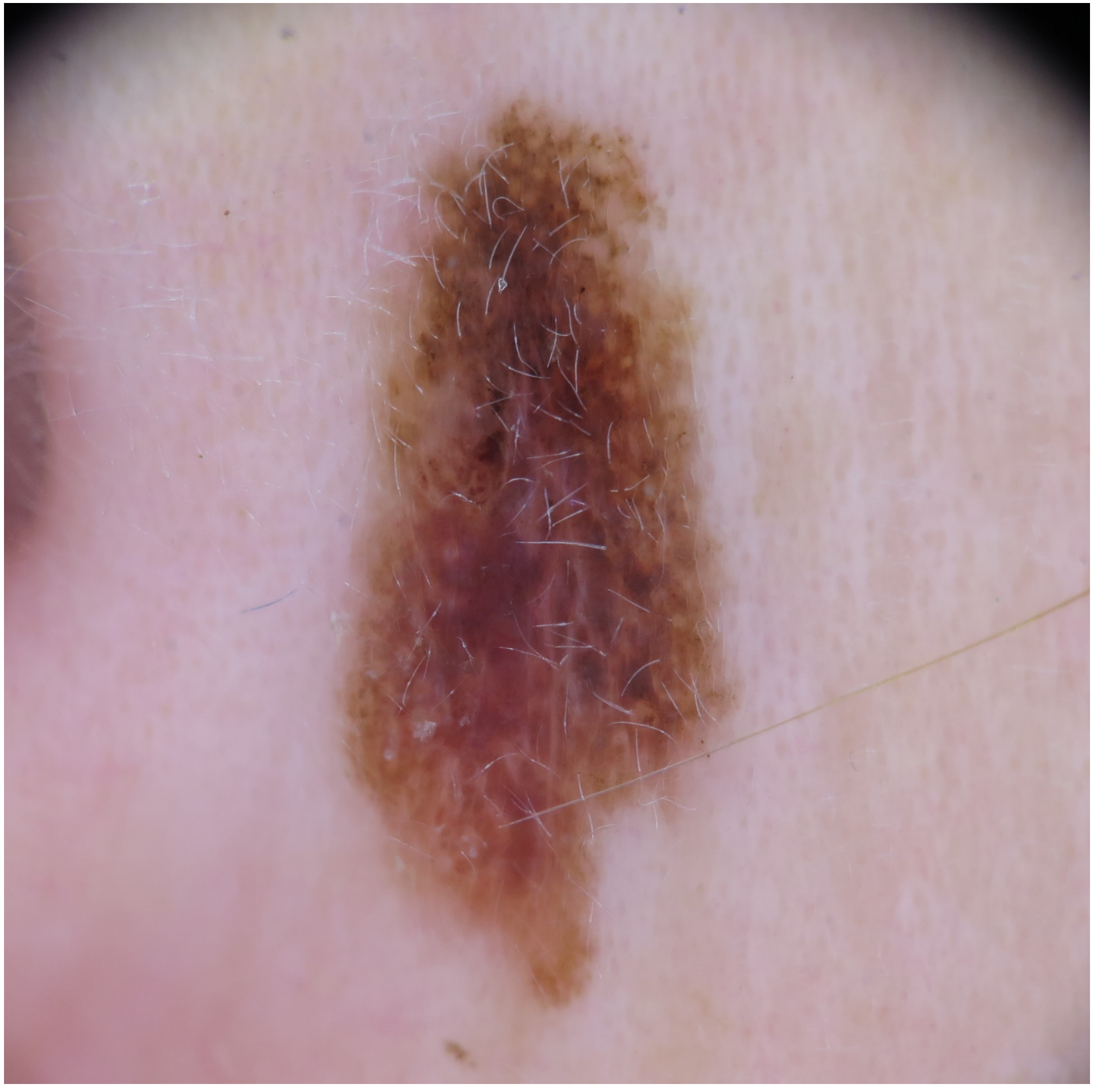

Location: Face

Invasive) Breslow interval: 0.9-1.0 mm

Case number 149

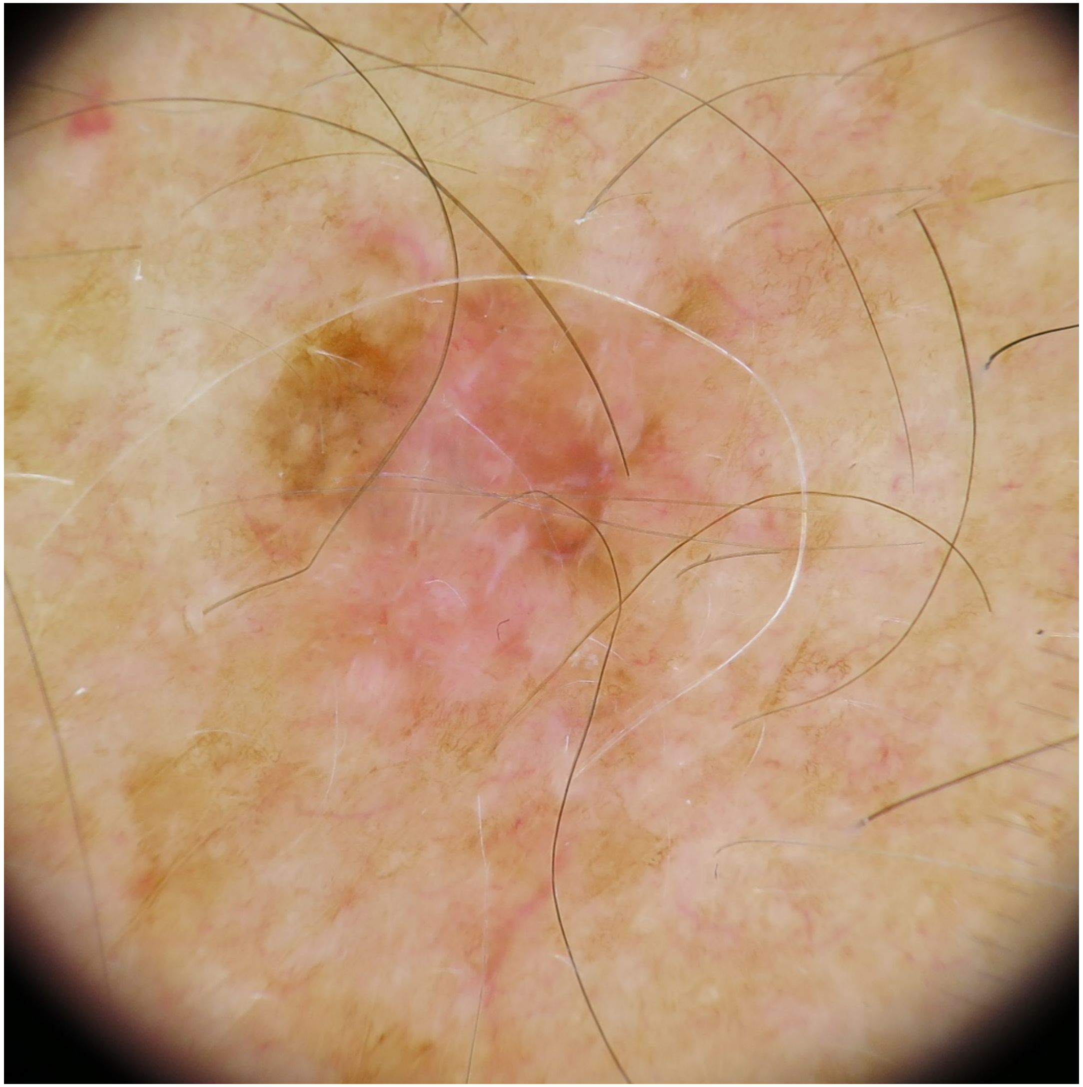

Location: Upper extremity

Invasive) Breslow interval: 0.1-0.5 mm

Case number 150

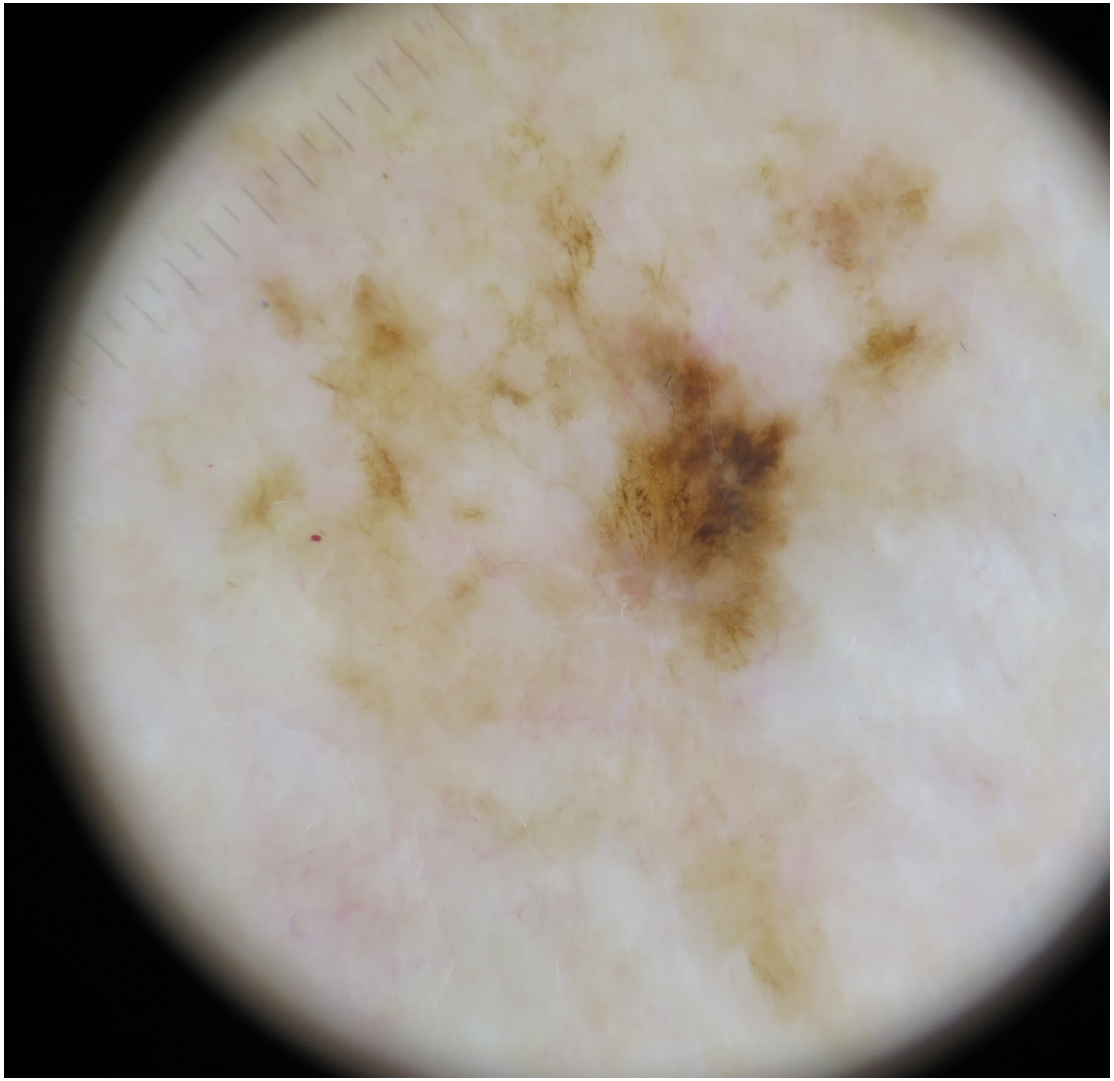

Location: Upper extremity

Invasive) Breslow interval: 0.1-0.5 mm

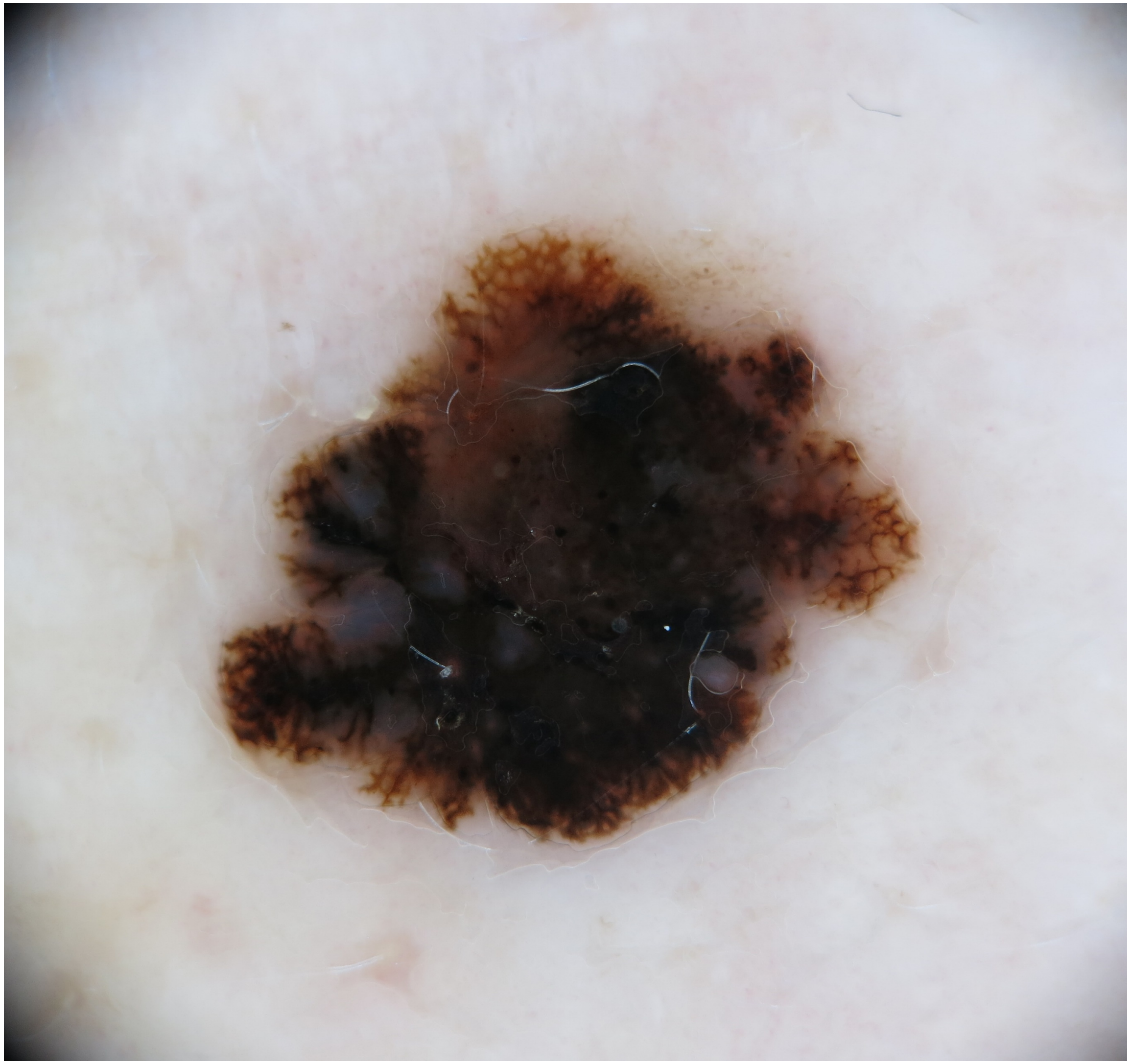

Location: Trunk

In situ melanoma

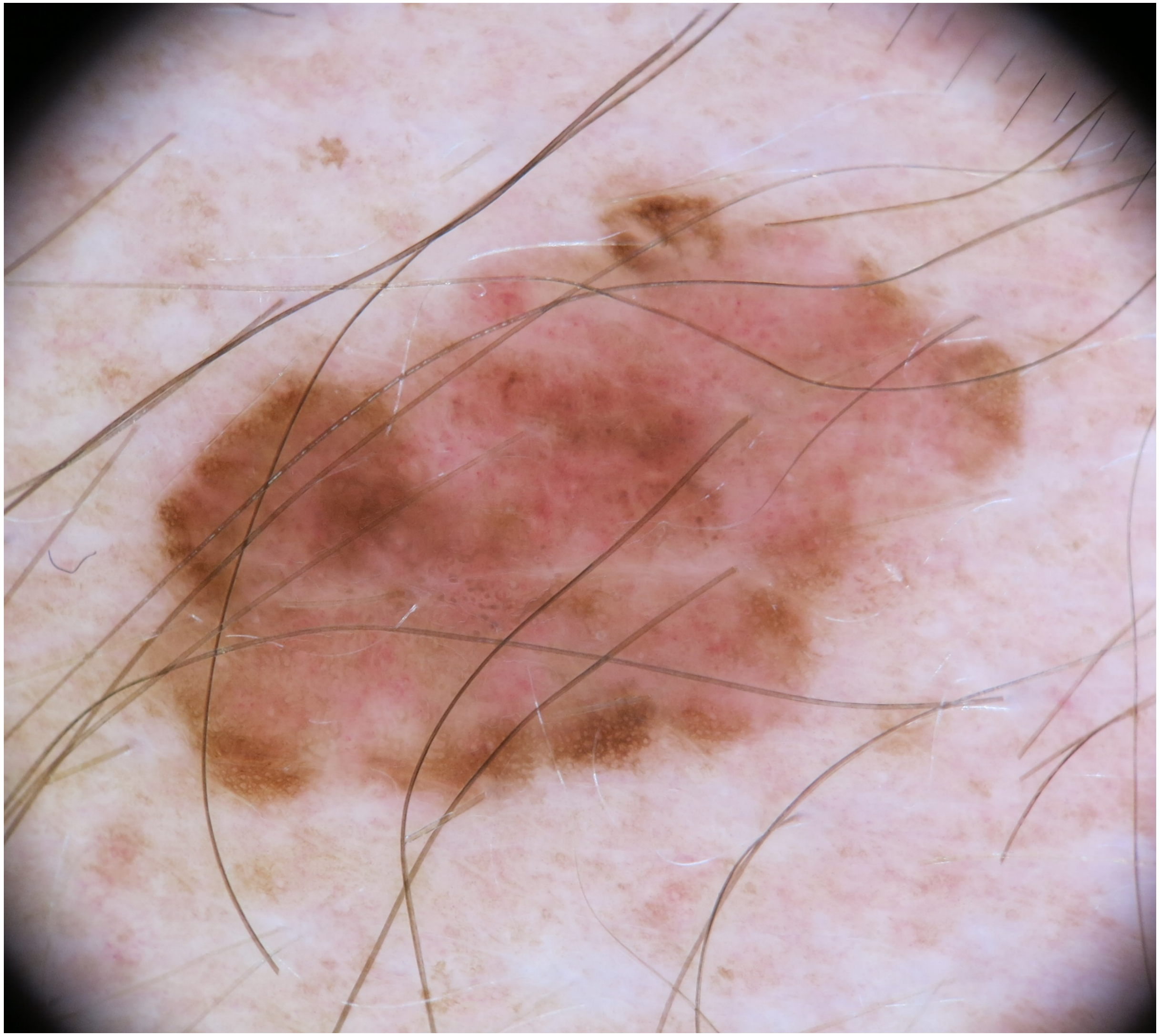

Location: Trunk

In situ melanoma

Case number 153

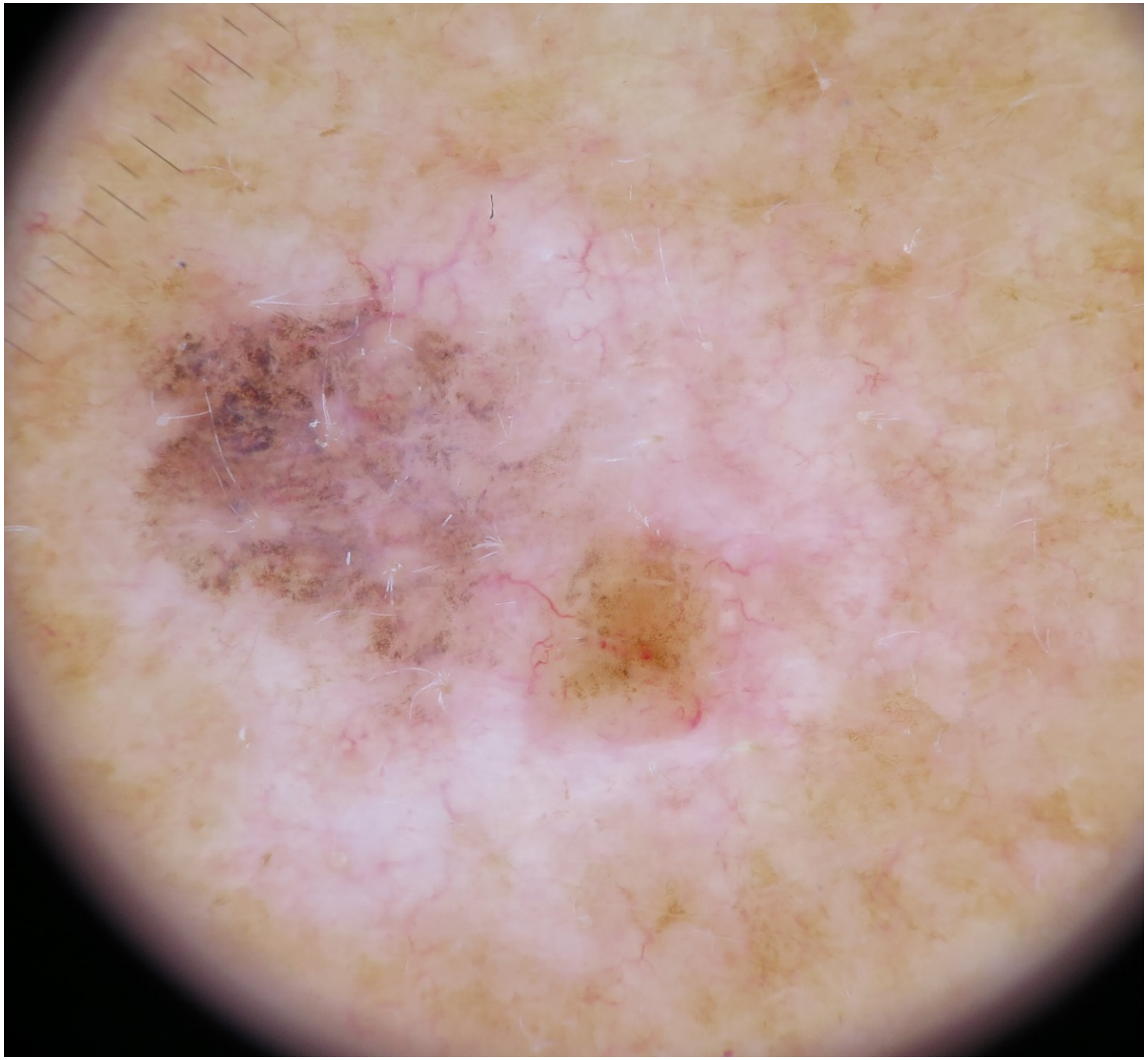

Location: Upper extremity

Invasive) Breslow interval: 0.6-0.8 mm

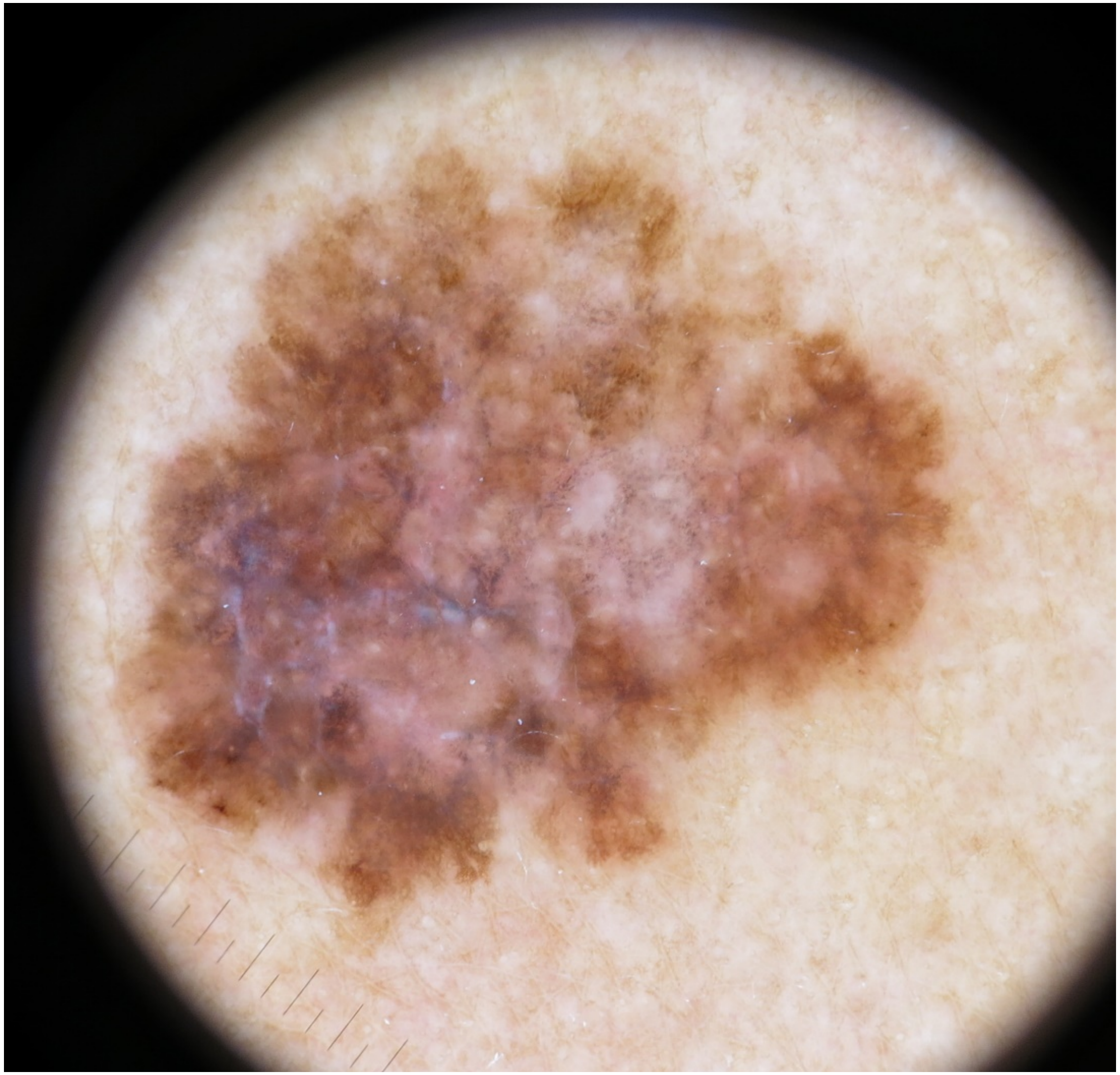

Location: Upper extremity

Invasive) Breslow interval: 0.1-0.5 mm

Case number 155

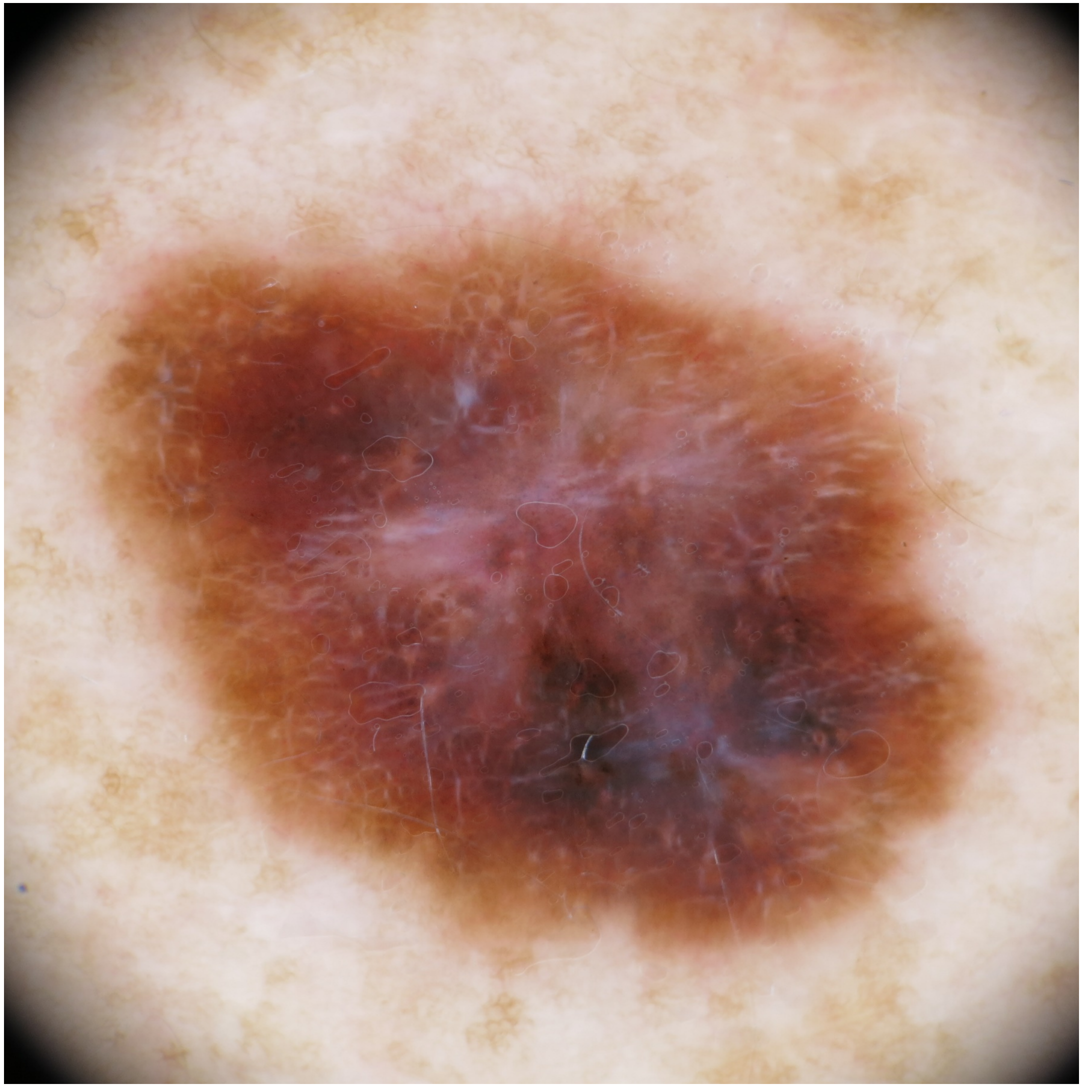

Location: Trunk

Invasive) Breslow interval: 0.6-0.8 mm

Case number 156

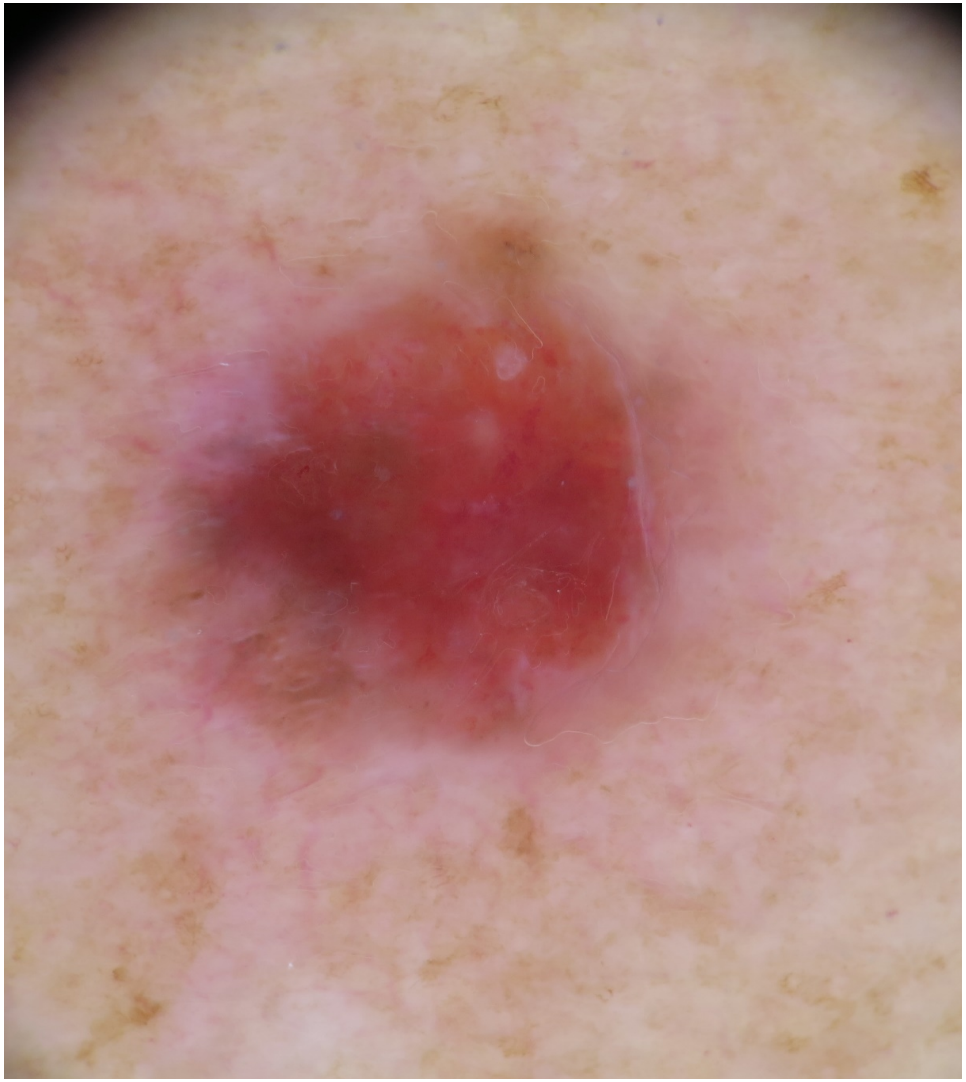

Location: Upper extremity

Invasive) Breslow interval: 1.1-2.0 mm

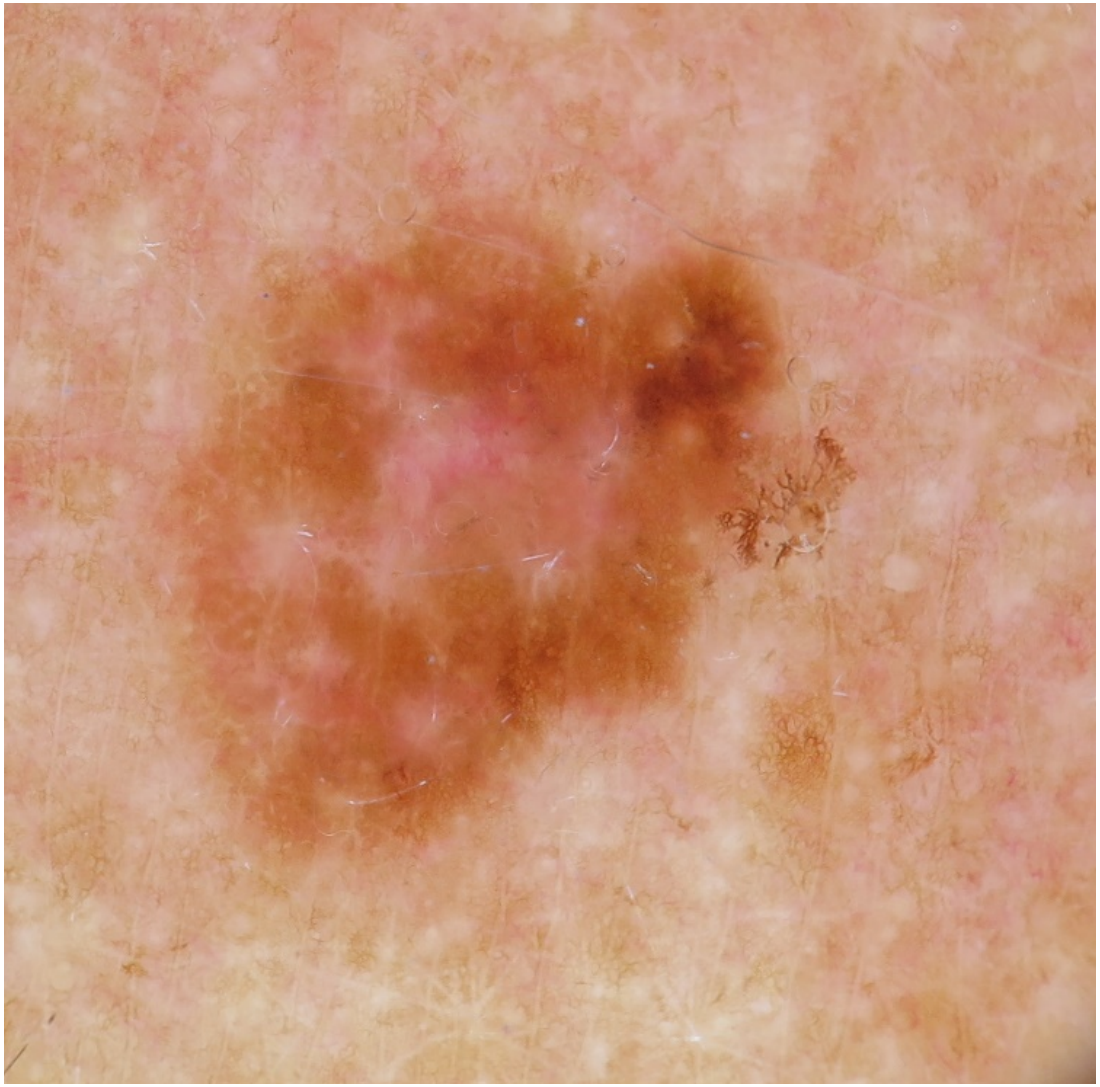

Location: Trunk

In situ melanoma

Case number 158

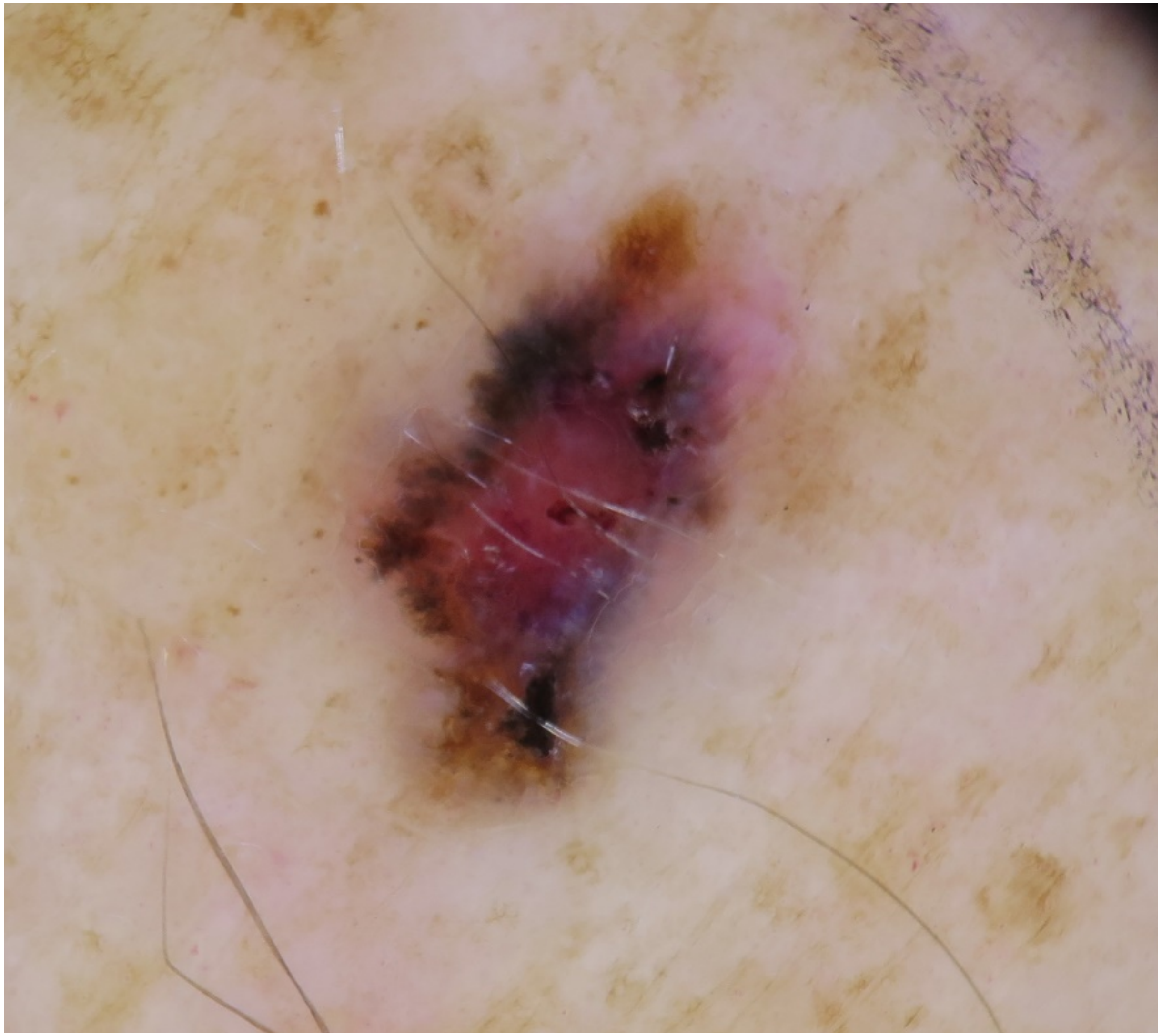

Location: Upper extremity

Invasive) Breslow interval: 1.1-2.0 mm

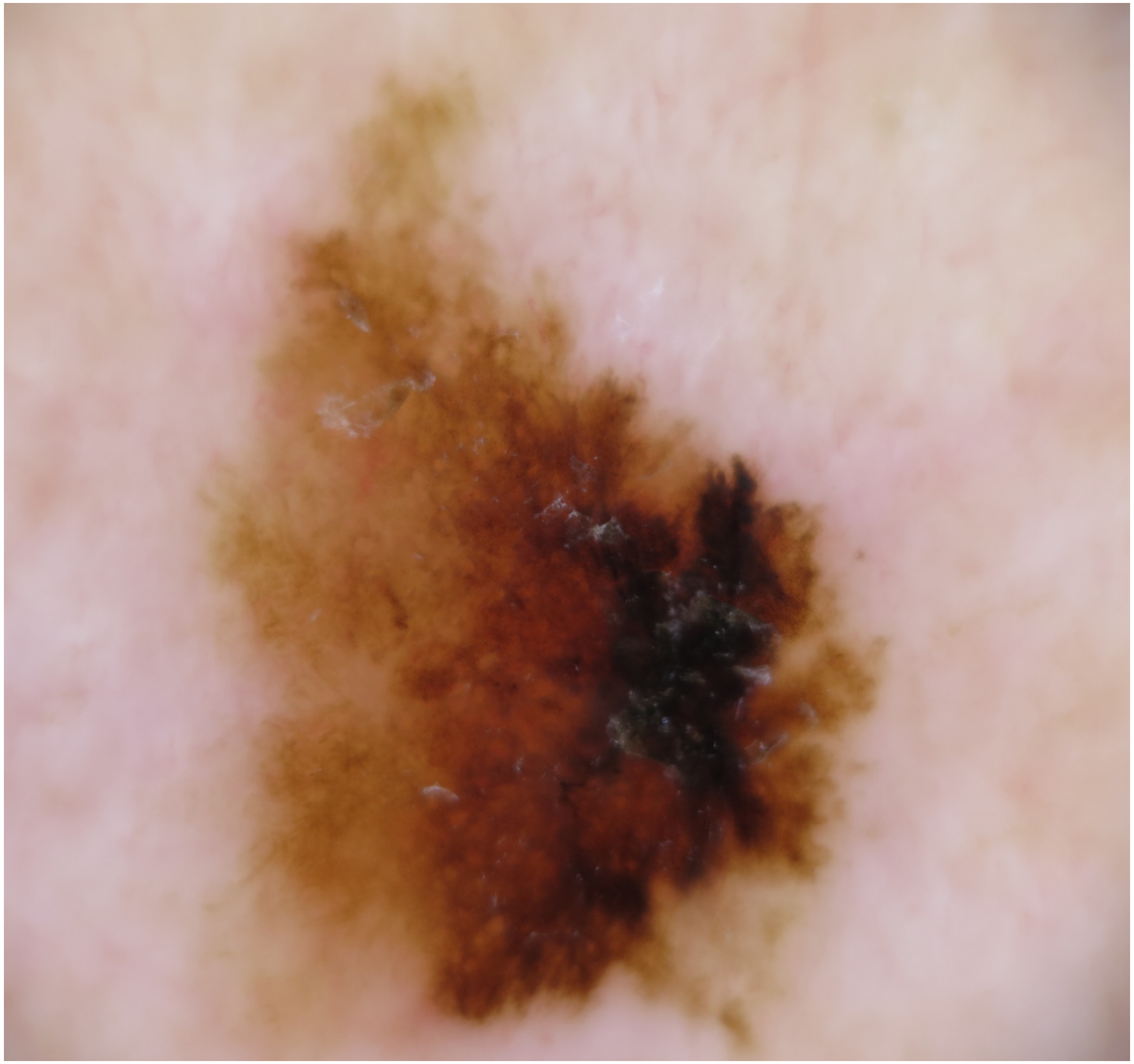

Location: Lower extremity

In situ melanoma

Case number 160

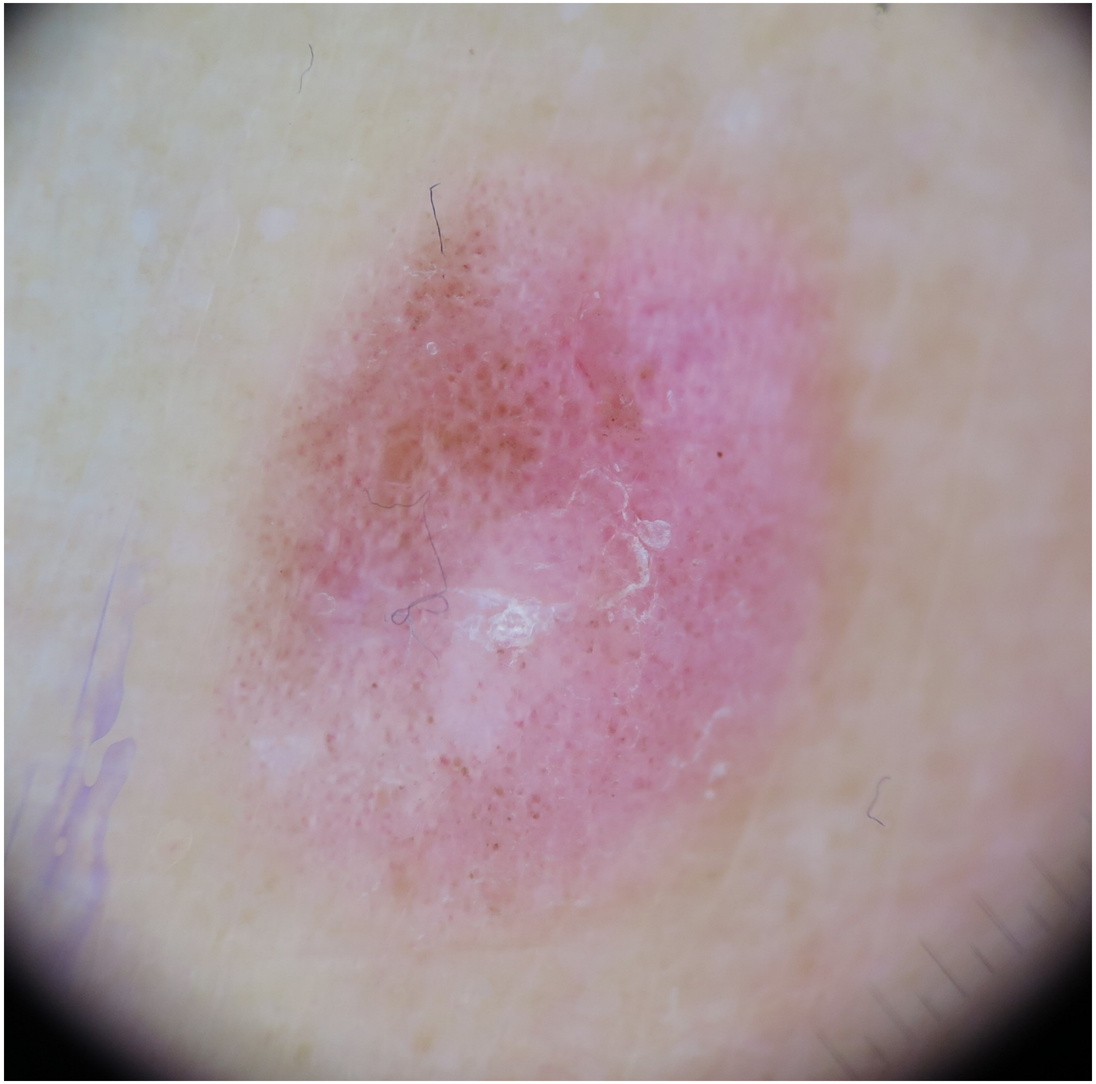

Location: Lower extremity

Invasive) Breslow interval: 0.9-1.0 mm

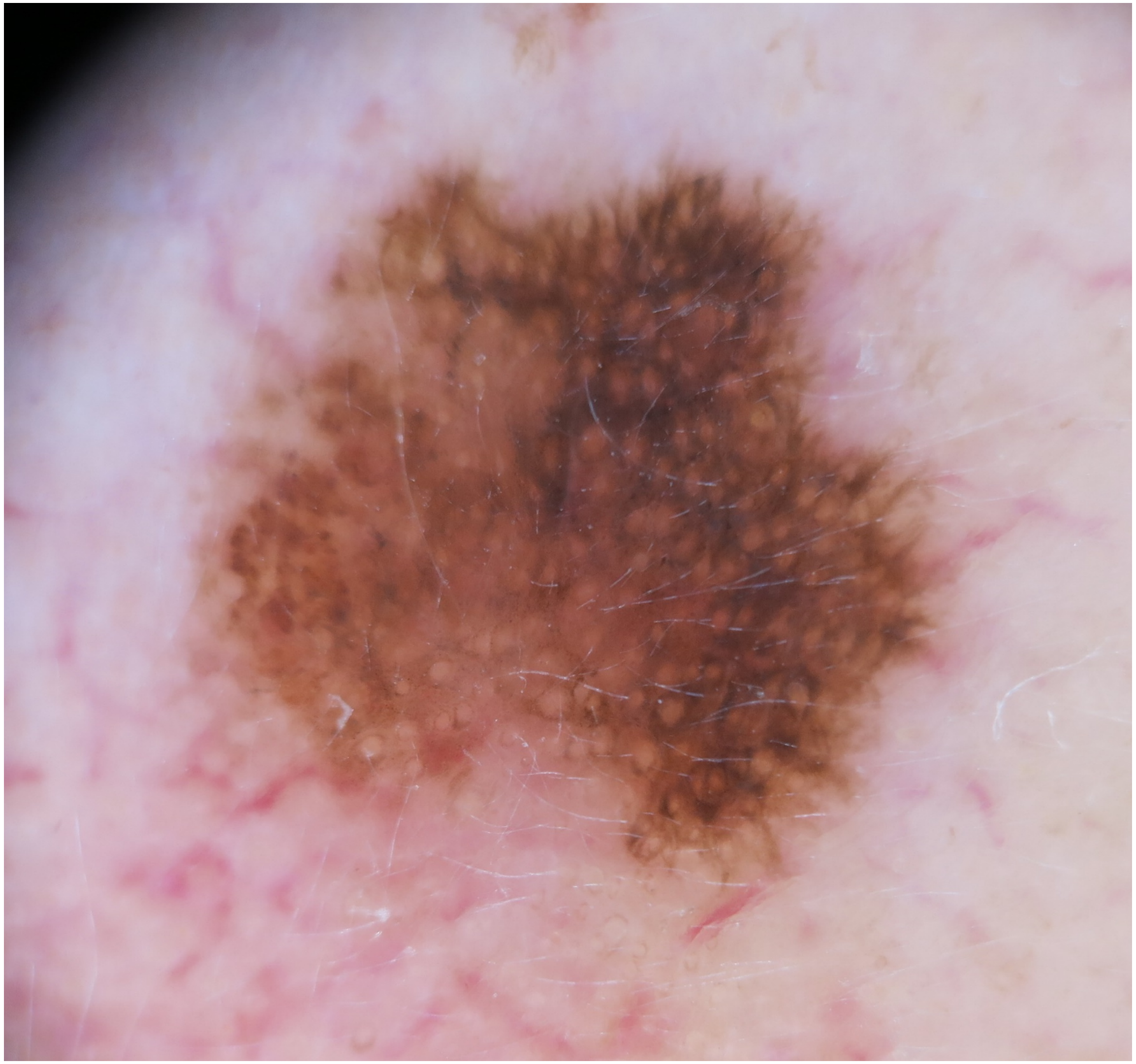

Location: Face

In situ melanoma

Case number 162

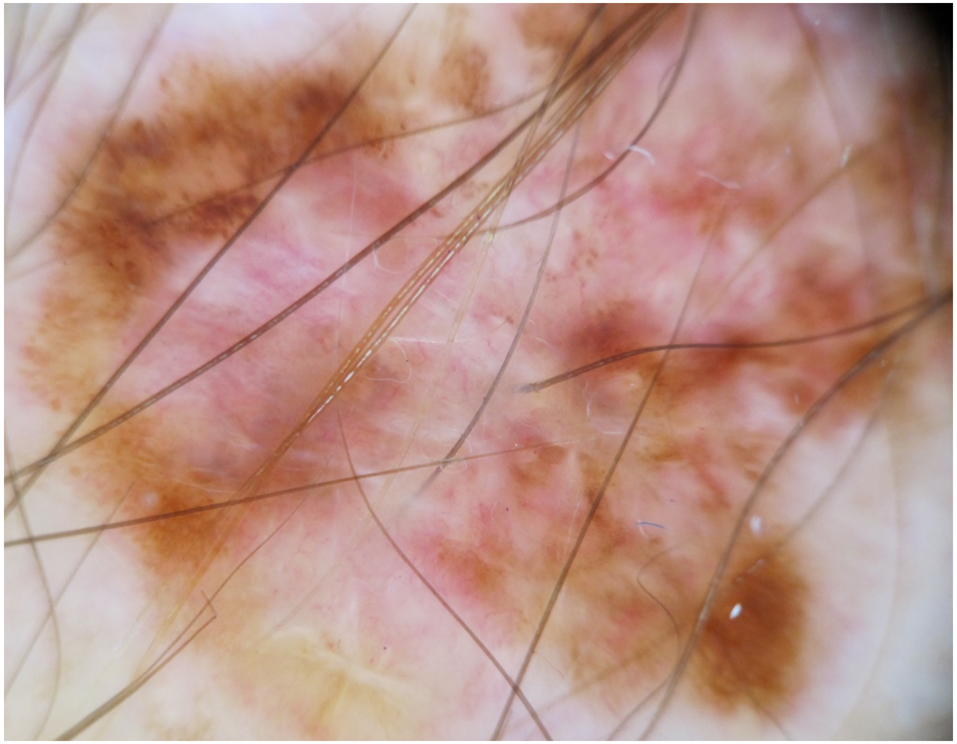

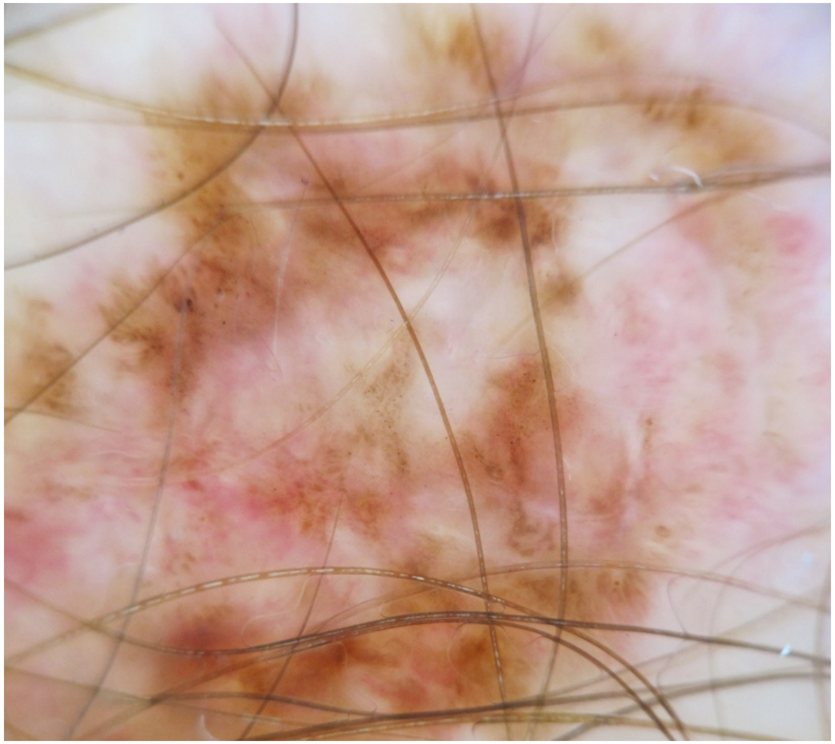

Location: Trunk

Invasive) Breslow interval: 0.1-0.5 mm

Case number 163

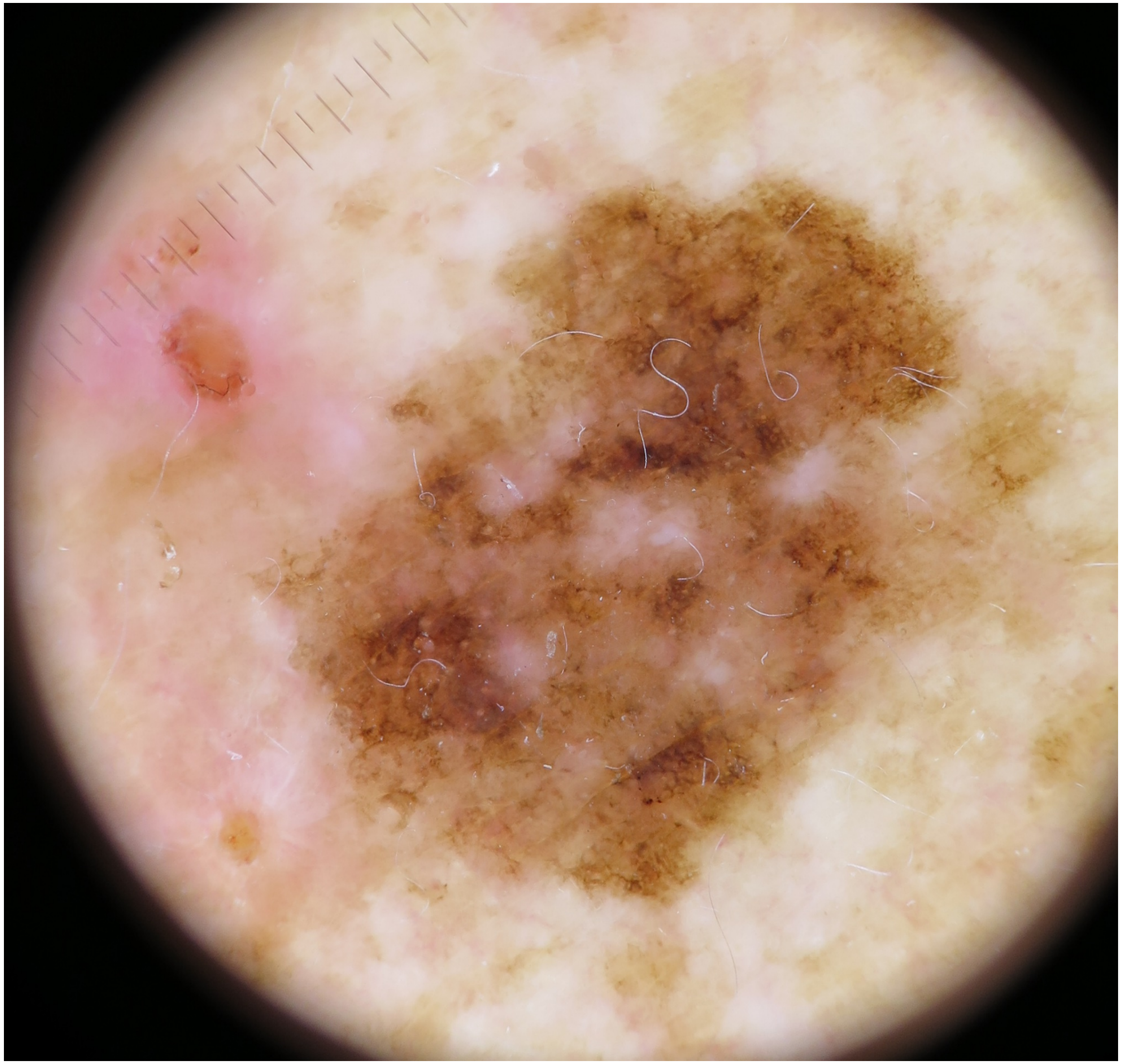

Location: Upper extremity

In situ melanoma

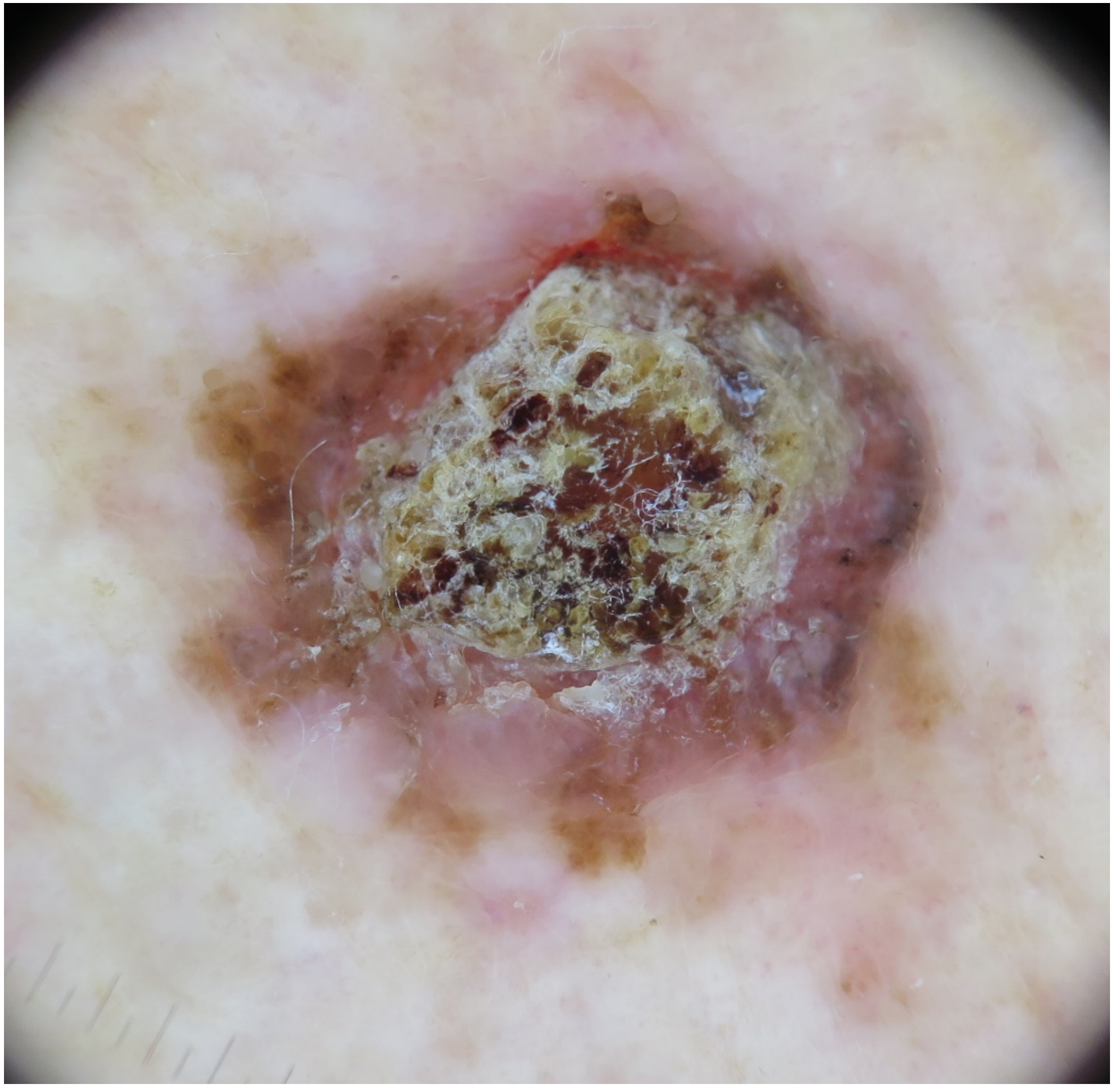

Location: Upper extremity

Invasive) Breslow interval: 2.1-4.0 mm

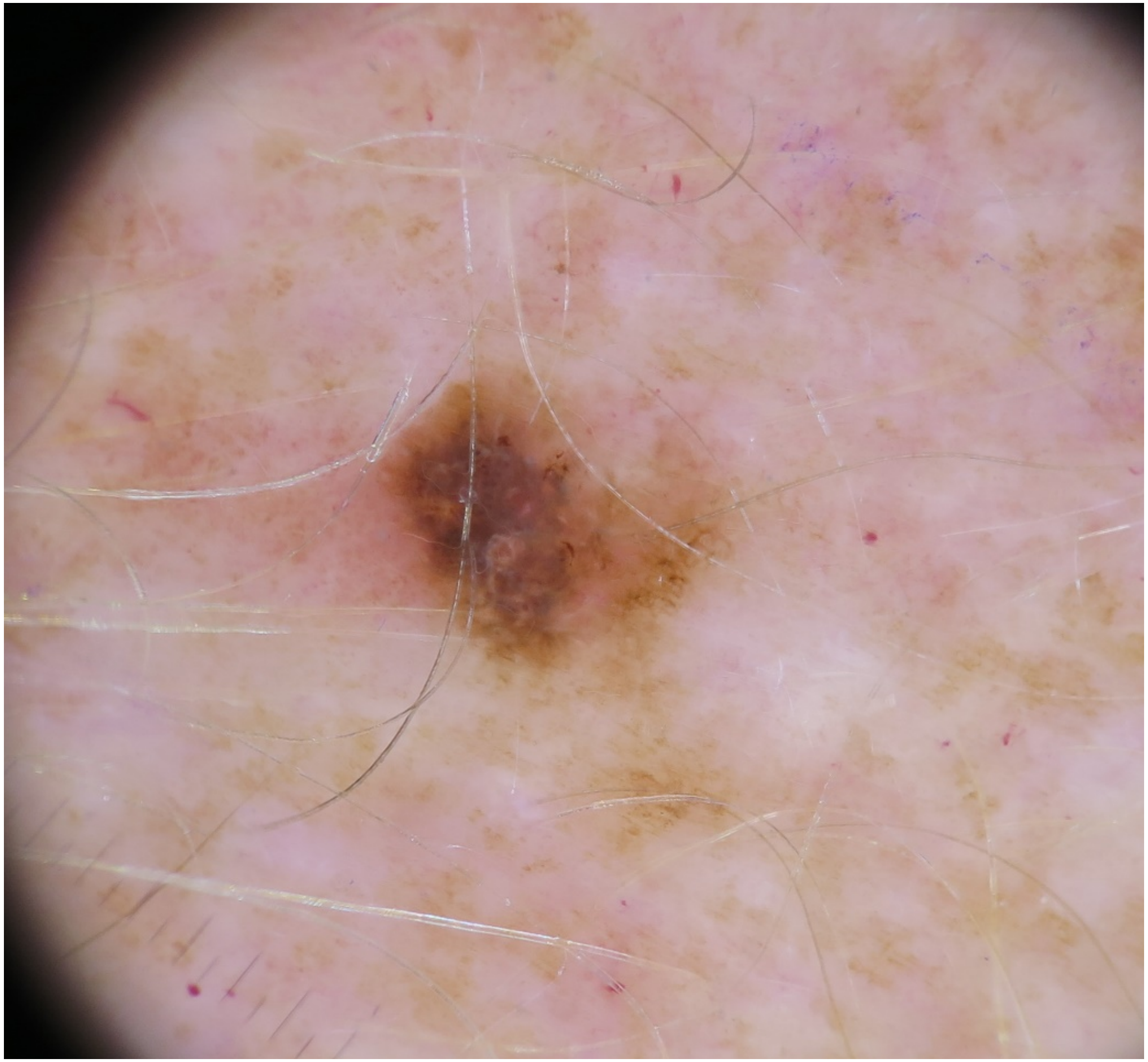

Location: Upper extremity

In situ melanoma

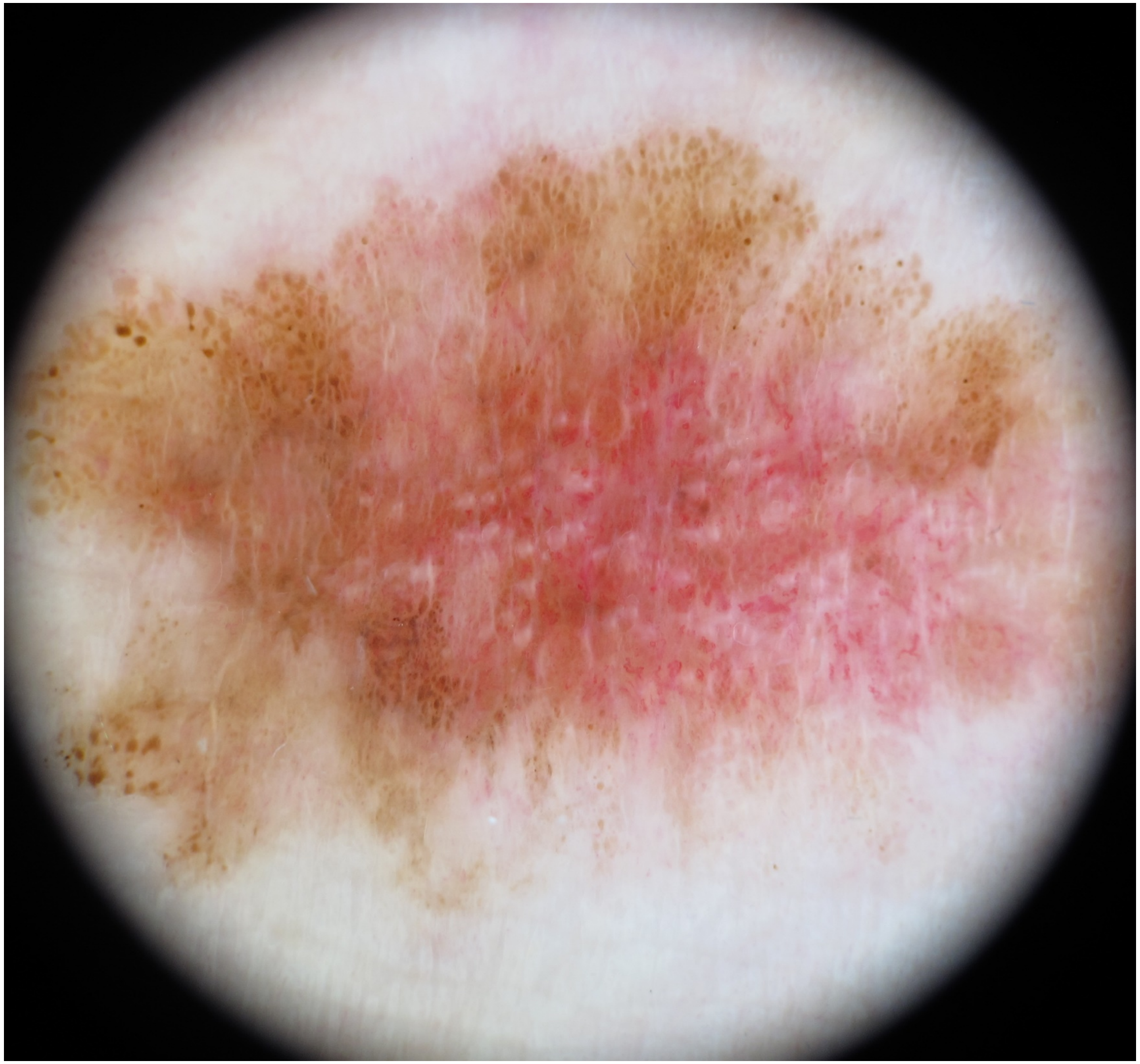

Location: Lower extremity

Invasive) Breslow interval: 0.9-1.0 mm

Case number 167

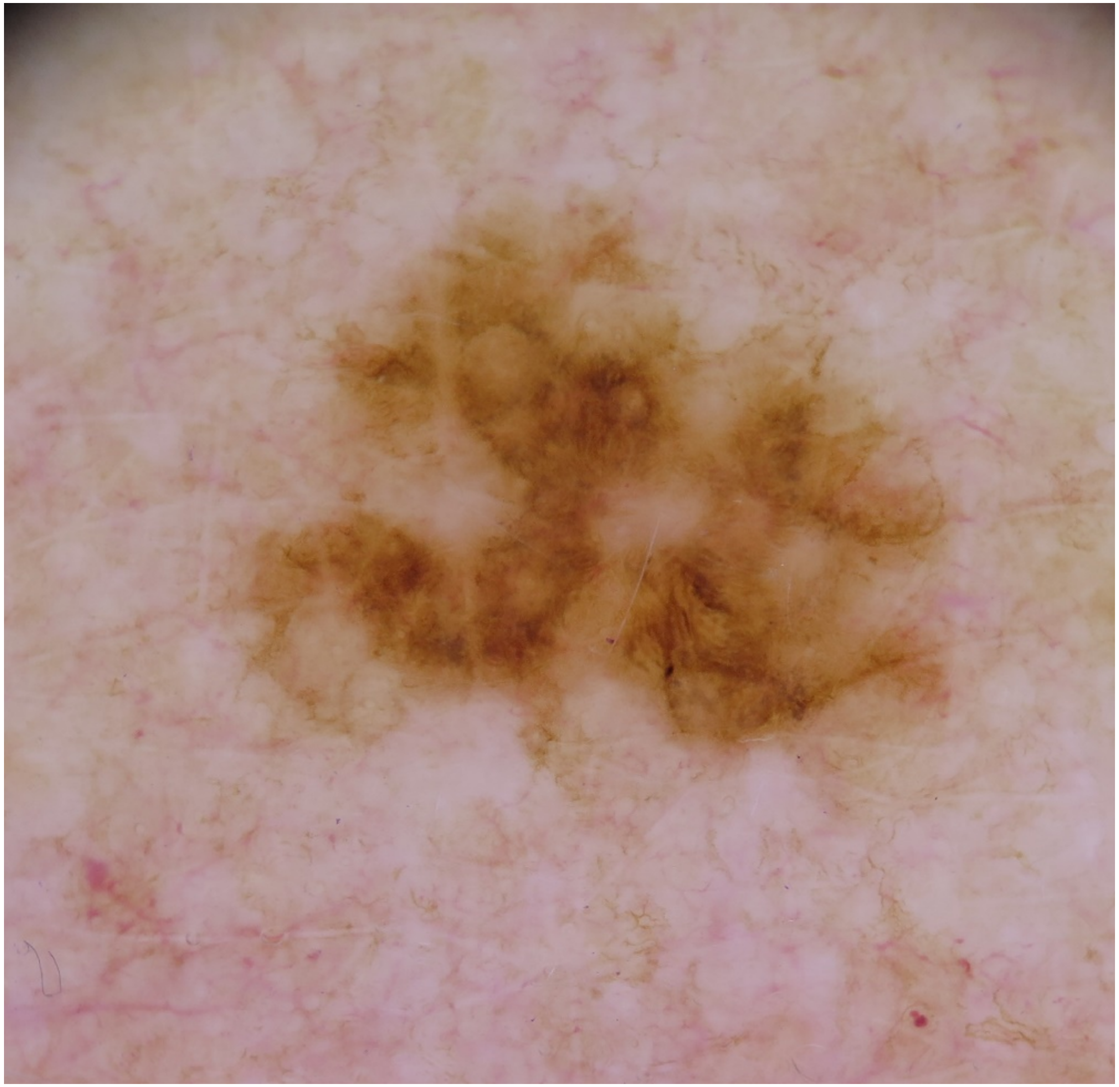

Location: Trunk

In situ melanoma

Case number 168

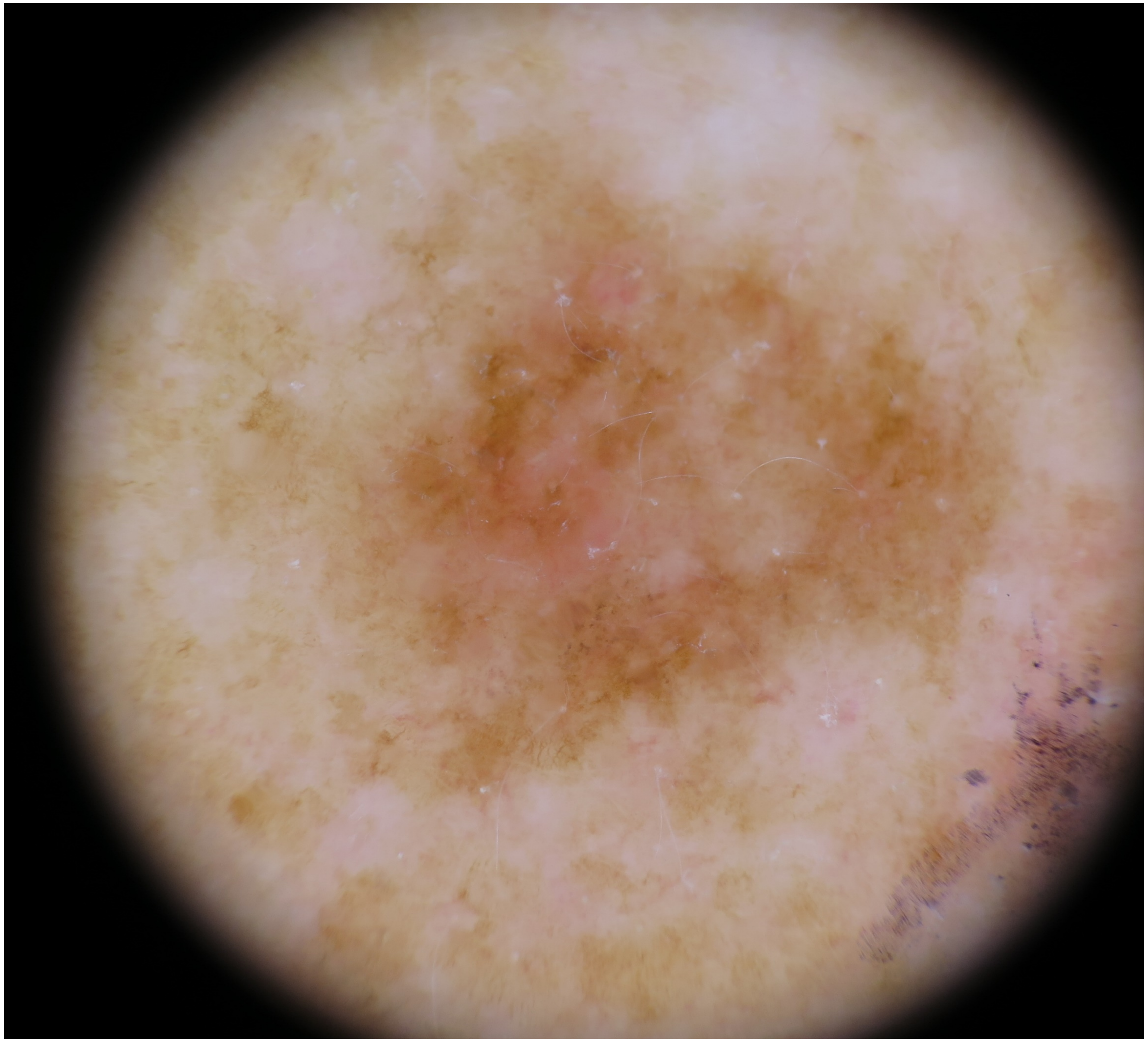

Location: Trunk

In situ melanoma

Case number 169

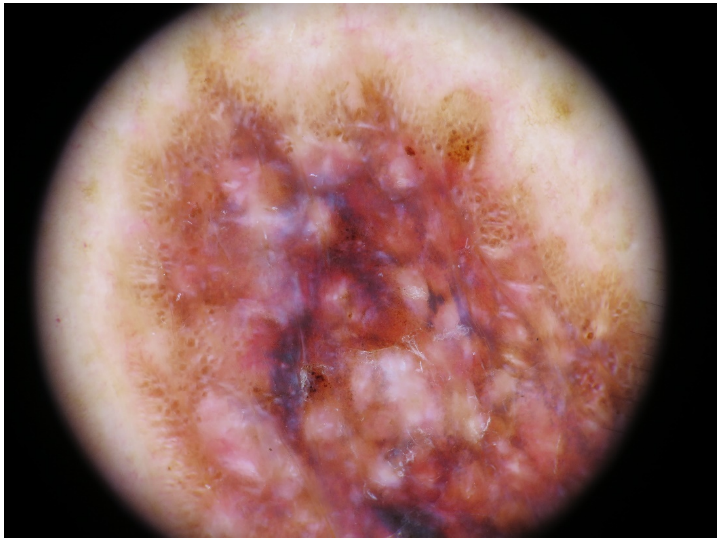

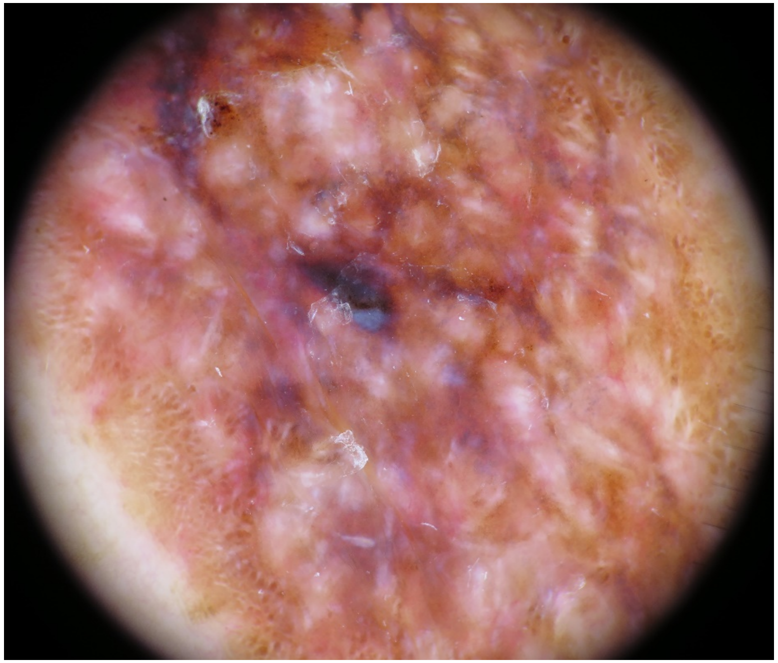

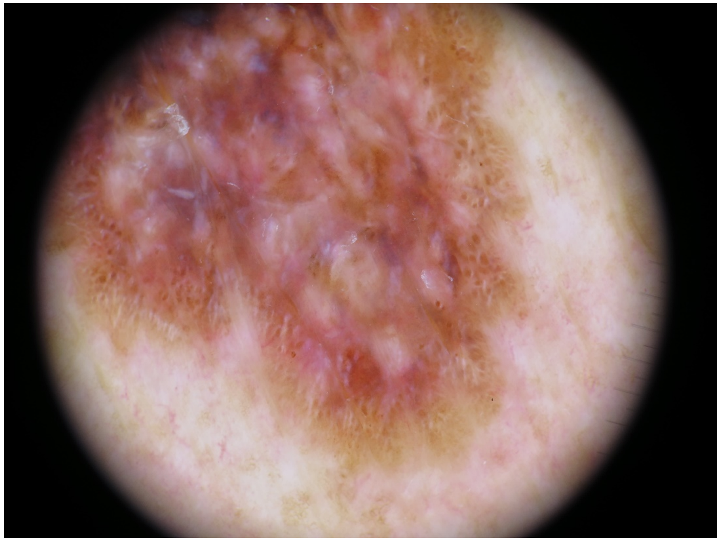

Location: Trunk

Invasive) Breslow interval: 0.1-0.5 mm

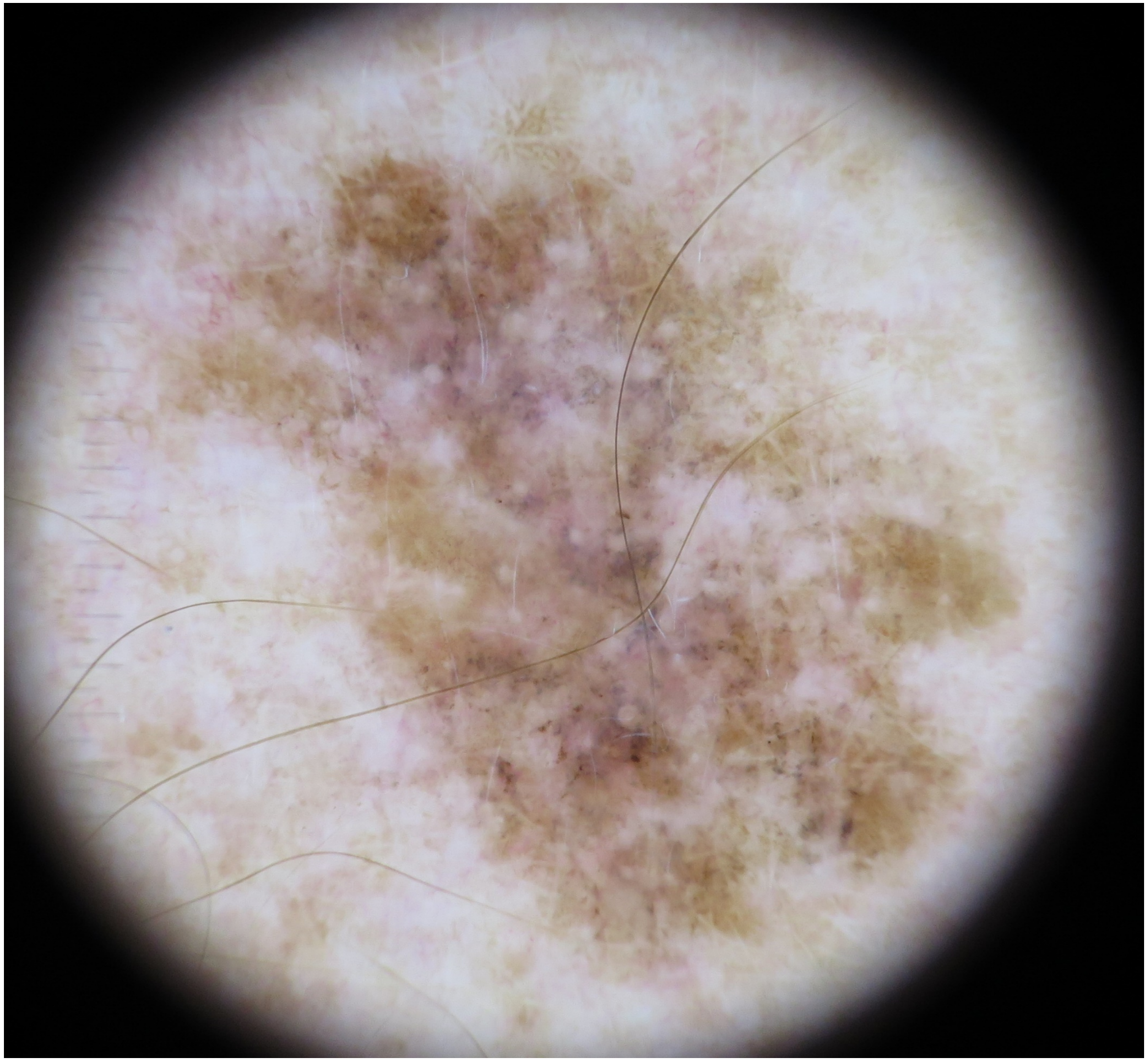

Location: Upper extremity

In situ melanoma

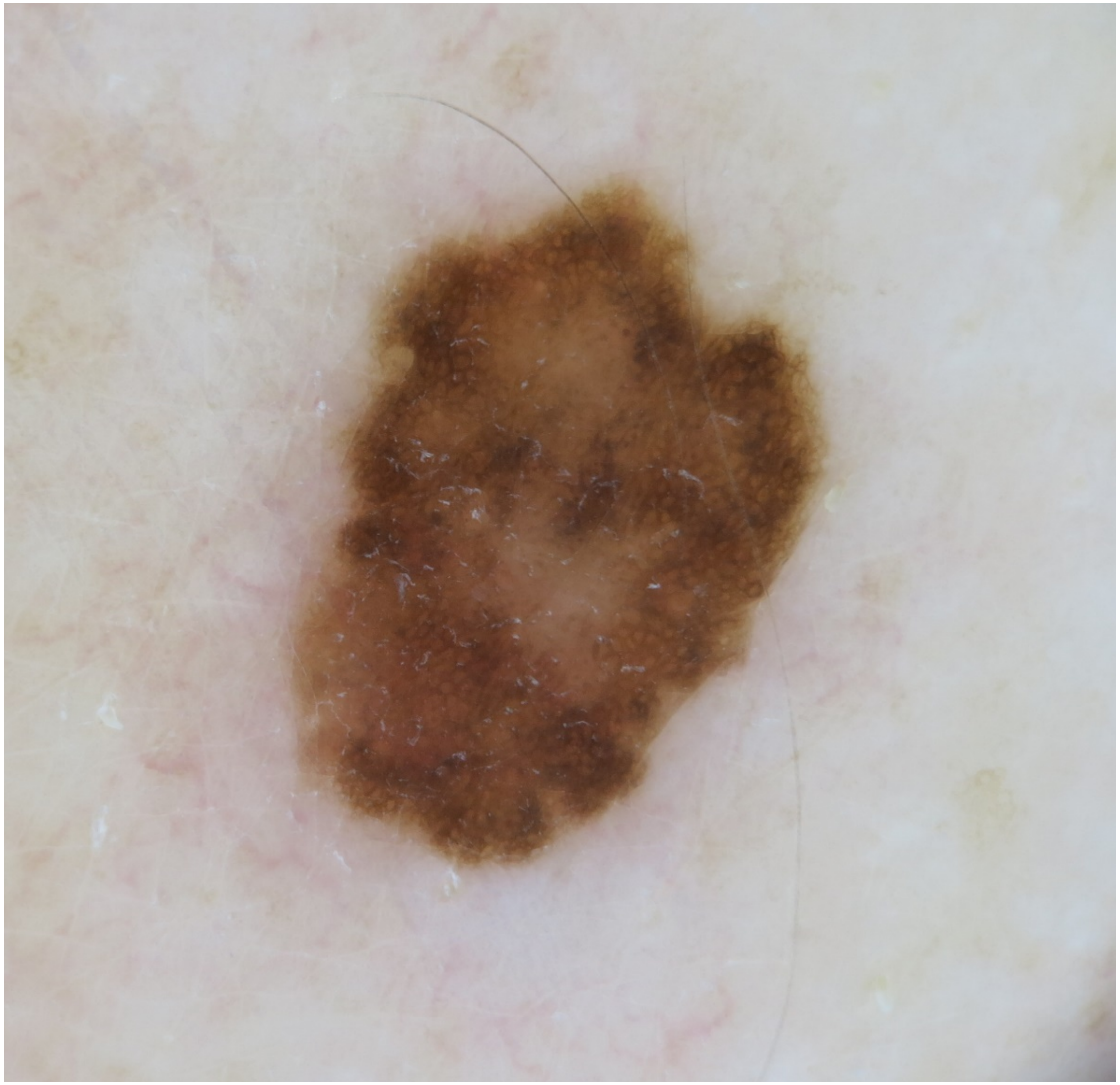

Location: Trunk

In situ melanoma

Case number 172

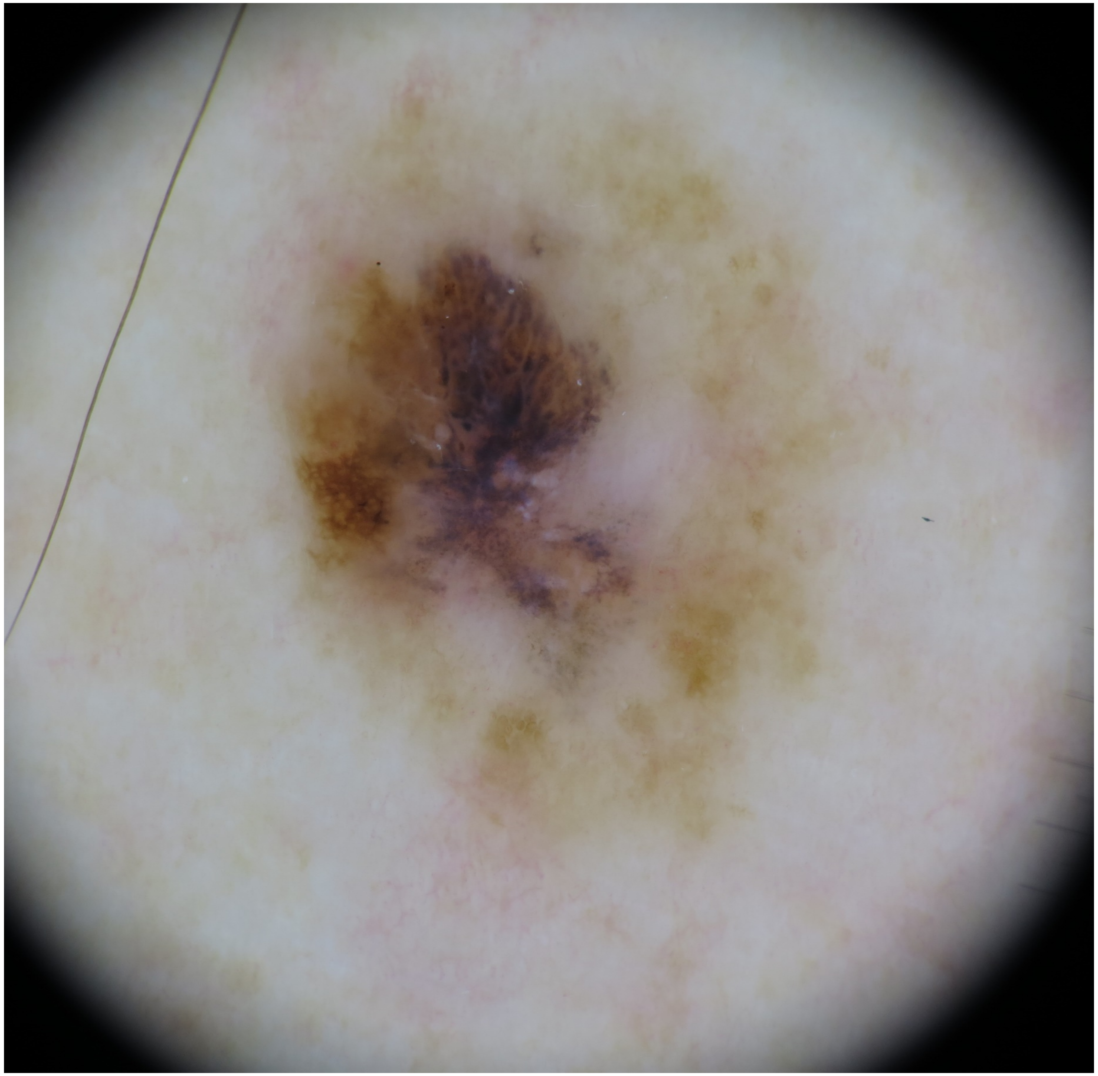

Location: Lower extremity

Invasive) Breslow interval: 0.6-0.8 mm

Case number 173

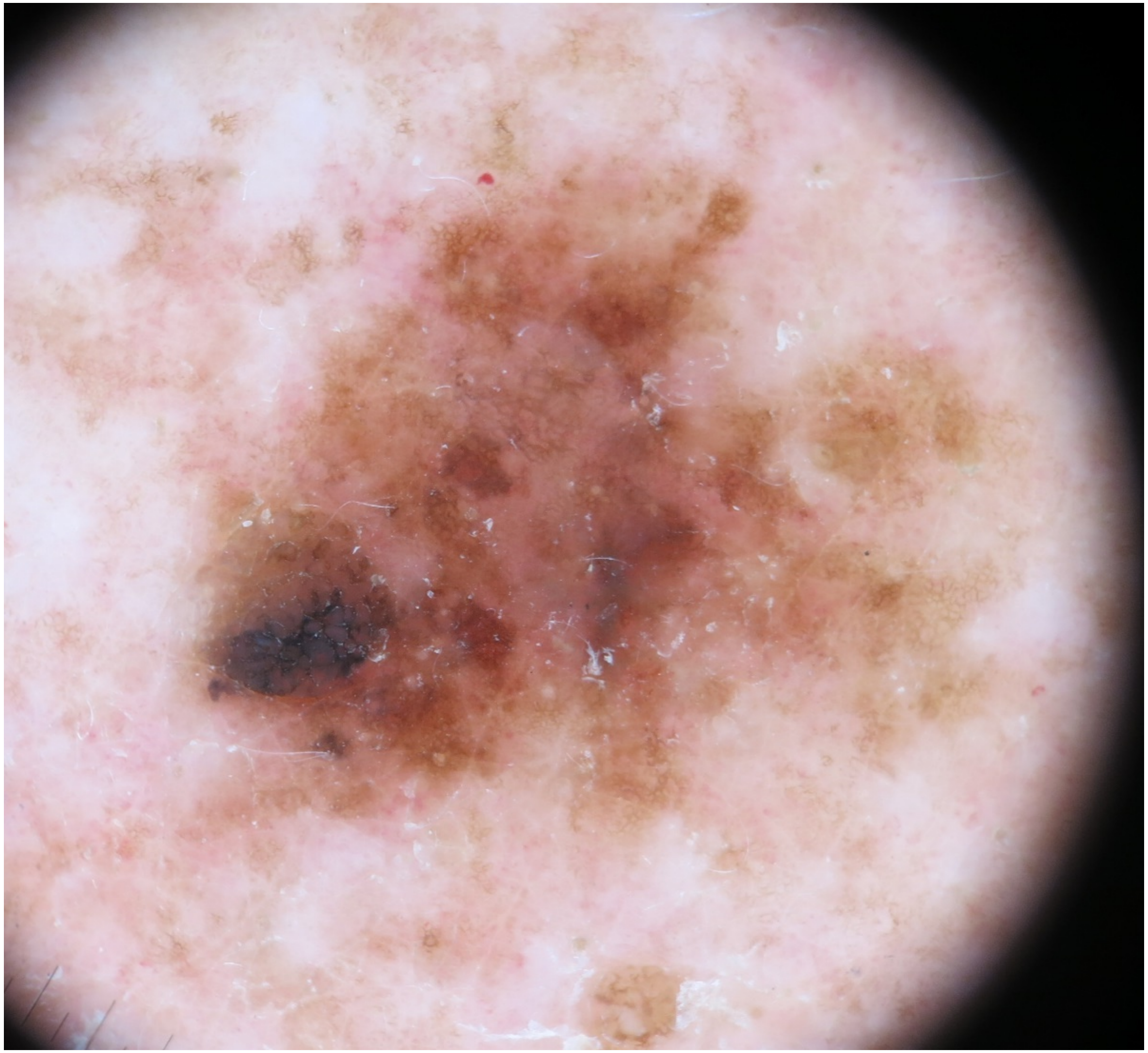

Location: Trunk

In situ melanoma

Case number 174

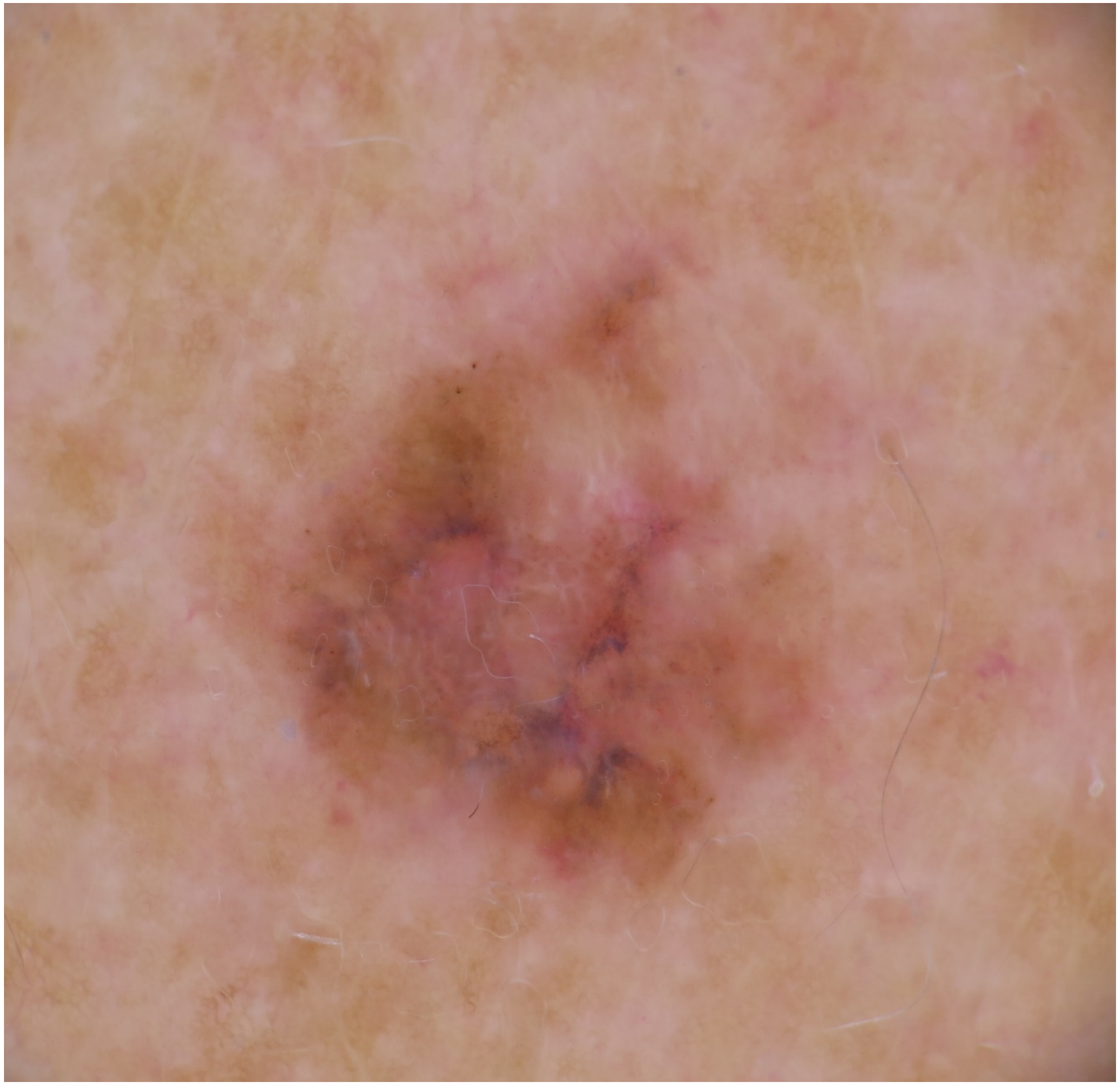

Location: Trunk

In situ melanoma

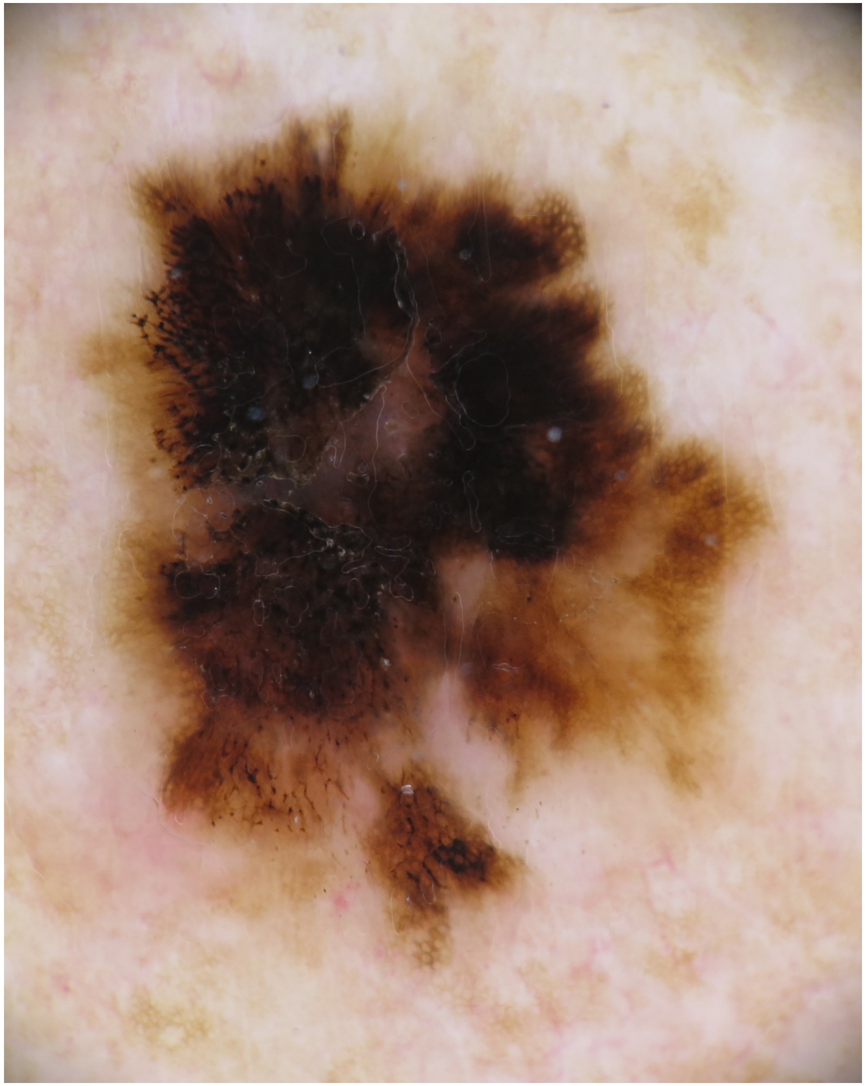

Location: Upper extremity

Invasive) Breslow interval: 0.6-0.8 mm

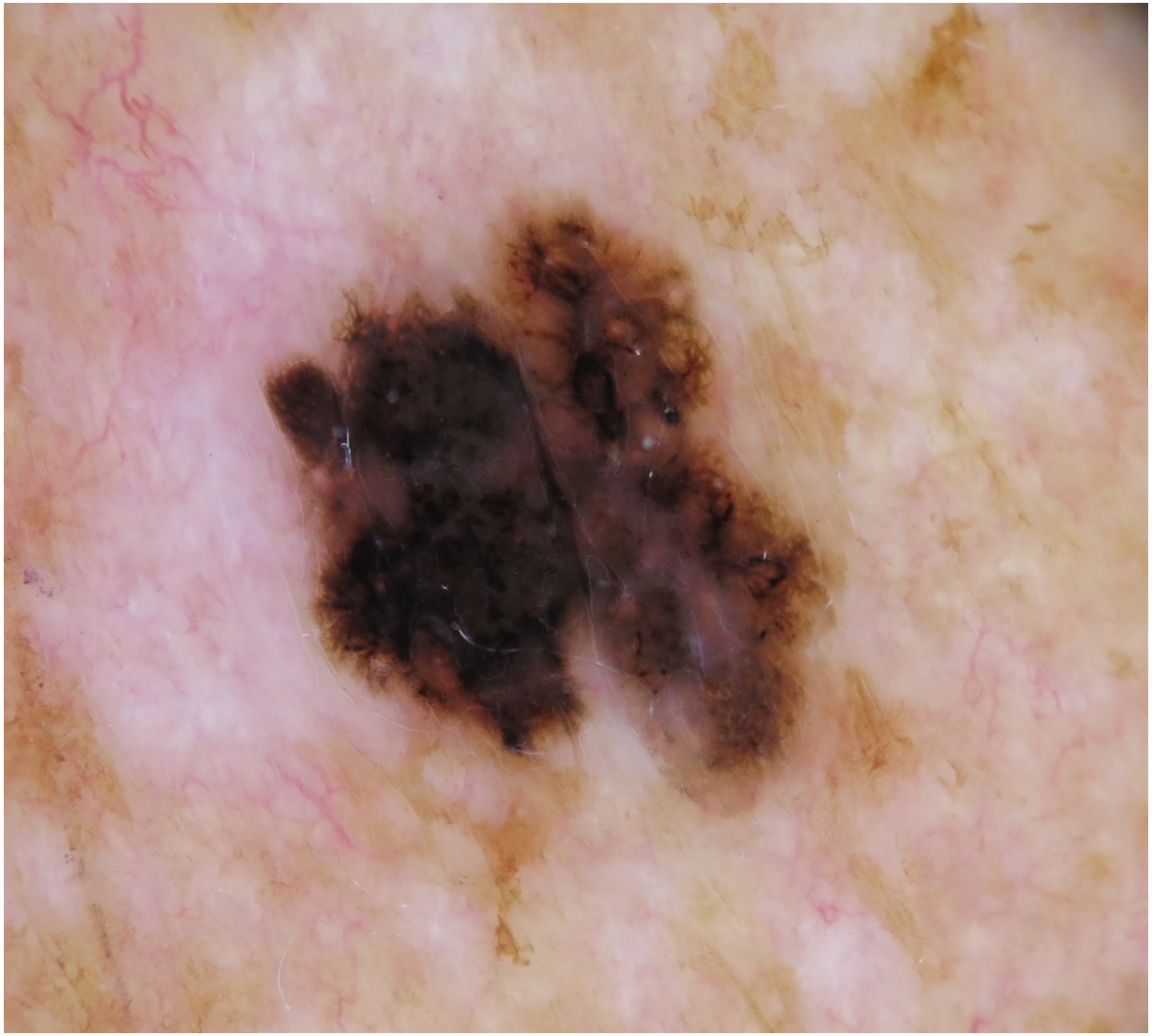

Location: Trunk

In situ melanoma

Case number 177

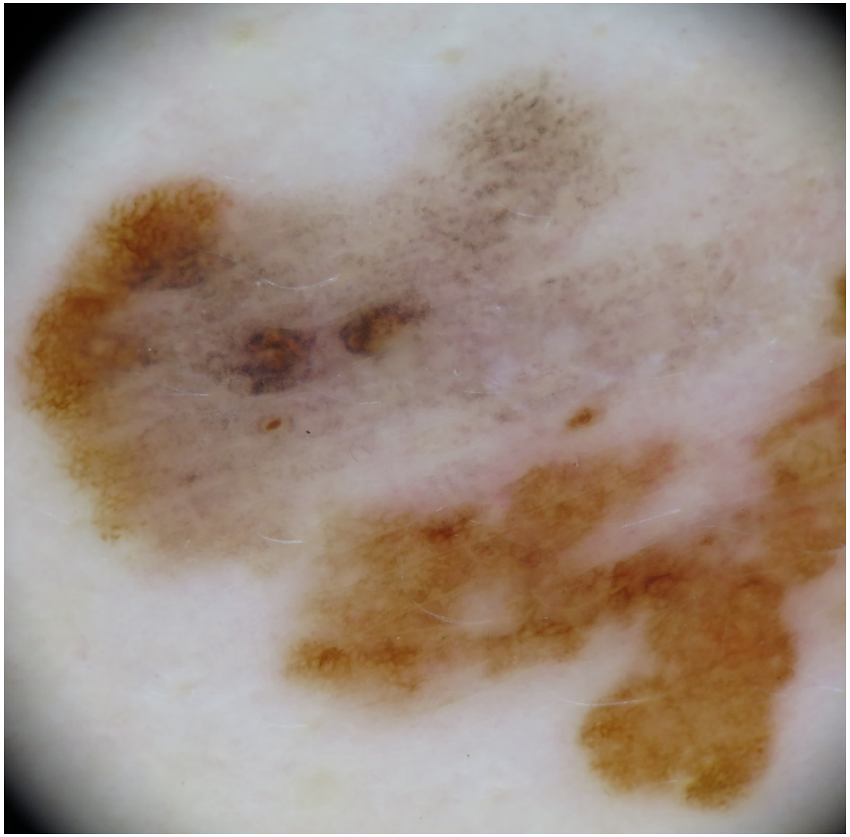

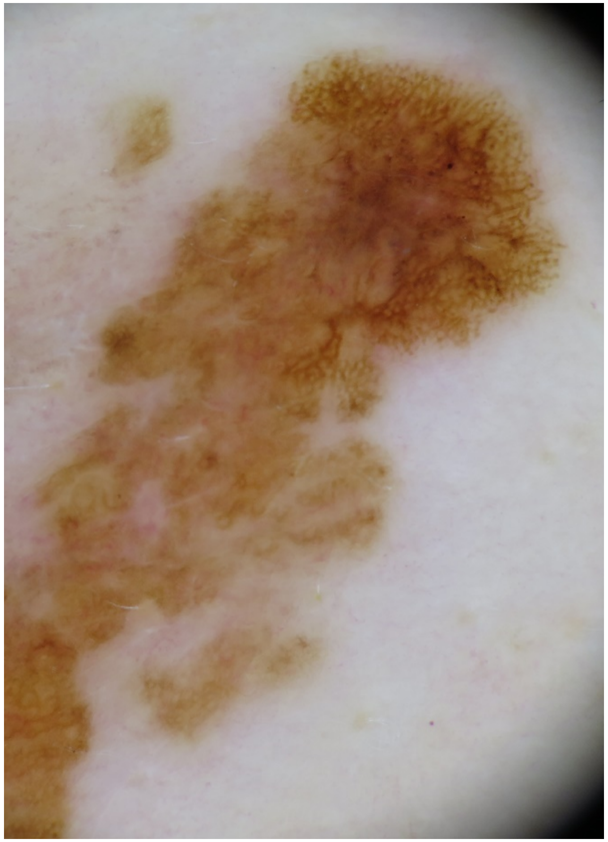

Location: Trunk

In situ melanoma

Case number 178

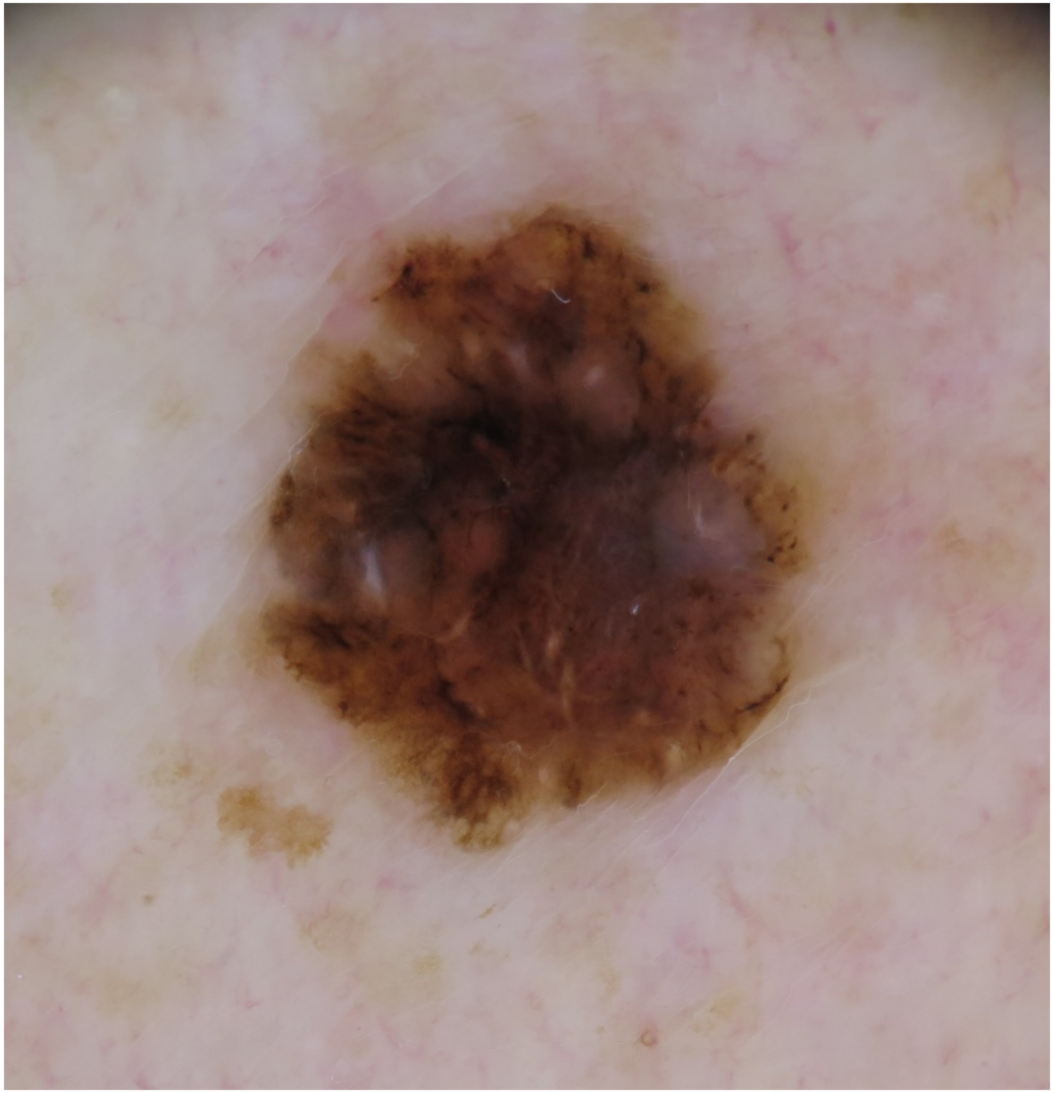

Location: Upper extremity

Invasive) Breslow interval: 0.1-0.5 mm

Case number 179

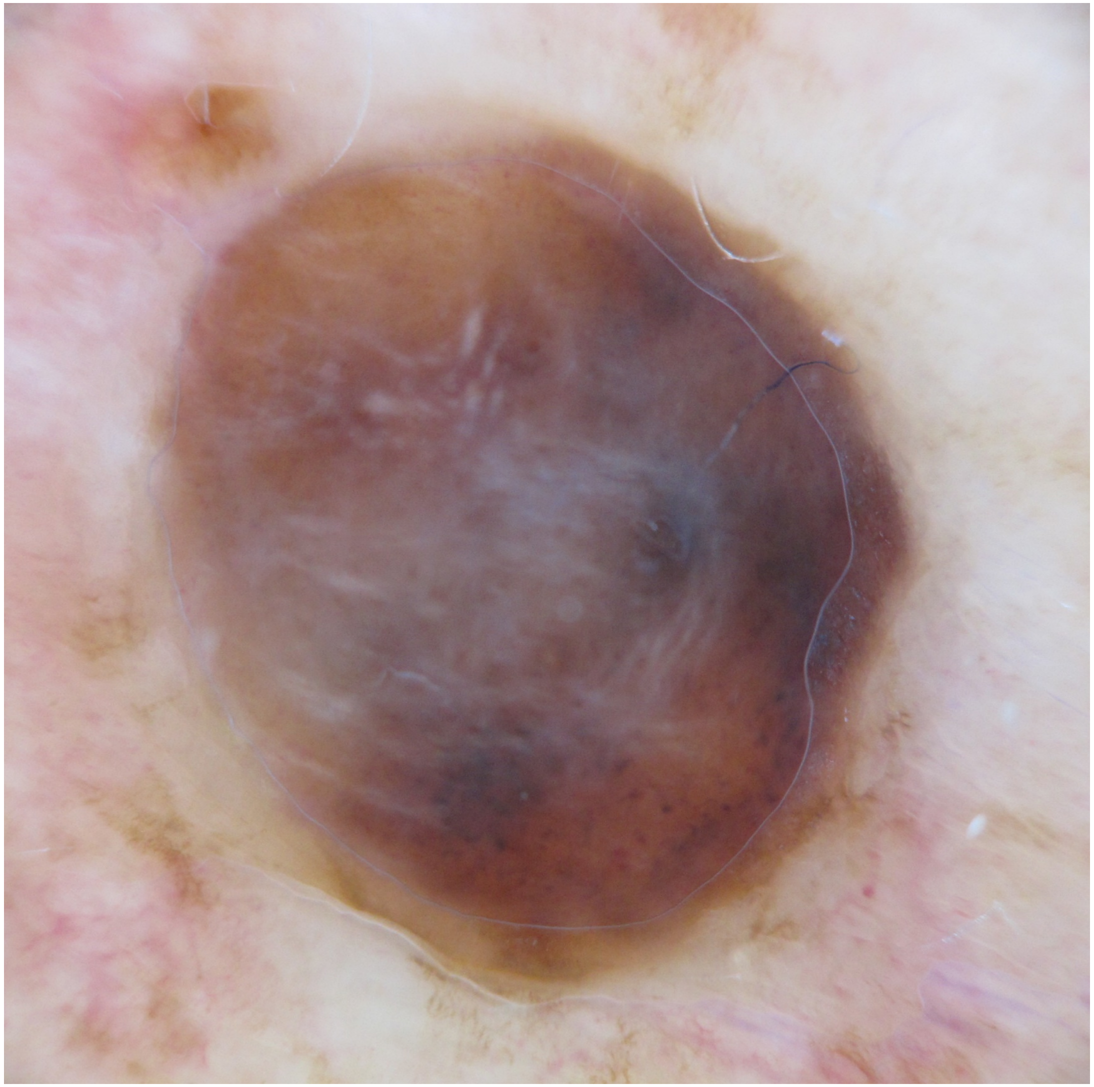

Location: Upper extremity

Invasive) Breslow interval: 1.1-2.0 mm

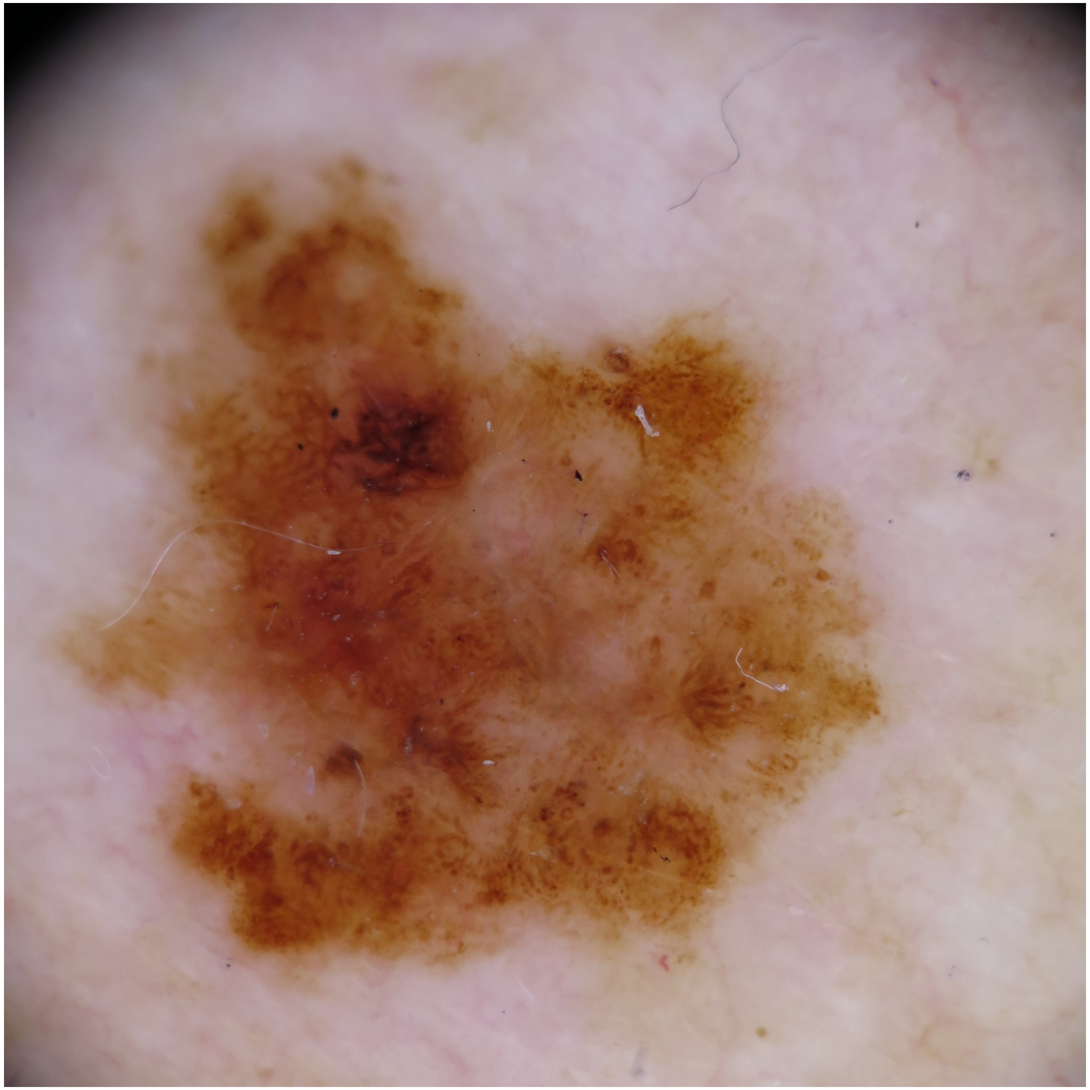

Location: Upper extremity

In situ melanoma

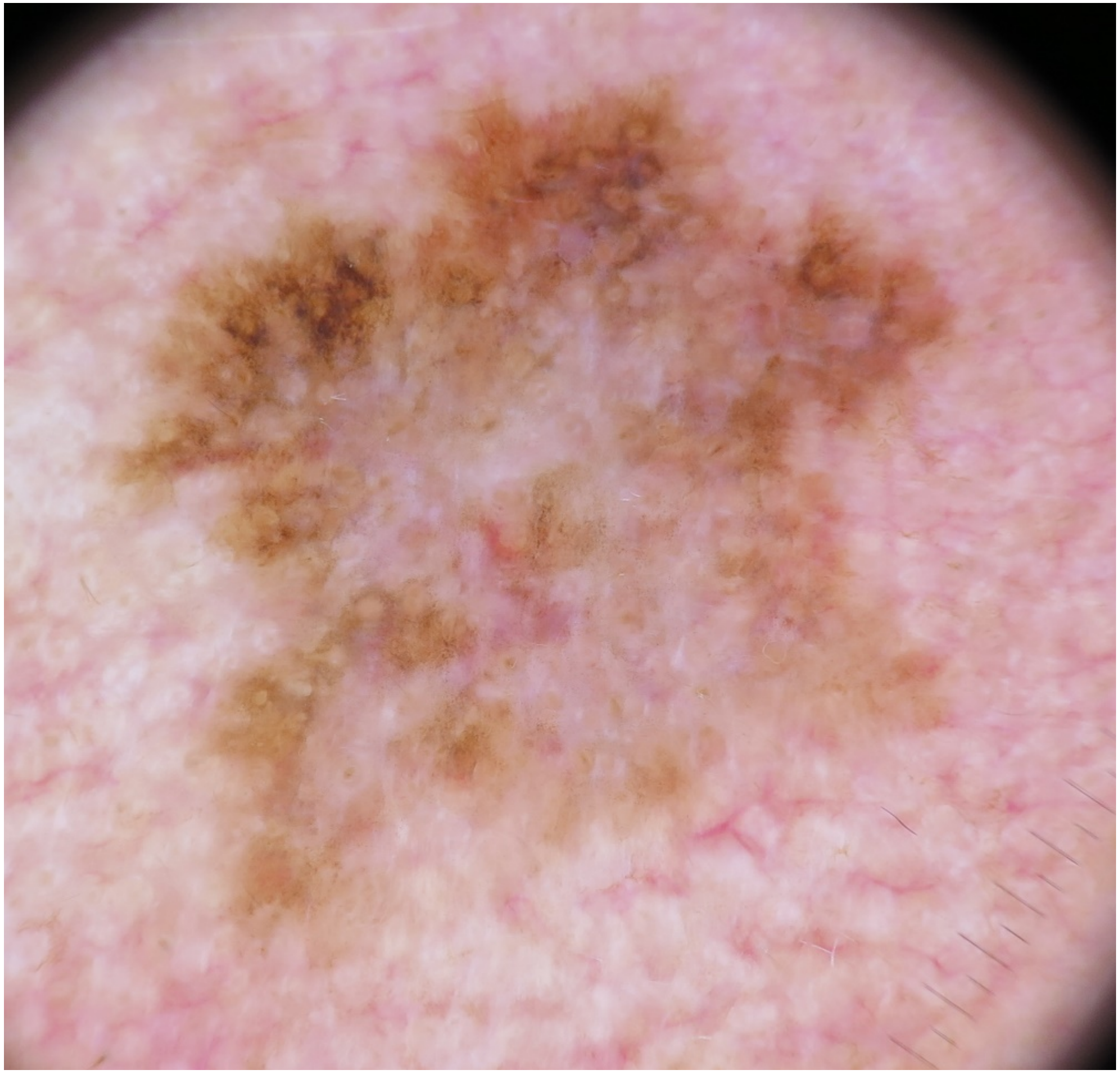

Location: Face

Invasive) Breslow interval: 0.1-0.5 mm

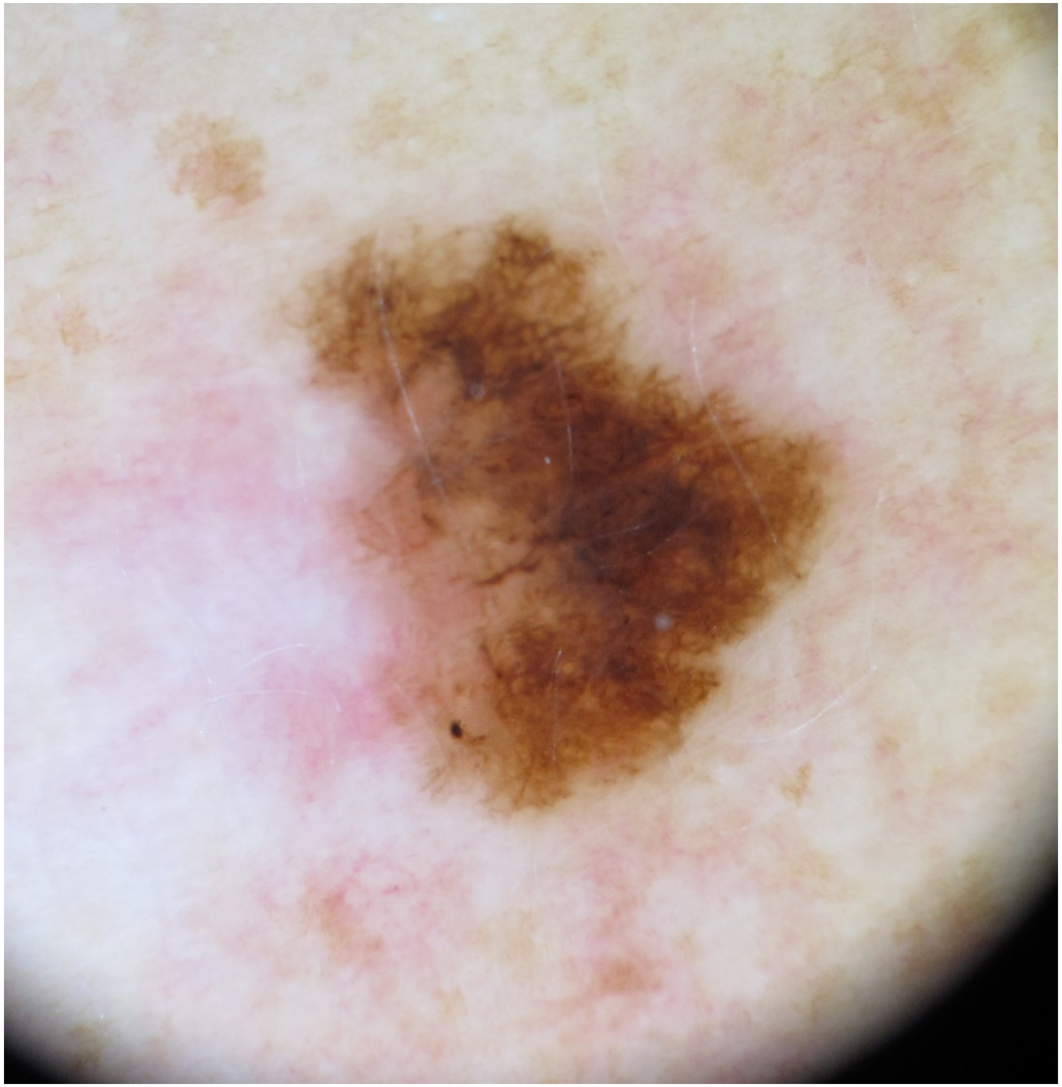

Location: Trunk

In situ melanoma

Case number 183

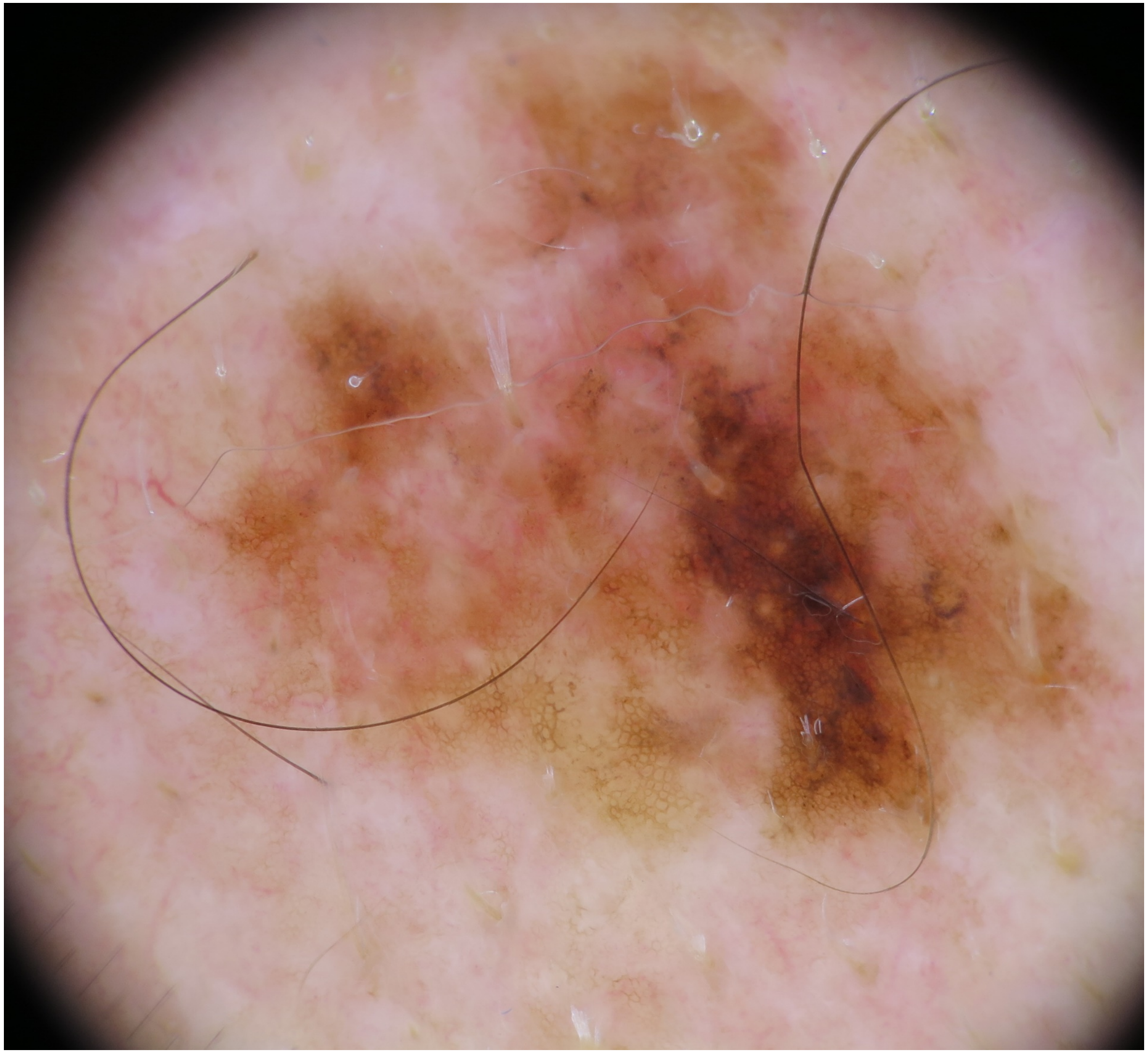

Location: Trunk

In situ melanoma

Case number 184

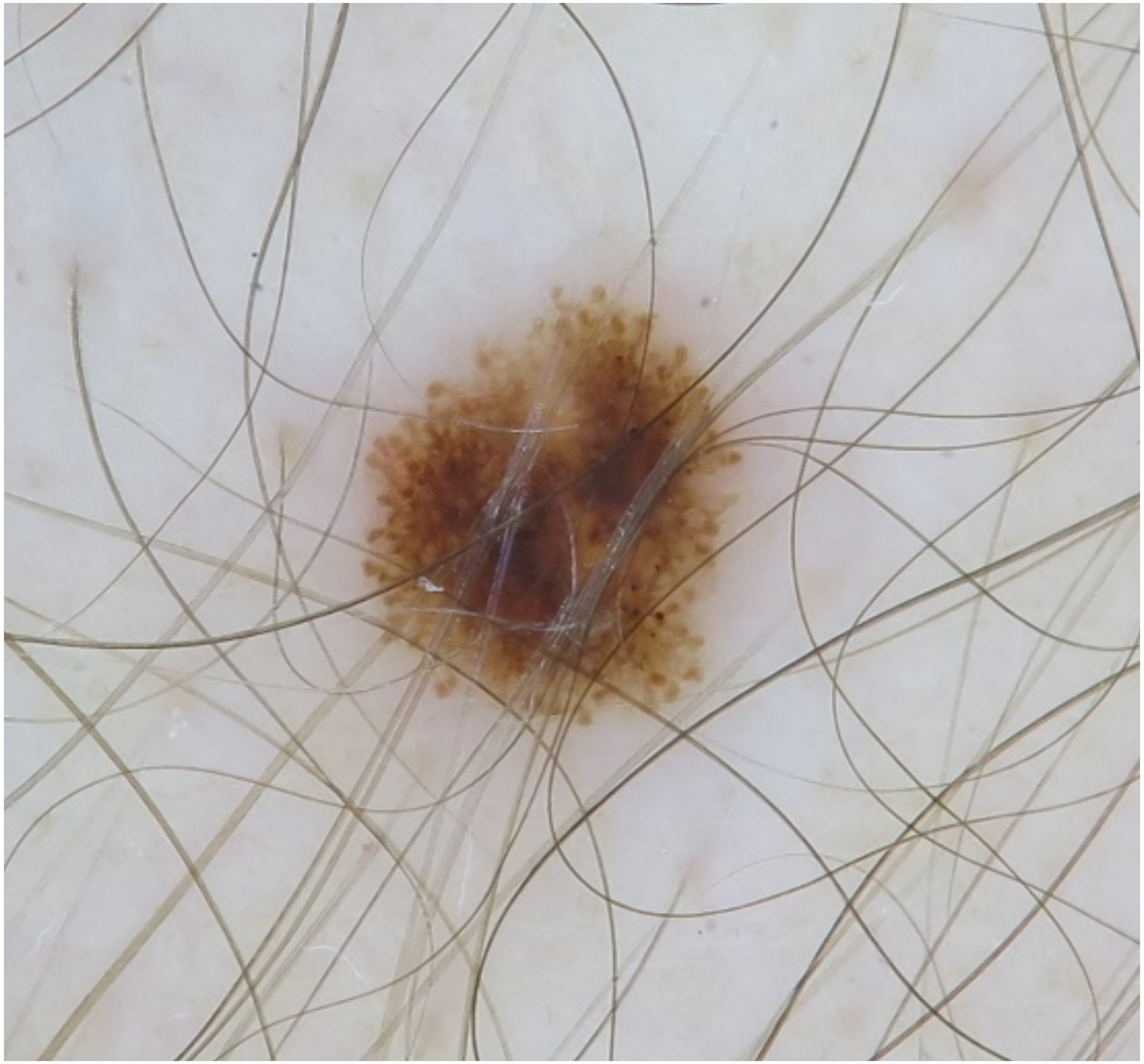

Location: Trunk

Invasive) Breslow interval: 0.1-0.5 mm
